# Genome Assembly of Three Shrub Mangroves in the Genus Acanthus Reveals Two Polyploidy Events and Expansion of Genes Linked to Root Adaptation in Coastal Habitats

**DOI:** 10.1093/gigascience/giaf162

**Published:** 2026-01-02

**Authors:** Wanapinun Nawae, Chaiwat Naktang, Peeraphat Paenpong, Duangjai Sangsrakru, Thippawan Yoocha, Sonicha U-thoomporn, Wasitthee Kongkachana, Poonsri Wanthongchai, Suchart Yamprasai, Chonlawit Samart, Sithichoke Tangphatsornruang, Wirulda Pootakham

**Affiliations:** National Center for Genetic Engineering and Biotechnology (BIOTEC), National Science and Technology Development Agency (NSTDA), Pathum Thani 12120, Thailand; National Center for Genetic Engineering and Biotechnology (BIOTEC), National Science and Technology Development Agency (NSTDA), Pathum Thani 12120, Thailand; National Center for Genetic Engineering and Biotechnology (BIOTEC), National Science and Technology Development Agency (NSTDA), Pathum Thani 12120, Thailand; National Center for Genetic Engineering and Biotechnology (BIOTEC), National Science and Technology Development Agency (NSTDA), Pathum Thani 12120, Thailand; National Center for Genetic Engineering and Biotechnology (BIOTEC), National Science and Technology Development Agency (NSTDA), Pathum Thani 12120, Thailand; National Center for Genetic Engineering and Biotechnology (BIOTEC), National Science and Technology Development Agency (NSTDA), Pathum Thani 12120, Thailand; National Center for Genetic Engineering and Biotechnology (BIOTEC), National Science and Technology Development Agency (NSTDA), Pathum Thani 12120, Thailand; Department of Marine and Coastal Resources, 120 The Government Complex, Thung Song Hong, Bangkok 10210, Thailand; Department of Marine and Coastal Resources, 120 The Government Complex, Thung Song Hong, Bangkok 10210, Thailand; Department of Marine and Coastal Resources, 120 The Government Complex, Thung Song Hong, Bangkok 10210, Thailand; National Center for Genetic Engineering and Biotechnology (BIOTEC), National Science and Technology Development Agency (NSTDA), Pathum Thani 12120, Thailand; National Center for Genetic Engineering and Biotechnology (BIOTEC), National Science and Technology Development Agency (NSTDA), Pathum Thani 12120, Thailand

**Keywords:** Acanthus, mangrove genomics, chromosome-level assembly, comparative genomics, gene family evolution, salt stress, root adaptation, coastal habitat

## Abstract

**Background:**

The genomes of mangrove *Acanthus* species have not been reported, despite their ecological and medicinal importance. Here, we generated reference genomes for three shrub mangroves in the genus *Acanthus* to clarify their whole-genome duplication and hybridization events and identify genomic features underlying their evolution.

**Results:**

Using PacBio and Hi-C data, we generated a chromosome-scale genome assembly of the recently identified allotetraploid species *Acanthus tetraploideus* (2*n* = 96). The genomes of diploid progenitors, *Acanthus ilicifolius* and *Acanthus ebracteatus* (2*n* = 48), were assembled from single-tube long fragment read data. We identified an *Acanthus*-specific whole-genome duplication (WGD) event that occurred ∼43 million years ago (Mya). Ancestral karyotype reconstruction revealed a shift in haploid chromosome number from 11 to 24 in the progenitors, following the WGD and subsequent chromosomal fission events. The hybridization that formed *A. tetraploideus* was estimated to have occurred 0.7–1.8 Mya. Phylogenomic and synteny analyses clearly showed that *A. tetraploideus* inherited subgenomes SG1 and SG2 from *A. ilicifolius* and *A. ebracteatus*, respectively. Gene structure and retention analyses revealed a smaller and more structurally flexible genome in *A. ebracteatus* and SG2 compared with *A. ilicifolius* and SG1. Gene family and machine learning analyses identified expansions in protein families related to Casparian strip formation, root development, and salt stress response. Several of these families were expanded in *A. ilicifolius* and SG1 but contracted in *A. ebracteatus* and SG2. These genomic patterns might have contributed to the establishment of *A. tetraploideus* within the habitat of *A. ebracteatus*. For all three species, population analysis revealed clear genetic divergence between samples from the eastern and western coasts of Thailand.

**Conclusions:**

These genome assemblies clarify the polyploidy and hybridization history of *Acanthus* and highlight gene family changes potentially associated with coastal root adaptation and habitat establishment in intertidal environments. This study provides valuable genomic resources and insights into the evolutionary adaptation of plants to intertidal environments.

## Introduction

**Figure 1:**
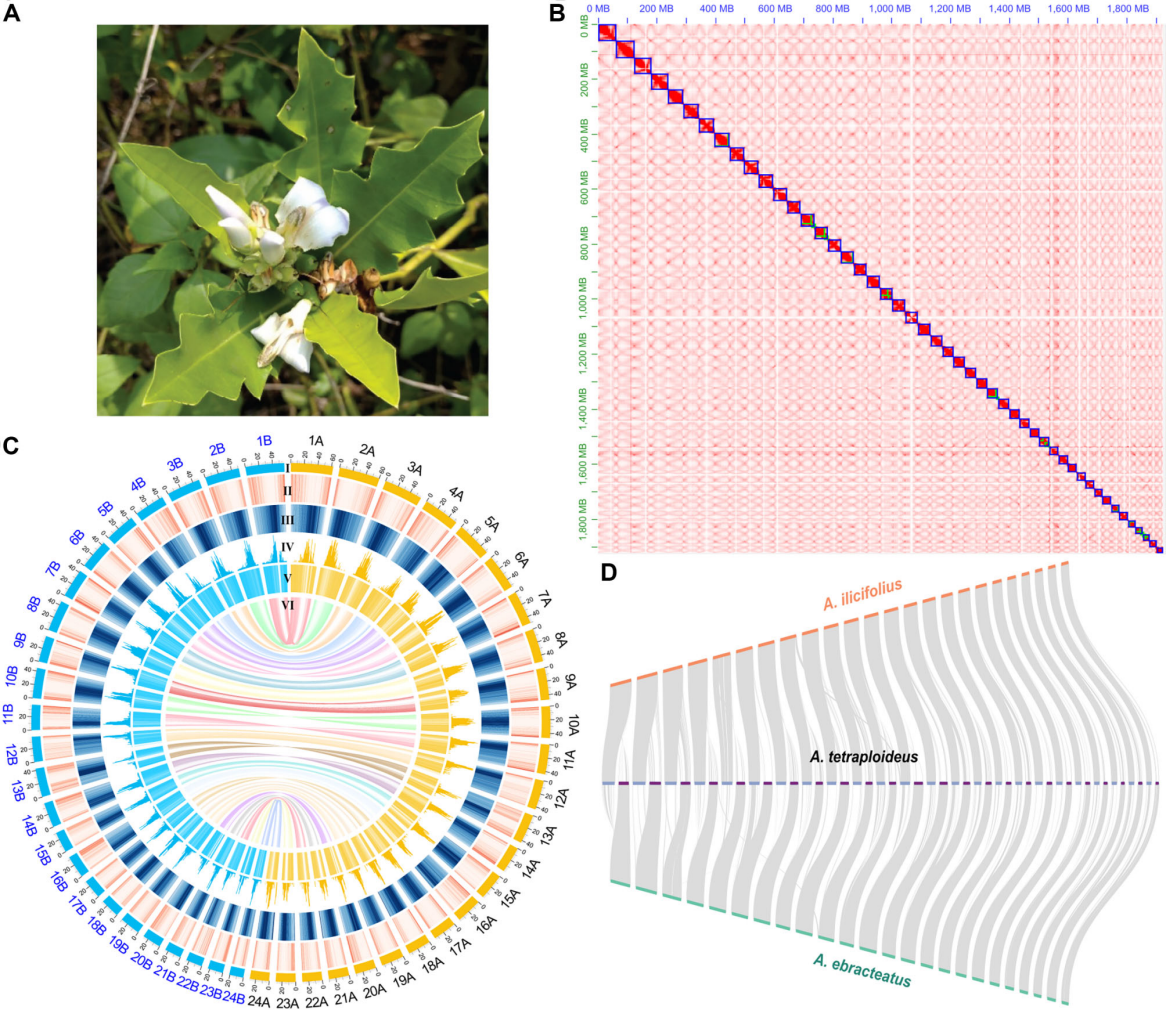
Chromosome-scale genome assembly of *Acanthus tetraploideus*. (A) Photograph of *A. tetraploideus* showing its characteristic lanceolate, spiny, leathery leaves and pale violet flowers. (B) Hi-C contact matrix of the *A. tetraploideus* genome assembly showing strong intrachromosomal interactions and clear chromosome boundaries, confirming high-quality scaffolding of 48 chromosomes. (C) Circos plot illustrates structural and sequence features of the *A. tetraploideus* genome and syntenic relationships between subgenomes SG1 (orange) and SG2 (light blue). The outermost ring (I) depicts the 48 chromosomes of *A. tetraploideus*, arranged as 24 homeologous pairs. The second (II) and third (III) tracks show gene density and repeat density, respectively. The fourth track (IV) presents the distribution of SG1- and SG2-specific *k*-mers, shown in yellow and blue. The fifth track (V) illustrates sequence similarity between SG1 and *A. ilicifolius* (yellow) and between SG2 and *A. ebracteatus* (blue). At the center, the innermost layer (VI) contains colored ribbons linking syntenic blocks between homeologous chromosome pairs. (D) Sankey-style diagram illustrating genome-wide syntenic relationships between *A. tetraploideus* and its progenitor species, where SG1 (blue bars) corresponds to *A. ilicifolius* (orange bars) and SG2 (violet bars) corresponds to *A. ebracteatus* (green bars), indicating their respective parental contributions to the allotetraploid genome.

The genus *Acanthus* (family Acanthaceae) consists of approximately 30 species of flowering plants distributed across tropical and subtropical regions worldwide [1]. *Acanthus* species are highly adaptive, as indicated by their significant diversity in morphology and habitat preferences, ranging from terrestrial to mangrove environments [2]. While many *Acanthus* species are terrestrial, three mangrove species—*Acanthus ilicifolius, Acanthus ebracteatus*, and *Acanthus volubilis*—inhabit intertidal zones where saltwater and freshwater converge [1]. These species have long been used as medicinal plants across Asia and Oceania [3]. Morphologically, *A. volubilis* is clearly different from the other two species. In contrast, *A. ilicifolius* and *A. ebracteatus* share highly similar leaf morphology, characterized by lanceolate, spiny, and leathery leaves (Fig. 1A). However, their floral characteristics differ, as *A. ilicifolius* produces violet flowers with bracteoles, while *A. ebracteatus* bears smaller white flowers. Recent phylogenetic and biogeographic investigations introduced a new *Acanthus* species, *Acanthus tetraploideus*, with mixed phenotypic and genotypic characteristics from *A. ilicifolius* and *A. ebracteatus* [1]. *A. tetraploideus* has 96 chromosomes (2*n* = 96), double the chromosome number of *A. ilicifolius* and *A. ebracteatus* (2*n* = 48) [1]. Exploring the genomes of these three species will provide valuable insights into the evolution of mangrove species within challenging and dynamic coastal habitats.

The genome assembly of *Acanthus* species has not yet been reported, although several sequencing efforts have been made to understand the genetics of this lineage. For example, transcriptomic analyses identified positively selected genes that were related to salt, heat, and ultraviolet stress tolerance in *A. ilicifolius* when compared to its terrestrial relative *Acanthus leucostachyus* [2]. *A. ilicifolius* likely diverged from *A. leucostachyus* approximately 11.6 to 22.1 million years ago (Mya), and the selection of these genes was suggested to be associated with its adaptation to intertidal zones [2]. Additionally, phylogenetic analyses based on chloroplast genomes, eight nuclear genes, and transcriptome data have identified *A. ilicifolius* and *A. ebracteatus* as the putative progenitors of the allotetraploid *A. tetraploideus* genome [1, 4]. These studies recommended that whole-genome sequencing is necessary for accurately identifying the origin of this new allotetraploid species [1, 4]. Genome sequencing technologies, including PacBio, Hi-C, and linked-read sequencing, have significantly advanced our understanding of complex plant genomes and their evolution [5]. These technologies have been applied to identify salinity tolerance genes and intertidal adaptations of *Avicennia marina* [6], uncover whole-genome duplication (WGD) in *Ceriops tagal* [7], and reveal the origin of *Bruguiera hainesii* from the hybridization of *Bruguiera gymnorhiza* and *Bruguiera cylindrica* [8].

In this study, we generate a chromosome-level genome assembly of the tetraploid species *A. tetraploideus* (NCBI:txid3383851) using PacBio HiFi and Hi-C sequencing data. Additionally, we assemble the diploid genomes of *A. ilicifolius* (NCBI:txid328098) and *A. ebracteatus* (NCBI:txid241842), the candidate parental species of the tetraploid, using single-tube long fragment read (stLFR) sequencing. These high-quality genomic resources enable comprehensive investigations into the evolutionary history, polyploidization events, and adaptive mechanisms of *Acanthus* species. Our work marks a significant advancement in mangrove genomics and provides a valuable foundation for future research on the conservation and sustainable use of these ecologically and economically important plants.

## Materials and Methods

### Plant materials and nucleic acid isolation

Young leaf tissues were collected from mature individuals of *A. ilicifolius, A. ebracteatus*, and *A. tetraploideus* in natural mangrove habitats in Thailand. Samples of *A. tetraploideus* and *A. ebracteatus* were collected from Samut Sakhon province at coordinates 13°30′24.9″N, 100°16′15.2″E and 13°30′32.4″N, 100°15′16.1″E, respectively. *A. ilicifolius* leaves were obtained from Phuket province at 8°09′53.6″N, 98°18′17.2″E. All leaf samples were immediately flash-frozen in liquid nitrogen in the field and stored at –80°C until processing. High-molecular-weight genomic DNA was extracted using the QIAGEN Genomic-tip 100/G kit following the manufacturer’s protocol. DNA quality and integrity were assessed using a Pippin Pulse Electrophoresis System (Sage Science) and quantified with a Qubit fluorometer prior to library preparation.

For transcriptome sequencing, total RNA was isolated from leaf tissues collected from the same individual used for genome sequencing, following the protocol of Pootakham et al. [9]. Poly(A) mRNA was enriched using the Dynabeads mRNA Purification Kit (Thermo Fisher Scientific). The integrity of RNA samples was evaluated using the Fragment Analyzer System (Agilent) prior to library construction.

We also collected leaf samples from 90 accessions of *A. ilicifolius, A. ebracteatus*, and *A. tetraploideus* across mangrove forests in 14 provinces of Thailand. Sampling sites included Chumphon (CPN), Nakhon Si Thammarat (NST), Phatthalung (PLG), Phetchaburi (PBI), Samut Songkhram (SKM), Samut Sakhon (SKN), Surat Thani (SNI), and Trat (TRT) along the Gulf of Thailand and Krabi (KBI), Phang Nga (PNG), Phuket (PKT), Ranong (RNG), Satun (STN), and Trang (TRG) along the Andaman coast. All leaf tissues were immediately flash-frozen in liquid nitrogen and stored at –80°C until processing. Genomic DNA was extracted using the CTAB method as described by Pootakham et al. [10], and its quality was assessed using a Qubit fluorometer (Thermo Fisher Scientific). The genome sizes of all samples used in this study were estimated using flow cytometry on the BD Accuri C6 Plus system (BD Biosciences) and the maize genome as a reference standard.

### Library preparation and sequencing

High-molecular-weight genomic DNA was sheared to ∼15 kb using the Megaruptor 2 (Diagenode, Liège, Belgium) system. SMRTbell libraries were prepared with the SMRTbell Express Template Prep Kit 2.0 (PacBio). The libraries were purified with AMPure PB beads and size-selected (15–18 kb) using the Sage ELF system. Quality and quantity were assessed using FEMTO Pulse and Qubit. Final libraries were bound to Sequel II polymerase and sequenced on the PacBio Sequel II platform (RRID:SCR_017990) using an 8-M SMRT Cell with 1,800-minute movies. To generate chromosome-scale scaffolds, a Dovetail Omni-C (Hi-C) library was prepared by Dovetail Genomics. The protocol involved cross-linking chromatin with formaldehyde, digesting with DNase I, repairing ends, and ligating biotinylated adapters, followed by proximity ligation and purification. Biotinylated DNA fragments were isolated using streptavidin beads, and sequencing libraries were constructed using NEBNext Ultra (New England Biolabs) reagents. The library was sequenced on an Illumina HiSeq X (RRID:SCR_016385). PacBio HiFi and Dovetail Omni-C library preparation, quality control, and sequencing were carried out by BMKGENE (Biomarker Technologies) following the provider’s standard protocols.

For linked-read sequencing of *A. ilicifolius* and *A. ebracteatus*, high-molecular-weight genomic DNA was used to construct stLFR libraries using the MGIEasy stLFR Library Prep Kit (MGI Tech). For transcriptome sequencing, polyadenylated mRNA extracted from leaf tissue was used to prepare libraries with the MGIEasy RNA Library Prep Kit v3.0 (MGI Tech). To assess genetic variation across populations, RADseq libraries were constructed from individual samples using the MGIEasy RAD Library Prep Kit (MGI Tech). All libraries were sequenced on the MGI DNBSEQ-G400 platform (RRID:SCR_017980).

### Genome assembly and annotation

For *A. tetraploideus*, the genome was assembled using PacBio HiFi long reads and Hi-C scaffolding. HiFi reads were first assembled with Hifiasm 0.25 (RRID:SCR_021069) [11] in Hi-C mode to generate contigs. The Hi-C reads were mapped to the contigs following the pipeline described in the Dovetail Omni-C document [12]. Scaffolding was subsequently performed using YaHS 1.2 (RRID:SCR_022965) [13] with default parameters. Juicebox was used to curate the scaffolding results and visualize the Hi-C contact map. Only the chromosome sequences were used in downstream analysis. BUSCO 5.2 (RRID:SCR_015008) [14] was used to assess assembly completeness.

The stLFR reads from *A. ilicifolius* and *A. ebracteatus* were assembled using stLFRdenovo with default parameters [15]. D-GENIES 1.5 (RRID:SCR_018967) [16] was used to visualize dot plots of pairwise whole-genome alignments between the allotetraploid *A. tetraploideus* genome with *A. ilicifolius* and *A. ebracteatus* genomes. We used RagTag v2.1 (RRID:SCR_027293) [17], a widely applied scaffolding tool with robust filtering steps, to scaffold *A. ilicifolius* and *A. ebracteatus* contigs using the chromosomes of the corresponding subgenomes as references. RagTag employs minimap2 (RRID:SCR_018550) with a default option (-x asm5), optimized for high-quality long-read assemblies, to map the query to the reference and does not alter the query sequences, but only orders and orients them, joining with gaps where necessary.

For gene prediction, genome annotation was performed using BRAKER2 3.0 (RRID:SCR_018964) [18], which integrated RNA sequencing (RNA-seq) evidence and *ab initio* predictions. RNA-seq data were used as extrinsic evidence to train the gene models. Viridiplantae protein sequences from the OrthoDB 11 database (RRID:SCR_011980) [19] were incorporated to support and refine gene predictions. Repeat masking was carried out using RepeatModeler (RRID:SCR_015027) and RepeatMasker (RRID:SCR_012954) prior to annotation.

### Genome analysis

Assembly statistics, including total assembly size, contig/scaffold N50, and number of contigs were generated using QUAST 5.3 (RRID:SCR_001228) [20]. SubPhaser 1.2 [21] was used to assign homoeologous chromosome pairs, which were obtained from D-GENIES alignments, into subgenomes SG1 and SG2. Synteny blocks were then detected using MCScanX 1.0 (RRID:SCR_022067) [22] and JCVI 1.5 (RRID:SCR_021641) [23] with their default parameters. To show the relationship between *A. tetraploideus* chromosomes and progenitor sequences, JCVI was used to visualize pairs of matched synteny blocks. Circos 0.52 (RRID:SCR_011798) [24] was used to show the relationship between subgenomes SG1 and SG2. To investigate synonymous substitution rates (Ks) among duplicated and orthologous gene pairs, collinear blocks were first identified using the WGDI pipeline [25]. Ks values for each gene pair within these blocks were calculated using MUSCLE 5.3 (RRID:SCR_011812) [26] and the yn00 program of the PAML 4.9 package (RRID:SCR_014932) [27] under the WGDI environment [25]. For each block, both the average and median Ks values across collinear gene pairs were extracted and fitted with Gaussian Mixture Models (GMMs) to visualize Ks distributions. To minimize the influence of recent tandem duplications, which can generate spurious low Ks values, paralogous pairs located within 200 bp on the same chromosome were excluded prior to plotting. This WGDI default threshold was used to remove ultra-proximal tandem duplicates while retaining true WGD-derived paralogs. WGDI was also employed to reconstruct ancestral chromosomes of all studied *Acanthus* species based on sorted dot plots of shared synteny blocks. Additionally, gene retention from the parental genomes on *A. tetraploideus* chromosomes was identified using WGDI. The Liftoff program was used to transfer the annotations from reference to target sequences. The completeness of the sequences within the transferred annotation region was checked to investigate possible structural aberrations of unretained genes. Homologous genes used as inputs for MCScanX, JCVI, and WGDI were identified using BLASTP (RRID:SCR_001010) with e-value cutoff of 10^−10^.

### Comparative genomics

Protein sequences from *A. tetraploideus, A. ilicifolius, A. ebracteatus*, and an additional 16 plant species, including *Nypa fruticans* [28], *Lumnitzera racemosa* [29], *Combretum micranthum* [29], *Sonneratia alba* [30], *Sonneratia caseolaris, Bruguiera parviflora* [31], *Rhizophora apiculate* [32], *Kandelia obovata* [33], *Ceriops tagal* [7], *Aegiceras corniculatum* [34], *Olea europaea* (GCF_002742605.1), *Rehmannia glutinosa* [35], *Salvia hispanica* (GCF_023119035.1), *A. marina* [36], *Strobilanthes cusia* [37], and *Andrographis paniculata* [38], were used to identify orthologous groups (orthogroups) using OrthoFinder 2.5 (RRID:SCR_017118) [39]. The protein sequences of single-copy orthologs identified by OrthoFinder were aligned using MUSCLE 5.3 (RRID:SCR_011812) [26]. Poorly aligned regions were trimmed by trimAl 1.5 (RRID:SCR_017334) [40] with the heuristic mode (-automated1 option). A phylogenetic tree was inferred from the processed alignment using RAxML-NG 1.2 (RRID:SCR_022066) [41] under the best-fit substitution model determined by ModelTest-NG 0.1 (RRID:SCR_026633) [42]. The tree topology was evaluated using 1,000 bootstrap replicates in RAxML-NG and further validated by comparison with previously published phylogenetic trees of mangroves and Acanthaceae. We used MCMCTree implemented in the PAML 4.9 (RRID:SCR_014932) [27] to calculate divergence times among species in the tree based on fossil calibration times obtained from and the Timetree database (RRID:SCR_021162) [43] and associated references therein.

### Estimation of the timing of whole-genome duplication and hybridization events

The timing of the WGD event was estimated from peaks in the Ks distribution, following approaches described in recent genomic studies [44, 45], based on the neutral theory of molecular evolution [46]. In this framework, divergence time (*T*) is related to the number of synonymous substitutions per synonymous site (Ks) and the neutral substitution rate per site per year (μ), according to


\begin{eqnarray*}
T = \frac{{Ks}}{{2\mu }}
\end{eqnarray*}

In this study, the divergence between *A. ilicifolius* and *A. ebracteatus* was used as a calibration point to estimate *μ*. The divergence time (*T_speciation_*) was independently inferred using MCMCTree, and the Ks peak corresponding to the WGD event (*Ks_WGD_*) was scaled relative to the Ks value at the progenitor divergence peak (*Ks_speciation_*) to estimate the timing of the WGD. Assuming both events follow the same lineage-specific substitution rate, their relationship can be expressed as


\begin{eqnarray*}
2\mu = \frac{{K{s}_{\textit{speciation}}}}{{{T}_{\textit{speciation}}}} = \frac{{K{s}_{WGD}}}{{{T}_{WGD}}}
\end{eqnarray*}

Rearranging gives


\begin{eqnarray*}
{T}_{WGD} = \frac{{K{s}_{WGD}}}{{K{s}_{\textit{speciation}}}} \times {T}_{\textit{speciation}}
\end{eqnarray*}

This internally calibrated approach allowed the estimation of the timing of the WGD event using relative Ks values and the independently inferred speciation time.

To estimate the timing of allotetraploidization, we followed a previously established analytical framework [47, 48]. Transposable element (TE) divergence profiles for SG1 and SG2 were generated using RepeatMasker (RRID:SCR_012954) and plotted as distributions (Supplementary Fig. S1). The initial separation of TE divergence curves indicated the progenitor speciation event, and their later convergence, after independent evolution, marked the genome merger when both subgenomes began sharing a similar TE substitution rate. The TE divergence values at these two points (*D_speciation_* and *D_merger_*, respectively) were then calibrated against *T_speciation_* to estimate the timing of allotetraploidization using an equation analogous to the Ks-based estimation:


\begin{eqnarray*}
{T}_{\textit{merger}} = \frac{{{D}_{\textit{merger}}}}{{{D}_{\textit{speciation}}}} \times {T}_{\textit{speciation}}
\end{eqnarray*}

### Gene family expansion/contraction analysis and machine learning–based selection of lineage-specific gene families

Gene family size changes were analyzed using CAFE 5 (RRID:SCR_005983) [49]. The OrthoFinder (RRID:SCR_017118) protein count table and the calibrated phylogenetic tree were provided as input. A global λ parameter was estimated, and families with significantly expanded or contracted sizes (*P* < 0.05) were identified.

To identify orthogroups that distinguish the Acanthus lineage (class A) from non-Acanthus species (class B), we implemented a multistep analytical pipeline incorporating phylogenetic filtering, statistical testing, and machine learning–based feature selection. The species phylogenetic tree and the protein count matrix from OrthoFinder were used as inputs for the analysis. First, gene count data were normalized and screened to exclude extremely high-count outliers. The *z*-scores were calculated for each orthogroup, and preoutliers were defined as those showing unusually high expression in only one or two species. To enrich the feature space with phylogenetically informative signals, Blomberg’s *K* and Pagel’s λ were calculated for each orthogroup using the phylosig() function in the R package phytools 2.0 (RRID:SCR_015502) [50]. Orthogroups with intermediate conservation signals (*K* > 1 and λ > 0.5) were retained for downstream analysis. After filtering out orthogroups with zero counts in all selected species, we performed Welch’s *t*-tests by applying a relaxed threshold (*P* < 0.1) to preliminarily screen for orthogroups showing a differential copy number between the two classes. The retained orthogroups were then subjected to recursive feature elimination with three classification algorithms—logistic regression, random forest, and gradient boosting—to prioritize orthogroups with the strongest discriminatory power. These models were implemented with the scikit-learn library in Python. Feature importance scores were derived from model coefficients (logistic regression) or feature importance values (tree-based models). For each classifier, the top 20 ranked orthogroups were designated as lineage-associated gene families.

### Single-nucleotide polymorphism (SNP) calling and population structure analysis

Paired-end RADseq reads from 90 accessions of *A. ilicifolius, A. ebracteatus*, and *A. tetraploideus* were mapped to their respective genome assemblies using BWA v0.7.17 (RRID:SCR_010910) [51] with default settings. Single-nucleotide polymorphisms (SNPs) were called separately for each species using the GATK v4.1.4.1 HaplotypeCaller (RRID:SCR_001876) [52]. Only high-quality biallelic SNPs were retained after filtering with the following thresholds: QUAL ≥30, read depth between 10× and 200×, minor allele frequency (MAF) ≥0.1, and missing data ≤5%.

Population structure was inferred from the filtered SNP dataset using STRUCTURE v2.3.4 (RRID:SCR_017637) under a Bayesian model-based framework [53]. Twenty independent replicates were performed for each *K* value ranging from 1 to 10, with a burn-in of 100,000 and 500,000 Markov Chain Monte Carlo (MCMC) iterations under an admixture model with correlated allele frequencies. The most likely number of genetic clusters (*K*) was determined using the Δ*K* method implemented in Structure Harvester (RRID:SCR_017636) [54, 55]. Based on the optimal *K* value, CLUMPP v1.1.2 [56] was used to average individual assignment probabilities across replicates.

## Results

### Genomes of *A. tetraploideus* and its progenitors

We generated a high-quality genome assembly of *A. tetraploideus* using a combination of PacBio HiFi and Hi-C sequencing data. PacBio HiFi sequencing produced 63 Gb of long-read data, with an average read length of 14.36 kb and a sequencing depth of 33×. The HiFi data assembly yielded a total contig length of 1.95 Gb and a contig N50 of 42.67 Mb, matching the estimated genome size (Supplementary Table S1; sample SKN-At-01). The contigs were scaffolded with a total of 230 Gb of Hi-C data into 48 chromosomes, with a total length of 1.92 Gb and a scaffold N50 of 43.75 Mb (Fig. 1B and Supplementary Table S2). Telomeric repeats (AAACCCT) were identified at both ends of 44 chromosomes, at a single end of three chromosomes, and absent from only one chromosome (Supplementary Table S3). All chromosomes had repeat-dense central regions, with annotated genes located outside these areas (Fig. 1C). BUSCO analysis indicated that 99.2% of conserved Embryophyta genes were present (Supplementary Table S4), demonstrating the high completeness of the *A. tetraploideus* assembly. These results indicated a high completeness level of the assembled *A. tetraploideus* genome. BUSCO analysis revealed that duplicated genes accounted for 95.5% of the *A. tetraploideus* genome, reflecting the polyploid nature of the assembled genome. Further analysis of orthologous chromosome pairs using SubPhaser identified two clearly separated subgenomes, SG1 (1.03 Gb) and SG2 (0.89 Gb). Synteny block analysis confirmed a 1:1 relationship between SG1 and SG2 across the genome (Fig. 1C).

The genomes of *A. ilicifolius* and *A. ebracteatus*, the two putative progenitors of *A. tetraploideus*, were sequenced using the stLFR technique. The assembled genome sizes were 0.98 Gb for *A. ilicifolius* and 0.89 Gb for *A. ebracteatus* (Supplementary Tables S5–S6). These numbers were also consistent with the genome sizes estimated by flow cytometry (Supplementary Tables S7–S8). BUSCO analysis indicated that both assemblies contained 98% of Embryophyta conserved genes, with only 11% classified as duplicated genes (Supplementary Tables S9–S10). Although the overall identity values were modest, genome alignments indicated that SG1 of *A. tetraploideus* shared a greater similarity with the *A. ilicifolius* assembly, whereas SG2 showed higher similarity with *A. ebracteatus* (Supplementary Table S11). The modest identity values reflected accumulated sequence divergence within long stretches of collinearity between each subgenome and its corresponding progenitor genome (Fig. 1D and Supplementary Table S11). Based on these matches, the contigs of *A. ilicifolius* and *A. ebracteatus* were scaffolded using SG1 and SG2 as reference sequences, respectively. The scaffolding resulted in 24 pseudochromosomes for each species, covering 93% and 94% of the initial lengths of the *A. ilicifolius* and *A. ebracteatus* assemblies, respectively. The genome annotation revealed 61,044, 30,210 and 30,799 protein-coding gene models in *A. tetraploideus, A. ilicifolius*, and *A. ebracteatus*, respectively (Supplementary Tables S12–S14). In *A. tetraploideus*, BUSCO analysis indicated that the annotated protein set contained 98% of the conserved Embryophyta genes, with 92% classified as duplicated (Supplementary Table S15). In *A. ilicifolius* and *A. ebracteatus*, the annotated proteins represented 97% of conserved genes, with 26% identified as duplicated (Supplementary Tables S16–S17). Synteny analysis of conserved gene order among genomes clearly demonstrated that *A. tetraploideus* inherited SG1 from *A. ilicifolius* and SG2 from *A. ebracteatus* (Fig. 1D).

### Ancestral karyotypes

**Figure 2:**
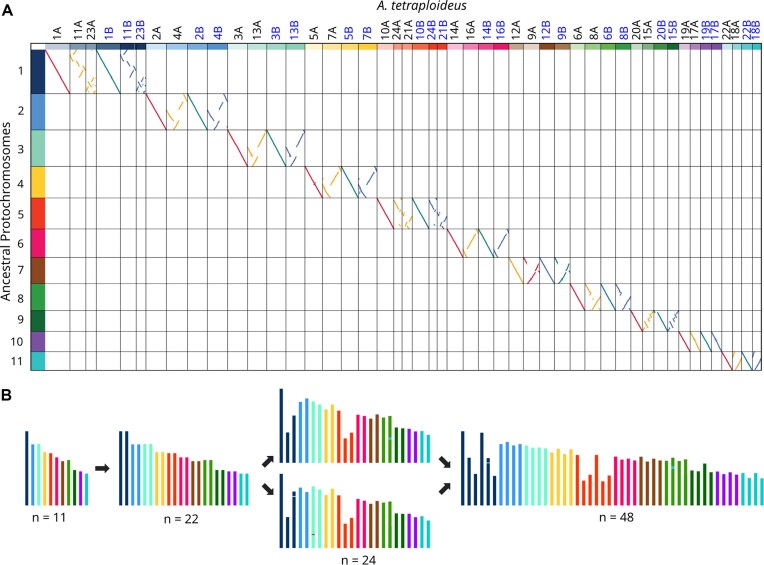
Ancestral karyotype reconstruction and chromosome evolution in *Acanthus tetraploideus*. (A) Alignment of *A. tetraploideus* chromosomes (columns) to 11 reconstructed ancestral protochromosomes (rows). Chromosomes belonging to subgenome SG1 are labeled in black (top) and shown with red and yellow alignment lines, while those of subgenome SG2 are labeled in blue and shown with green and blue alignment lines. Diagonal alignments indicate homeologous chromosome pairs derived from the same ancestral protochromosome. (B) Schematic model illustrating chromosome evolution in the *Acanthus* lineage. The ancestral karyotype (*n* = 11) underwent a whole-genome duplication (WGD), yielding a diploid genome with *n* = 22 chromosomes. Subsequent chromosomal fissions (see alignments between chromosome 1 with chromosomes 11 and 23, as well as between chromosome 10 with chromosomes 21 and 24) increased the haploid chromosome number to *n* = 24. Hybridization between two diploid progenitors with this karyotype led to the formation of the allotetraploid species *A. tetraploideus* (*n* = 48). Mixed-color bars in the final karyotype indicate chromosomal translocations and rearrangements.

A dot plot from pairwise comparisons of all chromosomes within the *A. tetraploideus* genome revealed 11 protochromosomes in the ancestral karyotype, each defined by conserved intervals of syntenic blocks (Supplementary Fig. S3). This number of protochromosomes corresponded to that of the ancestral karyotype in Lamiales [57]. Chromosome mapping indicated that 18 chromosomes in each subgenome (SG1 and SG2) aligned with nine ancestral chromosomes in a 2:1 ratio. These results represented the signal of a WGD event experienced by the ancestor of the Acanthus lineage. The dot plot also showed that most duplicated chromosome pairs displayed extensive rearrangements, with numerous interleaved syntenic fragments, indicating that the WGD occurred long before the divergence of the progenitor lineages (Fig. 2A). Interestingly, the remaining six chromosomes in each subgenome aligned with the remaining two ancestral chromosomes in a 3:1 ratio (Fig. 2A). For example, ancestral chromosome 1 aligned with chromosomes 1A, 11A, and 23A of SG1, as well as 1B, 11B, and 23B of SG2. In SG1, for example, chromosome 11A corresponded to approximately two-thirds of 1A, while 23A aligned with the remaining region, indicating that the two duplicated chromosomes were fragmented in the common ancestor of *A. ilicifolius* and *A. ebracteatus*.

Based on these results, we propose that the ancestral lineage of *Acanthus* and related species originally had 11 chromosomes (Fig. 2B). A WGD event doubled the haploid chromosome number to 22 (2*n* = 44). Subsequent chromosomal fissions increased the haploid chromosome number to 24 (2*n* = 48). This karyotype has been maintained in both *A. ilicifolius* and *A. ebracteatus*. Hybridization between these two species further raised the haploid chromosome number to 48 (2*n* = 96), resulting in the formation of the allotetraploid *A. tetraploideus*. The karyotype analysis also showed small segments of different colors embedded within the main bodies of some representative chromosomes of *A. tetraploideus, A. ilicifolius*, and *A. ebracteatus*, suggesting that the genomes underwent subtle chromosomal translocations and structural rearrangements (Fig. 2B). These structural modifications indicated limited genomic exchange between the two subgenomes following polyploid formation, implying that *A. tetraploideus* was a recently formed species.

### Genome evolution

**Figure 3:**
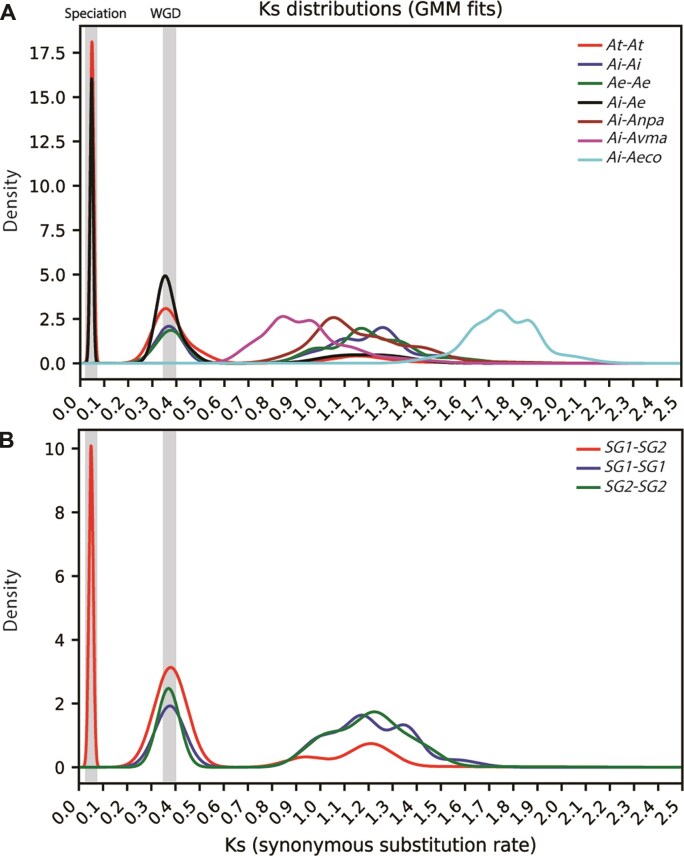
Ks distributions showing speciation and WGD signals in the *Acanthus* lineage. (A) Ks distributions of paralogous and orthologous gene pairs among *Acanthus* species. Two major peaks correspond to a recent speciation event (Ks ≈ 0.05) and an older whole-genome duplication (WGD; Ks ≈ 0.35). (B) Ks distributions between and within subgenomes of *A. tetraploideus*. The SG1–SG2 comparison shows the same WGD peak as observed in the diploid progenitors, indicating that the WGD occurred before hybridization and subgenome divergence.

Synteny analysis indicated that the WGD event preceded the hybridization between *A. ilicifolius* (Ai) and *A. ebracteatus* (Ae) that formed *A. tetraploideus* (At). To clarify the sequence of genome duplication and divergence events, we examined the distributions of synonymous divergence (Ks) values for orthologous (interspecific) and paralogous (intraspecific) gene pairs from syntenic blocks (Fig. 3). The Ai–Ai, Ae–Ae, and At–At paralogous, as well as the Ai–Ae orthologous, comparisons each showed a Ks peak at approximately 0.35–0.40, indicating that *A. ilicifolius* and *A. ebracteatus* shared a recent lineage-specific WGD event (Fig. 3A). In addition, a broader peak centered around ∼1.2–1.4 was consistently detected in all comparisons, suggesting older duplication events. The Ai–Ae orthologous comparison displayed an additional Ks peak at approximately 0.05, corresponding to their recent speciation event. This peak was also observed in the At–At paralogous comparison, reflecting the coexistence of both progenitor genomes within the allotetraploid nucleus. Correspondingly, the SG1–SG2 subgenome comparison revealed the same 3-peak pattern (Fig. 3B).

*A. ilicifolius* showed a Ks peak of ∼1.0–1.3 when compared with the terrestrial relative *A. paniculata* (Ap) and ∼0.9–1.2 with the mangrove relative *A. marina* (Am) within the Acanthaceae family (Fig. 3A). In addition, a Ks peak centered around ∼1.6–1.8 was observed in the comparison between *A. ilicifolius* and *A. corniculatum* (Ac), which was a distantly related mangrove species within the Asterids clade. Together, these results indicate that the Ks peak at ∼0.35–0.40 represents an *Acanthus*-specific WGD event that occurred after the divergence of the genus from its terrestrial and mangrove relatives, but before the speciation and subsequent hybridization event (Ks ≈ 0.05).

### The expansion of chromosome sequence

**Figure 4:**
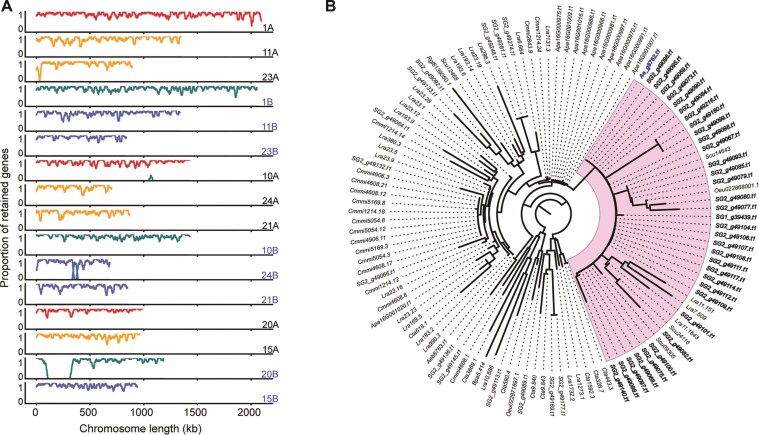
Subgenome-specific gene retention and phylogenetic relationships of duplicated genes on chromosome 20B. (A) Gene retention ratios along selected chromosomes of *A. tetraploideus*. Ratios represent the proportion of genes retained from each progenitor, ranging from 0 to 1. Red and yellow lines indicate genes retained from the 2 groups of WGD-derived scaffolds in *A. ilicifolius*, whereas green and blue lines represent genes inherited from *A. ebracteatus* (see matched colors in Fig. 2). Gene retention patterns across all *A. tetraploideus* chromosomes are shown in Supplementary Fig. S4. (B) Phylogenetic tree of a representative gene family containing multiple paralogous copies within the extended sequence of chromosome 20B (highlighted in pink). The paralogous gene copies in *A. tetraploideus* (subgenome SG2) are shown in bold black, and the orthologous gene in *A. ebracteatus* is shown in bold blue. Homologous genes from *L. racemosa* (Lra), *B. parviflora* (Bpa), *C. tagal* (Cta), *C. micranthum* (Cmmi), *O. europaea* (Oeu), *A. paniculata* (Apa), *S. cusia* (Scu), and *R. glutinosa* (Rgl) within the same gene family are also shown.

To further examine sequence modifications in the genome, the ratio of genes retained from each progenitor across the chromosomes of *A. tetraploideus* was analyzed (Fig. 4A). A high gene retention ratio across broad chromosomal regions indicated that SG1 and SG2 chromosomes preserved nearly complete sets of genes from their respective progenitor chromosomes, with minimal sequence exchange after hybridization. Notably, evidence of sequence exchange was more pronounced in SG2 than in SG1. For example, the result for chromosome 24B of *A. tetraploideus* showed an insertion of the Ae_A scaffold sequence within an Ae_B-derived chromosome (Fig. 4A). Moreover, there was a region on chromosome 20B of *A. tetraploideus* that lacked homologous gene matches from either the Ae_A scaffold or other scaffolds. Using genome sequences, annotation files, and the Liftoff program [58], gene annotations from chromosome 20B were mapped to the corresponding Ae_A scaffold. The results also showed that the mapped regions on the Ae_A scaffold contained repeat elements. As a result, these sequences, which masked repeat sequences, were not annotated by the annotation pipeline. Sequence translation revealed that many of the mapped annotations contained premature stop codons within their coding regions. These results were consistent with previous studies showing higher nucleotide diversity and novel expression bias in *A. ebracteatus* and SG2 compared with *A. ilicifolius* and SG1 [1, 4].

Chromosome 20B was longer than its homeologous chromosomes 15A, 20A, and 15B, as well as the *A. ebracteatus* scaffold Ae_A (Fig. 4A). Gene retention analysis revealed that the extended sequence of chromosome 20B comprised 176 genes that did not form any collinearity blocks with the progenitor genome sequences (Supplementary Table S18). In contrast, multiple internal collinearity blocks were detected within this region (Supplementary Fig. S5), indicating that it originated through segmental duplication. Orthologous analysis further assigned 105 of these genes to three gene families. BLAST research revealed that these genes matched uncharacterized or hypothetical proteins in the NCBI database, although they might play a role in the genome evolution of *A. tetraploideus*. The gene tree of the largest family showed 33 paralogous gene copies in *A. tetraploideus* that were orthologous to a single gene in *A. ebracteatus* (Fig. 4B). Paralogous relationships within this family were also found in other species, including *L. racemosa* (16 copies), *C. micranthum* (13 copies), and *A. paniculata* (10 copies), suggesting recurrent duplication of this gene group in mangrove and medicinal plants. Overall, this segmental duplication pattern likely contributed to the sequence extension observed in *A. tetraploideus*, as reported in other plants [59].

### Comparative genomics

**Figure 5:**
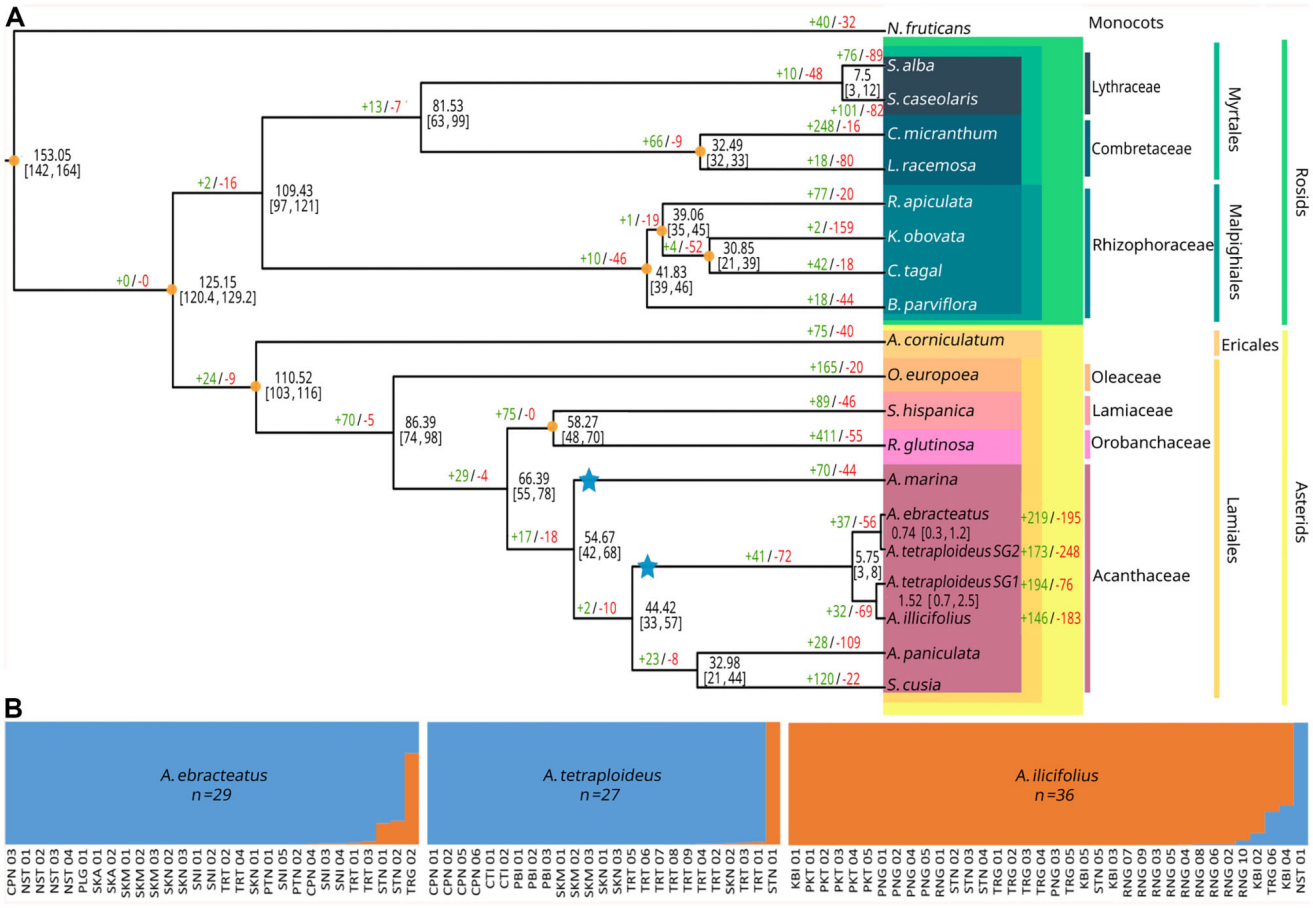
Divergence time and population structure of *Acanthus* species. (A) Maximum likelihood phylogenetic tree showing divergence times and gene family evolution among 19 plant species, including mangrove and nonmangrove taxa. Numbers at each node indicate divergence time estimates (in million years ago) with 95% confidence intervals in brackets. Green and red numbers represent significantly expanded (+) and contracted (−) gene families, respectively. Orange circles indicate calibration points derived from the TimeTree database, and blue stars mark lineage-specific WGD events in *Avicennia* and *Acanthus*. Subgenomes (SG1 and SG2) of *A. tetraploideus* are shown separately. Distinct background shades denote the clade, order, and family of each analyzed species. (B) Population structure analysis of *A. ebracteatus* (*n* = 29), *A. tetraploideus* (*n* = 27), and *A. ilicifolius* (*n* = 36) based on SNP variation. Each vertical bar represents an individual, with blue and orange segments indicating ancestry associated with Gulf of Thailand and Andaman coast populations, respectively. Gulf of Thailand populations included samples from Chumphon (CPN), Nakhon Si Thammarat (NST), Phatthalung (PLG), Phetchaburi (PBI), Samut Songkhram (SKM), Samut Sakhon (SKN), Surat Thani (SNI), and Trat (TRT), whereas Andaman coast populations included samples from Krabi (KBI), Phang Nga (PNG), Phuket (PKT), Ranong (RNG), Satun (STN), and Trang (TRG).

A phylogenetic tree of *A. ilicifolius, A. ebracteatus*, and the two subgenomes of *A. tetraploideus*, along with ten other mangrove species and six nonmangrove species, was constructed based on the sequences of 70 single-copy orthologous genes (Fig. 5A). Data of comparable scale have been sufficient to resolve species relationships in previous studies [60, 61]. The resulting topology was highly consistent with previously reported plastid- and genome-based phylogenies of mangroves and Acanthaceae [62–64]. The tree clearly separated the Rosids and Asterids clades, with the mangrove palm *Nypa fruticans* (monocot) serving as the outgroup. In this phylogeny, mangrove species were predominantly placed within the Rosids, whereas only five mangrove plants, including *A. corniculatum, A. marina, A. ilicifolius, A. ebracteatus*, and *A. tetraploideus*, were located within the Asterids. *A. corniculatum* represented the earliest diverging lineage among them and the only member of the Ericales in this clade. The remaining species belonged to the order Lamiales, whose common ancestor diverged from *A. corniculatum* approximately 103–116 Mya. Most species in this group, including *A. paniculata* and *S. cusia* (both in the family Acanthaceae), are terrestrial medicinal plants. Within Acanthaceae, the *Acanthus* lineage diverged from its terrestrial relatives approximately 33–57 Mya (Fig. 5A). The speciation between *A. ilicifolius* and *A. ebracteatus* was estimated to have occurred approximately 3–8 Mya. Using the relationship between Ks values and divergence time (see Materials and Methods), the WGD event was dated by calibrating the Ks peak corresponding to the duplication event (*Ks_WGD_*​ = 0.35) against the Ks value for progenitor divergence (*Ks_speciation_* ​= 0.05) and its associated divergence time (*T_speciation_* = 3–8 Mya). The WGD event was estimated to have occurred 21–56 Mya, during the period when the Acanthus lineage diverged from its terrestrial relatives. Based on the estimated progenitor divergence time of 5.75, the substitution rate within the*Acanthus* lineage was 4.3 × 10^−9^ substitutions per site per year. According to this rate, the major duplication event associated with the oldest peak within the *Acanthus* lineage (Ks ≈ 1.2–1.4) was estimated to have occurred approximately 138–161 Mya.

A new tetraploid species, recently named *A. tetraploideus* [1], was later present within the *Acanthus* lineage through the hybridization of *A. ilicifolius* and *A. ebracteatus*. To estimate the genome merger time, the divergence time between each subgenome and its corresponding progenitor was estimated. The results, however, yielded two distinct divergence times. The SG1 subgenome was estimated to have diverged from *A. ilicifolius* approximately 0.7–2.5 Mya, whereas SG2 diverged from *A. ebracteatus* about 0.3–1.2 Mya. The divergence pattern between the subgenomes of *A. tetraploideus* and its diploid progenitors appeared to be influenced by geographic history and gene flow. The primary habitats of *A. ilicifolius* on the Andaman coast and *A. ebracteatus* and *A. tetraploideus* in the Gulf of Thailand suggested that geographic isolation contributed to their evolutionary divergence. The sequenced *A. ilicifolius* individual (collected from the Andaman coast) may have diverged substantially from the ancestral *A. ilicifolius* lineage that contributed as SG1 in *A. tetraploideus*. In contrast, *A. ebracteatus* and *A. tetraploideus* still co-occurred in the Gulf of Thailand and were subjected to similar environmental pressures. In addition, spatial overlap between *A. ebracteatus* and *A. tetraploideus* could have facilitated postformation gene flow or backcrossing, potentially contributing to the lower divergence observed between SG2 and its diploid progenitor.

To support the geographic component of this hypothesis, SNP data from 90 accessions collected across 14 sampling sites were analyzed using population structure analysis. Consistent with their geographic distribution, the analysis identified 2 distinct genetic clusters, separating populations from the Gulf of Thailand and the Andaman coast in all *Acanthus* species (Fig. 5B). In *A. ilicifolius*, the single sample from the Gulf side was assigned to the blue cluster (the Gulf of Thailand cluster), while all individuals from Andaman provinces were consistently assigned to the orange cluster (the Andaman coast cluster) with little admixture. Similarly, in *A. ebracteatus*, individuals from Gulf provinces were uniformly assigned to the blue cluster, while only a few samples from the Andaman side exhibited partial Andaman ancestry. Nearly all *A. tetraploideus* samples were assigned to the Andaman cluster, with only one sample from Satun (STN-At-01) fully assigned to the Gulf of Thailand cluster. These results indicated a strong east–west genetic separation.

The timing of the hybridization event leading to allotetraploid *A. tetraploideus* was estimated from TE divergence profiles, following the framework described in [47, 48]. Divergence profiles of TE sequences from SG1 and SG2 were compared, with the values corresponding to progenitor divergence and a subsequent genome merger identified at 27.3% and 6.3%, respectively (Supplementary Fig. S1). Applying the same calibration framework used in Ks-based dating (see Materials and Methods), these divergence values were scaled against the estimated progenitor divergence time of 3–8 Mya, yielding an estimated genome merger (hybridization) time of 0.7–1.8 Mya.

### Protein family analysis

To enhance our understanding of the evolution of *Acanthus* species, protein family expansion and contraction were analyzed. Protein families were defined as expanded when they contained more protein members in a given species (leaf node) or ancestor (internal node) than in their most recent common ancestor (MRCA) and contracted when the reverse pattern was observed. Using this framework, numerous cases of gene family expansion and contraction across *A. marina, A. paniculata, S. cusia, A. ebracteatus, A. ilicifolius*, and the two subgenomes of *A. tetraploideus* (SG1 and SG2) were identified (Supplementary Table S19).

Several families were expanded in the MRCA of *Acanthus* species and contracted in the MRCA of *A. paniculata* and *S. cusia*. Many of these families were associated with root development. For example, families OG0001555 and OG0000052 were exclusively expanded within *Acanthus*. OG0001555 contained NFD6/NOXY2-like proteins, which were associated with lateral root development, while OG0000052 included MYB36 transcription factors, essential for Casparian strip formation. MYB36 was linked (via STRING co-occurrence) to protein MIZU-KUSSEI 1 (Ai1gPKTg3706.t1) in OG0006410, which was associated with the hydrotropism Gene Ontology (GO) term (GO:001027), suggesting a potential functional network involved in water-responsive root development. An additional family that could participate in Casparian strip formation was family OG0000343, which contained dirigent proteins. The protein counts in this family were higher in *Acanthus* species and Am than in Ap and Sc. In contrast, another root-related family (OG0001760), containing DEEPER ROOTING 1, showed reduced representation in all *Acanthus* species compared to other members of the Acanthaceae clade. The contraction of this family might reflect an evolutionary shift in *Acanthus* toward shallower root architectures, potentially as an adaptation to anoxic, waterlogged soils and surface-level substrate anchorage typical of intertidal environments.

Some families were expanded in the MRCA of *A. paniculata* and *S. cusia* but contracted in the broader Acanthaceae MRCAs. Many of these were involved in terpenoid biosynthesis. Their expansion seemed mainly driven by increased protein numbers in *S. cusia*, while other species maintained relatively stable counts. Some families also showed high variability across species. For instance, OG0000029 (germacrene synthases) exhibited 2- to 3-fold higher copy numbers in *A. paniculata* and *S. cusia* than in *A. marina, A. ilicifolius, A. ebracteatus*, SG1, and SG2 (Table 1). Family OG0000080 (beta-amyrin monooxygenases) displayed a gradual contraction across the phylogeny, with the fewest copies observed in both SG1 and SG2 of *A. tetraploideus*. These shifts implied that *Acanthus* species may have reduced their reliance on specific triterpenoid pathways relative to other Acanthaceae.

**Table 1:** Comparative analysis of gene family expansion and contraction in *Acanthus* and related Acanthaceae species.

Functional group	Family ID	Representative protein	Pattern in *Acanthus*	Pattern in other species
Root Development and Casparian Strip	OG0001555	NFD6/NOXY2-like	Expanded in *Acanthus* MRCA	Contracted in Ap/Sc
	OG0000052	MYB36	Expanded in *Acanthus* MRCA	Contracted in Ap/Sc
	OG0000343	Dirigent protein	Higher in *Acanthus* and Am	Lower in Ap/Sc
	OG0002958	Peroxidase 64	Expanded in SG1	Contracted in Ae/SG2
	OG0001760	Deeper rooting 1	Lower in *Acanthus*	Higher in other Acanthaceae
	OG0009241	Homeobox-leucine zipper ANL2	Higher in *Acanthus*	Lower in others
	OG0017721	Homeobox-leucine zipper ANL2	Higher in *Acanthus*	Lower in others
	OG0000866	Rho GDP-dissociation inhibitor 1	Highest in *Acanthus*	Present in all
Osmotic Stress and Water Transport	OG0000188	WIP-type zinc finger	Expanded in Ae/SG2/Am	Lower in Ai/SG1/Ap/Sc
	OG0013347	Aquaporin PIP2-7	Expanded in Ae/SG2	Not expanded in others
	OG0000840	Mechanosensitive ion channel	Contracted in Ae	Present in others
	OG0004372	A20/AN1 stress protein	Higher in *Acanthus*	Lower in others
	OG0012170	A20/AN1 stress protein	Higher in *Acanthus*	Lower in others
Cell Wall Remodeling	OG0000012	Xyloglucan endotransglucosylase	Expanded in Ai/SG1	Contracted in Ae/SG2
	OG0000227	Pectinesterase inhibitor	Expanded in Ai/SG1	Contracted in Ae/SG2
Secondary Metabolism (Flavonoids and Terpenoids)	OG0010398	Chalcone synthase	Expanded in Ai	Contracted in Ae
	OG0000848	Chalcone synthase	Expanded in Ai	Contracted in Ae
	OG0000029	Germacrene synthase	Fluctuated	Higher in Ap/Sc
	OG0000080	Beta-amyrin monooxygenase	Contracted in *Acanthus*	Higher in Am
	OG0006911	Ferruginol synthase	Unique to *Acanthus*	Absent in others
	OG0014066	Ferruginol synthase	Unique to *Acanthus*	Absent in others
Stress/Signaling Regulators	OG0003440	PHD finger Alfin1	Higher in *Acanthus*	Lower in others
Unknown/Hypothetical	OG0000061	Hypothetical protein	Very high in *Acanthus*	Low in others

Several protein families exhibited opposing patterns of expansion and contraction between the Ai–SG1 and Ae–SG2 lineages. For instance, families OG0000012 and OG0000227, encoding probable xyloglucan endotransglucosylase/hydrolase (XTH) and putative pectinesterase/pectinesterase inhibitors (PMEI), were expanded in the MRCA of *A. ilicifolius* and SG1 but contracted in *A. ebracteatus* and SG2. A similar trend was observed for families OG0010398 and OG0000848, both containing chalcone synthase in the flavonoid biosynthesis pathway, which were expanded in *A. ilicifolius* but contracted in *A. ebracteatus*. Likewise, the family of endodermis-specific peroxidase 64 (OG0002958) was expanded in SG1 but contracted in Ae and SG2. Family OG0000840, encoding mechanosensitive ion channel proteins, was also contracted in *A. ebracteatus*. Conversely, family OG0013347, comprising aquaporin PIP2-7 members, underwent significant expansion in the MRCA of *A. ebracteatus* and SG2, with the highest number of copies observed in *A. ebracteatus*. Family OG0000188 of WIP-type zinc finger proteins was expanded in Ae, SG2, and Am but had lower copy numbers in Ap, Sc, Ai, and SG1. These contrasting patterns highlighted divergent evolutionary pressures acting on the two parental lineages and their respective contributions to the *A. tetraploideus* genome.

To complement the phylogenetic expansion and contraction analysis, we applied machine learning to identify protein families with markedly different member counts in *Acanthus* compared to other taxa. Several families exhibited *Acanthus*-specific abundance patterns. Examples were OG0003440 (PHD finger protein Alfin1), OG0004372 and OG0012170 (A20/AN1 stress-associated proteins), and OG0009241 and OG0017721 (homeobox-leucine zipper protein ANTHOCYANINLESS 2), associating with salt, osmotic, or water deprivation responses. They appeared specific to the Acanthaceae clade (except OG0003440) and were more abundant in *Acanthus* species than in all others studied. Family OG0000866 (Rho GDP-dissociation inhibitor 1), involved in root epidermal cell differentiation, was found in all species but was most abundant in *Acanthus*. Finally, families OG0006911 and OG0014066, containing ferruginol synthases, were uniquely present in *Acanthus*. Ferruginol is a diterpene phenol with antibacterial, antitumor, and antimalarial activities [65].

## Discussion

Our genome assemblies confirm that *A. tetraploideus* is an allotetraploid derived from *A. ilicifolius* (SG1) and *A. ebracteatus* (SG2). The strong synteny between subgenomes and their progenitors, together with the high proportion of duplicated genes, underscores a relatively recent hybridization and whole-genome duplication event. Based on our findings, we propose that the evolutionary history of *Acanthus* was collectively shaped by genome duplication, ancient divergence, subsequent lineage-specific evolution, and recent hybridization.

The evolutionary trajectory of the genus began with the divergence of the *Acanthus* lineage from the ancestor of the terrestrial species *A. paniculata* and *S. cusia* approximately 33–57 Mya and was subsequently shaped by a WGD event dated to about 21–56 Mya. Both events coincided with early–middle Eocene (∼50–40 Mya) climatic upheavals, which created unstable coastal habitats [66]. These stresses likely promoted ecological separation from terrestrial relatives, while WGD provided genetic redundancy and flexibility to adapt to salinity fluctuations, tidal inundation, and anoxic soils [67, 68], similar to patterns reported in Malpighiales [67]. Following this duplication, *Acanthus* underwent lineage-specific remodeling of root-associated gene families. The expansions of *MYB36, DIR10-like*, and *PER64* might reinforce Casparian strip barriers [69–71], while *NOXY2-like, ANL2, RhoGDI1, Alfin1*, and *SAP* could support root development and stress tolerance [72–76]. In contrast, contraction of *DRO1* suggests a shift toward shallow, laterally spreading root systems suited to sediment-rich mangrove shorelines [58]. Contractions in terpenoid biosynthesis gene families, compared with the terrestrial medicinal plants *A. paniculata* [77] and *S. cusia* [37], further indicate a shift in secondary metabolism associated with mangrove adaptation. Together, these changes highlight how WGD and subsequent gene family evolution enabled *Acanthus* to persist and specialize in dynamic coastal environments.

Later, during the Late Miocene (5–10 Mya), *A. ilicifolius* and *A. ebracteatus* diverged under intensified monsoons, fluctuating sea levels, and tidal reorganization that reshaped Southeast Asian coastlines [78–80], which likely fragmented mangrove populations, promoting dispersal and allopatric divergence. This scenario is supported by modeled tidal ranges during the Messinian (∼6 Mya) that align with the present-day distribution of *A. ilicifolius* along the open coasts of the Andaman Sea and *A. ebracteatus* in the more sheltered estuaries of the Gulf of Thailand [1, 78]. The distribution of these species is further supported by genetic separation of populations from the east and west coasts, which appears to be influenced by environmental conditions such as salinity and hydrology [81]. Genomic comparisons revealed greater structural and sequence variation in *A. ebracteatus* than in *A. ilicifolius* [1], indicating independent evolutionary trajectories. Gene family expansions were more pronounced in *A. ilicifolius* and SG1, particularly in root-related families such as *PER64* and *XTH23*, which reinforced Casparian strip function and supported lateral root adaptation to salinity [82, 83]. An expansion of *CHS*, the first enzyme in flavonoid biosynthesis and a contributor to stress tolerance [84], also corresponded with the high flavonoid and phenolic content of *A. ilicifolius* [3]. Because *CHS* and other flavonoid biosynthesis genes influence flower pigmentation [85], this expansion may also underlie differences in floral coloration—violet in *A. ilicifolius*, white in *A. ebracteatus*, and pale violet in *A. tetraploideus*. Together, these lineage-specific genomic patterns and the east–west coastal separation indicate that *A. ilicifolius* and *A. ebracteatus* were shaped by contrasting ecological pressures.

Subsequently, glacial–interglacial cycles and sea-level fluctuations during the early Pleistocene (∼1.3–1.5 Mya) repeatedly isolated and reconnected mangrove habitats [86–88], providing opportunities for secondary contact between *A. ilicifolius* and *A. ebracteatus* and ultimately leading to the formation of the allotetraploid *A. tetraploideus*. The contrasting genomic architectures of the two progenitor species may have played a role in the origin of the allotetraploid. The smaller, more structurally dynamic genome of *A. ebracteatus* likely facilitated hybrid compatibility with the larger genome of *A. ilicifolius* [89]. In *A. tetraploideus*, transcriptomic analyses revealed a consistent expression bias toward SG1, reflecting subgenome dominance, whereas SG2 contributed lineage-specific and novel expression patterns [4]. Such asymmetric behavior mirrors that observed in other allopolyploids [90, 91], where one subgenome maintains regulatory integrity while the other enhances adaptive potential. Together, these complementary roles may have underpinned both the successful formation and ecological expansion of *A. tetraploideus*, enabling it to thrive across diverse coastal habitats inherited from its progenitor lineages. Additionally, unlike sterile hybrids in other mangrove genera such as *Rhizophora* and *Avicennia* [92], *A. tetraploideus* is capable of producing viable seeds in addition to clonal propagation [1]. Subgenomes of *A. tetraploideus* remain largely intact and collinear with their progenitors, and limited homeologous recombination likely supports disomic pairing and balanced gamete formation [93]. Thus, the integration of adaptive genes from ecologically divergent parents, transcriptomic reprogramming, and the capacity for both sexual and asexual reproduction underpin the evolutionary success of *A. tetraploideus* in coastal ecosystems.

In summary, the interplay of genome duplication, ecological divergence, and hybridization highlights the evolutionary history of *Acanthus*. Divergence from terrestrial relatives with a WGD during the Eocene, the split between *A. ilicifolius* and *A. ebracteatus* in the Miocene, and the formation of the allotetraploid *A. tetraploideus* through secondary contact during the Pleistocene together define the key stages of *Acanthus* evolution. This sequence also illustrates how climatic change and genomic processes shaped mangrove diversification. Future studies on stress-related gene families and subgenome interactions will clarify how polyploidy and hybridization contribute to resilience in coastal ecosystems.

## Additional Files

**Supplementary Fig. S1**. Distribution of TE divergence rates in the subgenomes of *Acanthus tetraploideus*. Kernel density estimates of LTR retrotransposon divergence rates (Kimura distances) are shown for subgenome SG1 (yellow) and subgenome SG2 (blue). The two vertical red lines indicate the first and last intersections between the density curves of SG1 and SG2 (at Kimura values of 6.3 and 27.3, respectively). Similar to Ks values, these two divergence rates corresponded to the genome merger and divergence events between the two subgenomes.

**Supplementary Fig. S2**. High-resolution Hi-C contact maps for all chromosomes of *Acanthus tetraploideus*. High-resolution Hi-C contact maps (100-kb resolution) for each of the 48 nuclear chromosomes of *A. tetraploideus*. These panels are snapshot images exported from Juicebox, showing intrachromosomal interaction intensity across each chromosome. Green boxes indicate boundaries of PacBio-based assembled contigs. Red dots indicate strong Hi-C contact signals, corresponding to regions with dense chromatin interactions. Repeat-rich regions identified from the PacBio-based assembly show reduced Hi-C signal intensity, as expected due to low mappability of repetitive sequences. Chromosome numbers are shown below each contact map, with the values in parentheses corresponding to the chromosome identifiers displayed in Fig. 1C.

**Supplementary Fig. S3**. Self-synteny dot plot of the *Acanthus tetraploideus* genome. The plot illustrates intragenomic synteny among chromosomes of the *A. tetraploideus* assembly. Red dots represent homoeologous matches between subgenomes SG1 and SG2, while blue dots indicate collinear regions resulting from WGD events within each subgenome. The presence of 11 diagonal syntenic blocks with dense dot patterns highlights conserved segments corresponding to 11 ancestral protochromosomes.

**Supplementary Fig. S4**. Gene retention ratios along all chromosomes of *A. tetraploideus*. Ratios represent the proportion of genes retained from each progenitor, ranging from 0 to 1. Red and yellow lines indicate genes retained from the two groups of WGD-derived scaffolds in *A. ilicifolius*, whereas green and blue lines represent genes inherited from *A. ebracteatus*.

**Supplementary Fig. S5**. Segmental duplications within chromosome 20B of *Acanthus tetraploideus*. (A) Segmental duplications detected in plus orientation within the extended sequence of chromosome 20B. (B) Segmental duplications detected in minus orientation within the same region. Each colored dot represents a duplicated block, with coordinates indicating the duplicated segments along the extended region. Multiple duplication blocks are observed, suggesting extensive internal segmental duplication within chromosome 20B.

**Supplementary Table S1**. Proportional assignment to genetic clusters (*K* = 2) and estimated genome sizes of *A. tetraploideus* individuals.

**Supplementary Table S2**. QUAST report summary for the Hi-C genome assembly of *Acanthus tetraploideus*.

**Supplementary Table S3**. Summary of telomeric repeat distribution and orientation in *Acanthus tetraploideus* chromosomes based on QuarTeT analysis.

**Supplementary Table S4**. BUSCO assessment of genome completeness for *Acanthus tetraploideus* assembly.

**Supplementary Table S5**. QUAST report summary for the stLFR genome assembly of *Acanthus ilicifolius*.

**Supplementary Table S6**. QUAST report summary for the stLFR genome assembly of *Acanthus ebracteatus*.

**Supplementary Table S7**. Proportional assignment to genetic clusters (*K* = 2) and estimated genome sizes of *A. ilicifolius* individuals.

**Supplementary Table S8**. Proportional assignment to genetic clusters (*K* = 2) and estimated genome sizes of *A. ebracteatus* individuals.

**Supplementary Table S9**. BUSCO assessment of genome completeness for *Acanthus ilicifolius* assembly.

**Supplementary Table S10**. BUSCO assessment of genome completeness for *Acanthus ebracteatus* assembly.

**Supplementary Table S11**. Nucleotide alignment percentages between the genomes of *Acanthus ilicifolius* and *Acanthus ebracteatus* and the subgenomes of *Acanthus tetraploideus* across identity thresholds.

**Supplementary Table S12**. Genome annotation statistics of *Acanthus tetraploideus*.

**Supplementary Table S13**. Genome annotation statistics of *Acanthus ilicifolius*.

**Supplementary Table S14**. Genome annotation statistics of *Acanthus ebracteatus*.

**Supplementary Table S15**. BUSCO assessment of genome completeness for *Acanthus tetraploideus* annotated proteins.

**Supplementary Table S16**. BUSCO assessment of genome completeness for *Acanthus ilicifolius* annotated proteins.

**Supplementary Table S17**. BUSCO assessment of genome completeness for *Acanthus ebracteatus* annotated proteins.

**Supplementary Table S18**. Gene insertion patterns in *Acanthus tetraploideus* based on retention ratios from each progenitor genome and associated orthogroups.

**Supplementary Table S19**. Gene families with significant copy number variation across Acanthaceae species and associated functional annotations.

## Abbreviations

BUSCO: Benchmarking Universal Single-Copy Orthologs; GMMs: Gaussian Mixture Models; GO: Gene Ontology; Hi-C: High-throughput Chromosome Conformation Capture; Ks: synonymous substitution rate; LTR: long terminal repeat; MAF: minor allele frequency; MCMC: Markov Chain Monte Carlo; MRCA: most recent common ancestor; Mya: million years ago; N50: contig or scaffold length at 50% of genome assembly; OG: orthogroup; RNA-seq: RNA sequencing; SNP: single-nucleotide polymorphism; stLFR: single-tube long fragment read; TE: transposable element; WGD: whole-genome duplication; XTH: xyloglucan endotransglucosylase/hydrolase.

## Supplementary Material

giaf162_Supplemental_Files

giaf162_Authors_Response_To_Reviewer_Comments_original_submission

giaf162_Authors_Response_To_Reviewer_Comments_Revision_1

giaf162_GIGA-D-25-00249_Original_Submission

giaf162_GIGA-D-25-00249_Revision_1

giaf162_GIGA-D-25-00249_Revision_2

giaf162_Reviewer_1_Report_Original_Submission Minghui Kang -- 7/31/2025

giaf162_Reviewer_1_Report_Revision_1 Minghui Kang -- 11/14/2025

giaf162_Reviewer_2_Report_Original_Submission Zhe Liang -- 8/5/2025

giaf162_Reviewer_2_Report_Revision_1 Zhe Liang -- 11/21/2025

## Data Availability

The assembled genome sequences of *A. tetraploideus, A. ilicifolius*, and *A. ebracteatus* were deposited in the NCBI database under BioProject PRJNA1102049, PRJNA1275650, and PRJNA1111239, respectively. All additional supporting data are available in the *GigaScience* repository, GigaDB [94].
