# Supplementary material for: Genome Assembly of Three Shrub Mangroves in the Genus Acanthus Reveals Two Polyploidy Events and Expansion of Genes Linked to Root Adaptation in Coastal Habitats
Source: Gigascience. 2026 Jan 2;15:giaf162. doi: 10.1093/gigascience/giaf162 (PMC12903786; doi:10.1093/gigascience/giaf162)
Supplement: giaf162_Supplemental_Files [file giaf162_supplemental_files.zip › Supplementary_Figures_1-5.pdf]

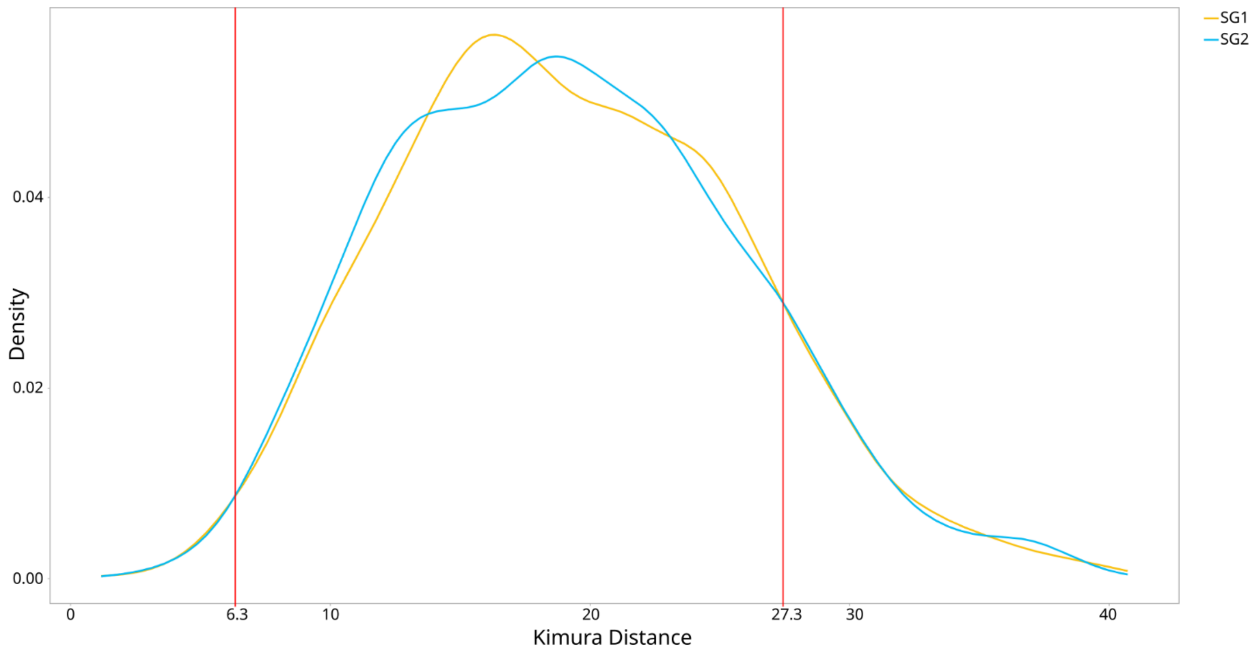

Supplementary Figure S1 Distribution of TE divergence rates in the subgenomes of *Acanthus tetraploideus*. Kernel density estimates of LTR retrotransposon divergence rates (Kimura distances) are shown for subgenome SG1 (yellow) and subgenome SG2 (blue). The Two vertical red lines indicate the first and last intersections between the density curves of SG1 and SG2 (at Kimura values 6.3 and 27.3, respectively). Similar to Ks values, these two divergence rates corresponded to the genome merger and divergence events between the two subgenomes.

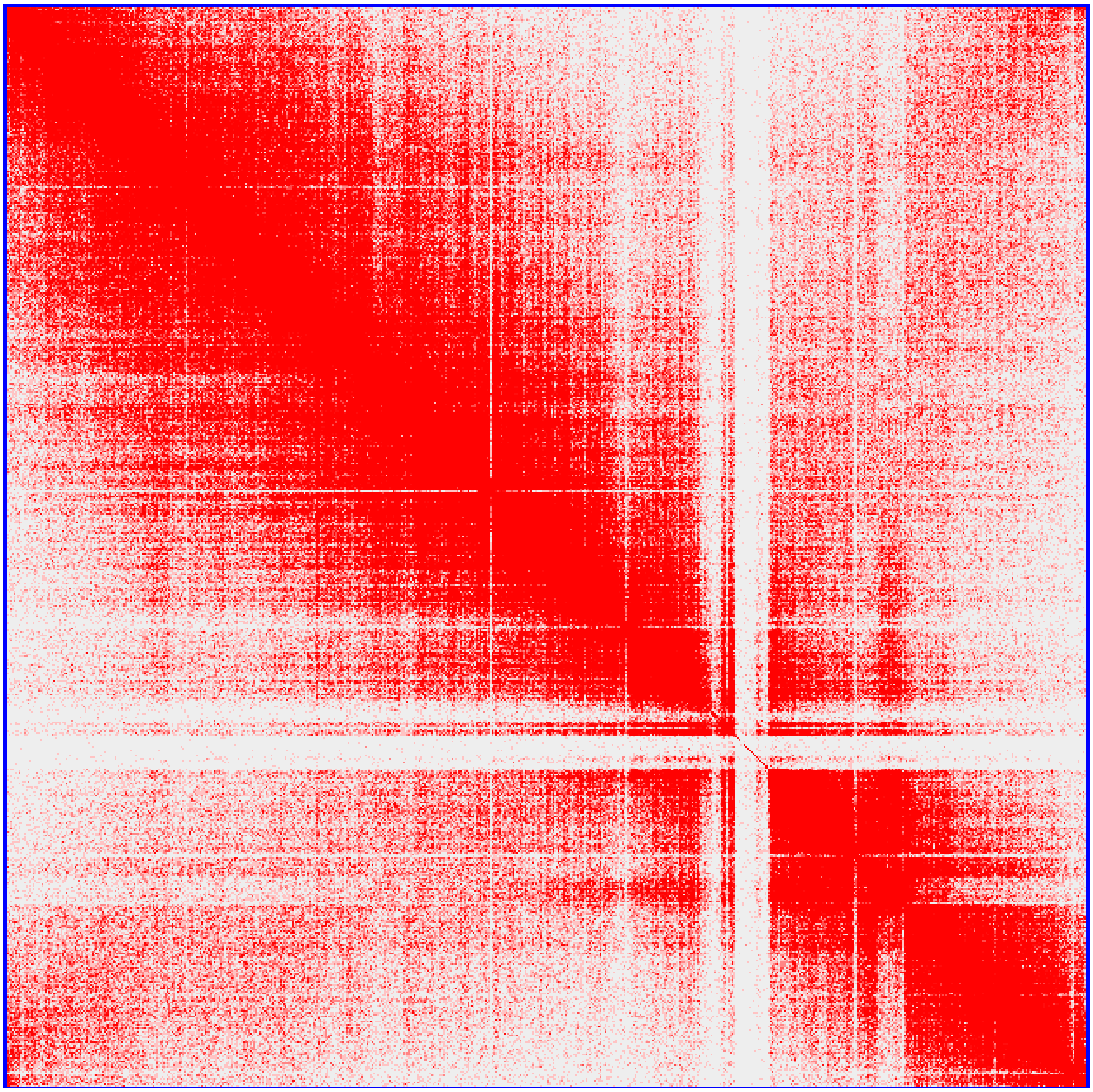

Chromosome 1 (1A)

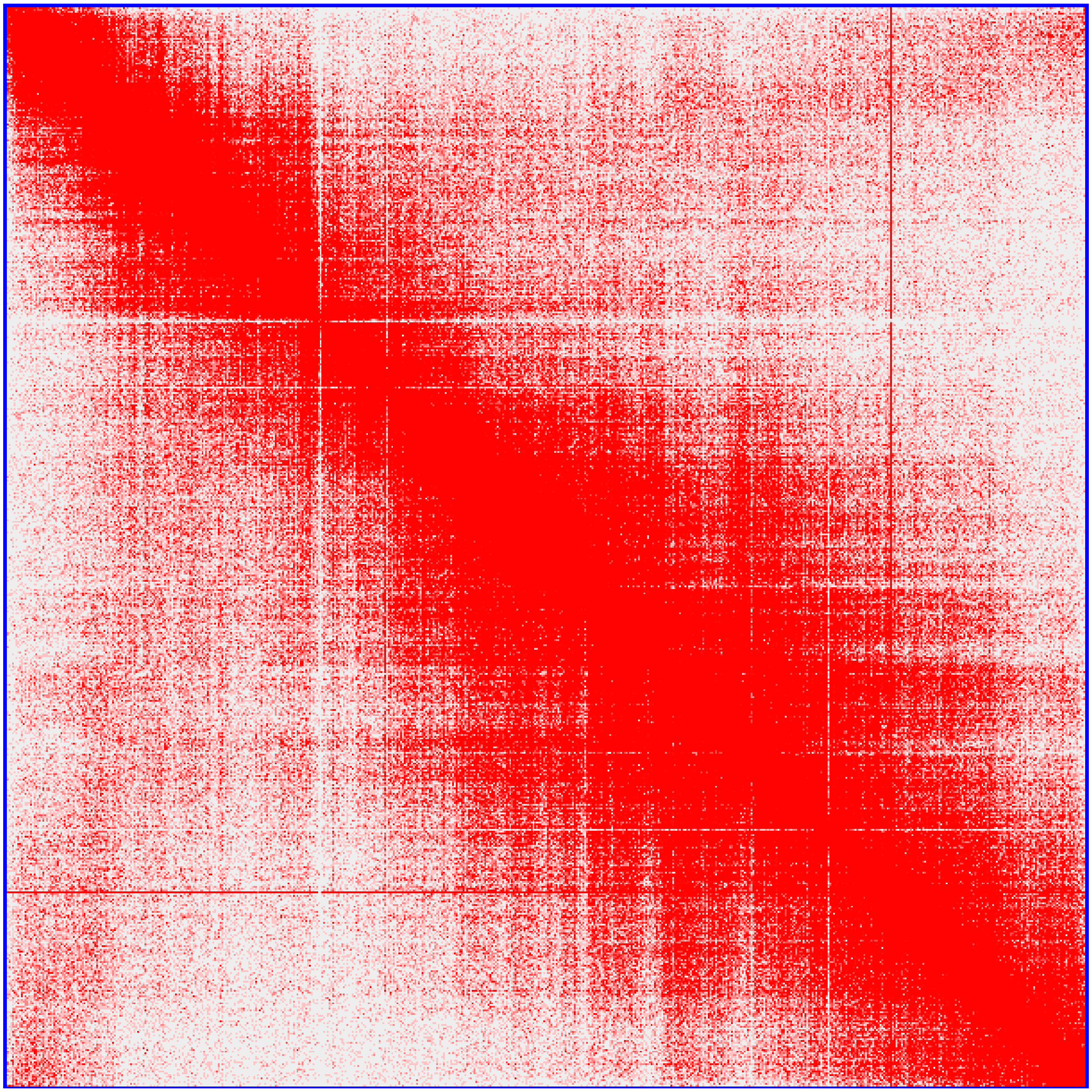

Chromosome 2 (2A)

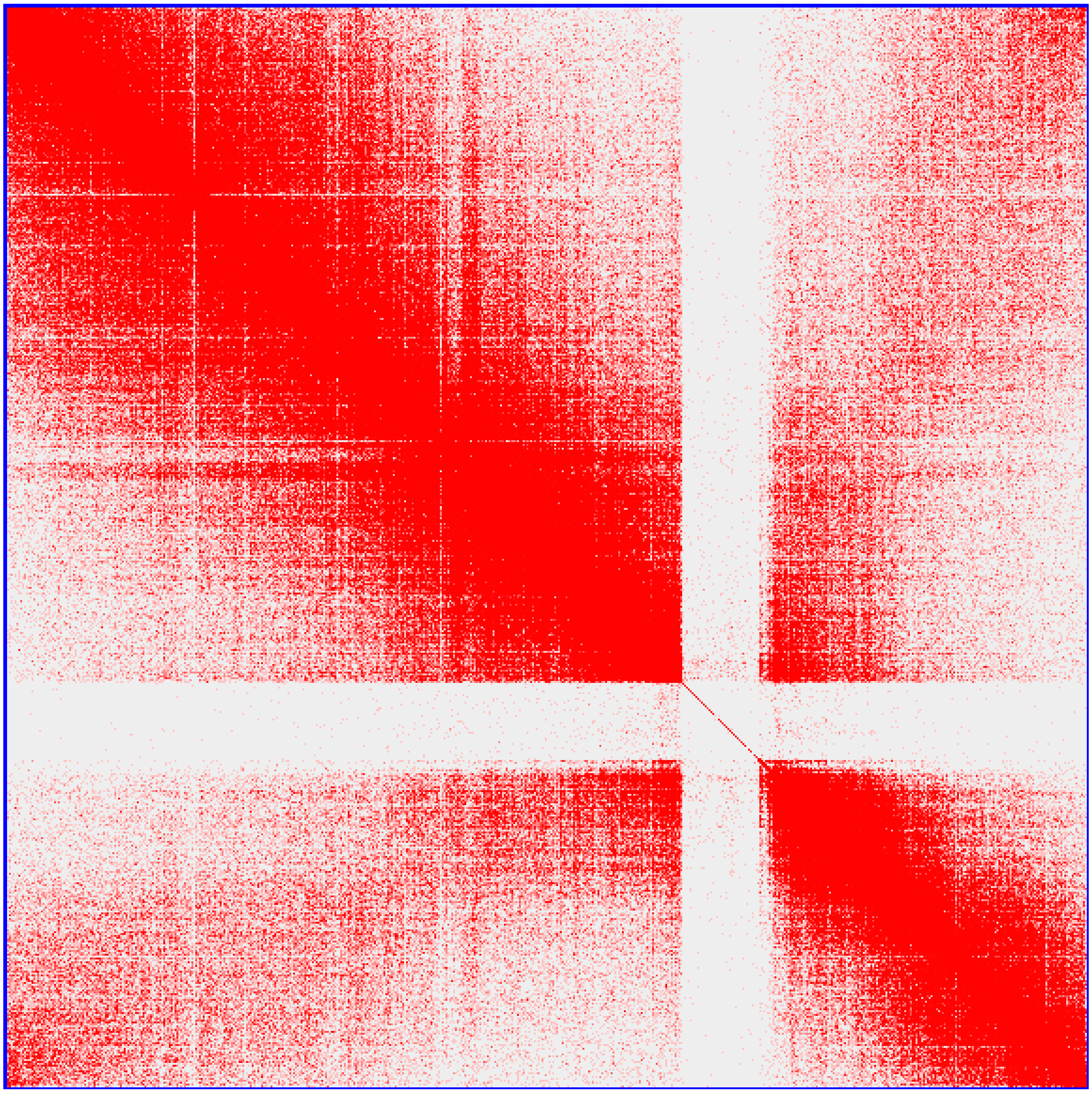

Chromosome 3 (1B)

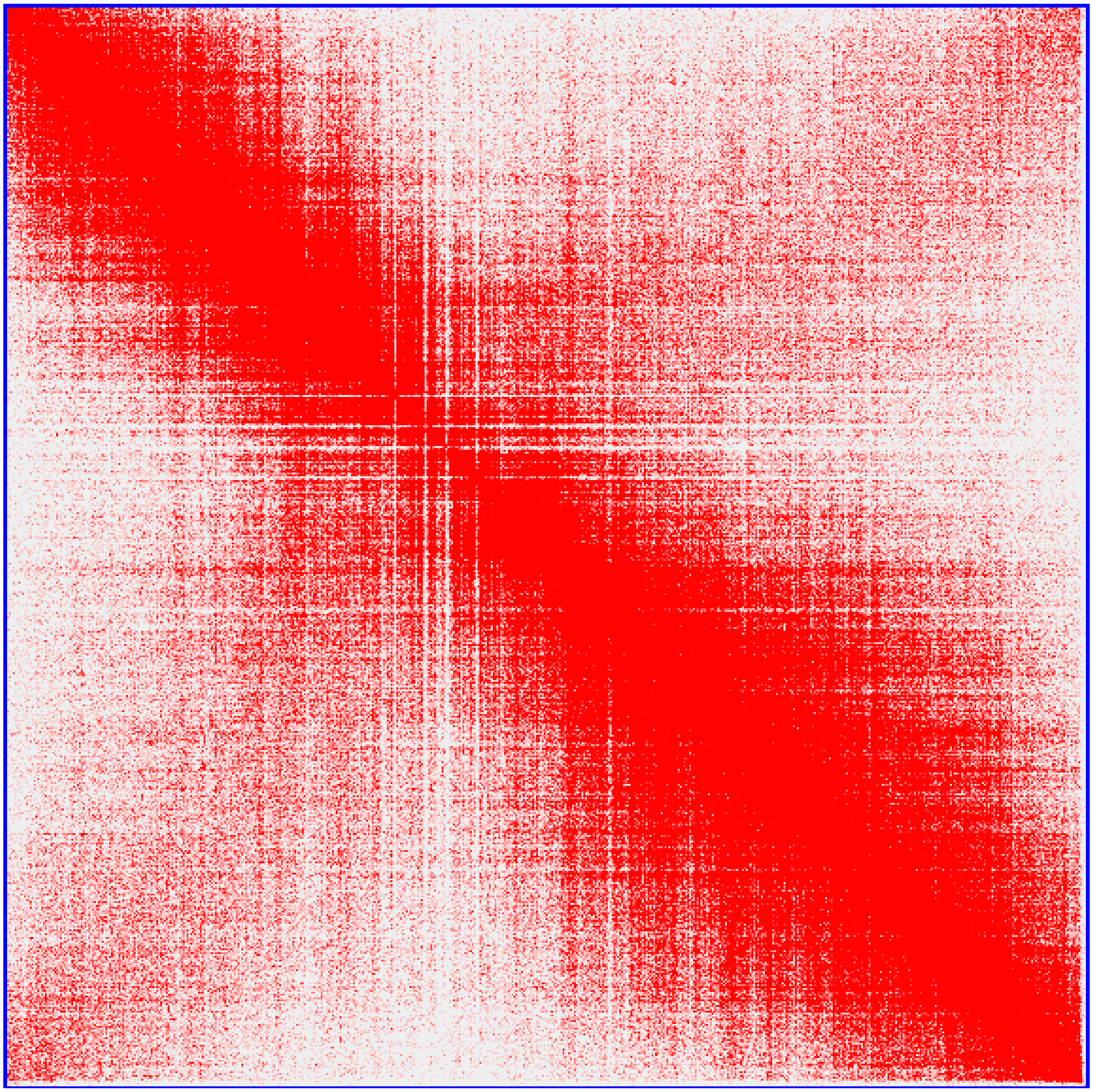

Chromosome 4 (3A)

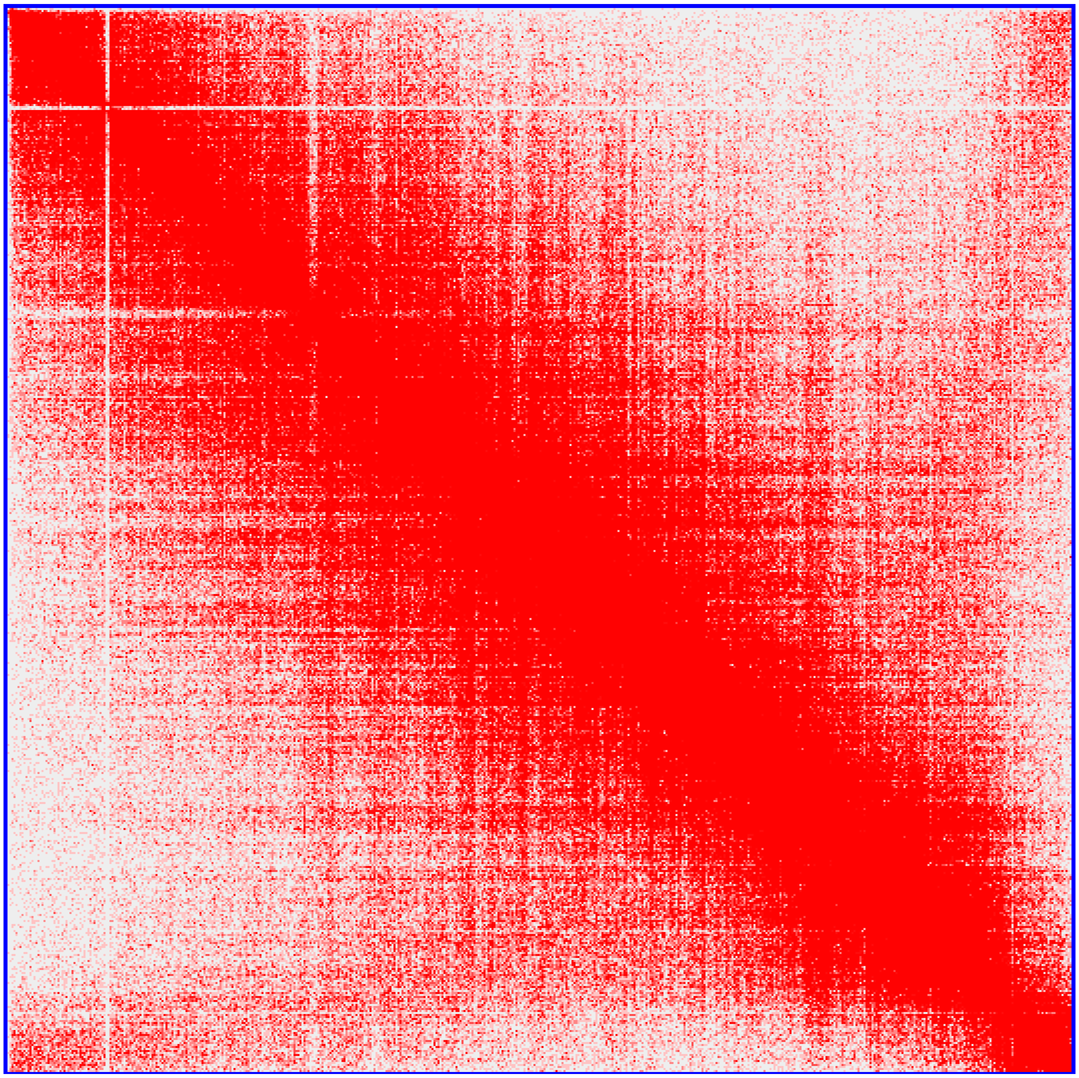

Chromosome 5 (4A)

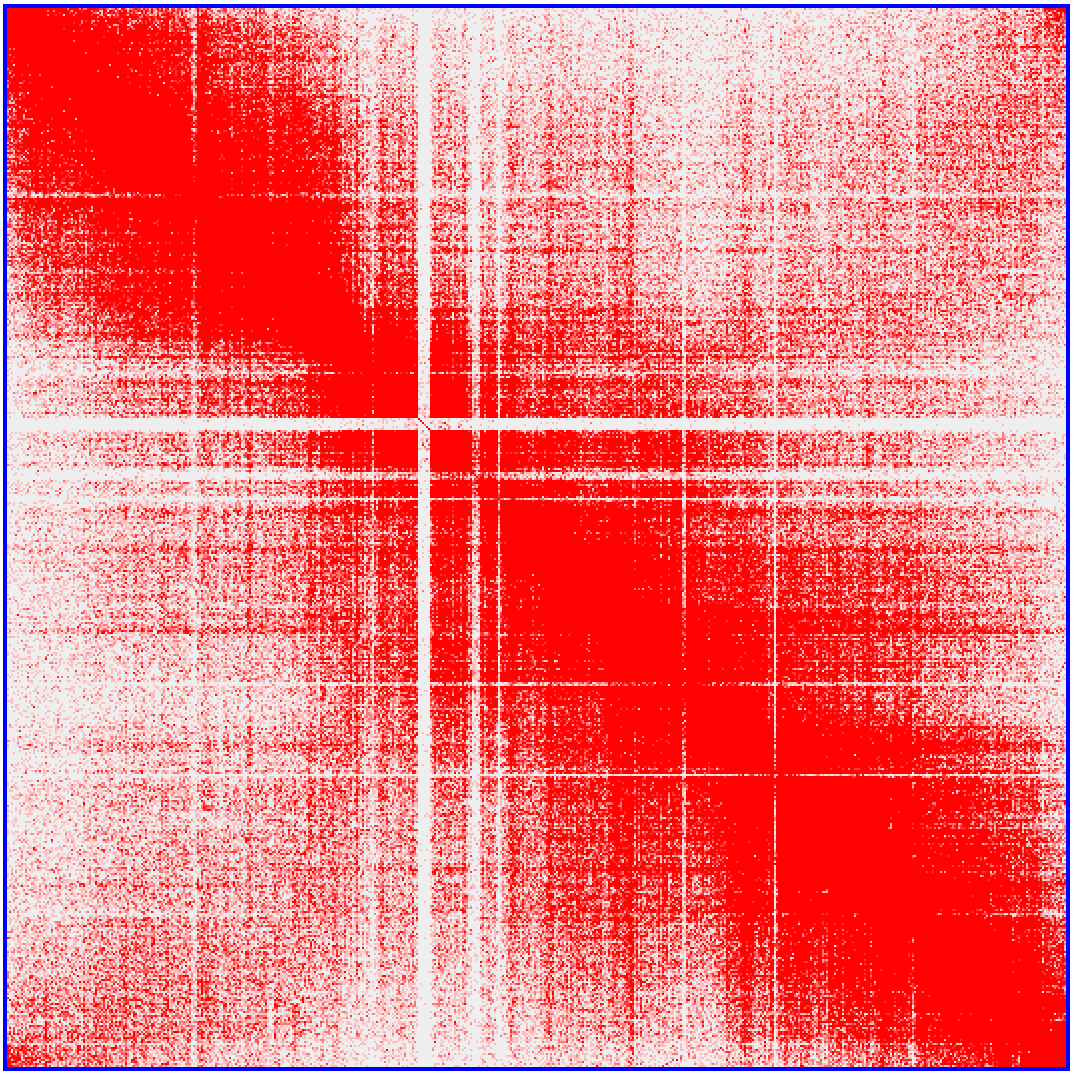

Chromosome 6 (5A)

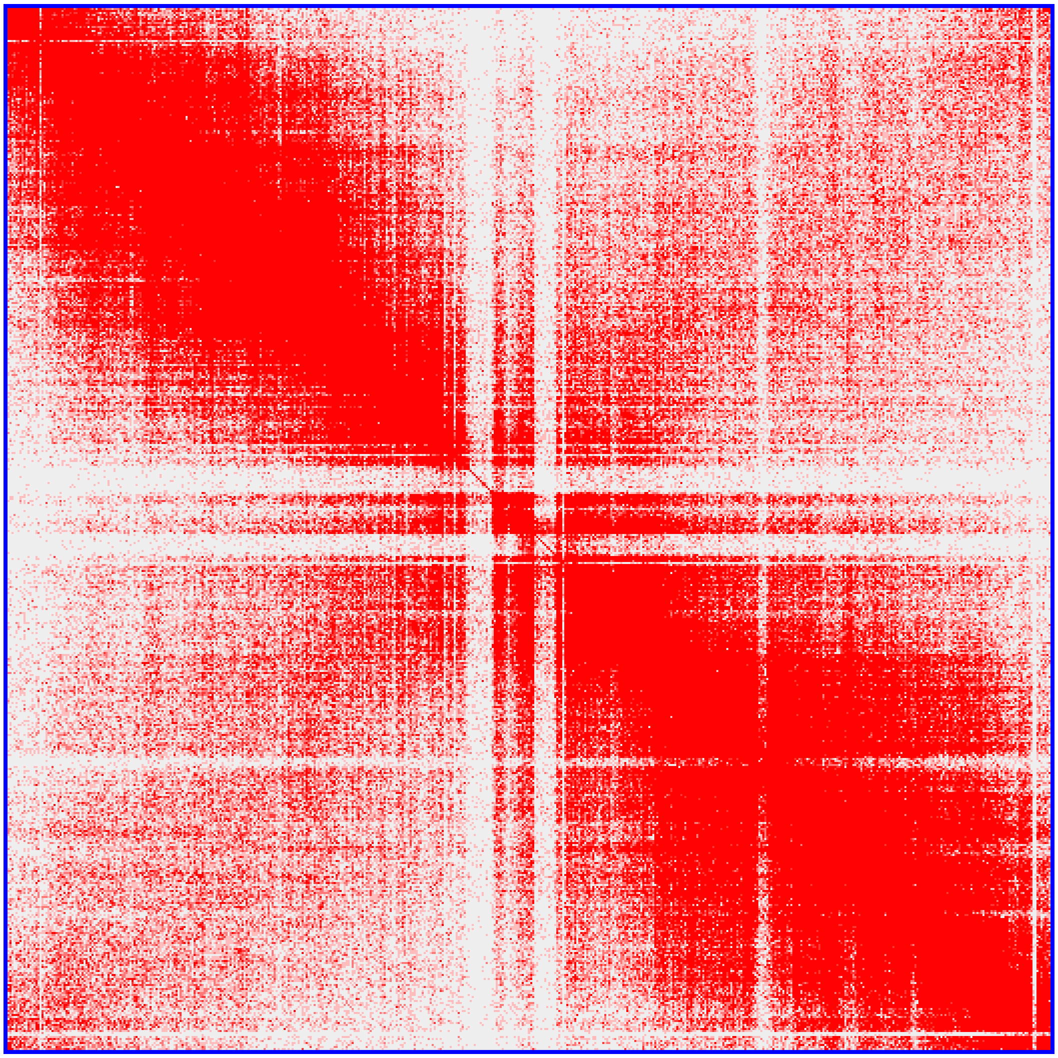

Chromosome 7 (6A)

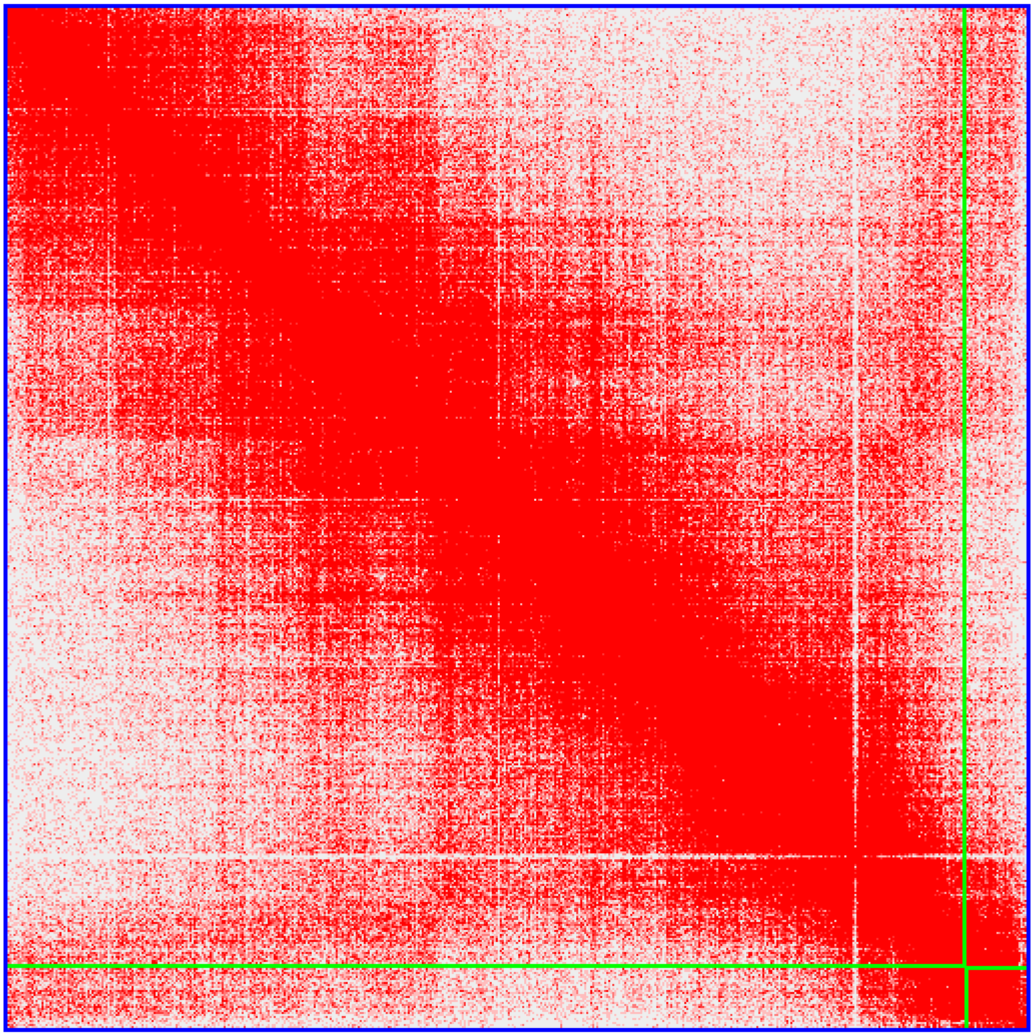

Chromosome 8 (2B)

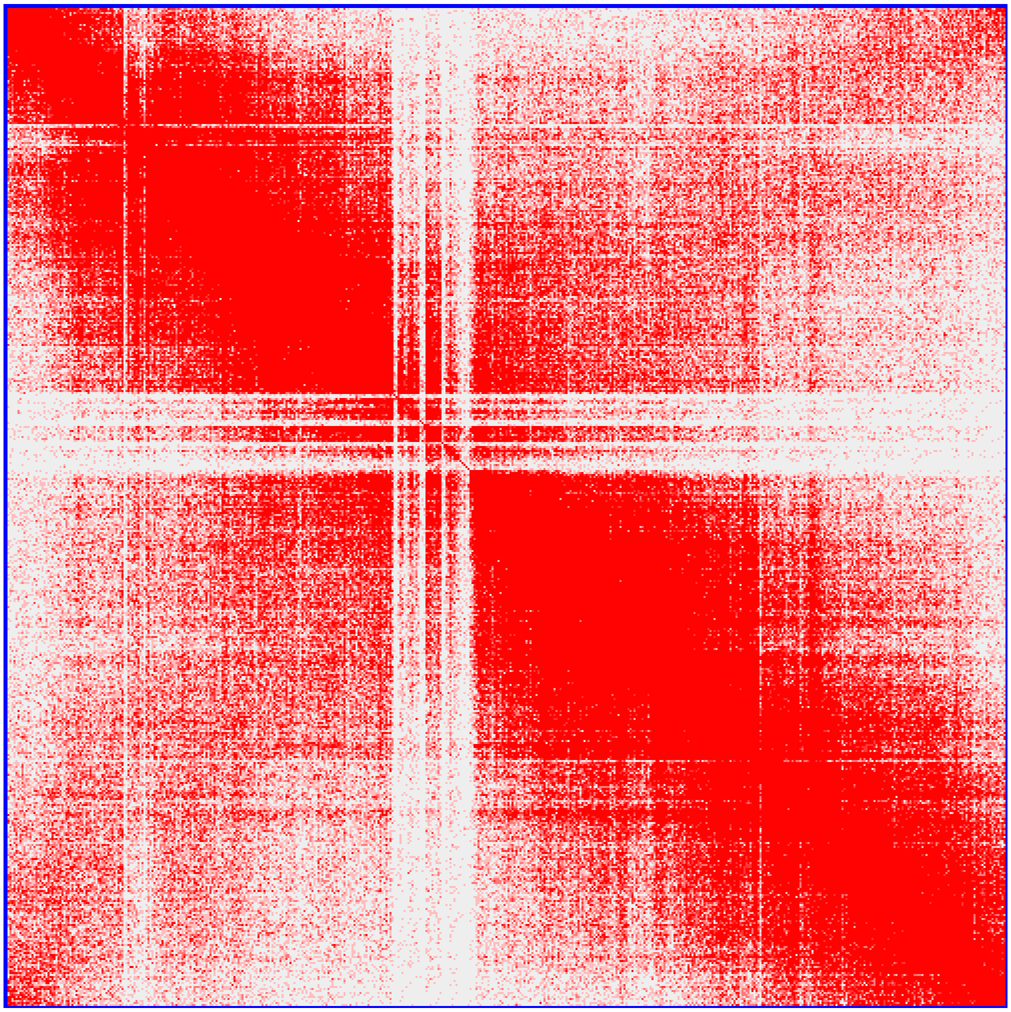

Chromosome 9 (7A)

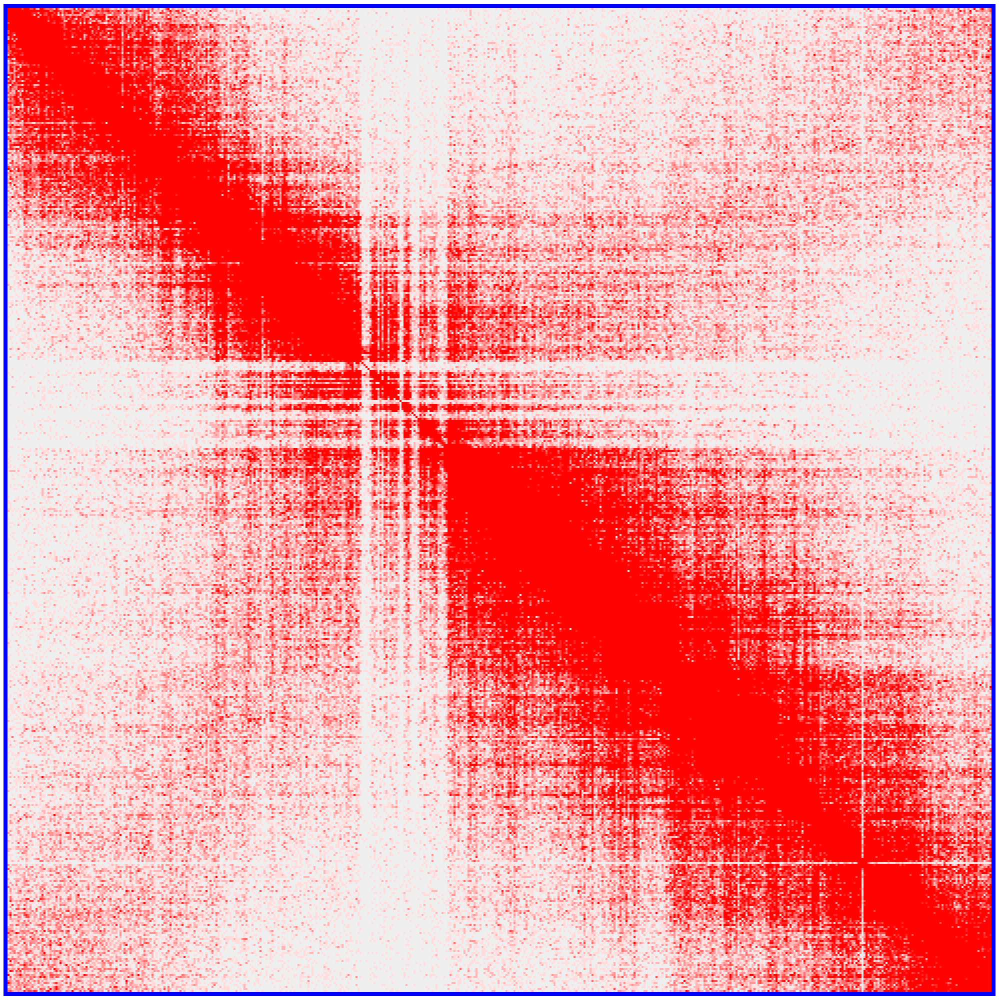

Chromosome 10 (3B)

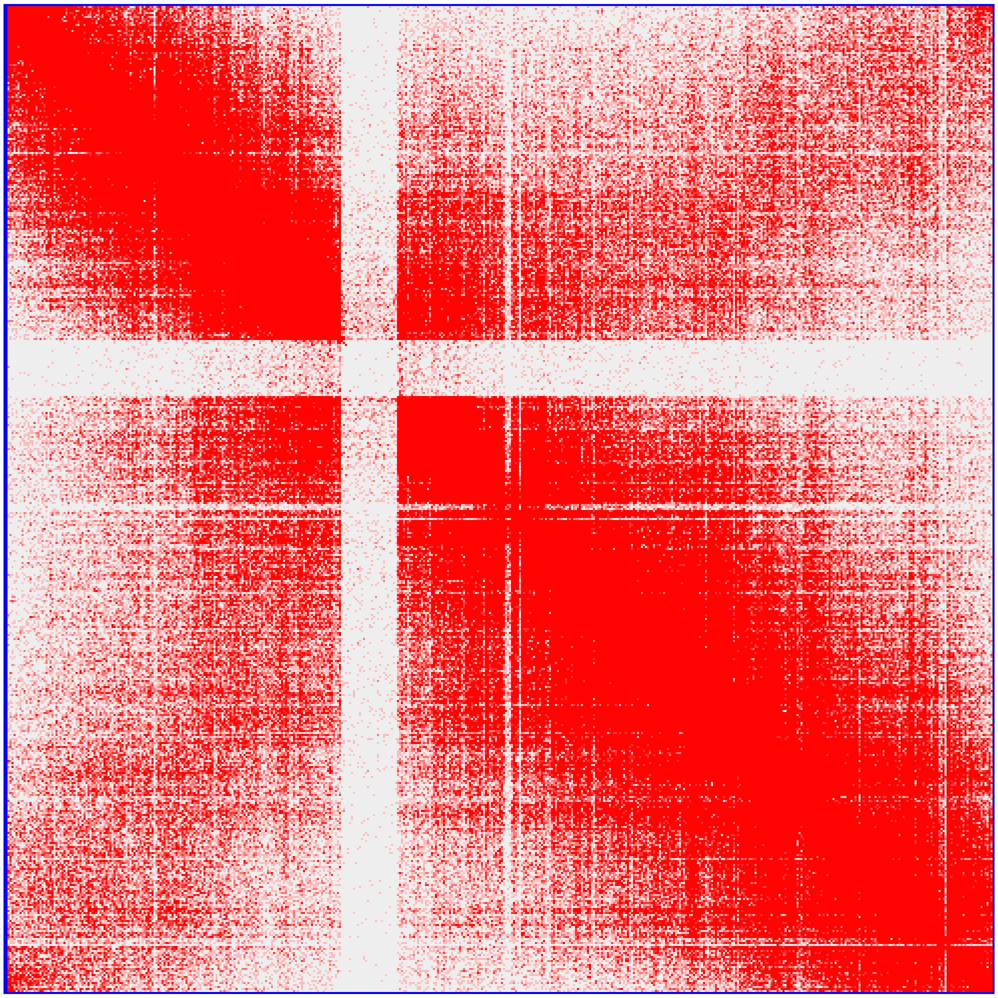

Chromosome 11 (8A)

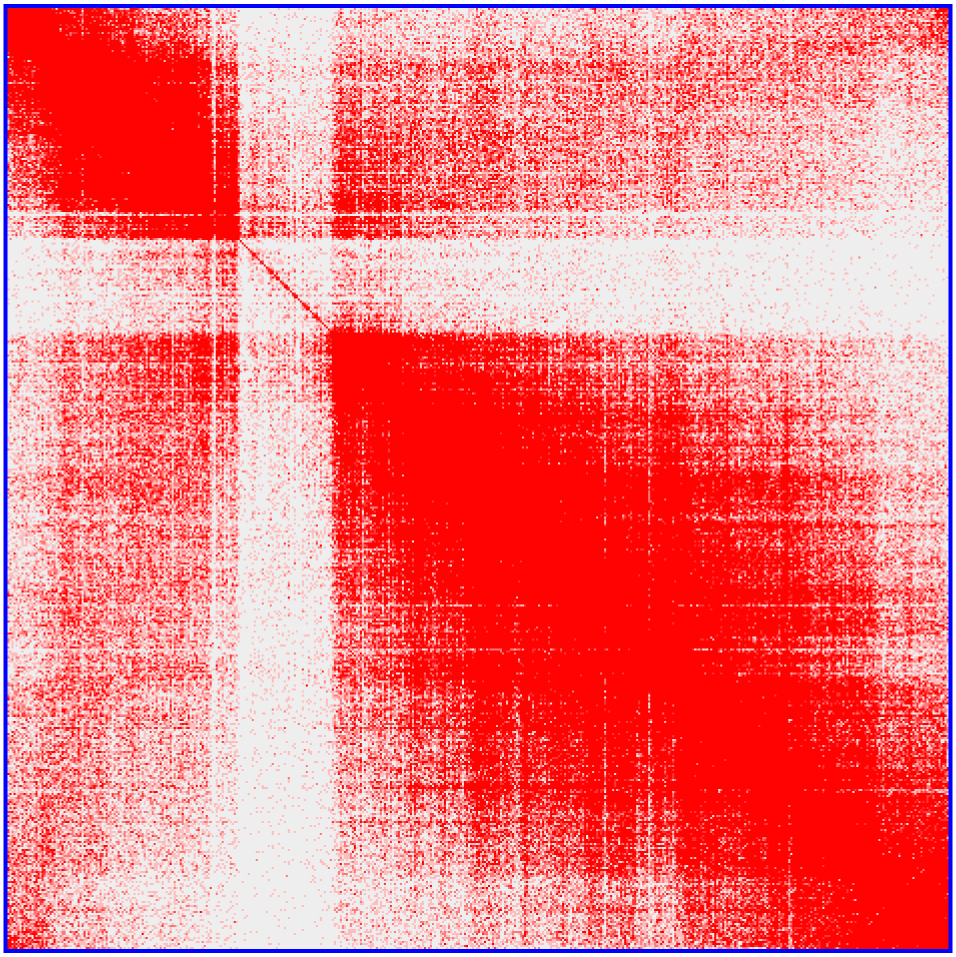

Chromosome 12 (9A)

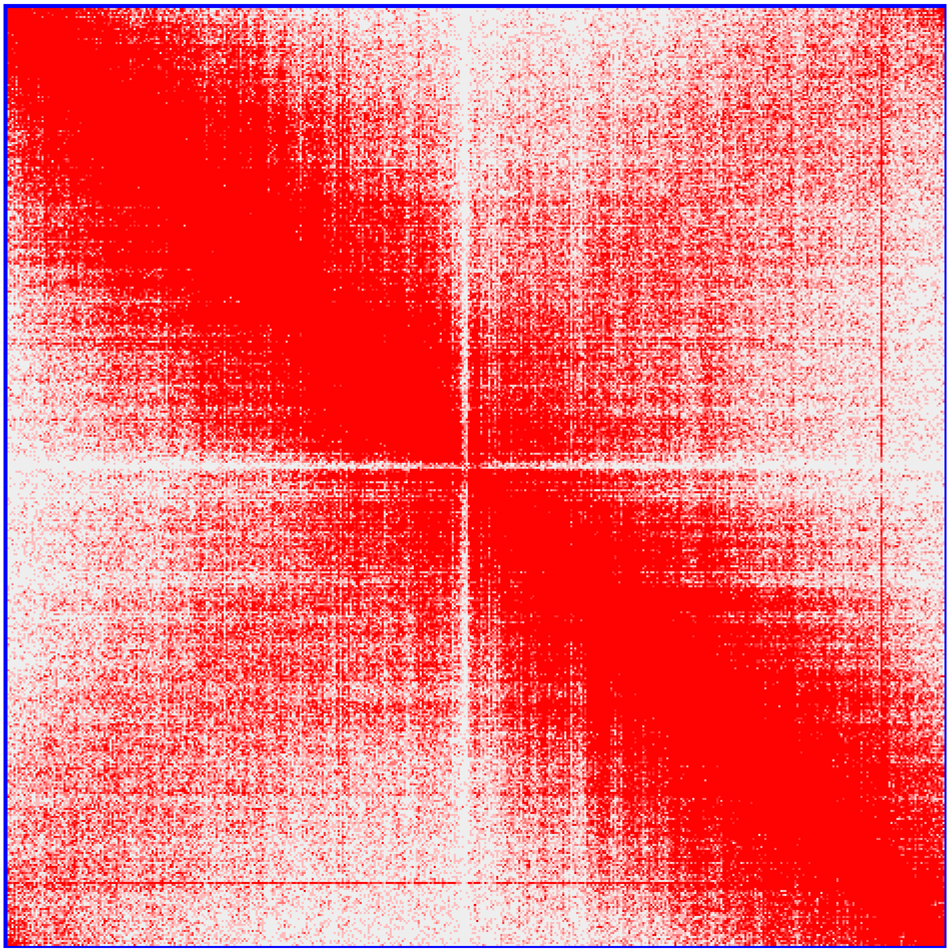

Chromosome 13 (10A)

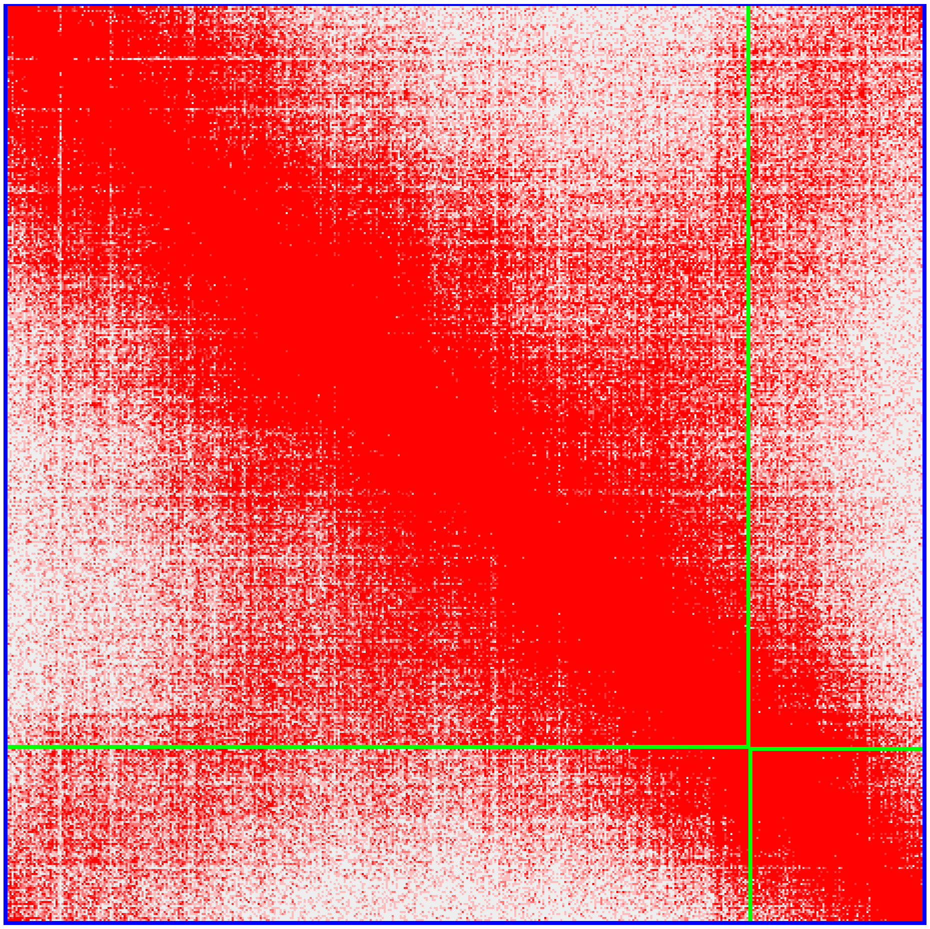

Chromosome 14 (8B)

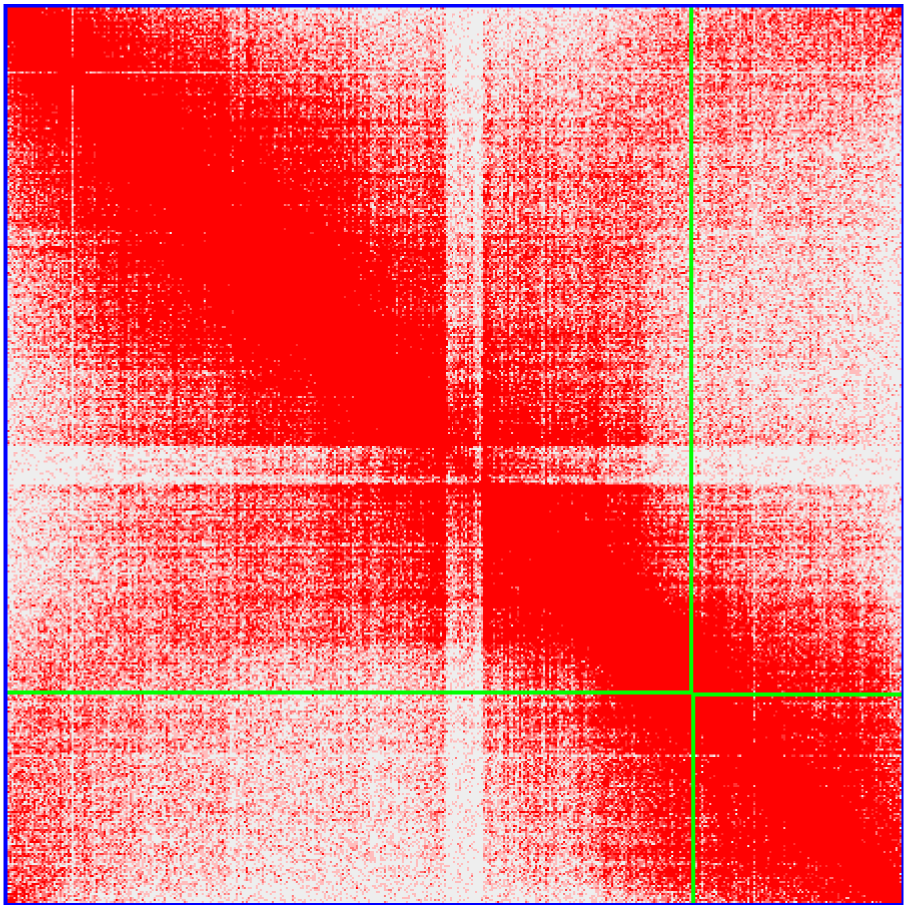

Chromosome 15 (5B)

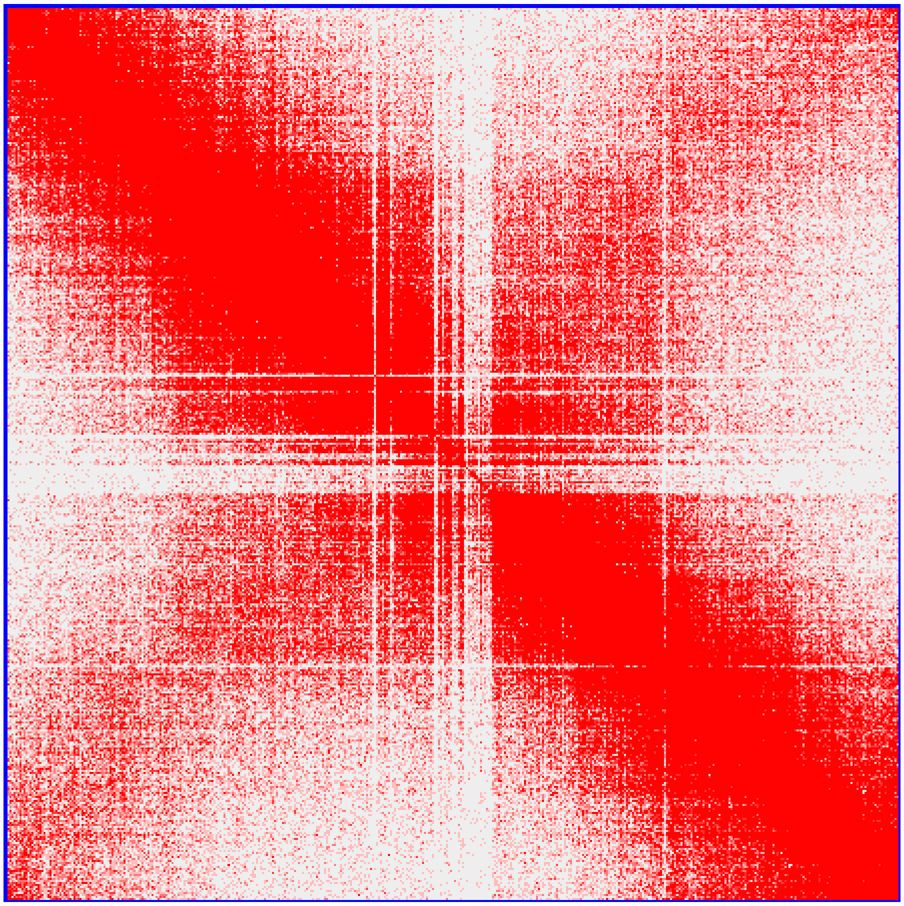

Chromosome 16 (10B)

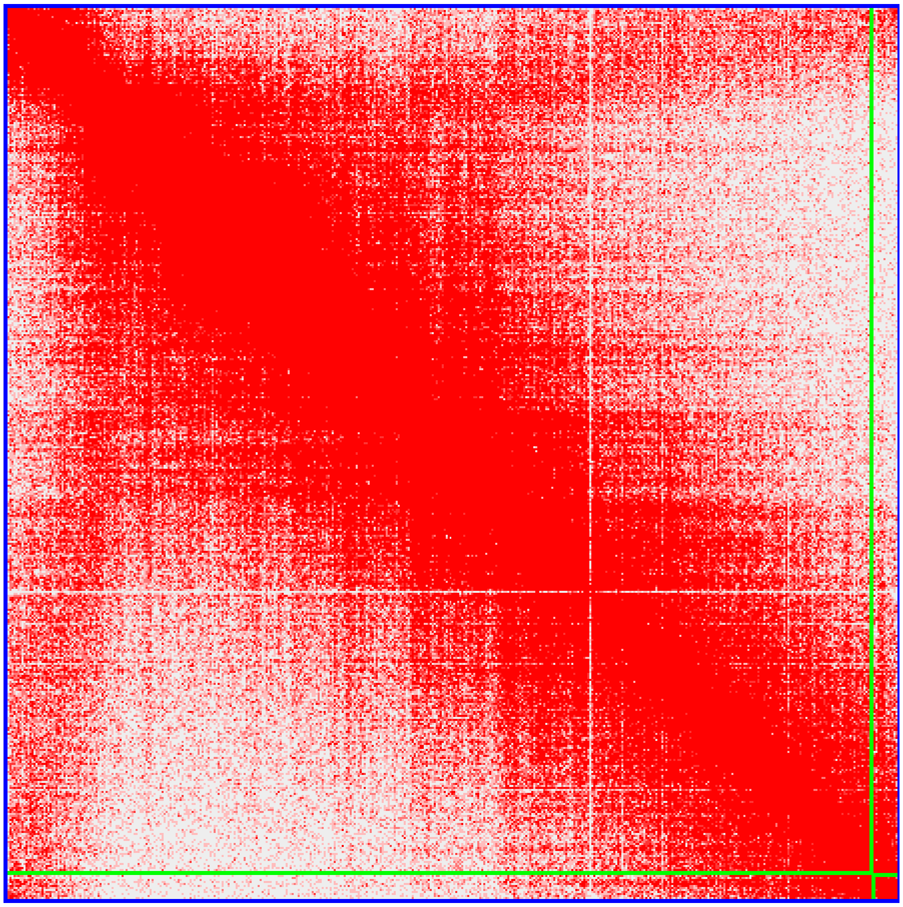

Chromosome 17 (4B)

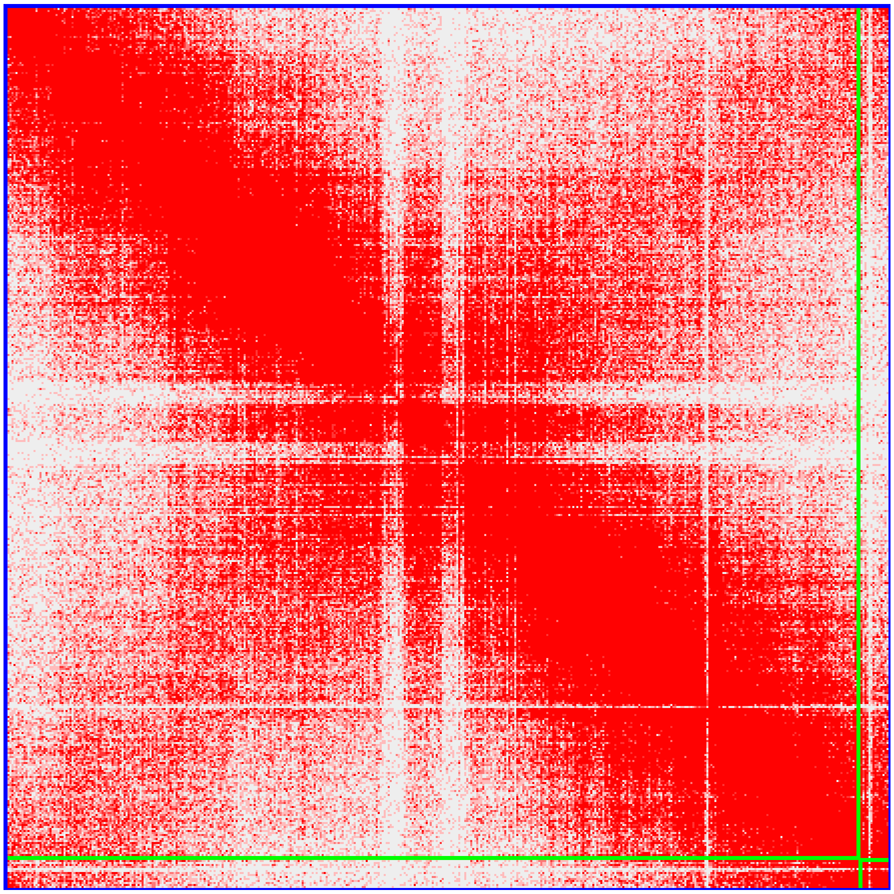

Chromosome 18 (6B)

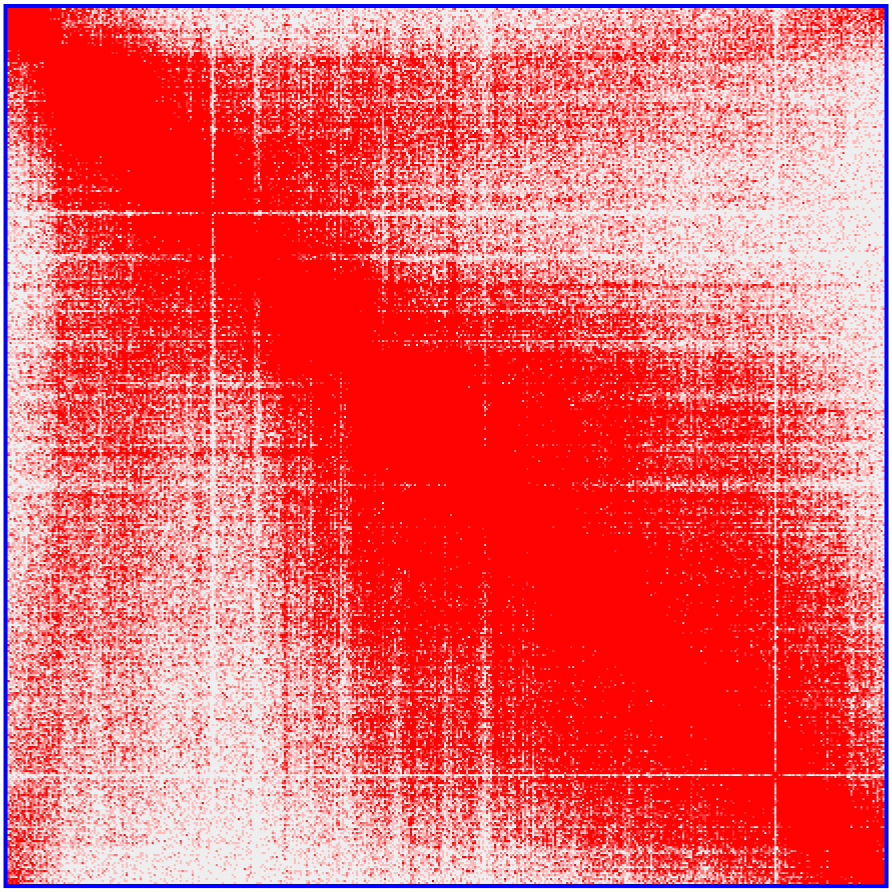

Chromosome 19 (11A)

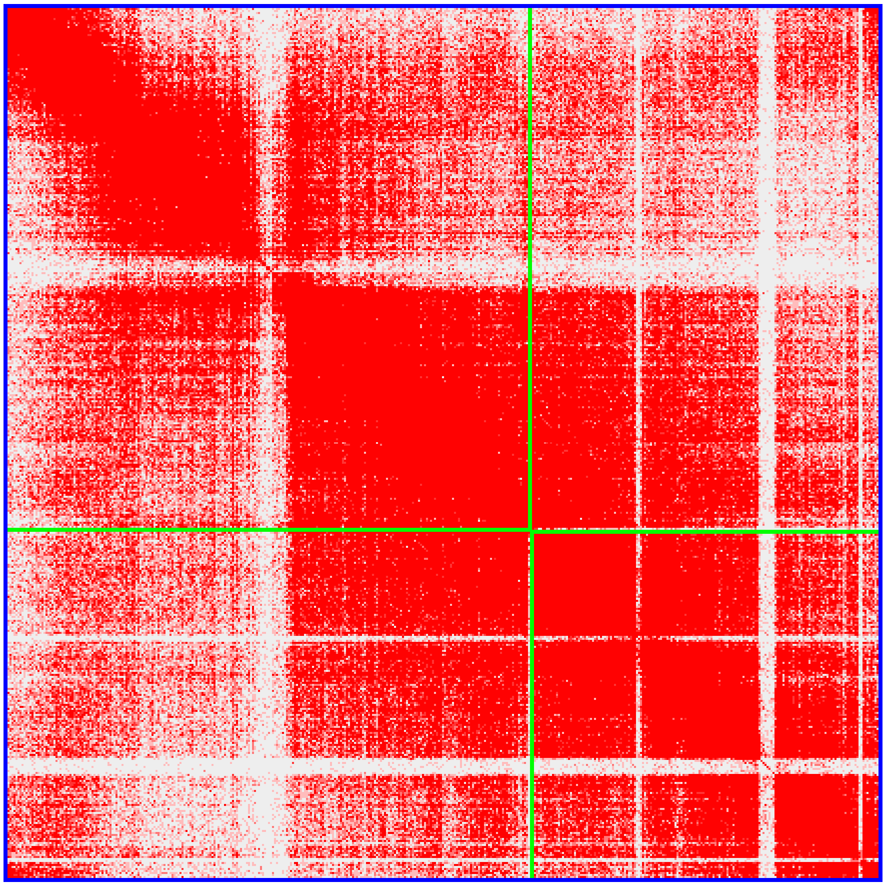

Chromosome 20 (12A)

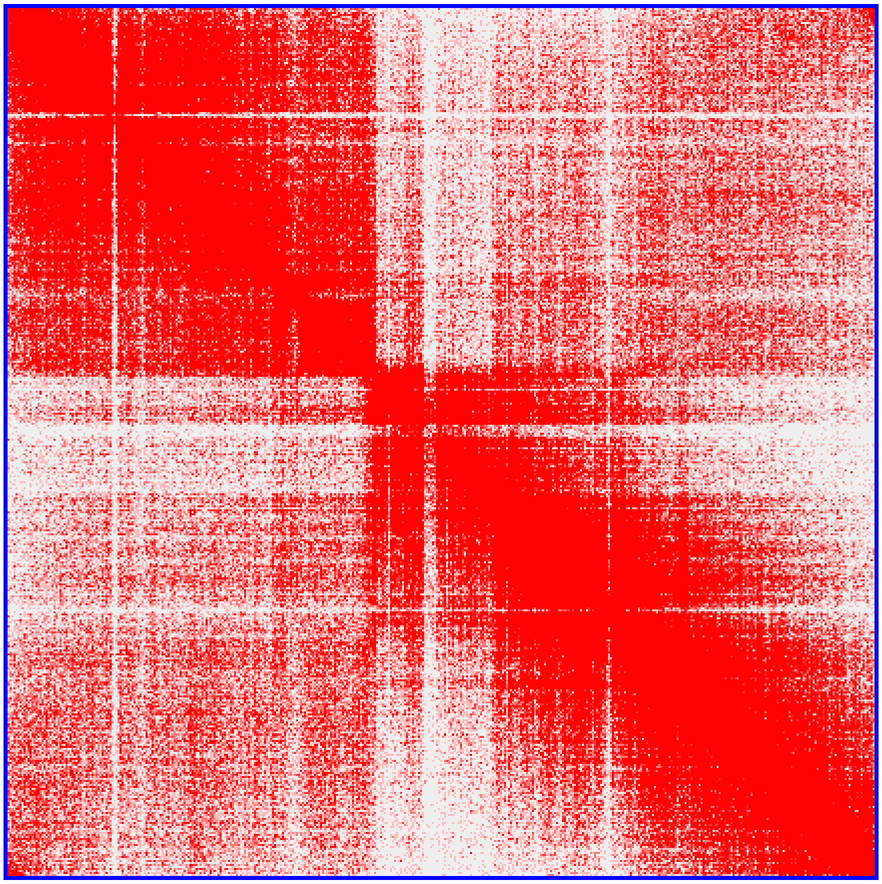

Chromosome 21 (13A)

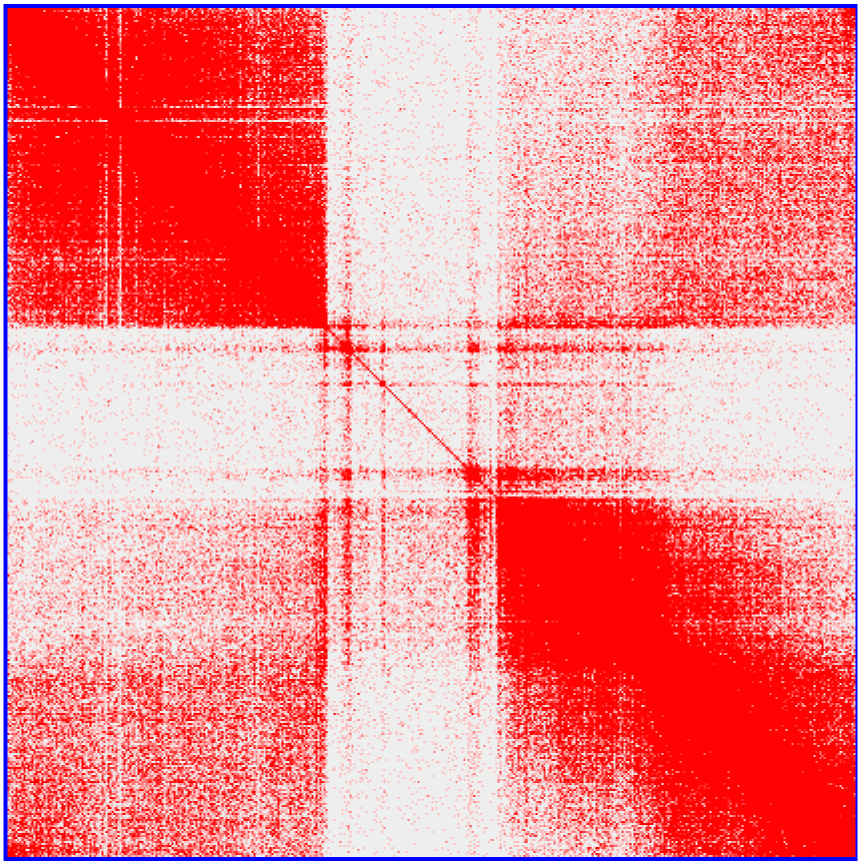

Chromosome 22 (13B)

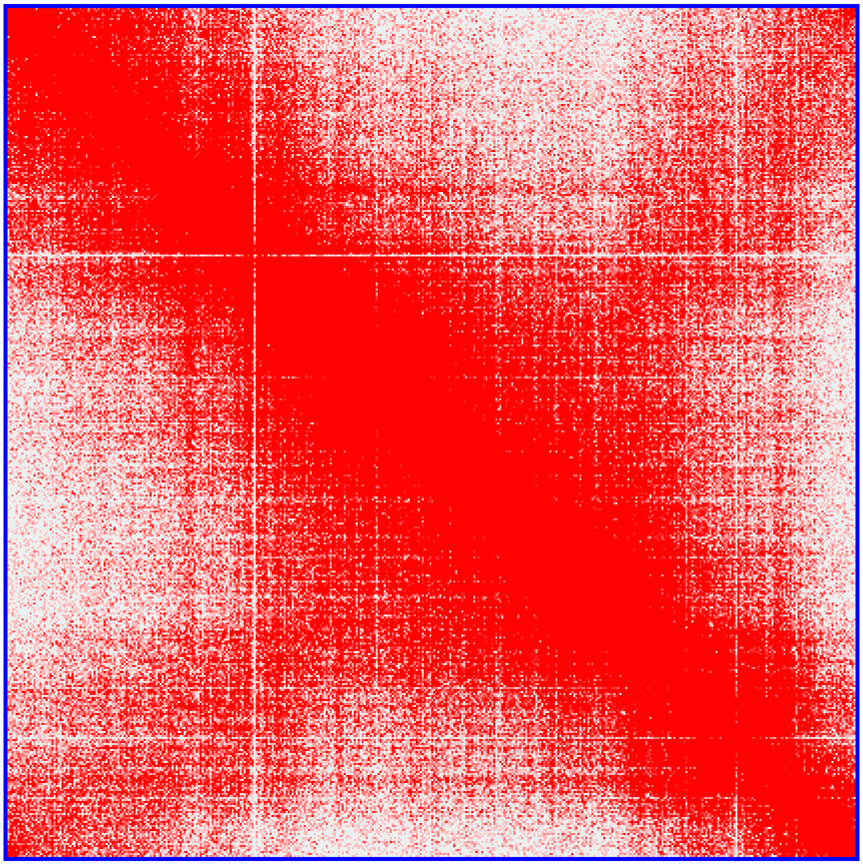

Chromosome 23 (7B)

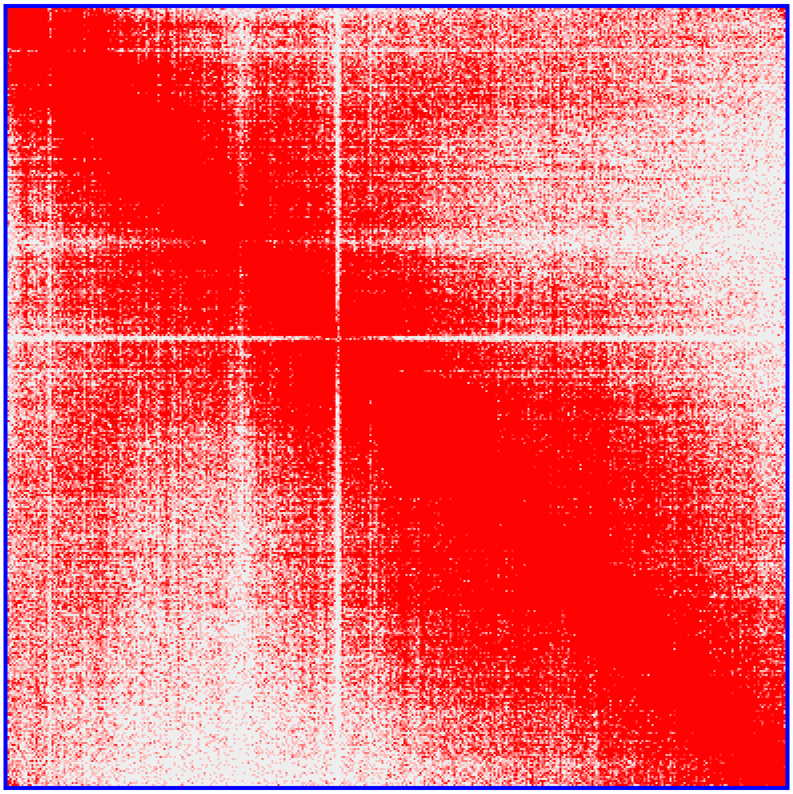

Chromosome 24 (14A)

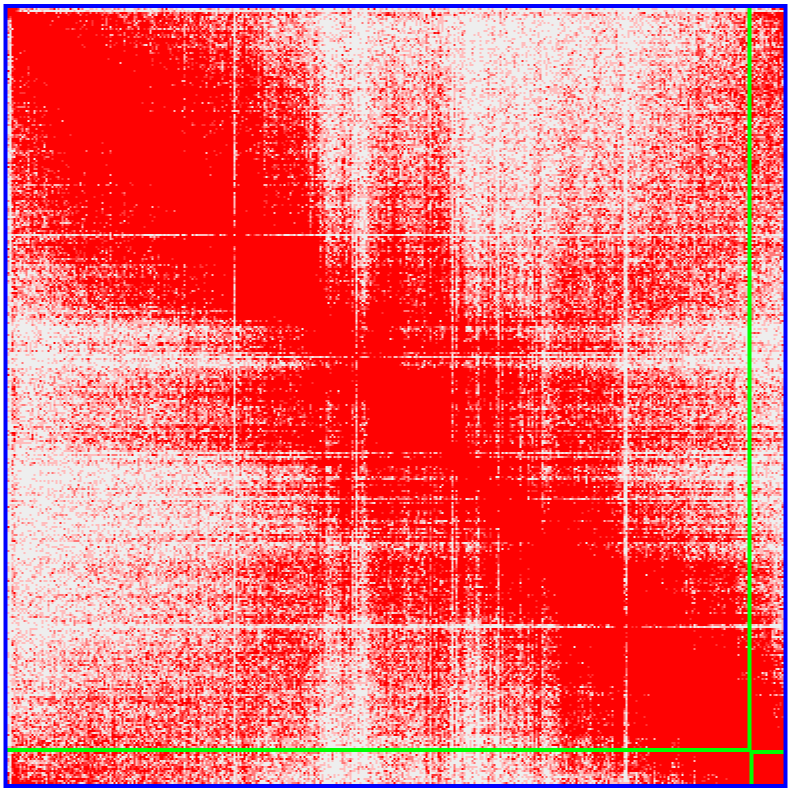

Chromosome 25 (15A)

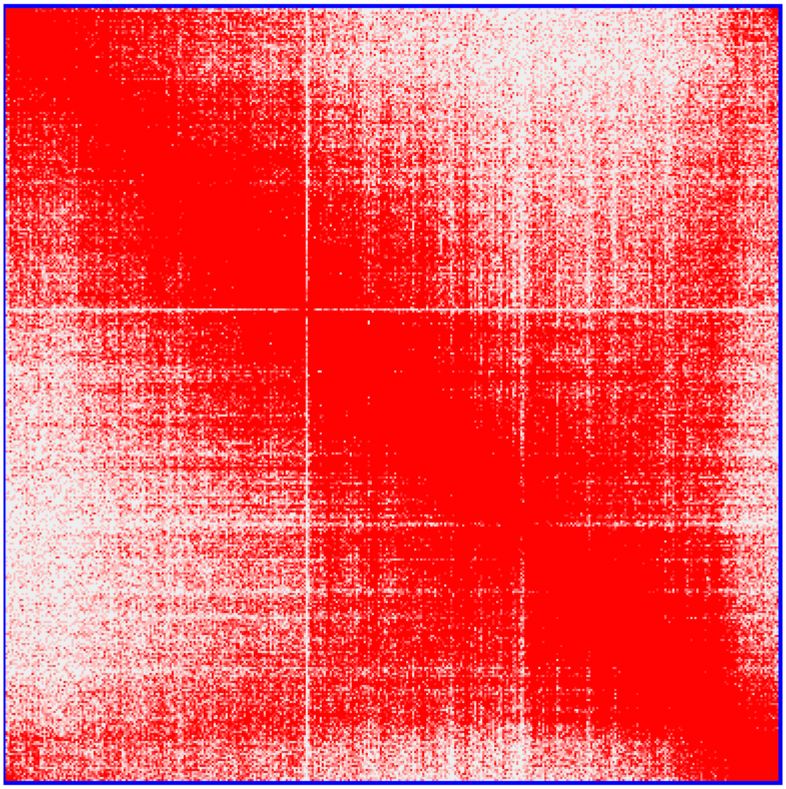

Chromosome 26 (9B)

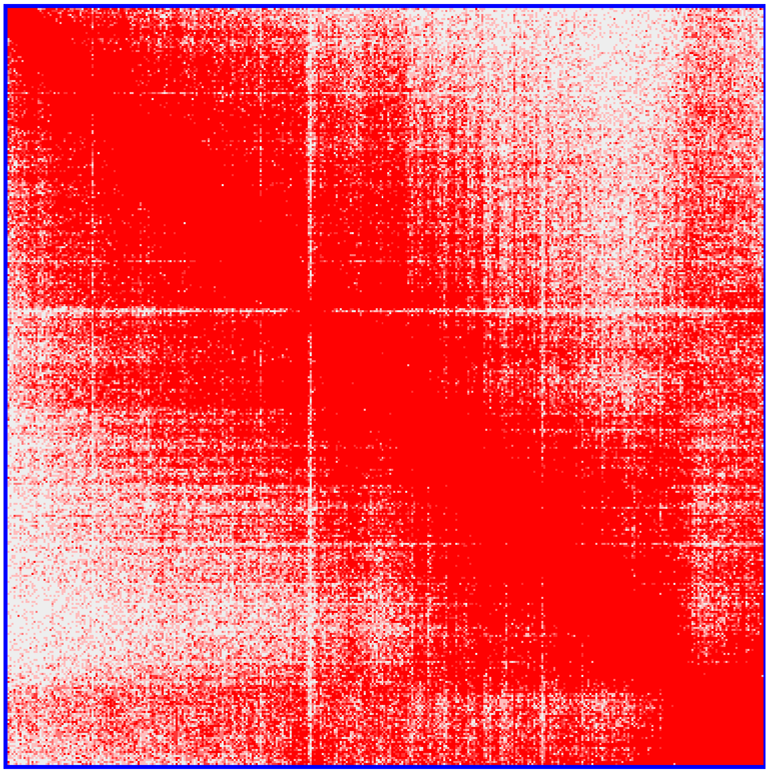

Chromosome 27 (16A)

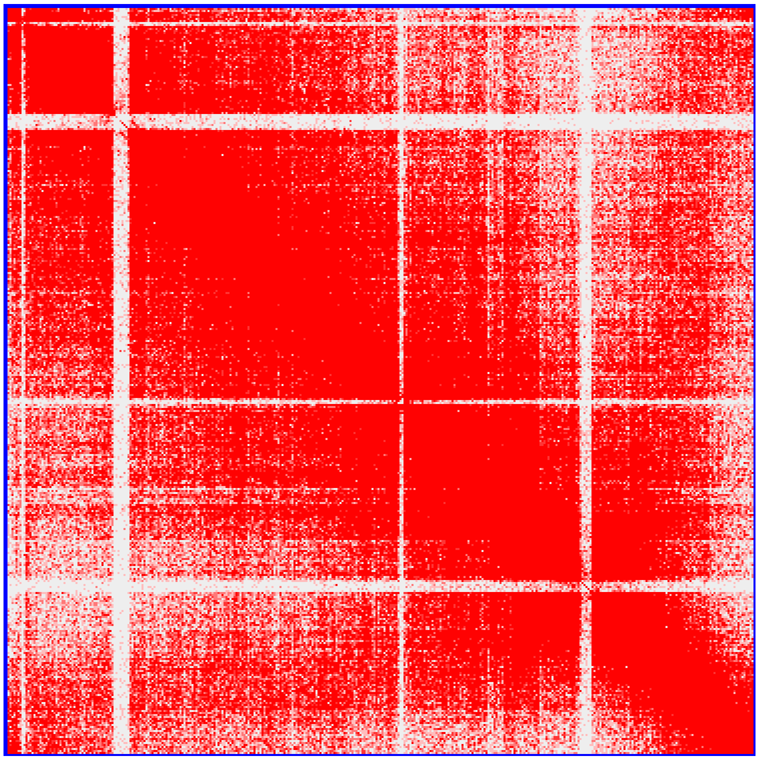

Chromosome 28 (12B)

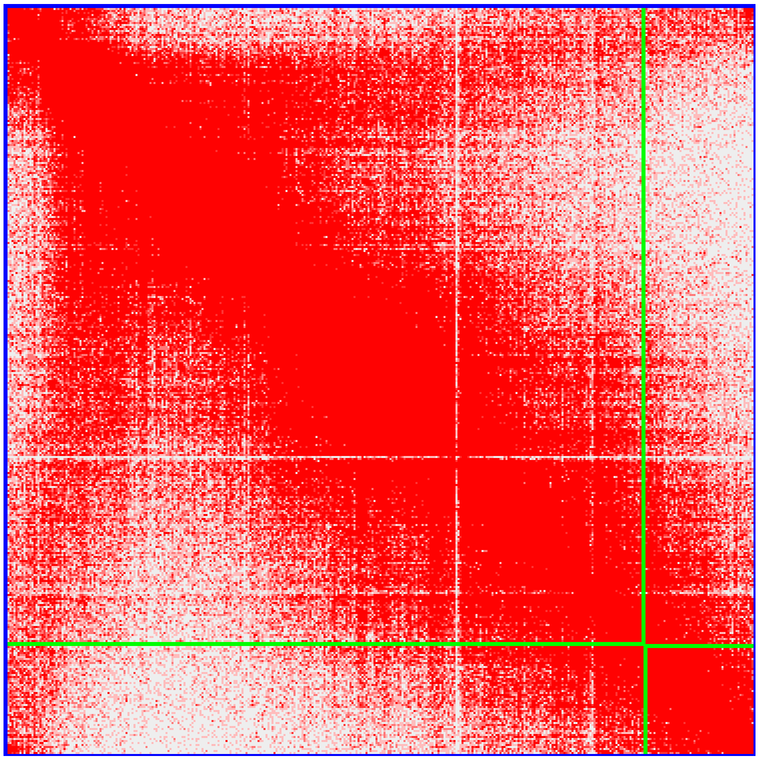

Chromosome 29 (11B)

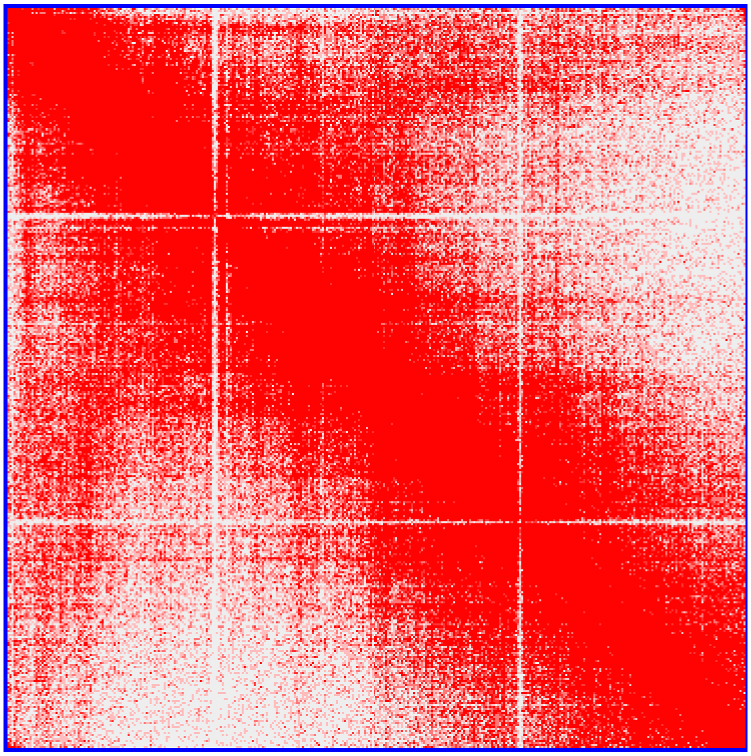

Chromosome 30 (14B)

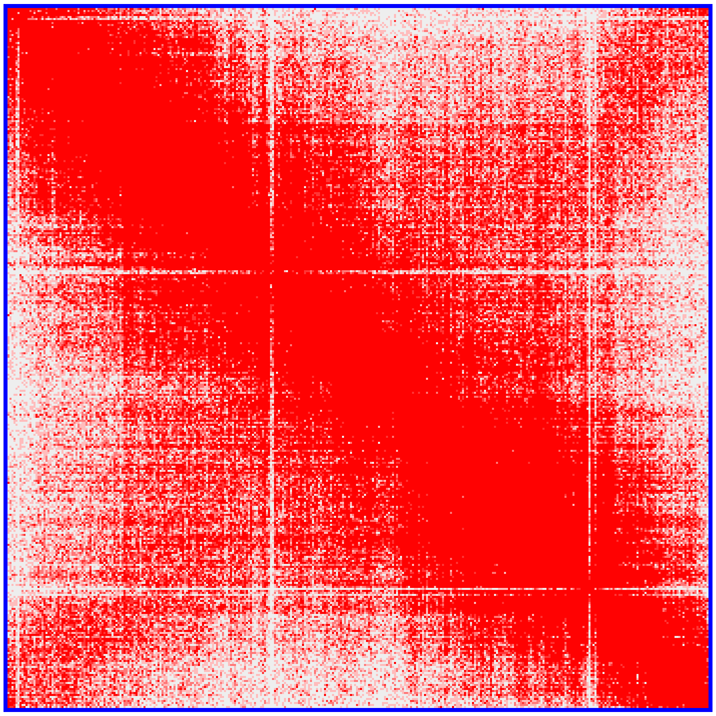

Chromosome 31 (17A)

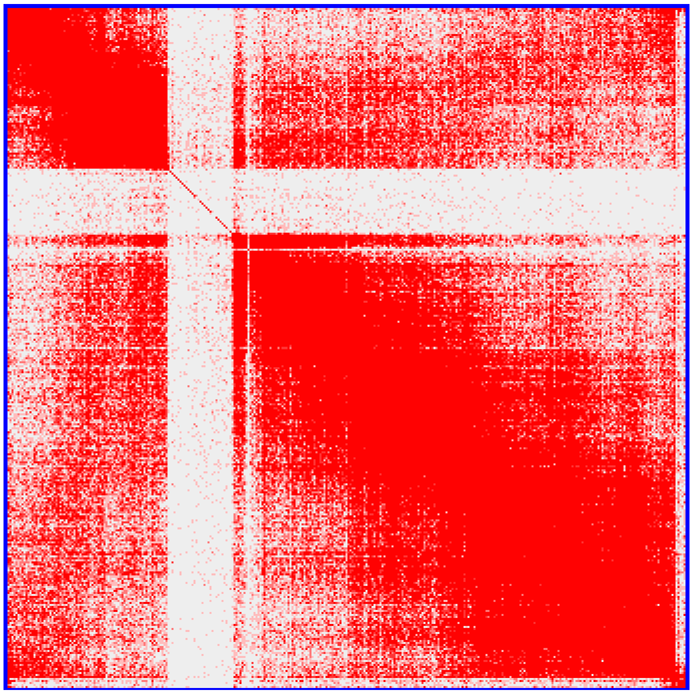

Chromosome 32 (18A)

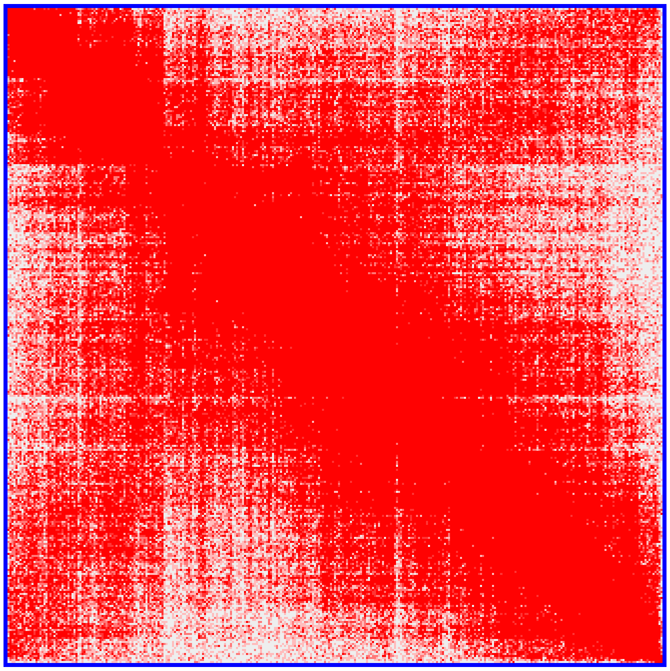

Chromosome 33 (19A)

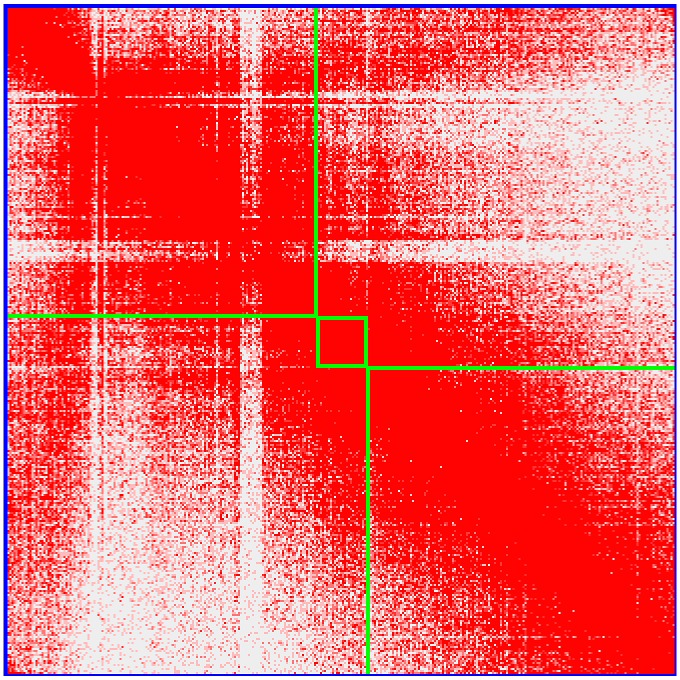

Chromosome 34 (16B)

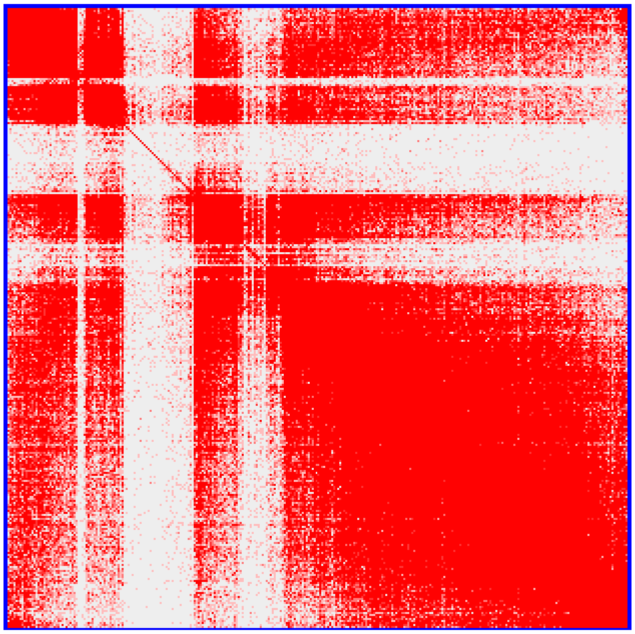

Chromosome 35 (20A)

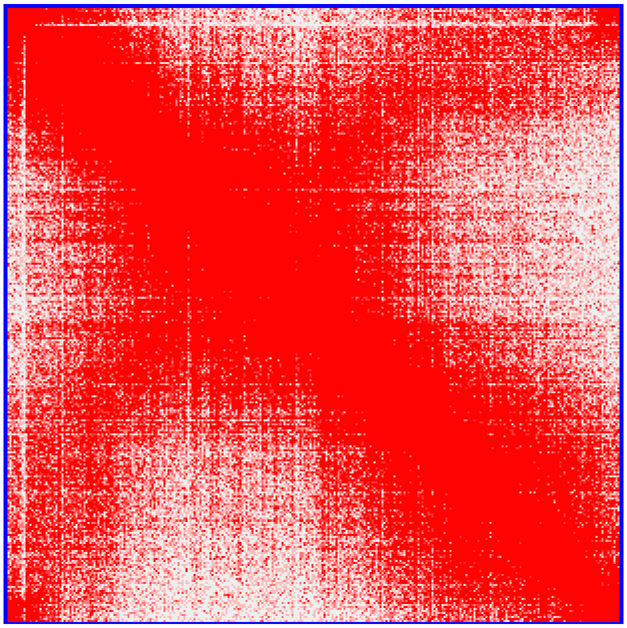

Chromosome 36 (15B)

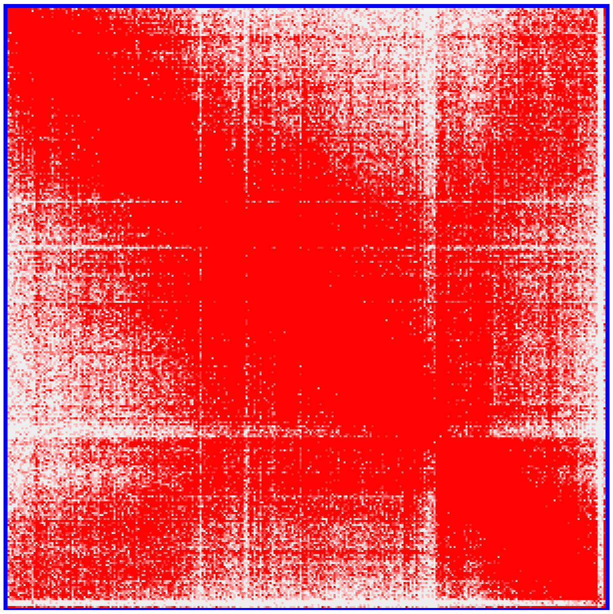

Chromosome 37 (19B)

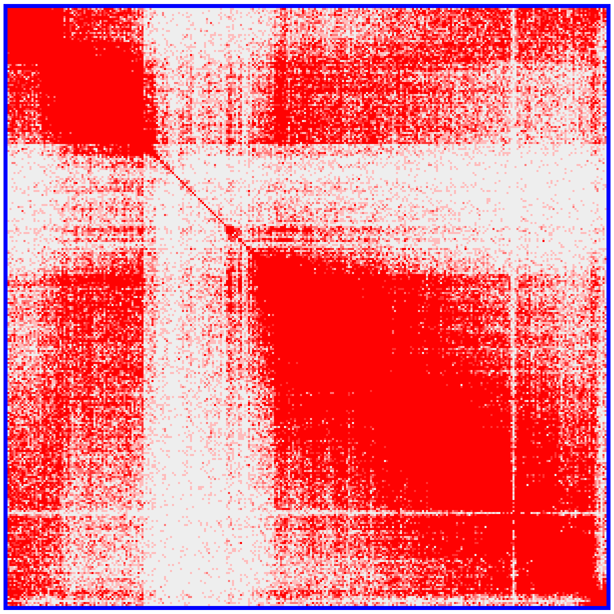

Chromosome 38 (21A)

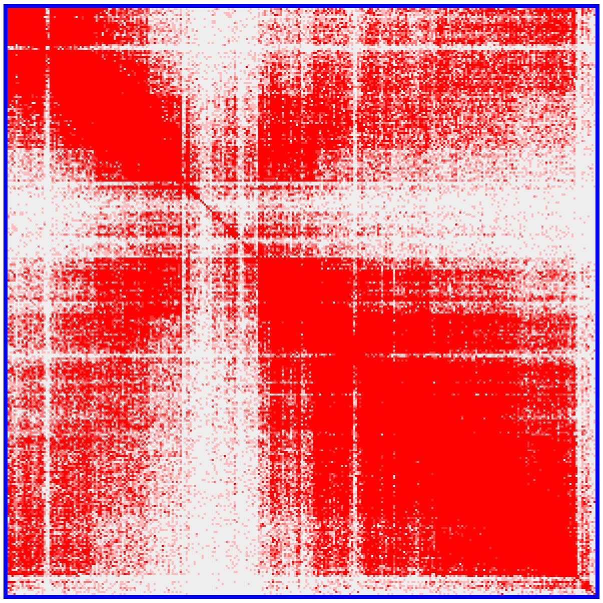

Chromosome 39 (22A)

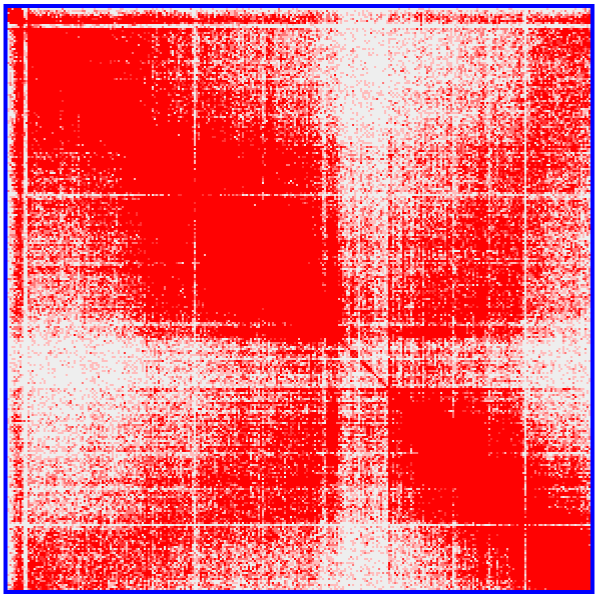

Chromosome 40 (23A)

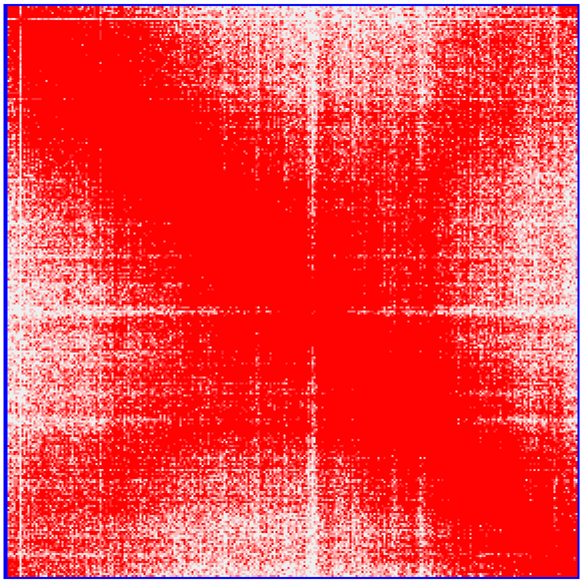

Chromosome 41 (17B)

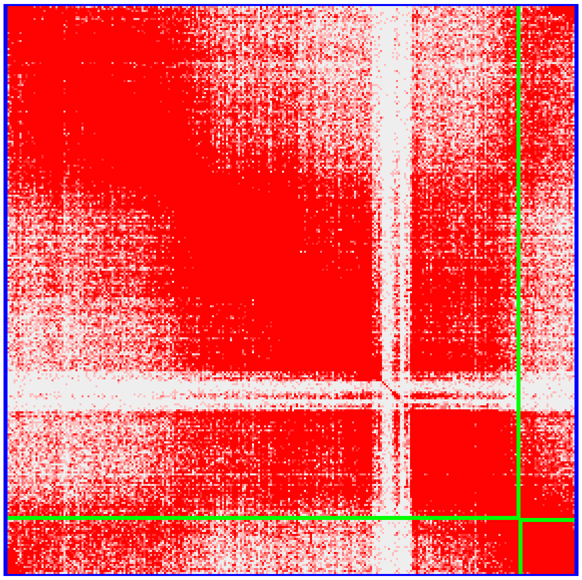

Chromosome 42 (18B)

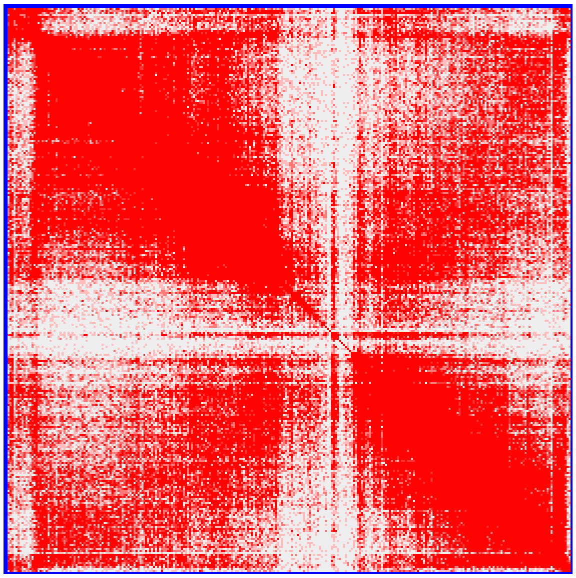

Chromosome 43 (24A)

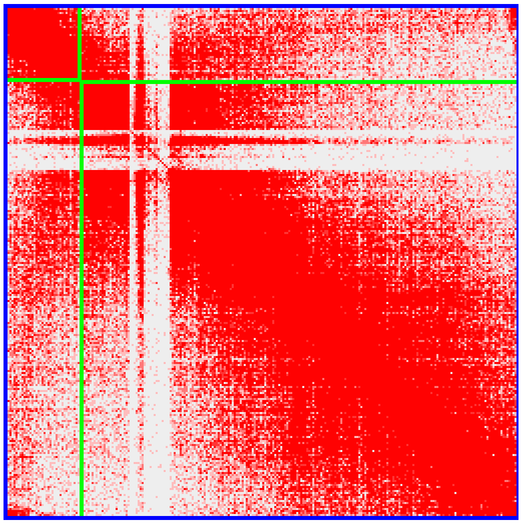

Chromosome 44 (20B)

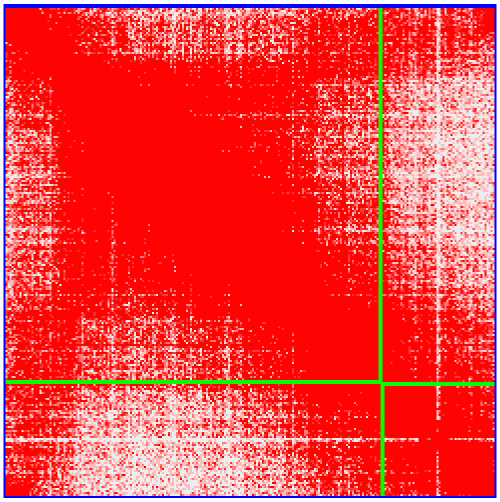

Chromosome 45 (21B)

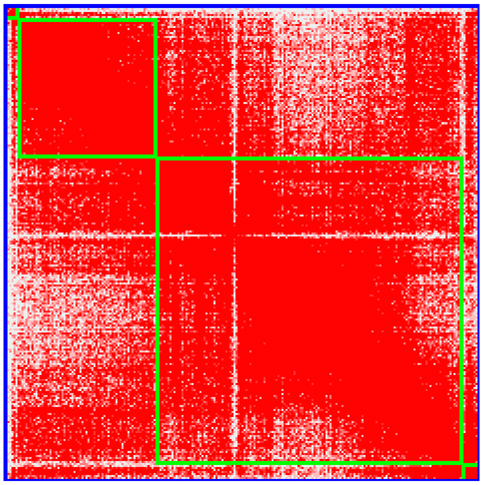

Chromosome 46 (22B)

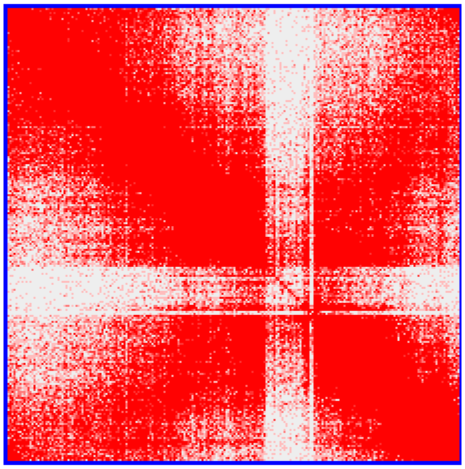

Chromosome 47 (23B)

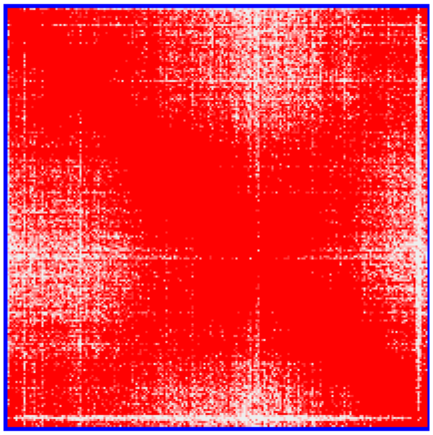

Chromosome 48 (24B)

Supplementary Figure S2. High-resolution Hi-C contact maps for all chromosomes of *Acanthus tetraploideus*. High-resolution Hi-C contact maps (100-kb resolution) for each of the 48 nuclear chromosomes of *A. tetraploideus*. These panels are snapshot images exported from Juicebox, showing intrachromosomal interaction intensity across each chromosome. Green boxes indicate boundaries of PacBio-based assembled contigs. Red dots indicate strong Hi-C contact signals, corresponding to regions with dense chromatin interactions. Repeat-rich regions identified from the PacBio-based assembly show reduced Hi-C signal intensity, as expected due to low mappability of repetitive sequences. Chromosome numbers are shown below each contact map, with the values in parentheses corresponding to the chromosome identifiers displayed in Fig. 1C.

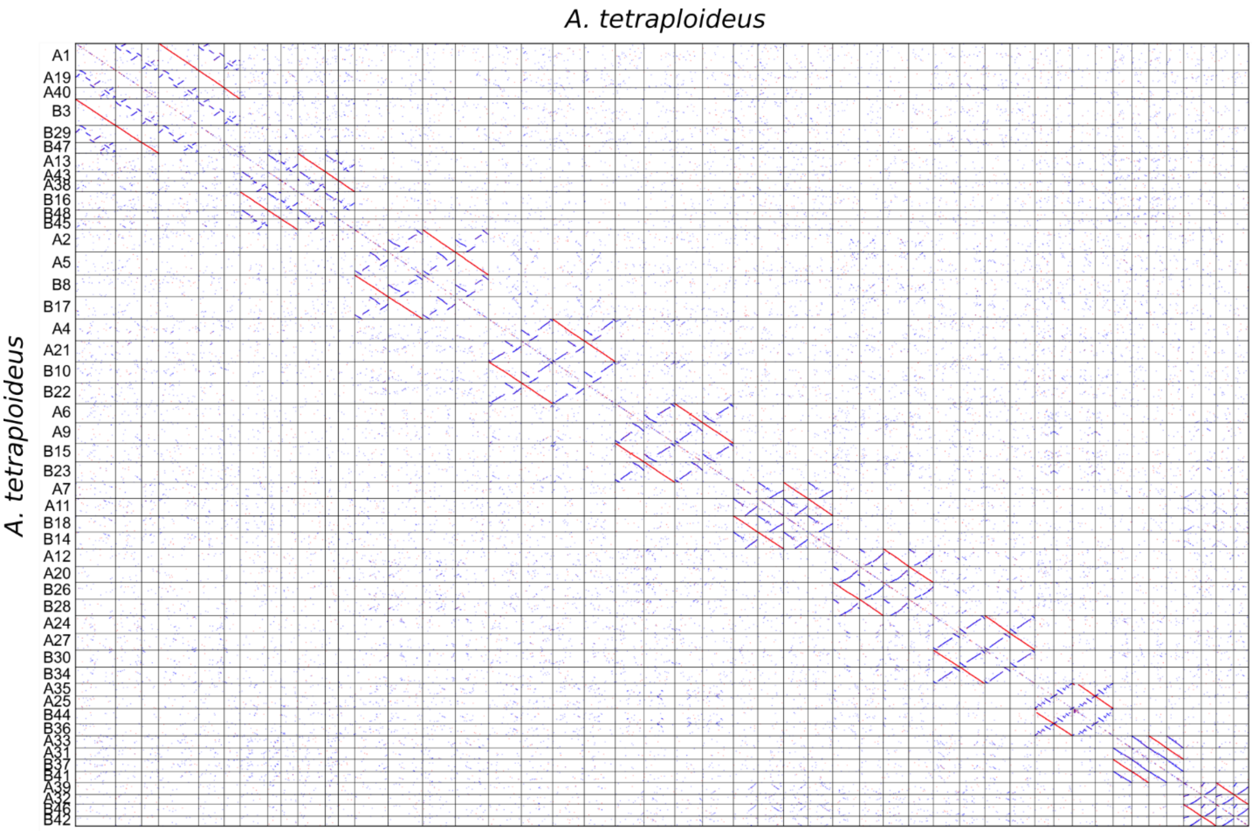

Supplementary Figure S3 Self-synteny dot plot of the *Acanthus tetraploideus* genome. The plot illustrates intragenomic synteny among chromosomes of the *A. tetraploideus* assembly. Red dots represent homoeologous matches between subgenomes SG1 and SG2, while blue dots indicate collinear regions resulting from WGD events within each subgenome. The presence of 11 diagonal syntenic blocks with dense dot patterns highlights conserved segments corresponding to 11 ancestral protochromosomes.

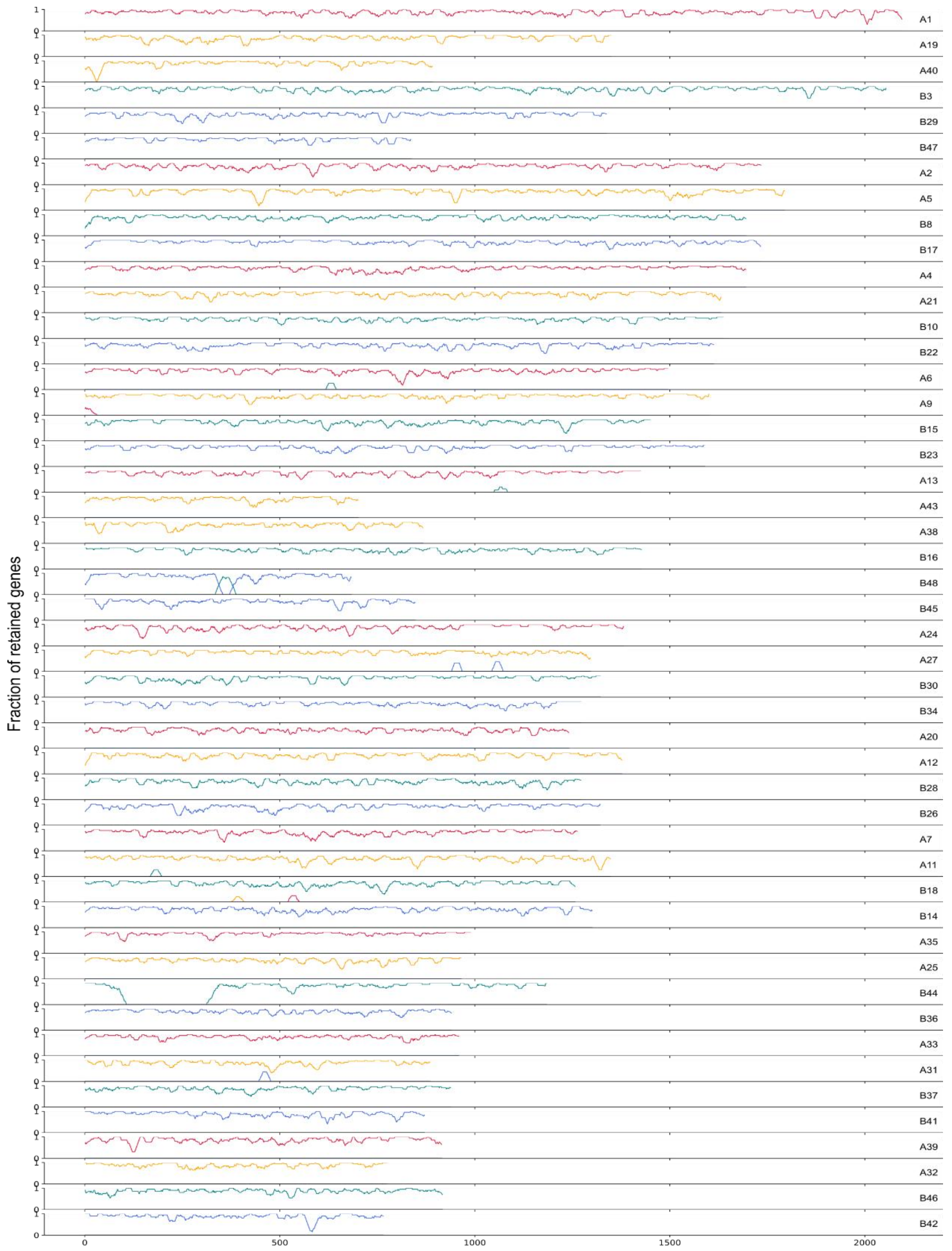

Supplementary Figure S4 Gene retention ratios along all chromosomes of *A. tetraploideus*. Ratios represent the proportion of genes retained from each progenitor, ranging from 0 to 1. Red and yellow lines indicate genes retained from the two groups of WGD-derived scaffolds in *A. ilicifolius*, whereas green and blue lines represent genes inherited from *A. ebracteatus*.

A

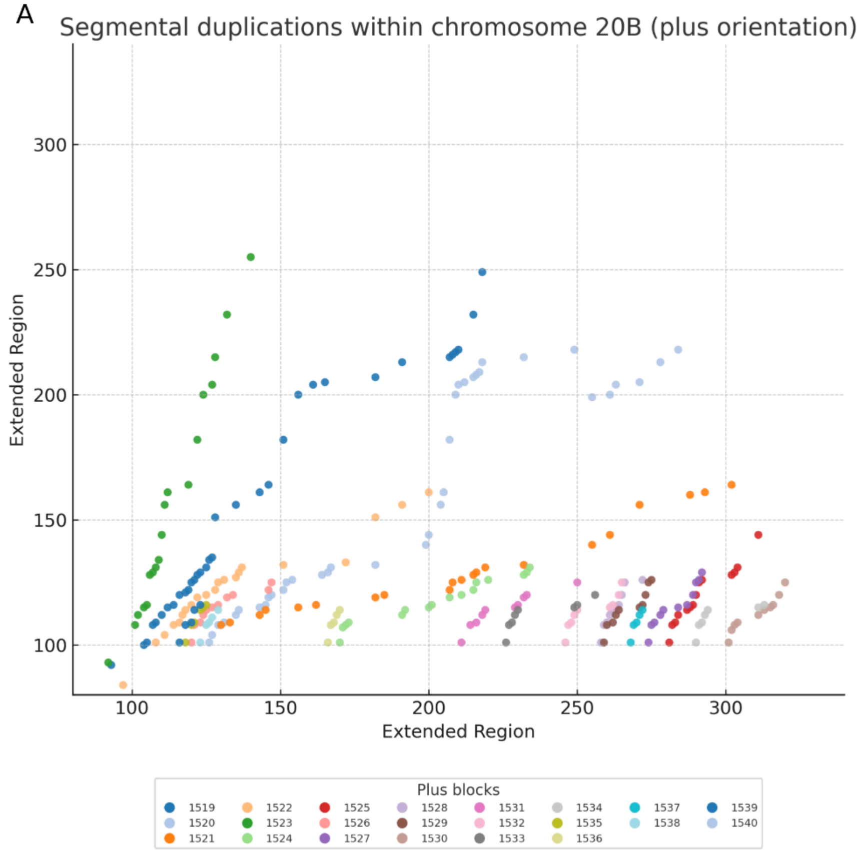
Segmental duplications within chromosome 20B (plus orientation)
Extended Region
Extended Region
Plus blocks
1519
1520
1521
1522
1523
1524
1525
1526
1527
1528
1529
1530
1531
1532
1533
1534
1535
1536
1537
1538
1539
1540

B

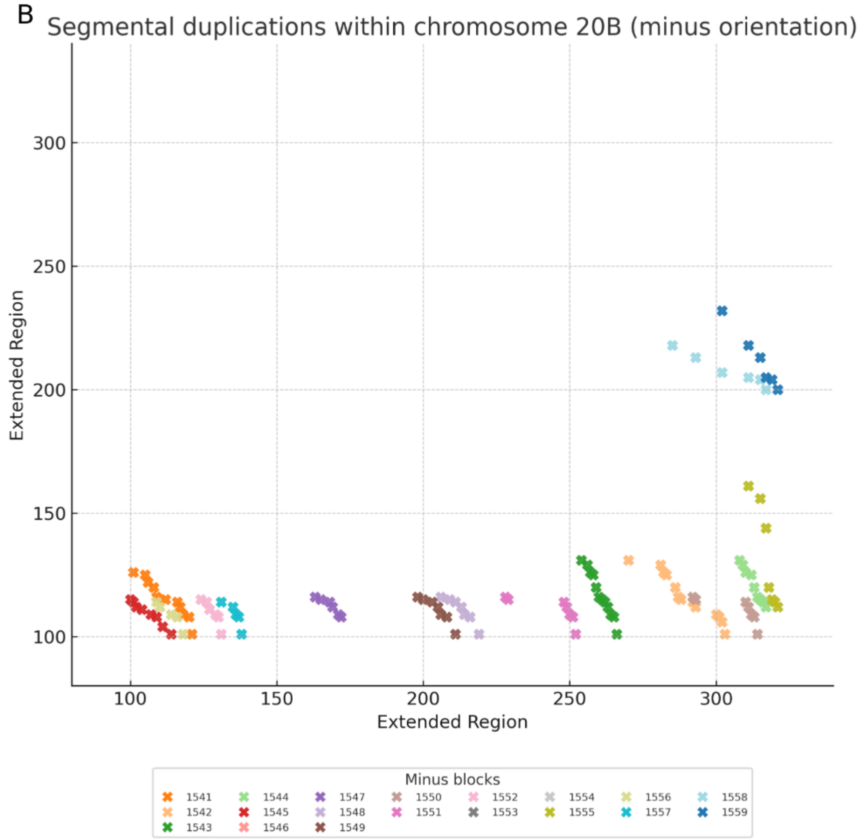
Segmental duplications within chromosome 20B (minus orientation)
Extended Region
Extended Region
Minus blocks
1541
1542
1543
1544
1545
1546
1547
1548
1549
1550
1551
1552
1553
1554
1555
1556
1557
1558
1559

Supplementary Figure S5 Segmental duplications within chromosome 20B of *Acanthus tetraploideus*. (A) Segmental duplications detected in plus orientation within the extended sequence of chromosome 20B. (B) Segmental duplications detected in minus orientation within the same region. Each colored dot represents a duplicated block, with coordinates indicating the duplicated segments along the extended region. Multiple duplication blocks are observed, suggesting extensive internal segmental duplication within chromosome 20B.
