# Supplementary material for: Genome Assembly of Three Shrub Mangroves in the Genus Acanthus Reveals Two Polyploidy Events and Expansion of Genes Linked to Root Adaptation in Coastal Habitats
Source: Gigascience. 2026 Jan 2;15:giaf162. doi: 10.1093/gigascience/giaf162 (PMC12903786; doi:10.1093/gigascience/giaf162)
Supplement: giaf162_GIGA-D-25-00249_Original_Submission [file giaf162_giga-d-25-00249_original_submission.pdf]

GigaScience

Genome Assembly of Three Shrub Mangroves in the Genus Acanthus Reveals Two Polyploidy Events and Expansion of Genes Linked to Root Adaptation in Coastal Habitats

--Manuscript Draft--

**Manuscript Number:**	GIGA-D-25-00249
**Full Title:**	Genome Assembly of Three Shrub Mangroves in the Genus Acanthus Reveals Two Polyploidy Events and Expansion of Genes Linked to Root Adaptation in Coastal Habitats
**Article Type:**	Data Note
**Funding Information:**	National Science and Technology Development Agency (NSTDA) (P2351523) — Not applicable
**Abstract:**	The genomes of mangrove Acanthus species have not been reported, despite their ecological and medicinal importance. Using PacBio and Hi-C data, we generated a chromosome-scale genome assembly of the recently identified allotetraploid species Acanthus tetraploideus (2n = 96). The genomes of diploid progenitors, A. ilicifolius and A. ebracteatus (2n = 48), were assembled from stLFR data. We identified an Acanthus-specific whole-genome duplication (WGD) event that occurred ~43 million years ago (Mya). Ancestral karyotype reconstruction revealed a shift in haploid chromosome number from 11 to 24 in the progenitors, following the WGD and subsequent chromosomal fission events. The hybridization that formed A. tetraploideus was estimated to have occurred 1.3–1.5 Mya. Phylogenomic and synteny analyses clearly showed that A. tetraploideus inherited subgenomes SG1 and SG2 from A. ilicifolius and A. ebracteatus, respectively. Gene structure and retention analyses suggested greater structural instability in the A. ebracteatus genome compared to A. ilicifolius genome. Gene family and machine learning analyses identified expansions in protein families related to Casparian strip formation, root development, and salt stress response. Several of these families were expanded in A. ilicifolius and SG1 but contracted in A. ebracteatus and SG2. These genomic patterns might have contributed to the establishment of A. tetraploideus within the habitat of A. ebracteatus. For all three species, population analysis revealed clear genetic divergence between samples from the eastern and western coasts of Thailand. This study provides valuable genomic resources and insights into the evolutionary adaptation of plants to intertidal environments.
**Corresponding Author:**	Wirulda Pootakham<br>National Science and Technology Development Agency<br>Khlong Luang, Pathum Thani THAILAND
**Corresponding Author Secondary Information:**	
**Corresponding Author's Institution:**	National Science and Technology Development Agency
**Corresponding Author's Secondary Institution:**	
**First Author:**	Wanapinun Nawae, Ph.D.
**First Author Secondary Information:**	
**Order of Authors:**	Wanapinun Nawae, Ph.D.
	Chaiwat Naktang
	Peeraphat Paenpong
	Duangjai Sangsrakru
	Thippawan Yoocha
	Sonicha U-thoomporn

Powered by Editorial Manager® and ProduXion Manager® from Aries Systems Corporation

	Wasitthee Kongkachana
	Poonsri Wanthongchai
	Suchart Yamprasai
	Chonlawit Samart
	Sithichoke Tangphatsornruang, Ph.D.
	Wirulda Pootakham, Ph.D.
**Order of Authors Secondary Information:**	
**Additional Information:**	
**Question**	**Response**
Are you submitting this manuscript to a special series or article collection?	No
**Experimental design and statistics**<br><br>Full details of the experimental design and statistical methods used should be given in the Methods section, as detailed in our Minimum Standards Reporting Checklist. Information essential to interpreting the data presented should be made available in the figure legends.<br><br>Have you included all the information requested in your manuscript?	Yes
**Resources**<br><br>A description of all resources used, including antibodies, cell lines, animals and software tools, with enough information to allow them to be uniquely identified, should be included in the Methods section. Authors are strongly encouraged to cite Research Resource Identifiers (RRIDs) for antibodies, model organisms and tools, where possible.<br><br>Have you included the information requested as detailed in our Minimum Standards Reporting Checklist?	Yes
**Availability of data and materials**<br><br>All datasets and code on which the	Yes

conclusions of the paper rely must be either included in your submission or deposited in publicly available repositories (where available and ethically appropriate), referencing such data using a unique identifier in the references and in the "Availability of Data and Materials" section of your manuscript.<br><br>Have you have met the above requirement as detailed in our Minimum Standards Reporting Checklist?	
GigaScience has policies and guidelines in place for the use of generative AI-writing tools such as ChatGPT. If you have used such writing tools to assist with writing the manuscript this must be declared and cited in the text. Authors should not list AI-writing tools and other AI-assisted technologies as an author or co-author and should acknowledge that they are fully responsible for text generated or refined by AI-writing tools.<p><br><br>A summary of use (particularly in the introduction or among methods) needs to be included at the end of the paper, and the outputs should also be included as a supplementary file hosted in GigaDB or other open repositories. Please read our guidelines for more information. <p><br><br>By submitting to GigaScience, you are aware of the journal's AI-writing tools policy, and if you have declared use of such tools below, you have acknowledged this where appropriate in your manuscript and have made a summary of use and outputs available. <p><br><b>AI-assisted writing tools have been used in the preparation of this manuscript?	No

Powered by Editorial Manager® and ProduXion Manager® from Aries Systems Corporation

Manuscript

Click here to access/download;Manuscript;Acnthus_manuscript_submit.docx

Genome Assembly of Three Shrub Mangroves in the Genus *Acanthus* Reveals Two Polyploidy Events and Expansion of Genes Linked to Root Adaptation in Coastal Habitats

Wanapinun Nawae[1], Chaiwat Naktang[1], Peeraphat Paenpong[1], Duangjai Sangsrakru[1], Thippawan Yoocha[1], Sonicha U-thoomporn[1], Wasitthee Kongkachana[1], Poonsri Wanthongchai[2], Suchart Yamprasai[2], Chonlawit Samart[2], Sithichoke Tangphatsornruang[1], Wirulda Pootakham[1*]

[1]National Center for Genetic Engineering and Biotechnology (BIOTEC), National Science and Technology Development Agency (NSTDA), Pathum Thani, Thailand

[2]Department of Marine and Coastal Resources, 120 The Government Complex, Thung Song Hong, Bangkok, Thailand

[*]Corresponding author: wirulda.poo@biotec.or.th

Abstract

The genomes of mangrove *Acanthus* species have not been reported, despite their ecological and medicinal importance. Using PacBio and Hi-C data, we generated a chromosome-scale genome assembly of the recently identified allotetraploid species *Acanthus tetraploideus* (2n = 96). The genomes of diploid progenitors, *Acanthus ilicifolius* and *Acanthus ebracteatus* (2n = 48), were assembled from stLFR data. We identified an *Acanthus*-specific whole-genome duplication (WGD) event that occurred ~43 million years ago (Mya). Ancestral karyotype reconstruction revealed a shift in haploid chromosome number from 11 to 24 in the progenitors, following the WGD and subsequent chromosomal fission events. The hybridization that formed *A. tetraploideus* was estimated to have occurred 1.3–1.5 Mya. Phylogenomic and synteny analyses clearly showed that *A. tetraploideus* inherited subgenomes SG1 and SG2 from *A. ilicifolius* and *A. ebracteatus*, respectively. Gene structure and retention analyses suggested greater structural instability in the *A. ebracteatus* genome compared to *A. ilicifolius* genome. Gene family and machine learning analyses identified expansions in protein families related to Casparian strip formation, root development, and salt stress response. Several of these families were expanded in *A. ilicifolius* and SG1 but contracted in *A. ebracteatus* and SG2. These genomic patterns might have contributed to the establishment of *A. tetraploideus* within the habitat of *A. ebracteatus*. For all three species, population analysis revealed clear genetic divergence between samples from the eastern and western coasts of Thailand. This study provides valuable genomic resources and insights into the evolutionary adaptation of plants to intertidal environments.

Introduction

The genus *Acanthus* (family Acanthaceae) consists of approximately 30 species of flowering plants distributed across tropical and subtropical regions worldwide [1]. *Acanthus* species are highly adaptive as indicated by their significant diversity in morphology and habitat preferences, ranging from terrestrial to mangrove environments [2]. While many *Acanthus* species are terrestrial, three mangrove species—*Acanthus ilicifolius*, *Acanthus ebracteatus*, and *Acanthus volubilis*—inhabit intertidal zones where saltwater and freshwater converge [1]. These species have long been used as medicinal plants across Asia and Oceania [3]. Morphologically, *A. volubilis* is clearly different from the other two species. In contrast, *A. ilicifolius* and *A. ebracteatus* share highly similar leaf morphology, characterized by lanceolate, spiny, and leathery leaves (Fig. 1A). However, their floral characteristics differ, as *A. ilicifolius* produces violet flowers with bracteoles, while *A. ebracteatus* bears smaller white flowers. Recent phylogenetic and biogeographic investigations introduced a new *Acanthus* species, *Acanthus tetraploideus*, with mixed phenotypic and genotypic characteristics from *A. ilicifolius* and *A. ebracteatus* [1]. *A. tetraploideus* has 96 chromosomes (2n = 96), double the chromosome number of *A. ilicifolius* and *A. ebracteatus* (2n = 48) [1]. Exploring the genomes of these three species will provide valuable insights into the evolution of mangrove species within challenging and dynamic coastal habitats.

The genome assembly of *Acanthus* species has not yet been reported, although several sequencing efforts have been made to understand the genetics of this lineage. For example, transcriptomic analyses identified positively selected genes that were related to salt, heat, and ultraviolet stress tolerance in *A. ilicifolius* when compared to its terrestrial relative *Acanthus leucostachyus* [2]. *A. ilicifolius* likely diverged from *A. leucostachyus* approximately 11.6 to 22.1 million years ago, and the selection of these genes was suggested to be associated with its adaptation to intertidal zones [2]. Additionally, analyses of eight nuclear genes and

transcriptome data were conducted to identify the progenitors of the allotetraploid *A. tetraploideus* genome [1,4]. These studies yet recommended that whole-genome sequencing is necessary for accurately identifying the of this hybrid the origin of this new allotetraploid species [1,4]. Genome sequencing technologies, including PacBio, Hi-C, and linked-read sequencing, have significantly advanced our understanding of complex plant genomes and their evolution [5]. These technologies have been applied to identify salinity tolerance genes and intertidal adaptations of *Avicennia marina* [6], uncover whole-genome duplication in *Ceriops tagal* [7], and reveal the origin of *Bruguiera hainesii* from the hybridization of *B. gymnorhiza* and *B. cylindrica* [8].

In this study, we generated a chromosome-level genome assembly of the tetraploid species *A. tetraploideus* using PacBio HiFi and Hi-C sequencing data. Additionally, we assembled the diploid genomes of *A. ilicifolius* and *A. ebracteatus*, the candidate parental species of the tetraploid, using stLFR sequencing. These high-quality genomic resources enable comprehensive investigations into the evolutionary history, polyploidization events, and adaptive mechanisms of *Acanthus* species. Our work marks a significant advancement in mangrove genomics and provides a valuable foundation for future research on the conservation and sustainable use of these ecologically and economically important plants.

Materials and Methods

1. Plant materials and nucleic acid isolation

Young leaf tissues were collected from mature individuals of *A. ilicifolius*, *A. ebracteatus* and *A. tetraploideus* in natural mangrove habitats in Thailand. Samples of *A. tetraploideus* and *A. ebracteatus* were collected from Samut Sakhon province at coordinates 13°30'24.9"N, 100°16'15.2"E and 13°30'32.4"N, 100°15'16.1"E, respectively. *A. ilicifolius* leaves were obtained from Phuket province at 8°09'53.6"N, 98°18'17.2"E. All leaf samples were immediately flash-frozen in liquid nitrogen in the field and stored at –80°C until processing. High-molecular-weight genomic DNA was extracted using the QIAGEN Genomic-tip 100/G kit following the manufacturer's protocol. DNA quality and integrity were assessed using a Pippin Pulse Electrophoresis System (Sage Science) and quantified with a Qubit fluorometer prior to library preparation.

For transcriptome sequencing, total RNA was isolated from leaf tissues collected from the same individual used for genome sequencing, following the protocol of Pootakham, et al. [9]. Poly(A) mRNA was enriched using the Dynabeads mRNA Purification Kit (Thermo Fisher Scientific, Waltham, USA). The integrity of RNA samples was evaluated using the Fragment Analyzer System (Agilent, Santa Clara, USA) prior to library construction.

We also collected leaf samples from 90 accessions of *A. ilicifolius*, *A. ebracteatus*, and *A. tetraploideus* across mangrove forests in 14 provinces of Thailand. Sampling sites included Chumphon (CPN), Nakhon Si Thammarat (NST), Phatthalung (PLG), Phetchaburi (PBI), Samut Songkhram (SKM), Samut Sakhon (SKN), Surat Thani (SNI), and Trat (TRT) along the Gulf of Thailand, and Krabi (KBI), Phang Nga (PNG), Phuket (PKT), Ranong (RNG), Satun (STN), and Trang (TRG) along the Andaman coast. All leaf tissues were immediately flash-frozen in liquid nitrogen and stored at –80 °C until processing. Genomic DNA was extracted using the CTAB

method as described by Pootakham et al. [10], and its quality was assessed using a Qubit fluorometer (Thermo Fisher Scientific). The genome sizes of all samples used in this study were estimated using flow cytometry on the BD Accuri™ C6 Plus system (BD biosciences) and the maize genome as a reference standard.

2. Library Preparation and Sequencing

High-molecular-weight genomic DNA was sheared to ~15 kb using the Megaruptor® 2 system. SMRTbell libraries were prepared with the SMRTbell Express Template Prep Kit 2.0 (PacBio). The libraries were purified with AMPure PB beads and size-selected (15–18 kb) using the Sage ELF system. Quality and quantity were assessed using FEMTO Pulse and Qubit. Final libraries were bound to Sequel II polymerase and sequenced on the Sequel II platform (PacBio) using an 8M SMRT Cell with 1800-minute movies. To generate chromosome-scale scaffolds, a Dovetail Omni-C (Hi-C) library was prepared by Dovetail Genomics (Scotts Valley, CA, USA). The protocol involved cross-linking chromatin with formaldehyde, digesting with DNase I, repairing ends, and ligating biotinylated adapters, followed by proximity ligation and purification. Biotinylated DNA fragments were isolated using streptavidin beads, and sequencing libraries were constructed using NEBNext Ultra reagents. The library was sequenced on an Illumina HiSeq X. PacBio HiFi and Dovetail Omni-C library preparation, quality control, and sequencing were carried out by BMKGENE (Biomarker Technologies, Beijing, China) following the provider’s standard protocols.

For linked-read sequencing of *A. ilicifolius* and *A. ebracteatus*, high-molecular-weight genomic DNA was used to construct stLFR (single-tube long fragment read) libraries using the MGIEasy stLFR Library Prep Kit (MGI Tech, Shenzhen, China). For transcriptome sequencing, polyadenylated mRNA extracted from leaf tissue was used to prepare libraries with the MGIEasy RNA Library Prep Kit v3.0 (MGI Tech). To assess genetic variation across populations,

RADseq libraries were constructed from individual samples using the MGIEasy RAD Library Prep Kit (MGI Tech). All libraries were sequenced on the MGI DNBSEQ-G400 platform.

4. Genome assembly and annotation

For *A. tetraploideus*, the genome was assembled using PacBio HiFi long reads and Hi-C scaffolding. HiFi reads were first assembled with Hifiasm 0.25 [11] in Hi-C mode to generate contigs. The Hi-C reads were mapped to the contigs following the pipeline described in Dovetail Omni-C document (https://omni-c.readthedocs.io/en/latest/index.html). Scaffolding was subsequently performed using YaHS 1.2 [12] with default parameters. Juicebox was used to curate the scaffolding results and visualize hi-c contact map. BUSCO 5.2 [13] was used to assess assembly completeness.

The stLFR reads from *Acanthus ilicifolius* and *A. ebracteatus* were assembled using stLFRdenovo (https://github.com/BGI-biotools/stLFRdenovo) using default parameters. D-GENIES1.5 [14] was used to visualize dot plots of pairwise whole-genome alignments between the allotetraploid *A. tetraploideus* genome with *A. ilicifolius* and *A. ebracteatus* genomes. RagTag 2.1 [15] was then employed to scaffold *A. ilicifolius* and *A. ebracteatus* contigs using the chromosomes of the corresponding subgenome as references.

For gene prediction, genome annotation was performed using BRAKER2 3.0 [16], which integrated RNA-seq evidence and ab initio predictions. RNA-seq data were used as extrinsic evidence to train the gene models. Viridiplantae protein sequences from the OrthoDB 11 database [17] were incorporated to support and refine gene predictions. Repeat masking was carried out using RepeatModeler and RepeatMasker prior to annotation.

5. Genome analysis

Assembly statistics, including total assembly size, contig/scaffold N50, and number of contigs were generated using QUAST 5.3 [18]. SubPhaser 1.2 [19] was used to assign homoeologous

chromosome pairs, which were obtained from D-GENIES alignments, into subgenomes SG1 and SG2. Synteny blocks were then detected using MCScanX 1.0 [20] and JCVI 1.5 [21] with their default parameters. To show the relationship between *A. tetraploideus* chromosomes and progenitor sequences, JCVI was used to visualize pairs of matched synteny blocks. Circos 0.52 [22] was used to show relationship between subgenomes SG1 and SG2. To identify whole-genome duplication events, MUSCLE 5.3 [23] and yn00 program of the PAML 4.9 package [24] were used under WGDI environment [25] to calculate and plot Ks values. WGDI was also employed to reconstruct ancestral chromosomes of all studied *Acanthus* species based on sorted dot plots of shared synteny blocks. Additionally, gene retention from the parental genomes on *A. tetraploideus* chromosomes was identified using WGDI. The Liftoff program was used to transfer the annotations from reference to target sequences. The completeness of the sequences within the transferred annotation region was checked to investigate possible structural aberrant of unretained genes. Homologous genes used as inputs for MCScanX, JCVI, and WGDI were identified using BLASTP with e-value cutoff of 10^{-10}.

6. Comparative genomics

Protein sequences from *A. tetraploideus*, *A. ilicifolius*, *A. ebracteatus*, and additional 16 plant species, including *Nypa fruticans*[26], *Lumnitzera racemosa* [27], *Combretum micranthum* [27], *Sonneratia alba* [28], *Sonneratia caseolaris* (https://evolution.sysu.edu.cn/Sequences.html), *Bruguiera parviflora* [29], *Rhizophora apiculate* [30], *Kandelia obovata* [31], *Ceriops tagal* [7], *Aegiceras corniculatum* [32], *Olea europaea* (GCF_002742605.1), *Rehmannia glutinosa* [33], *Salvia hispanica* (GCF_023119035.1), *Avicennia marina* [34], *Strobilanthes cusia* [35], *Andrographis paniculata* [36] were used to identify orthologous groups (orthogroups) using OrthoFinder 2.5 [37]. The protein sequences of single-copy orthologs identified by OrthoFinder were aligned using MUSCLE 5.3 [23]. Poorly aligned regions were trimmed by trimAl 1.5 with heuristic mode (-automated1 option) [38]. A phylogenetic tree was inferred from the processed

alignment using RAxML-NG 1.2 [39] under the best-fit substitution model determined by ModelTest-NG 0.1 [40]. We used MCMCTree implemented in the PAML 4.9 [24] to calculate divergence times among species in the tree based on fossil calibration times obtained from and the Timetree database and associated references therein. Transposable element divergence rates and genome merger time were identified following the pipline described in [41].

Gene family size changes were analyzed using CAFE 5 [42]. The OrthoFinder gene count table and the calibrated phylogenetic tree were provided as input. A global λ parameter was estimated, and families with significantly expanded or contracted sizes ($p < 0.05$) were identified. We additionally applied machine learning techniques to identify the most predictive orthogroups for lineage classification between the *Acanthus* group (class A) and the non-*Acanthus* group (class B). To enrich the feature space with phylogenetically informative signals, we assessed the phylogenetic signal of each orthogroup by calculating Blomberg's K and Pagel's λ from the species phylogenetic tree and gene count matrix. These calculations were implemented using the phylosig() function in the R package phytools 2.0 [43]. Orthogroups with intermediate conservation signals ($K > 1$ and $\lambda > 0.5$) were retained for downstream analysis. Next, Welch's *t*-test with a p-value threshold of 0.1 was used as a preliminary screening step to identify orthogroups showing relatively distinct protein count patterns between the two classes. This step was followed by recursive feature elimination using three classification algorithms, including logistic regression, random forest and gradient boosting, implemented with the scikit-learn library in Python. For each algorithm, orthogroups were ranked by their relative importance and the top twenty were considered lineage-associated gene families.

Results

1. Genomes of *Acanthus tetraploideus* and its progenitors

We generated a high-quality genome assembly of *Acanthus tetraploideus* using a combination of PacBio HiFi and Hi-C sequencing data. PacBio HiFi sequencing produced 63 Gb of long-read data, with an average read length of 14.36 kb and a sequencing depth of 33X. The HiFi data assembly yielded a total contig length of 1.95 Gb and a contig N50 of 42.67 Mb, closely matching the estimated genome size (Supplementary Table S1). The contigs were scaffolded with a total of 230 Gb of Hi-C data into 48 chromosomes, with a total length of 1.92 Gb and a scaffold N50 of 43.75 Mb (Fig. 1A and Supplementary Table S2). Telomeric repeats (AAACCCT) were identified at both ends of 44 chromosomes, at a single end of three chromosomes, and were absent from only one chromosome (Supplementary Table S3). All chromosomes exhibited high repeat density near their centers, corresponding to centromeric regions (Fig. 1B). Benchmarking Universal Single-Copy Orthologs (BUSCO) analysis indicated that 99.2% of conserved Embryophyta genes were present (Supplementary Table S4), demonstrating the high completeness of the *A. tetraploideus* assembly. These results indicated a high completeness level of the assemble *A. tetraploideus* genome. BUSCO analysis revealed that duplicated genes accounted for 95.5% of the *A. tetraploideus* genome, reflecting the polyploid nature of the assembled genome. Further analysis of orthologous chromosome pairs using SubPhaser identified two clearly separated subgenomes, SG1 (1.03 Gb) and SG2 (0.89 Gb). Synteny block analysis confirmed a 1:1 relationship between SG1 and SG2 across the genome (Fig. 1B).

The genomes of *Acanthus ilicifolius* and *Acanthus ebracteatus*, the two putative progenitors of *A. tetraploideus*, were sequenced using the stLFR technique. The assembled genome sizes were 0.98 Gb for *A. ilicifolius* and 0.89 Gb for *A. ebracteatus* (Supplementary Table S5-S6). These numbers were also consistent with the genome sizes estimated by flow cytometry (Supplementary Table S7-S8). BUSCO analysis indicated that both assemblies contained 98% of Embryophyta conserved genes, with only 11% classified as duplicated genes (Supplementary Table S9-S10). Genome alignments showed that SG1 of *A. tetraploideus* was more similar to

the *A. ilicifolius* assembly than to *A. ebracteatus*, while SG2 showed higher similarity to *A. ebracteatus* (Supplementary Table S11). Based on these matches, the contigs of *A. ilicifolius* and *A. ebracteatus* were scaffolded using SG1 and SG2 as reference sequences, respectively. The scaffolding resulted in 24 pseudochromosomes for each species, covering 93% and 94% of the initial lengths of the *A. ilicifolius* and *A. ebracteatus* assemblies, respectively. The genome annotation revealed 61,044, 30,210 and 30,799 protein-coding gene models in the *A. tetraploideus*, *A. ilicifolius* and *A. ebracteatus*, respectively (Supplementary Table S12-S14). Synteny analysis of conserved gene order among genomes clearly demonstrated that *A. tetraploideus* inherited SG1 from *A. ilicifolius* and SG2 from *A. ebracteatus* (Fig. 1C).

2. Ancestral karyotypes

A dot plot from pairwise comparisons of all chromosomes within the *A. tetraploideus* genome revealed 11 protochromosomes in ancestral karyotype, each defined by conserved intervals of syntenic blocks (Supplementary Fig. S1). Chromosome mapping indicated that 18 chromosomes in each subgenome (SG1 and SG2) aligned with nine ancestral chromosomes in a 2:1 ratio. These results represented the signal of a whole-genome duplication (WGD) event experienced by the ancestor of the Acanthus lineage. The dot plot also showed that most duplicated chromosome pairs displayed extensive rearrangements, with numerous interleaved syntenic fragments, indicating that the WGD occurred long before the divergence of the progenitor lineages (Fig. 2A). Interestingly, the remaining six chromosomes in each subgenome aligned with the remaining two ancestral chromosomes in a 3:1 ratio (Fig. 2A). For example, ancestral chromosome 1 aligned with chromosomes 1A, 11A, and 23A of SG1, and 1B, 11B, and 23B of SG2. In SG1, for example, chromosome 11A corresponded to approximately two-thirds of 1A, while 23A aligned with the remaining region, indicating that the two duplicated chromosomes were fragmented in the common ancestor of *A. ilicifolius* and *A. ebracteatus*.

Based on these results, we propose that the ancestral lineage of *Acanthus* and related species originally had 11 chromosomes (Fig. 2B). A WGD event doubled the haploid chromosome number to 22 (2n = 44). Subsequent chromosomal fissions increased the haploid chromosome number to 24 (2n = 48). This karyotype has been maintained in both *A. ilicifolius* and *A. ebracteatus*. Hybridization between these two species further raised the haploid chromosome number to 48 (2n = 96), resulting in the formation of the allotetraploid *A. tetraploideus*. The karyotype analysis also showed small segments of different color embedded within the main bodies of some representative chromosomes of *A. tetraploideus*, *A. ilicifolius*, and *A. ebracteatus*, suggesting that the genomes underwent subtle chromosomal translocations and structural rearrangements (Fig. 2B). These structural modifications indicated limited genomic exchange between the two subgenomes following polyploid formation, implying that *A. tetraploideus* was a recently formed species.

3. Genome evolution

Synteny analysis suggested that the WGD event occurred before the hybridization of *A. ilicifolius* (Ai) and *A. ebracteatus* (Ae) that formed *A. tetraploideus* (At). To elaborate the sequence of these two events, the distributions of synonymous divergence (Ks) values from the comparisons of orthologous (interspecific) or paralogous (intraspecific) gene pairs from syntenic blocks were plotted (Fig. 3A). The results showed one Ks peak at approximately 0.3 in each of Ai-Ai and Ae-Ae comparisons, confirming that these two species inherited a common WGD signature. In contrast, the At-At comparison showed two peaks, one at ~0.3 (WGD peak) and another one at ~0.04 (merger peak). These two peaks were also observed in the Ai-Ae comparison. In the At-At comparison, the signal of the WGD peak (shown by peak height) was weaker than that of the merger peak, and vice versa in the Ai-Ae comparison. These two peaks were also observed in the Ai-Ae comparison. These Ks peak patterns together supported that WGD event (the older peak) occurred before the polyploidization event (the younger peak).

Additional Ks distributions were obtained from the comparisons of *A. ilicifolius* genes (as a representative of *A. tetraploideus* progenitors) with those of *Andrographis paniculata* (Ap), *Avicennia Marina* (Am), and *Aegiceras corniculatum* (Ac). *A. paniculata* and *A. marina* were used as representatives of terrestrial and mangrove species in the family Acanthaceae, while *A. corniculatum* was a distantly diverged mangrove species within the Asterids clade. The results showed that the Ks value representing *Acanthus* WGD event (~0.3) was lower than the values at peaks from Ai-Am (~0.7), Ai-Ap (~1.0), and Ai-Ac (~1.8) comparisons. These patterns indicated that the WGD event occurred after *Acanthus* species diverged from *A. marina* and *A. paniculata*.

The Ks distribution was also analyzed between the subgenomes of *A. tetraploideus* (Fig. 3B). Similar to the Ai–Ae comparison, both merger and WGD peaks were detected in the SG1–SG2 comparison. Additionally, a small peak with very low Ks values (< ~0.01) was observed in both SG1–SG1 and SG2–SG2 comparisons (blue pointer in Fig. 3B), likely reflecting recent sequence changes within each *A. tetraploideus* subgenome following hybridization. Interestingly, a similar peak with a slightly higher Ks value (~0.04) was observed in the Ae–Ae comparison but was absent in the Ai–Ai comparison (black pointer in Fig. 3A), indicating that sequence alterations occurred more recently and extensively in the *A. ebracteatus* genome than in the *A. ilicifolius* genome.

4. The expansion of chromosome sequence

To further examine sequence modifications in the genome, the ratio of genes retained from each progenitor across the chromosomes of *A. tetraploideus* was analyzed (Fig. 3C). A high gene retention ratio across broad chromosomal regions indicated that SG1 and SG2 chromosomes preserved nearly complete sets of genes from their respective progenitor chromosomes, with minimal sequence exchange after hybridization. Notably, evidence of sequence exchange was more pronounced in SG2 than in SG1. For example, the result for chromosome 24B of *A.*

tetraploideus showed an insertion of Ae_A scaffold sequence within an Ae_B-derived chromosome (Fig. 3C). Moreover, there was a region on chromosome 20B of *A. tetraploideus* that lacked homologous gene matches from either the Ae_A scaffold or other scaffolds. Using genome sequences, annotation files, and the Liftoff program [44], gene annotations from chromosome 20B were mapped to the corresponding Ae_A scaffold. The results showed that the mapped regions on the Ae_A scaffold contained repeat elements. As a result, these sequences were not annotated by the annotation pipeline, which masked repeat sequences. Sequence translation revealed that many of the mapped annotations contained premature stop codons within their coding regions. These results, together with the presence of a small peak in the Ae–Ae Ks plot (Fig. 3A), suggested a greater degree of sequence modification in the *A. ebracteatus* genome compared to that of *A. ilicifolius*.

Gene retention analysis also showed that chromosome 20B was longer than the Ae_A scaffold and its homeologous chromosomes 15A, 20A, and 15B (Fig. 3C), suggesting that it acquired additional sequences after tetraploidization. The analysis revealed that 176 annotated genes in this region were not derived from either progenitor genome (Supplementary Table S15). Based on their protein sequences, 105 of these genes were assigned to three families, indicating that they were paralogs within the same families. These results suggested that the expansion of sequence in 20B chromosomes might have been driven by gene duplication. The findings further suggested that sequence modifications occurred more frequently in the SG2 subgenome compared to SG1.

5. Comparative genomics

A phylogenetic tree of *A. ilicifolius*, *A. ebracteatus*, and the two subgenomes of *A. tetraploideus*, along with ten other mangrove species and six non-mangrove species, was constructed based on the sequences of 70 single-copy genes (Fig. 4A). The results showed the clear separations of Rosids and Asterids clades, with *Nypa fruticans* mangrove (monocot) placed as the outgroup.

In this phylogenetic tree, mangrove species were predominantly found in the Rosids clade, whereas only five mangrove plants, including *A. corniculatum*, *A. marina*, *A. ilicifolius*, *A. ebracteatus*, and *A. tetraploideus*, were placed within the Asterids clade. *A. corniculatum* was the earliest diverging and the only representative of Ericales in this clade. The remaining species belonged to the order Lamiales, whose common ancestor diverged from *A. corniculatum* approximately 110 million years ago (Mya). Most species in this group, including *Andrographis paniculata* and *Strobilanthes cusia* (both in the family Acanthaceae), are terrestrial medicinal plants. Within Acanthaceae, the *Acanthus* lineage diverged from its terrestrial relatives approximately 44.42 Mya (Fig. 4A). The speciation between *A. ilicifolius* and *A. ebracteatus* was estimated to have occurred 5.75 Mya. Based on the relationship between Ks values and divergence times (Equation 1), the WGD event was estimated to have occurred 43.12 Mya (5.75 Mya × 0.3 / 0.04), approximately one million years after the divergence of the *Acanthus* lineage from its terrestrial relatives.

$$2\mu = \frac{T_s}{Ks_s} = \frac{T_d}{Ks_d}$$

Ks_s and Ks_d are values at Ks peaks representing speciation and WGD events (0.3 and 0.04). T_s and T_d are estimated times for speciation and WGD events. μ was a substitution rate.

A new tetraploid species, recently named *A. tetraploideus* [1], was later present within *Acanthus* lineage through the hybridization of *A. ilicifolius* and *A. ebracteatus*. To estimate the genome merger time, the divergence time between each subgenome and its corresponding progenitor was estimated. The results, however, yielded two distinct divergence times. The SG1 subgenome was estimated to have diverged from *A. ilicifolius* approximately 1.5 Mya, whereas SG2 diverged from *A. ebracteatus* about 0.7 Mya. This difference might be attributed to differences in the geographic origins of the samples. Depending on the primary distribution of these species, *A. ebracteatus* and *A. tetraploideus* were collected from the Gulf of Thailand,

while *A. ilicifolius* was sampled from the Andaman coast. Genetic variation associated with these distinct sampling locations might have influenced the divergence time estimates.

To support this hypothesis, SNPs of samples from two sites were compared using population structure analysis. The analysis showed two clear genetic differentiation between samples from the Gulf of Thailand and Andaman coast in all *Acanthus* species (Figure 4B). In *A. ilicifolius*, the single sample from the Gulf side was assigned to the blue cluster (the Gulf of Thailand cluster), while all individuals from Andaman provinces were consistently assigned to the orange cluster (the Andaman coast cluster) with little admixture. Similarly, in *A. ebracteatus*, individuals from Gulf provinces were uniformly assigned to the blue cluster, while only a few samples from the Andaman side exhibited partial Andaman ancestry. Nearly all *A. tetraploideus* samples were assigned to the Andaman cluster, with only one sample from Satun (STN-At-01) was fully assigned to the Gulf of Thailand cluster. These results indicated a strong east–west genetic separation.

Because of such genetic difference, the genome merger time was estimated using the divergence rates of transposable element (TE) sequences within *A. tetraploideus* genome. The TE divergence rates corresponding to the subgenome merger and divergence events were 6.3% and 27.3%, respectively (Supplementary Fig. S3). Analogous to the use of Ks rates, Equation 1 was adopted using these TE divergence values and the estimated progenitor divergence time of 5.75 Mya as inputs. As a result, the genome merger event was estimated to have occurred approximately 1.33 Mya (5.75 Mya × 6.3/27.3).

6. Protein family analysis

To enhance our understanding of the evolution of *Acanthus* species, protein family expansion and contraction were analyzed. Protein families were defined as expanded when they contained more protein members in a given species (leaf node) or ancestor (internal node) than in their

most recent common ancestor (MRCA) and contracted when the reverse pattern was observed. Using this framework, numerous cases of gene family expansion and contraction across *A. marina*, *A. paniculata*, *S. cusia*, *A. ebracteatus*, *A. ilicifolius*, and the two subgenomes of *A. tetraploideus* (SG1 and SG2) were identified (Supplementary Table S16).

Several families were expanded in the MRCA of *Acanthus* species and contracted in the MRCA of *A. paniculata* and *S. cusia*. Many of these families were associated with root development. For example, families OG0001555 and OG0000052 were exclusively expanded within *Acanthus*. OG0001555 contained NFD6/NOXY2-like proteins, which were associated with lateral root development, while OG0000052 included MYB36 transcription factors, essential for Casparian strip formation. MYB36 was linked (via STRING co-occurrence) to Protein MIZU-KUSSEI 1 (Ai1gPKTg3706.t1) in OG0006410, which was associated with hydrotropism GO term (GO:001027), suggesting a potential functional network involved in water-responsive root development. An additional family that could participate in Casparian strip formation was family OG0000343, which contained dirigent proteins. The protein counts in this family were higher in *Acanthus* species and Am than in Ap and Sc. In contrast, another root-related family (OG0001760), containing DEEPER ROOTING 1, showed reduced representation in all *Acanthus* species compared to other members of the Acanthaceae clade. The contraction of this family might reflect an evolutionary shift in *Acanthus* toward shallower root architectures, potentially as an adaptation to anoxic, waterlogged soils and surface-level substrate anchorage typical of intertidal environments.

Some families were expanded in the MRCA of *A. paniculata* and *S. cusia* but contracted in the broader Acanthaceae MRCAs. Many of these were involved in terpenoid biosynthesis. Their expansion seemed mainly driven by increased protein numbers in *S. cusia*, while other species maintained relatively stable counts. Some families also showed high variability across species. For instance, OG0000029 (germacrene synthases) exhibited two- to three-fold higher copy

numbers in *A. paniculata* and *S. cusia* than in *A. marina*, *A. ilicifolius*, *A. ebracteatus*, SG1, and SG2. Family OG0000080 (beta-amyrin monooxygenases) displayed a gradual contraction across the phylogeny, with the fewest copies observed in both SG1 and SG2 of *A. tetraploideus*. These shifts implied that *Acanthus* species might have reduced their reliance on specific triterpenoid pathways relative to other Acanthaceae.

Several protein families exhibited opposing patterns of expansion and contraction between the Ai–SG1 and Ae–SG2 lineages. For instance, families OG0000012 and OG0000227, encoding probable xyloglucan endotransglucosylase/hydrolase (XTH) and putative pectinesterase/pectinesterase inhibitors (PMEI), were expanded in the MRCA of *A. ilicifolius* and SG1 but contracted in *A. ebracteatus* and SG2. A similar trend was observed for families OG0010398 and OG0000848, both containing chalcone synthase in the flavonoid biosynthesis pathway, which were expanded in *A. ilicifolius* but contracted in *A. ebracteatus*. Likewise, the family of endodermis-specific peroxidase 64 (OG0002958) was expanded in SG1 but contracted in Ae and SG2. Family OG0000840, encoding mechanosensitive ion channel proteins, was also contracted in *A. ebracteatus*. Conversely, family OG0013347, comprising aquaporin PIP2-7 members, underwent significant expansion in the MRCA of *A. ebracteatus* and SG2, with the highest number of copies observed in *A. ebracteatus*. Family OG0000188 of WIP-type zinc finger proteins was expanded in Ae, SG2, and Am, but had lower copy numbers in Ap, Sc, Ai, and SG1. These contrasting patterns highlighted divergent evolutionary pressures acting on the two parental lineages and their respective contributions to the *A. tetraploideus* genome.

To complement the phylogenetic expansion and contraction analysis, we applied machine learning to identify protein families with markedly different member counts in *Acanthus* compared to other taxa. Several families exhibited Acanthus-specific abundance patterns. Examples were OG0003440 (PHD finger protein Alfin1), OG0004372 and OG0012170 (A20/AN1 stress-associated proteins), and OG0009241 and OG0017721 (homeobox-leucine

zipper protein ANTHOCYANINLESS 2), associating with salt, osmotic, or water deprivation responses. They appeared specific to the Acanthaceae clade (except OG0003440) and were more abundant in *Acanthus* species than in all others studied. Family OG0000866 (Rho GDP-dissociation inhibitor 1), involved in root epidermal cell differentiation, was found in all species but was most abundant in *Acanthus*. Finally, families OG0006911 and OG0014066, containing ferruginol synthases, were uniquely present in *Acanthus*. Ferruginol is a diterpene phenol with antibacterial, antitumor, and antimalarial activities [45].

Table 1 Comparative analysis of gene family expansion and contraction in *Acanthus* and related Acanthaceae species

Functional Group	Family ID	Representative Protein	Pattern in Acanthus	Pattern in Other Species
Root Development & Casparian Strip	OG0001555	NFD6/NOXY2-like	Expanded in Acanthus MRCA	Contracted in Ap/Sc
	OG0000052	MYB36	Expanded in Acanthus MRCA	Contracted in Ap/Sc
	OG0000343	Dirigent protein	Higher in Acanthus and Am	Lower in Ap/Sc
	OG0002958	Peroxidase 64	Expanded in SG1	Contracted in Ae/SG2
	OG0001760	Deeper Rooting 1	Lower in Acanthus	Higher in other Acanthaceae
	OG0009241	Homeobox-leucine zipper ANL2	Higher in Acanthus	Lower in others
	OG0017721	Homeobox-leucine zipper ANL2	Higher in Acanthus	Lower in others
	OG0000866	Rho GDP-dissociation inhibitor 1	Highest in Acanthus	Present in all
Osmotic Stress & Water Transport	OG0000188	WIP-type Zinc finger	Expanded in Ae/SG2/Am	Lower in Ai/SG1/Ap/Sc
	OG0013347	Aquaporin PIP2-7	Expanded in Ae/SG2	Not expanded in others
	OG0000840	Mechanosensitive ion channel	Contracted in Ae	Present in others
	OG0004372	A20/AN1 stress protein	Higher in Acanthus	Lower in others
	OG0012170	A20/AN1 stress protein	Higher in Acanthus	Lower in others
Cell Wall Remodeling	OG0000012	Xyloglucan endotransglucosylase	Expanded in Ai/SG1	Contracted in Ae/SG2

	OG0000227	Pectinesterase inhibitor	Expanded in Ai/SG1	Contracted in Ae/SG2
Secondary Metabolism (Flavonoids & Terpenoids)	OG0010398	Chalcone synthase	Expanded in Ai	Contracted in Ae
	OG0000848	Chalcone synthase	Expanded in Ai	Contracted in Ae
	OG0000029	Germacrene synthase	Fluctuated	Higher in Ap/Sc
	OG0000080	Beta-amyrin monooxygenase	Contracted in Acanthus	Higher in Am
	OG0006911	Ferruginol synthase	Unique to Acanthus	Absent in others
	OG0014066	Ferruginol synthase	Unique to Acanthus	Absent in others
Stress/Signaling Regulators	OG0003440	PHD finger Alfin1	Higher in Acanthus	Lower in others
Unknown / Hypothetical	OG0000061	Hypothetical protein	Very high in Acanthus	Low in others

Discussion

Allotetraploid origin of *Acanthus tetraploideus*

In this study, we generated a Hi-C-based genome assembly of the allotetraploid *A. tetraploideus* (~1.92 Gb, 48 chromosomes) and stLFR-based assemblies of its diploid progenitors, *A. ilicifolius* (~0.98 Gb) and *A. ebracteatus* (~0.89 Gb). The genome of *A. tetraploideus* comprised two subgenomes, SG1 (~1.03 Gb) and SG2 (~0.89 Gb), which corresponded in size and synteny to the genomes of *A. ilicifolius* and *A. ebracteatus*, respectively. Additionally, *A. tetraploideus* contained a high proportion of duplicated genes (~95.5%). These results together

clearly revealed that *A. tetraploideus* inherited SG1 from *A. ilicifolius* and SG2 from *A. ebracteatus*, which supported previous chloroplast and nuclear gene analyses [1].

Whole-genome duplication and lineage-specific expansion of root-related protein families in *Acanthus*

Comparative synteny and synonymous substitution analyses provided evidence for a WGD event in the *Acanthus* lineage prior to the divergence of *A. ilicifolius* and *A. ebracteatus*. This event was estimated to have taken place approximately 43 Mya, shortly after the separation of the *Acanthus* lineage from *A. paniculata* and *S. cusia* around 44 Mya. A separate WGD in *A. marina* was also estimated to have occurred within the same period (43.08–50 Mya), approximately 10 million years after its divergence from other Acanthaceae species [34]. There was no comparable genome duplication reported in *A. paniculata* [46] and *S. cusia* [35], indicating these WGD events were mangrove specific. These WGD events occurred during the early to middle Eocene (~50–40 Mya), when expanded shallow coastal habitats and dynamic conditions, such as salinity fluctuations, tidal inundation, and anoxic soils, appeared to drive the adaptation and diversification of mangrove genera [47]. Such climatic instability during the Eocene might have driven WGD in Acanthaceae mangroves. This is consistent with findings in Malpighiales, which showed that ancient WGDs were largely associated with major climatic upheavals [48]. WGD likely conferred a selective advantage under environmental stress by increasing genetic redundancy [48,49]. In *Acanthus*, this duplication might have enabled long-term persistence and diversification by allowing adaptation to newly formed ecological niches under rising sea levels and warming climates.

Gene family expansions and contractions appeared linked to selective pressures acting on *Acanthus* mangroves. Notably, several expanded families were involved in Casparian strip formation and root development. For Casparian strips, lineage-specific expansions were observed in MYB36 transcription factor, Dirigent protein 10-like proteins (DIR10-like or

ENHANCED SUBERIN 1 homologs), and peroxidases 64 (PER64). MYB36 promoted Casparian strip assembly by directly upregulating PER64 and DIR10-like encoding genes, which were responsible for localized polymerization of lignin [50,51]. These lignin/suberin bands blocked unselective salt flow and were vital for salt exclusion and structural support in saline, wave-exposed habitats [52]. In root development, the NFD6/NOXY2-like family was specifically expanded in *Acanthus*. *NOXY2* enhances root waving and contributes to defense, despite limiting lateral root formation [53]. In *Arabidopsis*, root waving occurred on an inclined and impenetrable medium [54,55]. Expansion of this protein family might promote the adaptation of *Acanthus* to sloped and sediment-rich mangrove shorelines that have been shaped by sea-level changes. Correspondingly, machine learning also identified elevated copy numbers of ANL2, RhoGDI1, Alfin1, and SAP families, which were linked to root development and salt stress responses. *RhoGDI1* and *Alfin1*, for example, regulated root growth under saline conditions [56,57]. In contrast, the Deeper Rooting 1 (DRO1) family was contracted in *Acanthus*. The introduction of *DRO1* gene resulted in deep-rooting phenotype in shallow-rooting rice plants in response to drought stress [58]. The observed expansion and contraction patterns of these families suggested that *Acanthus* adapted to produce shallow tap-root systems with lateral spread in the topsoil.

Allopatric divergence and differential gene family evolution in *A. ilicifolius* and *A. ebracteatus*

The speciation of *A. ilicifolius* and *A. ebracteatus* (~5.7 Mya) also occurred during a period of marked climatic instability in the Late Miocene. During this period, the South China Sea experienced reduced atmospheric CO_2 levels, intensified winter monsoons, and increased climatic seasonality [59], all of which could have influenced plant evolutionary trajectories [60]. The modeling of tidal patterns suggested that tidal amplification and sediment dynamics shaped intertidal environments during the Oligo–Miocene [61]. Notably, the modeled tidal range during

the Messinian (~6 Ma) aligned with the current biogeographic separation of *A. ilicifolius* and *A. ebracteatus* [1,61], suggesting that their speciation was likely associated with adaptation to ecologically distinct habitats. *A. ilicifolius* typically inhabits open, seaward-facing western coasts with higher salinity and prolonged tidal exposure, whereas *A. ebracteatus* is found along eastern coasts in upper intertidal or estuarine zones with greater freshwater influence. Additionally, we observed genetic differentiation between *A. ilicifolius* and *A. ebracteatus* populations across the Gulf of Thailand and the Andaman coast, highlighting the role of geographic and oceanographic barriers in shaping their genetic. Overall, the combination of intensified monsoons and shoreline reorganization likely fragmented and reconnected mangrove habitats across Southeast Asia, facilitating dispersal and subsequent allopatric divergence of *Acanthus* species.

Following divergence, several protein families showed contrasting patterns of expansion and contraction between the Ai–SG1 and Ae–SG2 lineages, reflecting independent genome evolution. More gene families were expanded in Ai–SG1 and contracted in Ae–SG2 than vice versa, with several of these related to root development. One such family was PER64, essential for Casparian strip formation and cell wall rigidification, critical for regulating water and ion uptake under salt stress [62]. Another family contained xyloglucan endotransglucosylase/hydrolase 23 (XTH23), which was known to contribute to lateral root adaptation to salt stress [63]. Moreover, chalcone synthase (CHS) was also expanded in Ai–SG1. CHS catalyzes the first step in flavonoid biosynthesis, and its expansion aligned with the richness in flavonoids and phenolics in *A. ilicifolius* [3]. *CHS* contributed to salt tolerance in transgenic tobacco, and its expression was triggered by environmental stimuli such as UV light, pathogens, and salinity [64]. The expansion of those families might have contributed to the adaptation of *A. ilicifolius* to open, high-salinity coastal environments, in contrast to *A. ebracteatus*, which thrived in a more sheltered, freshwater-influenced habitat.

Hybrid origin and evolutionary advantage of *A. tetraploideus*

A more recent evolutionary event in the *Acanthus* lineage was the hybridization of *A. ilicifolius* and *A. ebracteatus*, which resulted in the formation of the allotetraploid species *A. tetraploideus*. This event occurred ~1.3–1.5 Mya during the early Pleistocene, a period marked by glacial–interglacial cycles and sea-level fluctuations of up to ~100 meters [65]. These cycles alternately isolated and reconnected mangrove habitats through lowered glacial sea levels and higher interglacial seas, creating opportunities for secondary contact and hybridization among mangrove species [66,67]. Such dynamics likely facilitated secondary contact between *A. ilicifolius* and *A. ebracteatus*, leading to the emergence of the allotetraploid *A. tetraploideus*.

Once formed, *A. tetraploideus* became reproductively isolated from its diploid parents. It possessed the ability to produce viable seeds and even propagate clonally [1], making it exceptional among many mangrove genera such as *Rhizophora*, *Bruguiera*, *Sonneratia*, *Ceriops*, and *Avicennia*, in which hybridization typically resulted in sterile F1 hybrids and failed to establish stable lineages [68]. *A. tetraploideus* was a young allotetraploid and its two subgenomes remained largely intact and maintained collinearity with their progenitor genomes (Fig. 1). The absence of extensive homeologous recombination might have facilitated disomic pairing during meiosis, allowing each chromosome to align with its true homolog and resulting in the formation of balanced gametes [69].

The two subgenomes of *A. tetraploideus* showed slight asymmetry in gene retention. Consistently, we observed no substantial protein family expansion or contraction in either subgenome compared to the progenitor genomes. Transcriptomic analysis revealed modest expression bias toward the SG1 subgenome and both subgenomes remained functionally active, with expression reprogramming, and contributed to overall fitness [4]. Overall, the genome merger and transcriptomic reprogramming appeared to have enhanced the fitness of *A. tetraploideus*, potentially contributing to its proliferation in the Gulf of Thailand, where *A. ebracteatus* has existed since approximately 5.7 Mya. The genome of *A. ebracteatus* was

smaller and exhibited greater structural changes compared to *A. ilicifolius*, suggesting higher genomic instability in the former. In *Cirsium*, for example, species with smaller genomes produce interspecific hybrids more frequently than those with larger genome [70]. It is plausible that the genomic dynamics of *A. ebracteatus* made it more susceptible to hybridization, enabling the establishment and expansion of *A. tetraploideus* within its habitat.

Abbreviations

BUSCO: Benchmarking Universal Single-Copy Orthologs, GO: Gene Ontology, Hi-C: High-throughput Chromosome Conformation Capture, Ks: Synonymous Substitution Rate, LTR: Long Terminal Repeat, MRCA: Most Recent Common Ancestor, Mya: Million Years Ago, N50: Contig or Scaffold Length at 50% of Genome Assembly, OG: Orthogroup, stLFR: Single-Tube Long Fragment Read, TE: Transposable Element, WGD: Whole-Genome Duplication.

Data Availability Statement

The assembled genome sequences of *A. tetraploideus*, *A. ilicifolius*, and *A. ebracteatus* were deposited in the NCBI database under BioProject PRJNA1102049, PRJNA1275650, and PRJNA1111239, respectively.

Acknowledgments

We thank the research team from the Mangrove Forest Research Center for their sample collection. The authors would also like to acknowledge funding from the National Science and Technology Development Agency, Thailand (project ID: P2351523).

Competing interests

The authors declare that they have no competing interests.

References

1. Feng H, Banerjee AK, Guo W, Yuan Y, Duan F, Ng WL, et al.. Origin and evolution of a new tetraploid mangrove species in an intertidal zone. *Plant Diversity*. 2024; doi: 10.1016/j.pld.2024.04.007.

2. Yang Y, Yang S, Li J, Deng Y, Zhang Z, Xu S, et al.. Transcriptome analysis of the Holly mangrove Acanthus ilicifolius and its terrestrial relative, Acanthus leucostachyus, provides insights into adaptation to intertidal zones. *BMC Genomics*. 2015; doi: 10.1186/s12864-015-1813-9.

3. Matos P, Batista MT, Figueirinha A. A review of the ethnomedicinal uses, chemistry, and pharmacological properties of the genus Acanthus (Acanthaceae). *Journal of Ethnopharmacology*. 2022; doi: 10.1016/j.jep.2022.115271.

4. Guo W, Banerjee AK, Feng H, Ng WL, Wu H, Li W, et al.. Recent allopolyploidization and transcriptomic asymmetry in the mangrove shrub Acanthus tetraploideus. *BMC Genomics*. 2025; doi: 10.1186/s12864-025-11557-2.

5. Kong W, Wang Y, Zhang S, Yu J, Zhang X. Recent Advances in Assembly of Complex Plant Genomes. *Genomics, Proteomics & Bioinformatics*. 2023; doi: 10.1016/j.gpb.2023.04.004.

6. Natarajan P, Murugesan AK, Govindan G, Gopalakrishnan A, Kumar R, Duraisamy P, et al.. A reference-grade genome identifies salt-tolerance genes from the salt-secreting mangrove species Avicennia marina. *Commun Biol*. Nature Publishing Group; 2021; doi: 10.1038/s42003-021-02384-8.

7. Pootakham W, Naktang C, Sonthirod C, Kongkachana W, Narong N, Sangsrakru D, et al.. Chromosome-level genome assembly of Indian mangrove (Ceriops tagal) revealed a genome-wide duplication event predating the divergence of Rhizophoraceae mangrove species. *The Plant Genome*. 2022; doi: 10.1002/tpg2.20217.

8. Shearman JR, Naktang C, Sonthirod C, Kongkachana W, U-thoomporn S, Jomchai N, et al.. Assembly of a hybrid mangrove, *Bruguiera hainesii*, and its two ancestral contributors, *Bruguiera cylindrica* and *Bruguiera gymnorhiza*. *Genomics*. 2022; doi: 10.1016/j.ygeno.2022.110382.

9. Pootakham W, Nawae W, Naktang C, Sonthirod C, Yoocha T, Kongkachana W, et al.. A chromosome-scale assembly of the black gram (Vigna mungo) genome. *Molecular Ecology Resources*. 2021; doi: 10.1111/1755-0998.13243.

10. Pootakham W, Naktang C, Sonthirod C, Kongkachana W, Yoocha T, Jomchai N, et al.. De Novo Reference Assembly of the Upriver Orange Mangrove (Bruguiera sexangula) Genome. *Genome Biology and Evolution*. 2022; doi: 10.1093/gbe/evac025.

11. Cheng H, Concepcion GT, Feng X, Zhang H, Li H. Haplotype-resolved de novo assembly using phased assembly graphs with hifiasm. *Nat Methods*. Nature Publishing Group; 2021; doi: 10.1038/s41592-020-01056-5.

12. Zhou C, McCarthy SA, Durbin R. YaHS: yet another Hi-C scaffolding tool. *Bioinformatics*. 2023; doi: 10.1093/bioinformatics/btac808.

13. Manni M, Berkeley MR, Seppey M, Zdobnov EM. BUSCO: Assessing Genomic Data Quality and Beyond. *Current Protocols*. 2021; doi: 10.1002/cpz1.323.

14. Cabanettes F, Klopp C. D-GENIES: dot plot large genomes in an interactive, efficient and simple way. *PeerJ*. PeerJ Inc.; 2018; doi: 10.7717/peerj.4958.

15. Alonge M, Lebeigle L, Kirsche M, Jenike K, Ou S, Aganezov S, et al.. Automated assembly scaffolding using RagTag elevates a new tomato system for high-throughput genome editing. *Genome Biology*. 2022; doi: 10.1186/s13059-022-02823-7.

16. Brůna T, Hoff KJ, Lomsadze A, Stanke M, Borodovsky M. BRAKER2: automatic eukaryotic genome annotation with GeneMark-EP+ and AUGUSTUS supported by a protein database. *NAR Genomics and Bioinformatics*. 2021; doi: 10.1093/nargab/lqaa108.

17. Kuznetsov D, Tegenfeldt F, Manni M, Seppey M, Berkeley M, Kriventseva EV, et al.. OrthoDB v11: annotation of orthologs in the widest sampling of organismal diversity. *Nucleic Acids Research*. 2023; doi: 10.1093/nar/gkac998.

18. Gurevich A, Saveliev V, Vyahhi N, Tesler G. QUAST: quality assessment tool for genome assemblies. *Bioinformatics*. 2013; doi: 10.1093/bioinformatics/btt086.

19. Jia K-H, Wang Z-X, Wang L, Li G-Y, Zhang W, Wang X-L, et al.. SubPhaser: a robust allopolyploid subgenome phasing method based on subgenome-specific k-mers. *New Phytologist*. 2022; doi: 10.1111/nph.18173.

20. Wang Y, Tang H, Wang X, Sun Y, Joseph PV, Paterson AH. Detection of colinear blocks and synteny and evolutionary analyses based on utilization of MCScanX. *Nat Protoc*. Nature Publishing Group; 2024; doi: 10.1038/s41596-024-00968-2.

21. Tang H, Krishnakumar V, Zeng X, Xu Z, Taranto A, Lomas JS, et al.. JCVI: A versatile toolkit for comparative genomics analysis. *iMeta*. 2024; doi: 10.1002/imt2.211.

22. Krzywinski M, Schein J, Birol İ, Connors J, Gascoyne R, Horsman D, et al.. Circos: An information aesthetic for comparative genomics. *Genome Res*. 2009; doi: 10.1101/gr.092759.109.

23. Edgar RC. MUSCLE: a multiple sequence alignment method with reduced time and space complexity. *BMC Bioinformatics*. 2004; doi: 10.1186/1471-2105-5-113.

24. Yang Z. PAML 4: Phylogenetic Analysis by Maximum Likelihood. *Mol Biol Evol*. Oxford Academic; 2007; doi: 10.1093/molbev/msm088.

25. Sun P, Jiao B, Yang Y, Shan L, Li T, Li X, et al.. WGDI: A user-friendly toolkit for evolutionary analyses of whole-genome duplications and ancestral karyotypes. *Molecular Plant*. 2022; doi: 10.1016/j.molp.2022.10.018.

26. Wu W, Feng X, Wang N, Shao S, Liu M, Si F, et al.. Genomic analysis of Nypa fruticans elucidates its intertidal adaptations and early palm evolution. *Journal of Integrative Plant Biology*. 2024; doi: 10.1111/jipb.13625.

27. Xie W, Guo Z, Wang J, He Z, Li Y, Feng X, et al.. Evolution of woody plants to the land-sea interface – The atypical genomic features of mangroves with atypical phenotypic adaptation. *Molecular Ecology*. 2023; doi: 10.1111/mec.16587.

28. Feng X, Chen Q, Wu W, Wang J, Li G, Xu S, et al.. Genomic evidence for rediploidization and adaptive evolution following the whole-genome triplication. *Nat Commun*. Nature Publishing Group; 2024; doi: 10.1038/s41467-024-46080-7.

29. Pootakham W, Sonthirod C, Naktang C, Kongkachana W, Sangsrakru D, U-thoomporn S, et al.. A chromosome-scale reference genome assembly of yellow mangrove (Bruguiera parviflora) reveals a whole genome duplication event associated with the Rhizophoraceae lineage. *Molecular Ecology Resources*. 2022; doi: 10.1111/1755-0998.13587.

30. Ruang-areerate P, Naktang C, Kongkachana W, Sangsrakru D, Narong N, Maknual C, et al.. Assessment of the Genetic Diversity and Population Structure of Rhizophora apiculata Blume (Rhizophoraceae) in Thailand. *Biology*. Multidisciplinary Digital Publishing Institute; 2022; doi: 10.3390/biology11101449.

31. Hu M-J, Sun W-H, Tsai W-C, Xiang S, Lai X-K, Chen D-Q, et al.. Chromosome-scale assembly of the Kandelia obovata genome. *Hortic Res*. Nature Publishing Group; 2020; doi: 10.1038/s41438-020-0300-x.

32. Ma D, Guo Z, Ding Q, Zhao Z, Shen Z, Wei M, et al.. Chromosome-level assembly of the mangrove plant Aegiceras corniculatum genome generated through Illumina, PacBio and Hi-C sequencing technologies. *Molecular Ecology Resources*. 2021; doi: 10.1111/1755-0998.13347.

33. Ma L, Dong C, Song C, Wang X, Zheng X, Niu Y, et al.. De novo genome assembly of the potent medicinal plant Rehmannia glutinosa using nanopore technology. *Computational and Structural Biotechnology Journal*. Elsevier; 2021; doi: 10.1016/j.csbj.2021.07.006.

34. Ma D, Ding Q, Guo Z, Xu C, Liang P, Zhao Z, et al.. The genome of a mangrove plant, Avicennia marina, provides insights into adaptation to coastal intertidal habitats. *Planta*. 2022; doi: 10.1007/s00425-022-03916-0.

35. Hu Y, Ma D, Ning S, Ye Q, Zhao X, Ding Q, et al.. High-Quality Genome of the Medicinal Plant Strobilanthes cusia Provides Insights Into the Biosynthesis of Indole Alkaloids. *Front Plant Sci*. Frontiers; 2021; doi: 10.3389/fpls.2021.742420.

36. Liang Y, Chen S, Wei K, Yang Z, Duan S, Du Y, et al.. Chromosome Level Genome Assembly of Andrographis paniculata. *Front Genet*. Frontiers; 2020; doi: 10.3389/fgene.2020.00701.

37. Emms DM, Kelly S. OrthoFinder: phylogenetic orthology inference for comparative genomics. *Genome Biology*. 2019; doi: 10.1186/s13059-019-1832-y.

38. Capella-Gutiérrez S, Silla-Martínez JM, Gabaldón T. trimAl: a tool for automated alignment trimming in large-scale phylogenetic analyses. *Bioinformatics*. 2009; doi: 10.1093/bioinformatics/btp348.

39. Kozlov AM, Darriba D, Flouri T, Morel B, Stamatakis A. RAxML-NG: a fast, scalable and user-friendly tool for maximum likelihood phylogenetic inference. *Bioinformatics*. 2019; doi: 10.1093/bioinformatics/btz305.

40. Darriba D, Posada D, Kozlov AM, Stamatakis A, Morel B, Flouri T. ModelTest-NG: A New and Scalable Tool for the Selection of DNA and Protein Evolutionary Models. *Molecular Biology and Evolution*. 2020; doi: 10.1093/molbev/msz189.

41. Wang Y, Li Y, Wu W, Shao S, Fang Q, Xu S, et al.. The evolution history of an allotetraploid mangrove tree analysed with a new tool Allo4D. *Plant Biotechnology Journal*. 2024; doi: 10.1111/pbi.14281.

42. Mendes FK, Vanderpool D, Fulton B, Hahn MW. CAFE 5 models variation in evolutionary rates among gene families. *Bioinformatics*. 2020; doi: 10.1093/bioinformatics/btaa1022.

43. Revell LJ. phytools 2.0: an updated R ecosystem for phylogenetic comparative methods (and other things). *PeerJ*. PeerJ Inc.; 2024; doi: 10.7717/peerj.16505.

44. Shumate A, Salzberg SL. Liftoff: accurate mapping of gene annotations. *Bioinformatics*. 2021; doi: 10.1093/bioinformatics/btaa1016.

45. González-Cardenete MA, Rivas F, Basset R, Stadler M, Hering S, Padrón JM, et al.. Biological Profiling of Semisynthetic C19-Functionalized Ferruginol and Sugiol Analogues. *Antibiotics*. Multidisciplinary Digital Publishing Institute; 2021; doi: 10.3390/antibiotics10020184.

46. Sun W, Leng L, Yin Q, Xu M, Huang M, Xu Z, et al.. The genome of the medicinal plant Andrographis paniculata provides insight into the biosynthesis of the bioactive diterpenoid neoandrographolide. *The Plant Journal*. 2019; doi: 10.1111/tpj.14162.

47. Srivastava J, Prasad V. Evolution and paleobiogeography of mangroves. *Marine Ecology*. 2019; doi: 10.1111/maec.12571.

48. Cai L, Xi Z, Amorim AM, Sugumaran M, Rest JS, Liu L, et al.. Widespread ancient whole-genome duplications in Malpighiales coincide with Eocene global climatic upheaval. *New Phytologist*. 2019; doi: 10.1111/nph.15357.

49. Van de Peer Y, Mizrachi E, Marchal K. The evolutionary significance of polyploidy. *Nat Rev Genet*. Nature Publishing Group; 2017; doi: 10.1038/nrg.2017.26.

50. Kamiya T, Borghi M, Wang P, Danku JMC, Kalmbach L, Hosmani PS, et al.. The MYB36 transcription factor orchestrates Casparian strip formation. *Proceedings of the National Academy of Sciences*. Proceedings of the National Academy of Sciences; 2015; doi: 10.1073/pnas.1507691112.

51. Gao Y-Q, Huang J-Q, Reyt G, Song T, Love A, Tiemessen D, et al.. A dirigent protein complex directs lignin polymerization and assembly of the root diffusion barrier. *Science*. American Association for the Advancement of Science; 2023; doi: 10.1126/science.adi5032.

52. Chen T, Cai ,Xia, Wu ,Xiaoqin, Karahara ,Ichirou, Schreiber ,Lucas, and Lin J. Casparian strip development and its potential function in salt tolerance. *Plant Signaling & Behavior*. Taylor & Francis; 2011; doi: 10.4161/psb.6.10.17054.

53. Vellosillo T, Martínez M, López MA, Vicente J, Cascón T, Dolan L, et al.. Oxylipins Produced by the 9-Lipoxygenase Pathway in Arabidopsis Regulate Lateral Root Development and Defense Responses through a Specific Signaling Cascade. *The Plant Cell*. 2007; doi: 10.1105/tpc.106.046052.

54. Roy R, Bassham DC. Root growth movements: Waving and skewing. *Plant Science*. 2014; doi: 10.1016/j.plantsci.2014.01.007.

55. Porat A, Tekinalp A, Bhosale Y, Gazzola M, Meroz Y. On the mechanical origins of waving, coiling and skewing in Arabidopsis thaliana roots. *Proceedings of the National Academy of Sciences*. Proceedings of the National Academy of Sciences; 2024; doi: 10.1073/pnas.2312761121.

56. Winicov I. Alfin1 transcription factor overexpression enhances plant root growth under normal and saline conditions and improves salt tolerance in alfalfa. *Planta*. 2000; doi: 10.1007/PL00008150.

57. Liu X, Yu X, Shi Y, Ma L, Fu Y, Guo Y. Phosphorylation of RhoGDI1, a Rho GDP dissociation inhibitor, regulates root hair development in Arabidopsis under salt stress. *Proceedings of the National Academy of Sciences*. Proceedings of the National Academy of Sciences; 2023; doi: 10.1073/pnas.2217957120.

58. Uga Y, Sugimoto K, Ogawa S, Rane J, Ishitani M, Hara N, et al.. Control of root system architecture by DEEPER ROOTING 1 increases rice yield under drought conditions. *Nat Genet*. Nature Publishing Group; 2013; doi: 10.1038/ng.2725.

59. Holbourn AE, Kuhnt W, Clemens SC, Kochhann KGD, Jöhnck J, Lübbers J, et al.. Late Miocene climate cooling and intensification of southeast Asian winter monsoon. *Nat Commun*. Nature Publishing Group; 2018; doi: 10.1038/s41467-018-03950-1.

60. Herbert TD, Lawrence KT, Tzanova A, Peterson LC, Caballero-Gill R, Kelly CS. Late Miocene global cooling and the rise of modern ecosystems. *Nature Geosci*. Nature Publishing Group; 2016; doi: 10.1038/ngeo2813.

61. Collins DS, Avdis A, Allison PA, Johnson HD, Hill J, Piggott MD, et al.. Tidal dynamics and mangrove carbon sequestration during the Oligo–Miocene in the South China Sea. *Nat Commun*. Nature Publishing Group; 2017; doi: 10.1038/ncomms15698.

62. Lee Y, Rubio MC, Alassimone J, Geldner N. A Mechanism for Localized Lignin Deposition in the Endodermis. *Cell*. Elsevier; 2013; doi: 10.1016/j.cell.2013.02.045.

63. Xu P, Fang S, Chen H, Cai W. The brassinosteroid-responsive xyloglucan endotransglucosylase/hydrolase 19 (XTH19) and XTH23 genes are involved in lateral root development under salt stress in Arabidopsis. *The Plant Journal*. 2020; doi: 10.1111/tpj.14905.

64. Lijuan C, Huiming G, Yi L, Hongmei C. Chalcone synthase EaCHS1 from Eupatorium adenophorum functions in salt stress tolerance in tobacco. *Plant Cell Rep*. 2015; doi: 10.1007/s00299-015-1751-7.

65. Dumitru OA, Austermann J, Polyak VJ, Fornós JJ, Asmerom Y, Ginés J, et al.. Sea-level stands from the Western Mediterranean over the past 6.5 million years. *Sci Rep*. Nature Publishing Group; 2021; doi: 10.1038/s41598-020-80025-6.

66. Li J, Yang Y, Chen Q, Fang L, He Z, Guo W, et al.. Pronounced genetic differentiation and recent secondary contact in the mangrove tree Lumnitzera racemosa revealed by population genomic analyses. *Sci Rep*. Nature Publishing Group; 2016; doi: 10.1038/srep29486.

67. Yang Y, Li J, Yang S, Li X, Fang L, Zhong C, et al.. Effects of Pleistocene sea-level fluctuations on mangrove population dynamics: a lesson from Sonneratia alba. *BMC Evolutionary Biology*. 2017; doi: 10.1186/s12862-016-0849-z.

68. Ragavan P, Zhou R, Ng WL, Rana TS, Mageswaran T, Mohan PM, et al.. Natural hybridization in mangroves – an overview. *Botanical Journal of the Linnean Society*. 2017; doi: 10.1093/botlinnean/box053.

69. Soltis PS, Soltis DE. The role of genetic and genomic attributes in the success of polyploids. *Proceedings of the National Academy of Sciences*. Proceedings of the National Academy of Sciences; 2000; doi: 10.1073/pnas.97.13.7051.

70. BUREŠ P, WANG Y-F, HOROVÁ L, SUDA J. Genome Size Variation in Central European Species of Cirsium (Compositae) and their Natural Hybrids. *Annals of Botany*. 2004; doi: 10.1093/aob/mch151.

Figure legends

Figure 1. Chromosome-scale genome assembly of *Acanthus tetraploideus*. (A) The image of *A. tetraploideus* shows lanceolate, spiny, leathery leaves and pale violet flowers. (B) Hi-C contact matrix of the *A. tetraploideus* genome assembly shows strong intrachromosomal interactions and chromosome boundaries. The matrix confirms high-quality scaffolding of the 48 chromosomes. (C) Circos plot displays syntenic matches between subgenomes SG1 (orange) and SG2 (blue) of *A. tetraploideus*. The outermost layer represents the 48 chromosomes (24 pairs). Inner tracks display gene density (blue), repeat content (yellow), and syntenic blocks (colored ribbons) between homeologous chromosomes. (D) The Sankey-style diagram illustrates syntenic relationships between SG1 (blue bars) and *A. ilicifolius* (orange bars), and between SG2 (violet bars) and *A. ebracteatus* (green bars).

Figure 2. Ancestral karyotype reconstruction and chromosome number evolution in *Acanthus tetraploideus*. (A) Dot plot shows the alignment of *A. tetraploideus* chromosomes (columns) to 11 reconstructed ancestral protochromosomes (rows). Chromosomes in subgenome SG1 are labeled in black (top) and their alignments are indicated by red and yellow lines. Chromosomes in SG2 are labeled in blue and aligned using green and blue lines.

Diagonal lines indicate homeologous chromosome pairs derived from the same protochromosome. (B) A schematic model depicts chromosome evolution in the *Acanthus* lineage. The ancestral karyotype (n = 11) experienced a WGD event ~43.1 Mya, resulting in a doubled haploid chromosome number (n = 22). Subsequent chromosomal fissions increased haploid chromosome number to n = 24 approximately 5.7 Mya. Hybridization between two diploid progenitors carrying this karyotype resulted in the formation of the allotetraploid species *A. tetraploideus* (n = 48) approximately 1.3 Mya. Mixed color bars in the final karyotype indicate chromosomal indicate translocations and rearrangements.

Figure 3. Ks distribution and gene retention. (A and B) Ks distribution plots for pairwise paralogous gene comparisons among *Acanthus* and related species. (B) Ks distribution plots for the comparisons between *A. tetraploideus* sungenomes. Shaded areas highlight Ks peaks linked to genome merger (hybridization) and WGD events in the *Acanthus* lineage. Black (A) and Blue (B) pointers indicate the Ks peaks that are the signals of genome sequence alterations in *A. ebracteatus* and *A. tetraploideus*, respectively. (C) Gene retention ratios along selected chromosomes of *A. tetraploideus*. Ratios represent the proportion of genes retained from each progenitor, ranging from 0 to 1. Red and yellow lines indicate genes retained from the two groups of WGD-derived scaffolds in *A. ilicifolius*, whereas green and blue lines represent genes inherited from *A. ebracteatus* (see matched colors in Fig. 2). Gene retention patterns across all *A. tetraploideus* chromosomes are shown in Supplementary Fig. S2.

Figure 4. Population structure and gene family evolution in Acanthus and related species.

(A) Population structure analysis of three *Acanthus* species based on SNP variation. Each

vertical bar represents genetic ancestry proportions of an individual. The blue segment denotes ancestry associated with Gulf of Thailand populations and the orange segment denotes ancestry associated with Andaman-coast populations. Individuals of *A. ebracteatus* (n = 29), *A. tetraploideus* (n = 27), and *A. ilicifolius* (n = 36) are grouped by species. Gulf of Thailand populations included samples from Chumphon (CPN), Nakhon Si Thammarat (NST), Phatthalung (PLG), Phetchaburi (PBI), Samut Songkhram (SKM), Samut Sakhon (SKN), Surat Thani (SNI), and Trat (TRT), whereas Andaman coast populations included samples from Krabi (KBI), Phang Nga (PNG), Phuket (PKT), Ranong (RNG), Satun (STN), and Trang (TRG). (B) Maximum likelihood phylogenetic tree with divergence times and gene family evolution across 19 plant species, including mangrove and non-mangrove taxa. Green and red numbers represent significantly expanded (+) and contracted (−) gene families, respectively. *Acanthus* subgenomes (SG1 and SG2) are indicated for *A. tetraploideus*. Numbers at each branch indicate the estimated divergence time. Orange dots at nodes indicate calibration time points, obtained from the TimeTree database. Blue stars mark the *Avicennia*-specific and *Acanthus*-specific WGD events.

Figure 1

Click here to access/download;Figure;Figure1.tiff

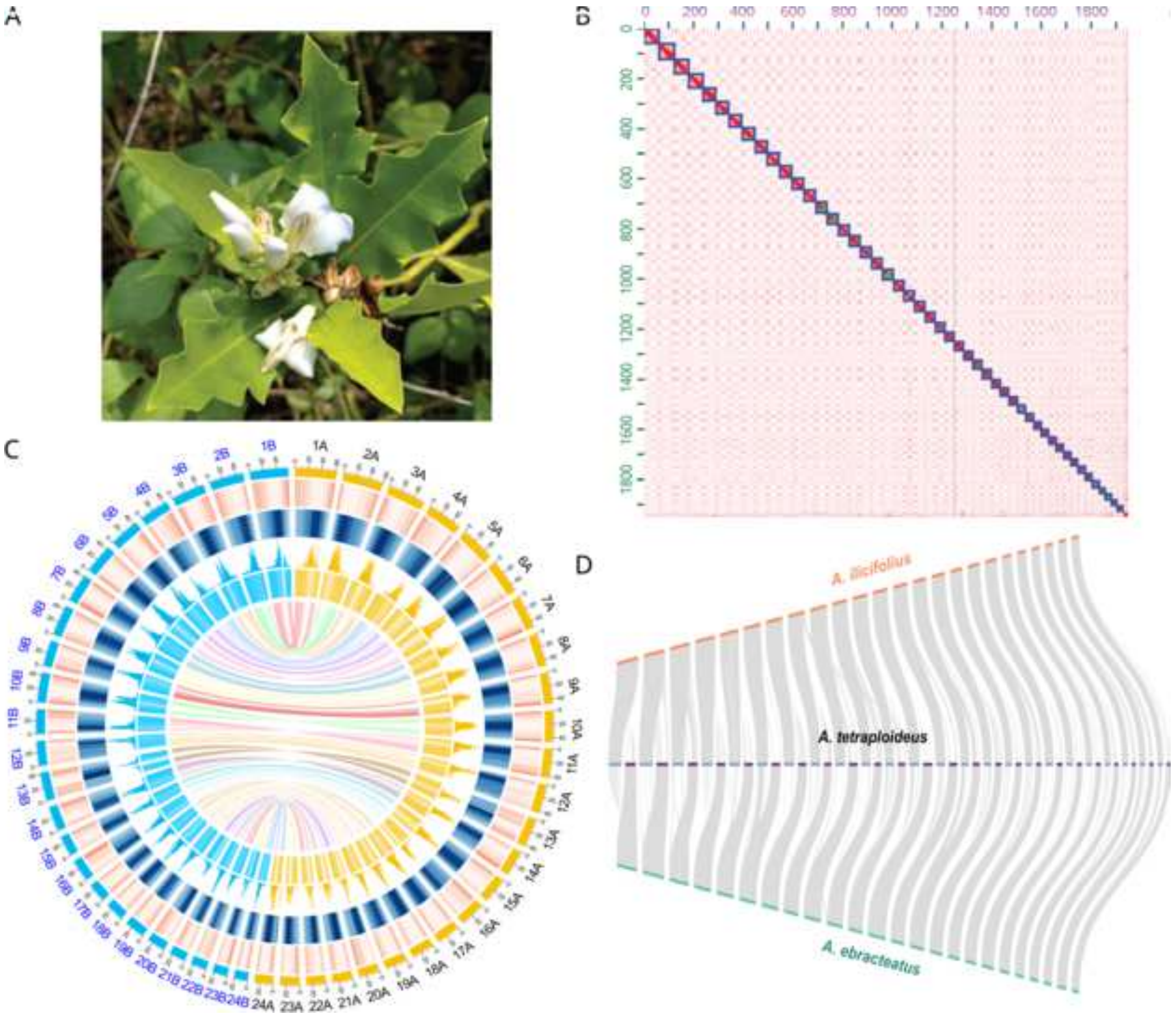

Figure 2

Click here to access/download;Figure;Figure2.tiff

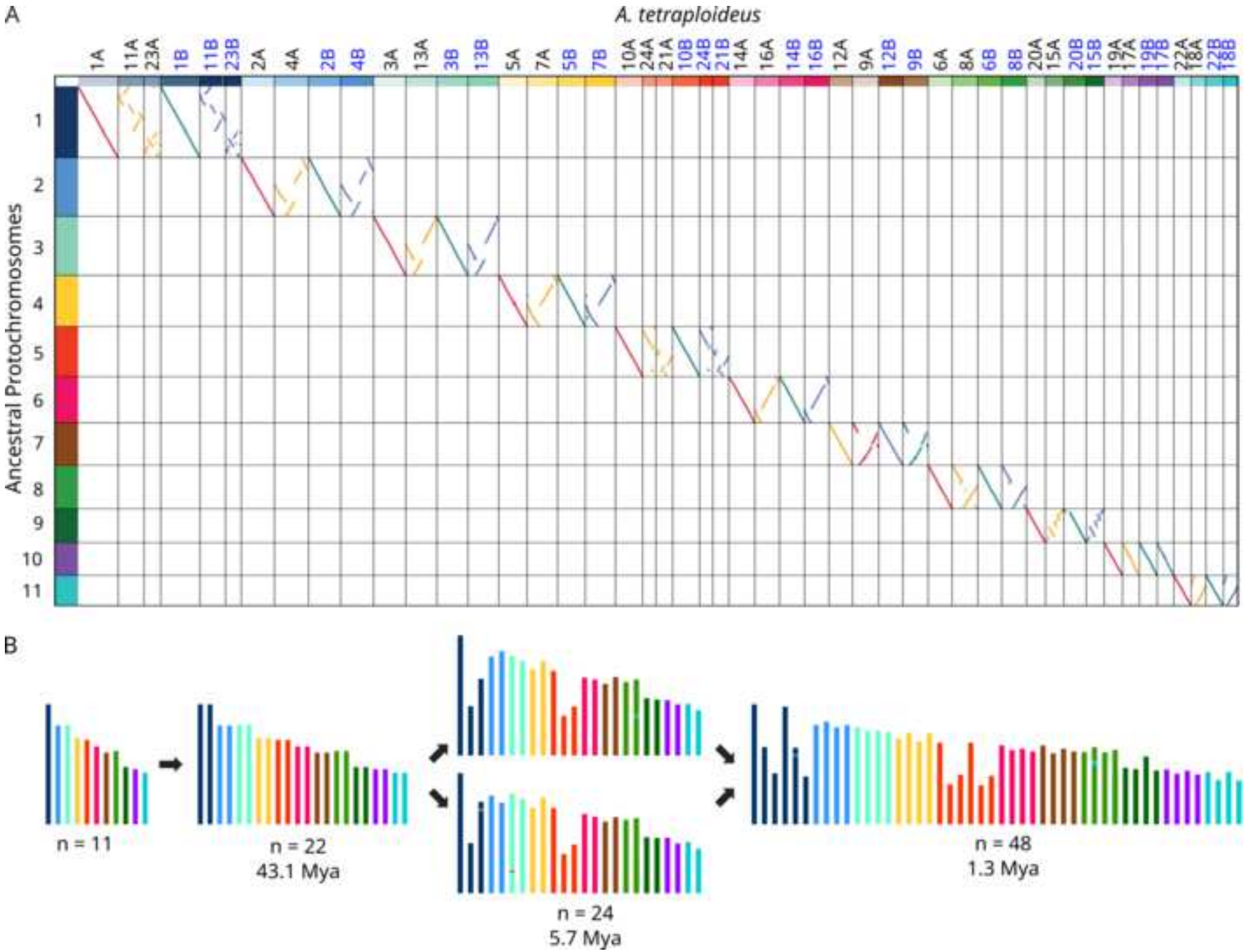

Figure 3

Click here to access/download;Figure;Figure3.tiff

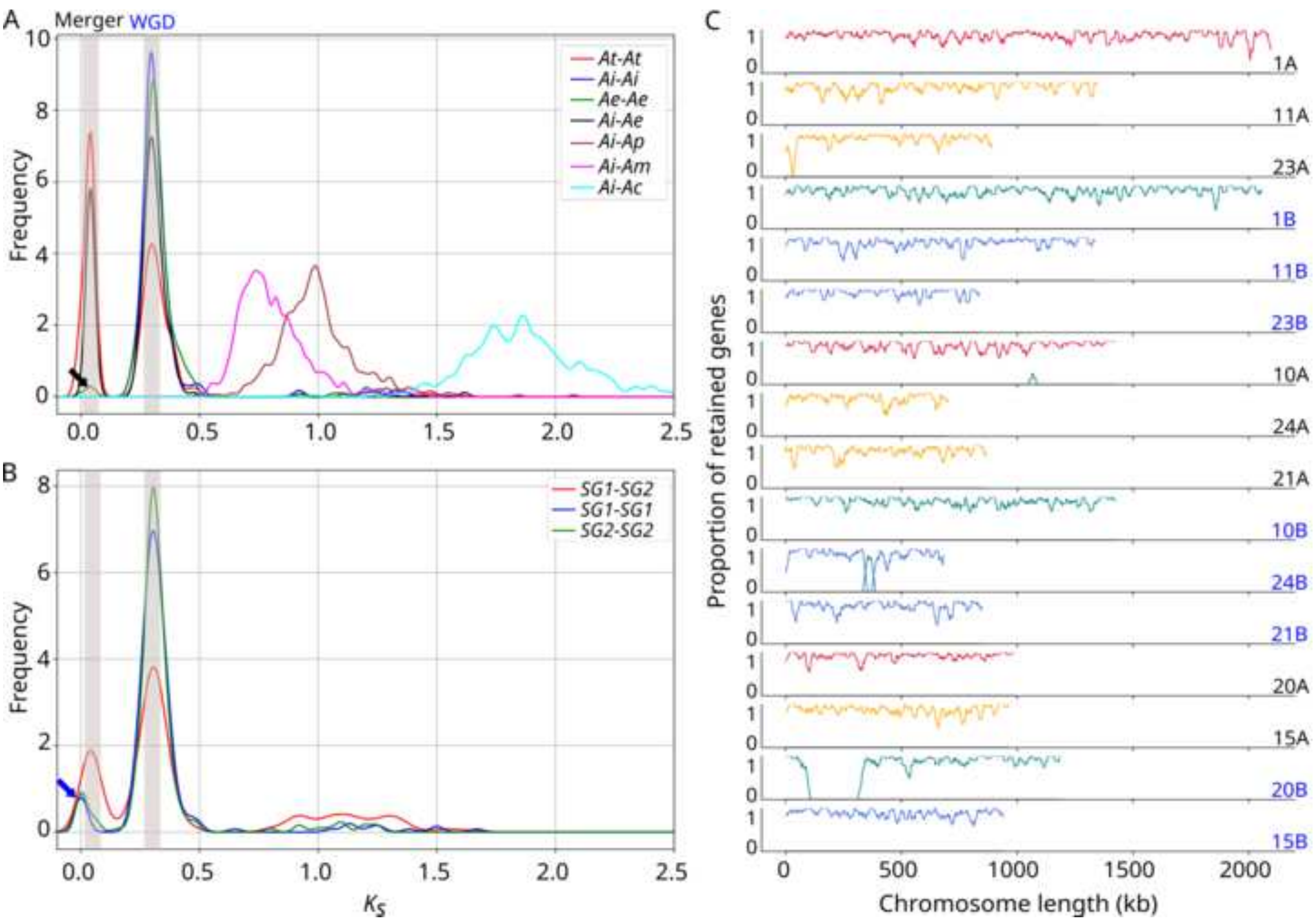

Figure 4

Click here to access/download;Figure;Figure4.tiff

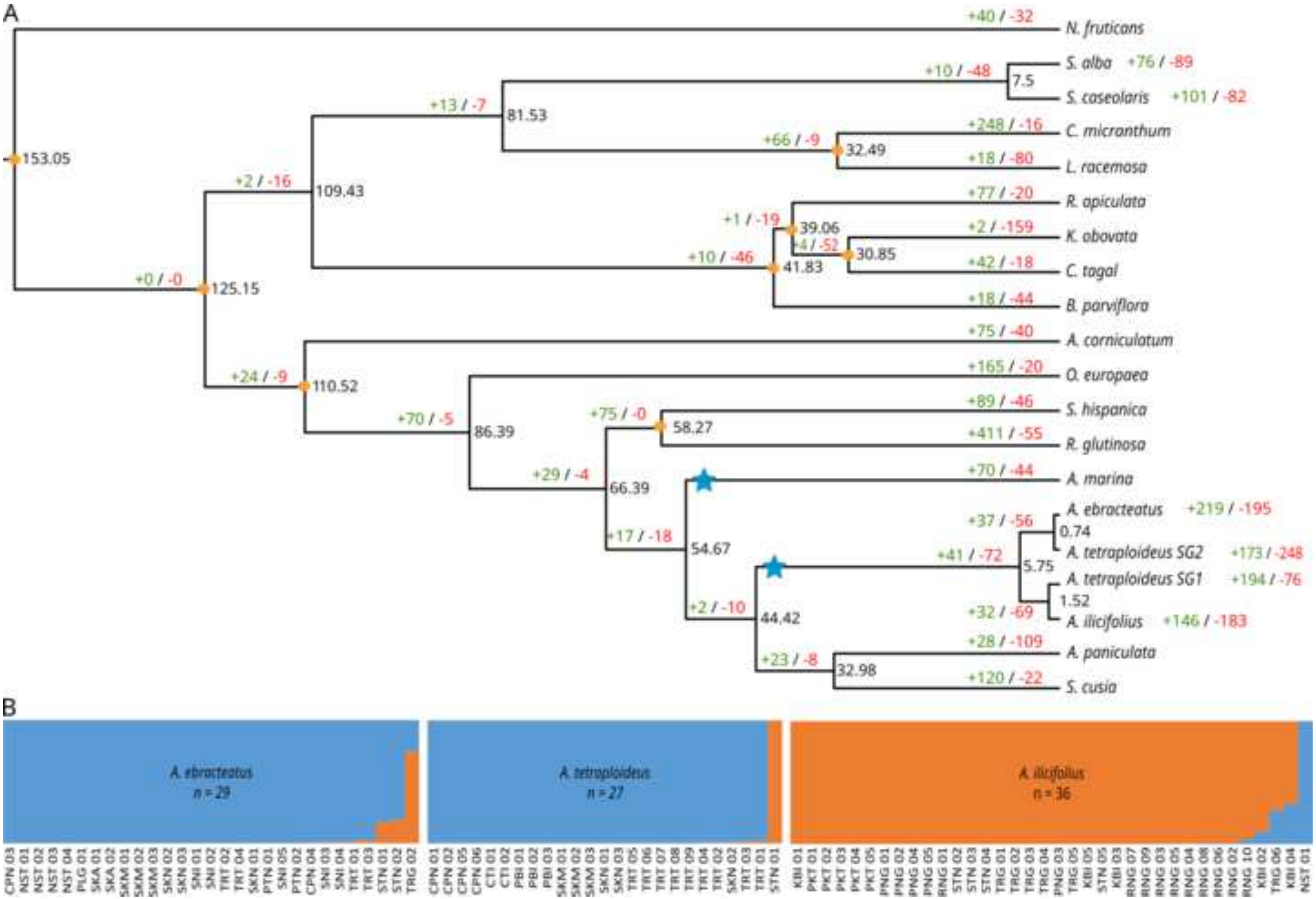

Supplementary Figures S1-S3

Click here to access/download
Supplementary Material
Supplementary_Figures.pdf

Supplementary Table S1

Click here to access/download
Supplementary Material
Supplementary_Table_S1.xlsx

Supplementary Table S2

Click here to access/download
Supplementary Material
Supplementary_Table_S2.xlsx

Supplementary Table S3

Click here to access/download
Supplementary Material
Supplementary_Table_S3.xlsx

Supplementary Table S4

Click here to access/download
Supplementary Material
Supplementary_Table_S4.xlsx

Supplementary Table S5

Click here to access/download
Supplementary Material
Supplementary_Table_S5.xlsx

Supplementary Table S6

Click here to access/download
Supplementary Material
Supplementary_Table_S6.xlsx

Supplementary Table S7

Click here to access/download
Supplementary Material
Supplementary_Table_S7.xlsx

Supplementary Table S8

Click here to access/download
Supplementary Material
Supplementary_Table_S8.xlsx

Supplementary Table S9

Click here to access/download
Supplementary Material
Supplementary_Table_S9.xlsx

Supplementary Table S10

Click here to access/download
Supplementary Material
Supplementary_Table_S10.xlsx

Supplementary Table S11

Click here to access/download
Supplementary Material
Supplementary_Table_S11.xlsx

Supplementary Table S12

Click here to access/download
Supplementary Material
Supplementary_Table_S12.xlsx

Supplementary Table S13

Click here to access/download
Supplementary Material
Supplementary_Table_S13.xlsx

Supplementary Table S14

Click here to access/download
Supplementary Material
Supplementary_Table_S14.xlsx

Supplementary Table S15

Click here to access/download
Supplementary Material
Supplementary_Table_S15.xlsx

Supplementary Table S16

Click here to access/download
Supplementary Material
Supplementary_Table_S16.xlsx
