# Supplementary material for: Genome Assembly of Three Shrub Mangroves in the Genus Acanthus Reveals Two Polyploidy Events and Expansion of Genes Linked to Root Adaptation in Coastal Habitats
Source: Gigascience. 2026 Jan 2;15:giaf162. doi: 10.1093/gigascience/giaf162 (PMC12903786; doi:10.1093/gigascience/giaf162)
Supplement: giaf162_GIGA-D-25-00249_Revision_1 [file giaf162_giga-d-25-00249_revision_1.pdf]

GigaScience

Genome Assembly of Three Shrub Mangroves in the Genus Acanthus Reveals Two Polyploidy Events and Expansion of Genes Linked to Root Adaptation in Coastal Habitats

--Manuscript Draft--

**Manuscript Number:**	GIGA-D-25-00249R1
**Full Title:**	Genome Assembly of Three Shrub Mangroves in the Genus Acanthus Reveals Two Polyploidy Events and Expansion of Genes Linked to Root Adaptation in Coastal Habitats
**Article Type:**	Data Note
**Funding Information:**	National Science and Technology Development Agency (NSTDA) (P2351523) — Not applicable
**Abstract:**	The genomes of mangrove Acanthus species have not been reported, despite their ecological and medicinal importance. Using PacBio and Hi-C data, we generated a chromosome-scale genome assembly of the recently identified allotetraploid species Acanthus tetraploideus (2n = 96). The genomes of diploid progenitors, Acanthus ilicifolius and Acanthus ebracteatus (2n = 48), were assembled from stLFR data. We identified an Acanthus-specific whole-genome duplication (WGD) event that occurred ~43 million years ago (Mya). Ancestral karyotype reconstruction revealed a shift in haploid chromosome number from 11 to 24 in the progenitors, following the WGD and subsequent chromosomal fission events. The hybridization that formed A. tetraploideus was estimated to have occurred 0.7-1.8 Mya. Phylogenomic and synteny analyses clearly showed that A. tetraploideus inherited subgenomes SG1 and SG2 from A. ilicifolius and A. ebracteatus, respectively. Gene structure and retention analyses revealed a smaller and more structurally flexible genome in A. ebracteatus and SG2 compared with A. ilicifolius and SG1. Gene family and machine learning analyses identified expansions in protein families related to Casparian strip formation, root development, and salt stress response. Several of these families were expanded in A. ilicifolius and SG1 but contracted in A. ebracteatus and SG2. These genomic patterns might have contributed to the establishment of A. tetraploideus within the habitat of A. ebracteatus. For all three species, population analysis revealed clear genetic divergence between samples from the eastern and western coasts of Thailand. This study provides valuable genomic resources and insights into the evolutionary adaptation of plants to intertidal environments.
**Corresponding Author:**	Wirulda Pootakham<br>National Science and Technology Development Agency<br>Khlong Luang, Pathum Thani THAILAND
**Corresponding Author Secondary Information:**	
**Corresponding Author's Institution:**	National Science and Technology Development Agency
**Corresponding Author's Secondary Institution:**	
**First Author:**	Wanapinun Nawae, Ph.D.
**First Author Secondary Information:**	
**Order of Authors:**	Wanapinun Nawae, Ph.D.
	Chaiwat Naktang
	Peeraphat Paenpong
	Duangjai Sangsrakru
	Thippawan Yoocha
	Sonicha U-thoomporn

Powered by Editorial Manager® and ProduXion Manager® from Aries Systems Corporation

	Wasitthee Kongkachana
	Poonsri Wanthongchai
	Suchart Yamprasai
	Chonlawit Samart
	Sithichoke Tangphatsornruang, Ph.D.
	Wirulda Pootakham, Ph.D.
**Order of Authors Secondary Information:**	
**Response to Reviewers:**	Editor's Comment<br>All web links and URLs should be given a reference number and included in the reference list rather than within the text of the manuscript. Please remove the URLs, cite them as reference and adjust the order of the reference accordingly. If you are using a reference manager then it's much easier for you to fix this. Response: All web links and URLs are now given a reference number and included in the reference list.<br>Response:<br>All web links and URLs have been assigned reference numbers and are now included in the reference list. The in-text URLs have been removed.<br>In the methods section it would be useful if you add RRID details to some of the software tools and sequencers. These needed to be listed after resources in brackets. If you are citing papers for these resources, the RRID do not replace these, and both should be included. This can be included in the methods section of the paper similar to the RRIDs included here: DNBSEQ-T7 (RRID:SCR_017981); PacBio Sequel II System (RRID:SCR_017990); PLINK (RRID:SCR_001757)<br>Response:<br>RRID identifiers have been added for the relevant software tools and sequencing platforms in the Methods section, following the format suggested by the editor.<br><br><br>Reviewer #1:<br>This paper makes significant contributions to the genome assembly and polyploidization analysis of the Acanthus genus, particularly in identifying adaptation-related genes in mangrove plants. The paper is well-structured, logically organized, and supported by rich figures and tables, which help readers understand the complex da ta and analysis results. However, there are still the following suggestions for this article:<br>Ln 80 In the final paragraph of the introduction, please use the present tense, and ensure that the first occurrence of any abbreviation is accompanied by its full form in parentheses, such as stLFR (single-tube long fragment read), to facilitate reader comprehension. Additionally, please review the rest of the article for consistency.<br>Response:<br>We thank the reviewer for this helpful suggestion. The final paragraph of the Introduction has been revised to use the present tense throughout (lines 77–84 in the revised manuscript; line numbers in the revised MS word file may differ from those in the generated PDF). The full form of each abbreviation, including stLFR (single-tube long fragment read), has been provided at its first occurrence. We have also reviewed the entire manuscript to ensure consistent usage of tense and abbreviations.<br>Ln 218 The estimated genome size in Supplementary Table S1 ranges from 1366 to 2496 Mb, which is a very wide range. Please specify which individual in the table corresponds to the assembled individual, as this is necessary to determine whether the assembled size matches the estimated genome size.<br>Response:<br>We thank the reviewer for pointing this out. In the revised manuscript, we have specified that the SKN-At-01 individual listed in Supplementary Table S1 was used for Acanthus tetraploideus genome sequencing (lines 273-274).<br>Ln 220, Ln 223 Fig 1A shows a plant photograph and does not display genome-related data, while Fig 1B is a Hi-C contact heatmap, which also does not align with the description in the text. Please verify the correspondence between all figure captions, table captions, and the content of the article. Additionally, the high repeat density observed in the central regions of chromosomes does not necessarily correspond to centromeric regions; it is essential to confirm whether these regions contain centromeric repeat sequences.

Response:
We thank the reviewer for this careful observation. In the revised manuscript, we have verified and corrected the correspondence between all figure citations and the text. We revised the citations of Fig. 1B (line 276), Fig. 1C (lines 279 and 287), and Fig. 1D (lines 298 and 309). Regarding the statement about centromeric regions, we agree with the reviewer's comment. Instead of referring to these as centromeric regions, we now describe them more cautiously as repeat-dense regions, with gene-annotated regions located outside these areas (lines 278-279).
Ln 232 Is there any literature or experimental evidence supporting the identification of these two species as the putative progenitors? According to Supplementary Table 11, most of the alignment thresholds are below 75%. If there are other diploid species within this genus, it cannot be conclusively determined that these two species are the progenitors. The accuracy of homologous scaffolding is also questionable, which further casts doubt on the reliability of subsequent analyses.
Response:
We thank the reviewer for this insightful comment. In the revised manuscript, we added supporting evidence from recent studies (Feng et al., 2024; Guo et al., 2025), which used chloroplast, nuclear, and transcriptomic data to confirm A. ilicifolius and A. ebracteatus as the most likely progenitors of A. tetraploideus, while excluding other congeners such as A. volubilis (lines 67–69).
We agree that the overall identity values are relatively modest. This is because the values were calculated across long stretches of collinearity, where sequence divergence between the subgenomes and their progenitor genomes has accumulated over time. Nevertheless, multiple lines of genome-wide evidence, including synteny block analysis (Fig. 1D) and phylogenetic reconstruction Fig. 5 (renumbered from Fig. 4), consistently reveal one-to-one correspondences between SG1 and A. ilicifolius, and between SG2 and A. ebracteatus. We have clarified this point in lines 293–298 of the revised manuscript.
Regarding scaffolding accuracy, we clarified that RagTag was used with minimap2 (asm5) for assembly-to-assembly alignment, which orders contigs without altering sequences (lines 149-154). This approach yielded 24 pseudochromosomes per progenitor, capturing >93% of total lengths with strong collinearity, supporting the reliability of the scaffolding (lines 300-302).
References:
Feng H, Banerjee AK, Guo W, Yuan Y, Duan F, Ng WL, et al. Origin and evolution of a new tetraploid mangrove species in an intertidal zone. Plant Diversity. 2024; doi:10.1016/j.pld.2024.04.007.
Guo W, Banerjee AK, Feng H, Ng WL, Wu H, Li W, et al. Recent allopolyploidization and transcriptomic asymmetry in the mangrove shrub Acanthus tetraploideus. BMC Genomics. 2025; doi:10.1186/s12864-025-11557-2.
(Reference numbers 1 and 4 in the revised manuscript.)
Ln 272 How were the WGD time and divergence time in Fig 2B obtained prior to the construction of the phylogenetic tree, and are they accurate, especially without 95% confidence interval? Additionally, how was the hybridization time between the two species calculated?
Response:
We thank the reviewer for this insightful comment. In the revised manuscript, the WGD and divergence times previously shown in Fig. 2B have been removed to avoid confusion, as these values were derived from the phylogenetic framework later presented in Fig. 5 (renumbered from Fig. 4). We also noted in lines 313-314 that the reconstructed ancestral karyotype corresponded to that of Lamiales (Chen et al., 2024). To address the reviewer's concern regarding accuracy, 95% confidence intervals from the MCMCTree analysis were added to Fig. 5, and the divergence times in the text were mentioned with the 95% confidence intervals.
The hybridization time leading to the formation of the allotetraploid A. tetraploideus was estimated using transposable element (TE) divergence profiles, following the approach of Xu et al. (2019) and Wang et al. (2024). This method compares divergence distributions of LTR retrotransposons between subgenomes, where their initial separation reflects progenitor divergence and subsequent convergence indicates genome merger. This TE-based approach complements Ks-based dating and was applied to the SG1 and SG2 profiles to infer the timing of hybridization. Corresponding revisions have been made in the Methods (lines 220–228) and Results (lines 446–453) sections of the revised manuscript.
References:

Xu P, Xu J, Liu G, Chen L, Zhou Z, Peng W, et al.. The allotetraploid origin and asymmetrical genome evolution of the common carp Cyprinus carpio. Nat Commun. Nature Publishing Group; 2019; doi: 10.1038/s41467-019-12644-1.
Wang Y, Li Y, Wu W, Shao S, Fang Q, Xu S, et al.. The evolution history of an allotetraploid mangrove tree analysed with a new tool Allo4D. Plant Biotechnology Journal. 2024; doi: 10.1111/pbi.14281.
Chen B-Z, Li D-W, Luo K-Y, Jiu S-T, Dong X, Wang W-B, et al.. Chromosome-level assembly of Lindenbergia philippensis and comparative genomic analyses shed light on genome evolution in Lamiales. Front Plant Sci. Frontiers; 2024; doi: 10.3389/fpls.2024.1444234.
(Reference numbers 47-48 and 57 in the revised manuscript.)

Ln 293 Ln 299 Has the Ks peak been fitted and the corresponding peak range obtained, as this step significantly impacts the determination of the WGD timing? Additionally, Ks values very close to 0 are most likely caused by tandem duplications, which could result in the subsequent peaks being shifted forward. Has filtering been applied to remove these tandem duplications?

Response:

We thank the reviewer for this valuable comment. We replaced the previous kernel density estimation (KDE) with Gaussian mixture modeling (GMM) fitting, which provided a smoother and more accurate fit of the Ks distribution. We also applied a tandem duplication filter, and the minor peaks with Ks values close to 0 disappeared from the plots. Accordingly, we removed the text that previously referred to these minor peaks. The new plots showed a slight shift in the positions of both major peaks (from 0.04 to 0.05 and from 0.3 to 0.35). These new values were used to infer WGD and hybridization timing. In addition, we observed a broader peak around Ks ≈ 1.2 in A. tetraploideus, A. ebracteatus, and A. ilicifolius, as well as SG1 and SG2. Based on the updated results, Fig. 3 and the “3. Genome evolution” subsection of the results (line 338-359) have been revised.

Ln 322 What is the biological significance of sequence modifications, and are the genomic differences between the two species and the two subgenomes related to environmental adaptation and trait variation?

Response:

We thank the reviewer for this insightful question. A. ebracteatus and SG2 exhibit greater sequence and structural variation than A. ilicifolius and SG1, consistent with previous reports of higher nucleotide diversity and novel expression bias (Feng et al., 2024; Guo et al., 2025). Such genomic asymmetry may have facilitated both the formation and adaptive success of the allotetraploid (Bureš et al., 2004; Cai et al., 2021; Wang et al., 2022), with SG1 providing regulatory stability and SG2 contributing adaptive flexibility. These findings indicate that the observed sequence modifications are biologically meaningful and linked to the environmental adaptation of A. tetraploideus. This discussion has been added to lines 561–570 of the revised manuscript.

As noted in the previous comment, the text referring to minor peaks has been removed. The original sentence, “These results, together with the presence of a small peak in the Ae–Ae Ks plot (Fig. 3A), suggested a greater degree of sequence modification in the A. ebracteatus genome compared to that of A. ilicifolius,” has been replaced with: “These results were consistent with previous studies showing higher nucleotide diversity and novel expression bias in A. ebracteatus and SG2 compared with A. ilicifolius and SG1 (Feng et al., 2024; Guo et al., 2025),” summarizing the relevant findings described in the paragraph (lines 375–377).

We also revised the abstract for accuracy by replacing “Gene structure and retention analyses suggested greater structural instability in the A. ebracteatus genome compared to the A. ilicifolius genome” with “Gene structure and retention analyses revealed a smaller and more structurally flexible genome in A. ebracteatus and SG2 compared with A. ilicifolius and SG1,” which is more directly supported by the results (lines 32-33).

References:
Feng H, Banerjee AK, Guo W, Yuan Y, Duan F, Ng WL, et al.. Origin and evolution of a new tetraploid mangrove species in an intertidal zone. Plant Diversity. 2024; doi: 10.1016/j.pld.2024.04.007.
Guo W, Banerjee AK, Feng H, Ng WL, Wu H, Li W, et al.. Recent allopolyploidization and transcriptomic asymmetry in the mangrove shrub Acanthus tetraploideus. BMC Genomics. 2025; doi: 10.1186/s12864-025-11557-2.
BUREŠ P, WANG Y-F, HOROVÁ L, SUDA J. Genome Size Variation in Central

Powered by Editorial Manager® and ProduXion Manager® from Aries Systems Corporation

European Species of Cirsium (Compositae) and their Natural Hybrids. Annals of Botany. 2004; doi: 10.1093/aob/mch151.
Cai X, Chang L, Zhang T, Chen H, Zhang L, Lin R, et al.. Impacts of allopolyploidization and structural variation on intraspecific diversification in Brassica rapa. Genome Biology. 2021; doi: 10.1186/s13059-021-02383-2.
Wang Z, Yang J, Cheng F, Li P, Xin X, Wang W, et al.. Subgenome dominance and its evolutionary implications in crop domestication and breeding. Hortic Res. 2022; doi: 10.1093/hr/uhac090.
(Reference numbers 1, 4 and 90-92 in the revised manuscript.)

Ln 334 The use of only 70 single-copy genes is notably insufficient and may significantly impact the stability of the phylogenetic tree's topology. It is recommended to replace some species and the outgroup. Additionally, neither the divergence time nor the WGD time is accompanied by a 95% confidence interval. Has bootstrap validation been performed to confirm their accuracy?

Response:

We thank the reviewer for this important comment. The moderate number of single-copy orthologues reflects both the broad taxon sampling and the recent WGD shared by all Acanthus species, which together reduced the number of genes retained as single copies. Data of comparable scale have been sufficient to resolve species relationships in previous genome studies (Shen et al., 2021; Zhang et al., 2022). Our sampling strategy, which included both mangrove and non-mangrove species, was designed to maximize taxonomic diversity and ensure robust phylogenetic inference across lineages. Bootstrap validation was performed using 1,000 replicates in RAxML-NG, with most internal nodes showing 100% support. Additionally, the resulting topology is consistent with previous genomic and plastid-based phylogenies of mangroves and Acanthaceae (Xu et al., 2017; He et al., 2022; Pootakham et al., 2022), confirming the robustness of our phylogenetic reconstruction. We have now included 95% highest posterior density (HPD) intervals for both divergence and WGD times in Fig. 5 (renumbered from Fig. 4). These updates and clarifications were additionally shown in the Methods (lines 197–199) and Results (lines 394–400) sections of the revised manuscript.

References:
Shen Z, Li W, Li Y, Liu M, Cao H, Provart N, et al.. The red flower wintersweet genome provides insights into the evolution of magnoliids and the molecular mechanism for tepal color development. The Plant Journal. 2021; doi: 10.1111/tpj.15533.
Zhang B, Yao X, Chen H, Lu L. High-quality chromosome-level genome assembly of Litsea coreana L. provides insights into Magnoliids evolution and flavonoid biosynthesis. Genomics. 2022; doi: 10.1016/j.ygeno.2022.110394.
Xu L, Wang X-R, Sun K, Yu T, Xu J-H, Ding P-X, et al.. The complete chloroplast genome of Acanthus ilicifolius, an excellent mangrove plant. Mitochondrial DNA Part B. Taylor & Francis; 2021; doi: 10.1080/23802359.2021.1884022.
He Z, Feng X, Chen Q, Li L, Li S, Han K, et al.. Evolution of coastal forests based on a full set of mangrove genomes. Nat Ecol Evol. Nature Publishing Group; 2022; doi: 10.1038/s41559-022-01744-9.
Pootakham W, Sonthirod C, Naktang C, Kongkachana W, U-thoomporn S, Phetchawang P, et al.. A de novo reference assembly of the yellow mangrove Ceriops zippeliana genome. G3 Genes|Genomes|Genetics. 2022; doi: 10.1093/g3journal/jkac025.
(Reference numbers 60-64 in the revised manuscript.)

Ln 353 According to this formula, the WGD time is significantly influenced by the calculated divergence time. Has it been corrected based on the substitution rate?

Response:

We thank the reviewer for this important observation. The timing of the whole-genome duplication (WGD) event was estimated from peaks in the Ks distribution, following approaches used in previous genomic studies (Sensalari et al., 2022; Padgitt-Cobb et al., 2023). In our analysis, the substitution rate (μ) was inferred by calibrating Ks values against the divergence time between A. ilicifolius and A. ebracteatus, which was independently estimated using MCMCTree with multiple fossil calibrations under a relaxed molecular clock framework. The derived substitution rate was then incorporated into the calculation according to the relationship:

$$2\mu = \frac{Ks_{speciation}}{T_{speciation}} = \frac{Ks_{WGD}}{T_{WGD}}$$

Here, μ represents the lineage-specific substitution rate. Therefore, the WGD estimate was corrected for substitution rate variation as accounted for by the MCMCTree calibration. To improve clarity, we have revised the Methods (lines 203–228) and

Powered by Editorial Manager® and ProduXion Manager® from Aries Systems Corporation

Results (lines 408-418 and 446–453) sections in the revised manuscript.
References:
Sensalari C, Maere S, Lohaus R. ksrates: positioning whole-genome duplications relative to speciation events in KS distributions. Bioinformatics. 2022; doi: 10.1093/bioinformatics/btab602.
Padgitt-Cobb LK, Pitra NJ, Matthews PD, Henning JA, Hendrix DA. An improved assembly of the “Cascade” hop (Humulus lupulus) genome uncovers signatures of molecular evolution and refines time of divergence estimates for the Cannabaceae family. Hortic Res. 2023; doi: 10.1093/hr/uhac281.
(Reference numbers 44-45 in the revised manuscript.)
Ln 364 Does the CV value support K=2 as the optimal choice?
Response:
We thank the reviewer for the comment. Details of the population structure analysis have been clarified in the Methods (Lines 254–267). STRUCTURE analysis (K = 1–10) identified K = 2 as the optimal number of clusters based on the ΔK method in Structure Harvester. This grouping corresponds to two major genetic groups consistent with the geographic distribution of samples along the Andaman Sea and Gulf of Thailand coasts. Relevant text (Lines 435–438) has been revised accordingly.
Ln 416, Ln 432 Is the expansion and contraction of gene families related to the medicinal properties and floral trait differences mentioned in the abstract? Additionally, what specific machine learning methods were used, and what parameters and code were applied?
Response:
We thank the reviewer for this insightful question. We observed contractions in terpenoid biosynthesis families, suggesting a shift in secondary metabolism associated with mangrove adaptation, in contrast to the terpenoid-rich terrestrial medicinal relatives A. paniculata and S. cusia. These results were mentioned in lines 477-486 and further clarified in the Discussion (Lines 534-538) of the revised manuscript.
For floral traits, A. ilicifolius showed an expansion of chalcone synthase (CHS), consistent with its high flavonoid/phenolic content and violet flowers, whereas A. ebracteatus and A. tetraploideus displayed white and pale-violet flowers, respectively. These results were mentioned in Lines 491–493 and further clarified in the Discussion (Lines 551–555) of the revised manuscript.
Regarding the machine learning analysis, we employed logistic regression, random forest (500 estimators), and gradient boosting (learning rate = 0.1), implemented in Python using scikit-learn. Orthogroups were prefiltered using phylogenetic signal metrics and Welch’s t-test before applying recursive feature elimination. The methodology has been detailed in the revised Methods section (Lines 235–253), and all codes and parameters have been provided in the GigaDB database.
Ln 452 The discussion section could be more concise to avoid repetition of results. Could it focus more on the biological significance and future research directions?
Are all the codes and scripts used for analysis provided? This is crucial for the reproducibility of the study.
Response:
We thank the reviewers for these valuable suggestions. In lines 516-585 of the manuscript, the Discussion section has been restructured to improve clarity, focus, and logical flow. Instead of repeating results, the revised text emphasizes the biological significance of our findings and situates them within the broader evolutionary and ecological context of mangrove adaptation. We now highlight how ancient WGD, lineage-specific gene family evolution, and recent hybridization jointly shaped the evolutionary trajectory of Acanthus. We have also added a conclusion and future research directions at the end of the discussion.

Reviewer #2:
This study presents valuable new genome assemblies for three Acanthus mangrove species, employed to investigate their evolutionary history—including an ancient whole-genome duplication (WGD) event and a recent allopolyploidization—as well as adaptive gene family evolution and population structure. While the breadth of analyses is commendable, the current work lacks sufficient analytical depth and a cohesive narrative. Connections between distinct analytical components are often unclear, and comparative genomic analyses, in particular, require more thorough investigation.
Major points:
In Line 285, the Ai-Ae comparison exhibits a peak trend opposite to that of the At-At

Powered by Editorial Manager® and ProduXion Manager® from Aries Systems Corporation

comparison. Further analysis is advised to elucidate the underlying reasons for this discrepancy.

Response:

We thank the reviewer for this valuable suggestion. In the original analysis, Ks distributions were estimated using Gaussian kernel density estimation (KDE). In the revised version, we replotted the Ks histograms and applied Gaussian mixture modeling (GMM) to visualize the distributions. The updated distribution plots have been updated in Fig. 3. In these new plots, the peak patterns in the Ai–Ae and At–At comparisons now follow the same trend. The subsection describing the Ks analysis has been reorganized in lines 338–359 (line numbers in the revised MS word file may differ from those in the generated PDF) of the revised manuscript.

In Lines 299 and 302, the two noted sets of Ks-related genes should be explicitly identified and screened. I recommend analyzing whether their distribution within each genome is random and investigating whether they exhibit significant enrichment in KEGG or GO functional categories.

Response:

We thank the reviewer for this valuable suggestion. As noted by another reviewer, we reanalyzed the Ks distribution and found that the minor peaks near Ks ≈ 0 resulted from tandem duplication. After applying tandem duplication filtering, these minor peaks disappeared. We, therefore, removed the text mentioning these peaks in the revised manuscript.

In Line 326, since the 176 genes annotated in this region do not derive from any ancestral genome sequence, it is critical to discuss the potential origin of this genomic region. Additionally, 105 of these 176 genes belong to three gene families; please elucidate the functions of these families. The results suggest this region may have arisen via tandem or segmental duplication, and further in-depth analysis of its origin and the characteristics of its constituent genes is recommended.

Response:

We thank the reviewer for this insightful comment. In the revised manuscript, we clarified that the 176 genes in the extended region of chromosome 20B do not form any collinearity blocks with the progenitor genomes but likely originated through segmental duplication, supported by the detection of multiple internal collinearity blocks. Among the three gene families identified, we visualized the gene tree of the largest family, which showed 33 paralogous gene copies in A. tetraploideus that were orthologous to a single gene in A. ebracteatus. Similar duplication patterns were also observed in L. racemosa, C. micranthum, and A. paniculata within the same tree. We have created new Fig. 4 combining the gene retention analysis results previously shown in Fig. 3C with this gene tree. Consistently, previous studies have shown that local duplications often contribute to genome expansion and structural variation in polyploids (Qiao et al., 2019). BLAST searches of these 105 proteins revealed matches to proteins from multiple plant species, which were annotated as uncharacterized or hypothetical. Further investigation is needed in the future to clarify their functions and evolutionary significance. The results have been revised accordingly in lines 378-392.

References

Qiao X. et al. (2019). Gene duplication and evolution in recurring polyploidization–diploidization cycles in plants. Genome Biology, 20: 38. https://doi.org/10.1186/s13059-019-1650-2

(Reference numbers 59 in the revised manuscript.)

In Line 359, the explanation that inconsistent divergence times (between the tetraploid's subgenomes and their diploid progenitors) stem from differences in geographic distribution is overly simplistic. Specific underlying mechanisms must be elaborated. For example, investigate whether this discrepancy could arise from post-formation gene introgression via backcrossing between the allopolyploid and its diploid ancestors.

Response:

We thank the reviewer for this valuable suggestion. The collection sources of our materials (A. ilicifolius from the Andaman coast and A. ebracteatus and A. tetraploideus from the Gulf of Thailand), together with the observed genetic separation between Andaman and Gulf populations, suggest a link between their geographic distributions and evolutionary divergence. We hypothesize that the sequenced A. ilicifolius individual from the Andaman coast may have diverged substantially from the ancestral A. ilicifolius lineage that contributed as SG1 in A. tetraploideus around ~5.7 Mya. In contrast, A. ebracteatus and A. tetraploideus still co-occur in the Gulf of Thailand and may therefore have been subjected to more similar environmental

Powered by Editorial Manager® and ProduXion Manager® from Aries Systems Corporation

conditions. Consequently, the divergence between A. ilicifolius and SG1 appears greater than that between A. ebracteatus and SG2.

We agree that geographic separation alone cannot fully explain this pattern. However, our current dataset, which lacks A. ilicifolius genome data from the Gulf of Thailand, does not permit a formal test of introgression. In Response: to the reviewer's suggestion, we have clarified in the revised manuscript that spatial overlap between A. ebracteatus and A. tetraploideus could have facilitated post-formation gene flow or backcrossing, potentially contributing to the lower divergence observed between SG2 and its diploid progenitor. The continued geographic proximity of these species may have promoted stronger genomic continuity or reduced divergence along the SG2 lineage. All these clarifications have been added to lines 424–434 in the revised manuscript.

In Line 364, the manuscript must clarify its SNP calling strategy in the Methods section, including details of the reference genome used, specific procedures, and all software parameters. The current variant calling approach appears methodologically flawed: if the allotetraploid A. tetraploideus genome was used as the reference, calling SNPs from diploid samples (A. ilicifolius and A. ebracteatus) would be highly problematic due to ambiguous read mapping between homeologous subgenomes, leading to unreliable variant data. Conversely, using a diploid genome as the reference would introduce severe mapping bias for tetraploid reads, as reads from the non-reference subgenome would fail to align properly, resulting in an incomplete and skewed SNP dataset. This methodology is thus not robust for this diploid-polyploid system, and the validity of downstream analyses is questionable.

Response:

We thank the reviewer for this insightful comment. In the revised manuscript, we have clarified the SNP calling strategy in a new subsection of the Methods (Lines 254–260). To avoid mapping bias in the diploid–polyploid system, RADseq reads from each species were mapped to their respective genome assemblies, and SNPs were called independently for each population. This strategy minimized cross-subgenome misalignment and ensured reliable variant detection across both diploid and tetraploid genomes.

The population structure analysis is also described in detail in the revised Methods (Lines 261–267), including the use of STRUCTURE v2.3.4 with 20 replicates for K = 1–10, 100,000 burn-in steps, and 500,000 MCMC iterations under an admixture model. The optimal K = 2 was determined using the ΔK method implemented in Structure Harvester, revealing two major genetic clusters corresponding to the Andaman and Gulf of Thailand coasts.

In Line 377, supporting scientific literature must be provided to validate the use of TE divergence rates for dating subgenome merger and divergence events. The current approach— inferring the timing of this event solely from the intersection of two kernel density estimates of LTR retrotransposon divergence in Supplementary Fig. S3—lacks sufficient theoretical and methodological justification. Pinpointing this intersection as the definitive merger time is an overreach, as it is not adequately supported by a single data feature and requires validation via established methods.

Response:

We appreciate the reviewer's comment. The age of the hybridization event leading to the allotetraploid A. tetraploideus was inferred from transposable element (TE) divergence profiles, following the approach of Xu et al. (2019). This method was also used by Wang et al. (2024) to estimate the timing of polyploidization and hybridization events in mangrove evolution. Both studies compared the divergence distributions of LTR retrotransposons between parental subgenomes, showing that the initial separation of TE divergence distributions corresponds to the substitution rate at progenitor divergence, while their subsequent convergence reflects the substitution rate at genome merger. This approach complements Ks-based dating, particularly for the hybridization event where corresponding Ks values were unavailable. Following this framework, we compared the TE divergence profiles of SG1 and SG2 in A. tetraploideus and applied the same calibration method to estimate the timing of the genome merger event. For clarity, we have revised the Methods (lines 203–228) and Results (lines 446–453) in the revised manuscript. Supplementary Fig. S3 has been renumbered as Supplementary Fig. S1 in the revised manuscript.

References:

Xu P, Xu J, Liu G, Chen L, Zhou Z, Peng W, et al.. The allotetraploid origin and asymmetrical genome evolution of the common carp Cyprinus carpio. Nat Commun. Nature Publishing Group; 2019; doi: 10.1038/s41467-019-12644-1.

Powered by Editorial Manager® and ProduXion Manager® from Aries Systems Corporation

	Wang Y, Li Y, Wu W, Shao S, Fang Q, Xu S, et al.. The evolution history of an allotetraploid mangrove tree analysed with a new tool Allo4D. Plant Biotechnology Journal. 2024; doi: 10.1111/pbi.14281.<br>(Reference numbers 47 and 48 in the revised manuscript.)<br>In Line 452, the Discussion section is overly verbose and requires restructuring. It tends to summarize results rather than interpret their implications. Please rewrite this section to be more focused, logical, and analytical, with clear explanations of the significance and context of your findings.<br>Response:<br>We thank the reviewers for these valuable suggestions. In lines 516-585 of the revised manuscript, the Discussion section has been restructured to improve clarity, focus, and logical flow. Instead of repeating results, the revised text emphasizes the biological significance of our findings and situates them within the broader evolutionary and ecological context of mangrove adaptation. We now highlight how ancient WGD, lineage-specific gene family evolution, and recent hybridization jointly shaped the evolutionary trajectory of Acanthus. We have also added a conclusion and future research directions at the end of the discussion.<br>Minor points:<br>In Line 217, please explicitly indicate which table reports the values "a total contig length of 1.95 Gb" and "a contig N50 of 42.67 Mb."<br>Response:<br>We appreciate the reviewer’s comment. These values correspond to the preliminary PacBio HiFi assembly, which served as the template contigs for Hi-C scaffolding. Therefore, they were not presented in a separate table but are directly reported in the text.<br>The numbering of Supplementary Table S2 and all subsequent supplementary tables appears incorrect; please carefully review and revise table numbering accordingly.<br>Response:<br>We appreciate the reviewer’s comment. We have carefully reviewed and corrected the numbering of Supplementary Table S2 and all subsequent supplementary tables throughout the manuscript and supporting files.<br>In Lines 220, 223, 231, and 247, citations to specific panels of Fig. 1 are misaligned with the text. Please revise figure citations to ensure the correct figure components are referenced in their respective contexts.<br>Response:<br>We thank the reviewer for this careful observation. In the revised manuscript, we have verified and corrected the correspondence between figure citations and text. Specifically, we revised the citations of Fig. 1B (line 276), Fig. 1C (lines 279 and 287), and Fig. 1D (lines 298 and 309) in the revised manuscript.<br>In Line 224, Supplementary Table S4 reports BUSCO completeness at the genome level under genome mode, which does not reflect gene annotation completeness. Please provide an additional BUSCO assessment based on predicted protein sequences of all annotated genes to evaluate gene annotation quality.<br>Response:<br>We thank the reviewer for this helpful suggestion. We have performed an additional BUSCO assessment in protein mode using the predicted protein sequences of all annotated genes. The results have been added in lines 304–308 of the revised manuscript and were highly consistent with those obtained from the BUSCO analysis in genome mode.<br>In Line 336, for the phylogenetic tree in Fig. 4A, please use distinct colored blocks to indicate the clade, order, and family of each analyzed species.<br>Response:<br>We thank the reviewer for this helpful suggestion. We have added distinct colored blocks to the phylogenetic tree to indicate the clade, order, and family of each analyzed species. In the revised manuscript, the phylogenetic tree has been renumbered from Fig. 5 (renumbered from Fig. 4).<br>In Line 351, please relocate mathematical formulas to the Methods section for detailed explanation and clarification.<br>Response:<br>We appreciate the reviewer’s comment. The corresponding equation has been relocated to the new subsection titled “7. Estimation of the timing of whole-genome duplication and hybridization events” in the Methods section for detailed explanation and clarification (Lines 203–228).<br>In Line 431, please provide detailed analytical procedures of the machine learning

	approach in the Methods section.<br>Response:<br>We thank the reviewer for this helpful suggestion. In the revised manuscript, we have expanded the Methods section to include a detailed description of the machine learning workflow, covering data inputs, phylogenetic filtering, feature selection, and model implementation. To enhance clarity and accessibility, this content has been organized into a new subsection titled “Gene family expansion/contraction analysis and machine learning-based selection of lineage-specific gene families” (Lines 235–253).<br>In Line 441, add a citation for Table 1 in the main text.<br>Response:<br>We thank the reviewer for the suggestion. A citation for Table 1 has been added to Line 482 of the revised manuscript.
**Additional Information:**	
**Question**	**Response**
Are you submitting this manuscript to a special series or article collection?	No
**Experimental design and statistics**<br><br>Full details of the experimental design and statistical methods used should be given in the Methods section, as detailed in our Minimum Standards Reporting Checklist. Information essential to interpreting the data presented should be made available in the figure legends.<br><br>Have you included all the information requested in your manuscript?	Yes
**Resources**<br><br>A description of all resources used, including antibodies, cell lines, animals and software tools, with enough information to allow them to be uniquely identified, should be included in the Methods section. Authors are strongly encouraged to cite Research Resource Identifiers (RRIDs) for antibodies, model organisms and tools, where possible.<br><br>Have you included the information requested as detailed in our Minimum Standards Reporting Checklist?	Yes
**Availability of data and materials**	Yes

All datasets and code on which the conclusions of the paper rely must be either included in your submission or deposited in publicly available repositories (where available and ethically appropriate), referencing such data using a unique identifier in the references and in the "Availability of Data and Materials" section of your manuscript.<br><br>Have you have met the above requirement as detailed in our Minimum Standards Reporting Checklist?	
GigaScience has policies and guidelines in place for the use of generative AI-writing tools such as ChatGPT. If you have used such writing tools to assist with writing the manuscript this must be declared and cited in the text. Authors should not list AI-writing tools and other AI-assisted technologies as an author or co-author and should acknowledge that they are fully responsible for text generated or refined by AI-writing tools.<p><br><br>A summary of use (particularly in the introduction or among methods) needs to be included at the end of the paper, and the outputs should also be included as a supplementary file hosted in GigaDB or other open repositories. Please read our guidelines for more information. <p><br><br>By submitting to GigaScience, you are aware of the journal's AI-writing tools policy, and if you have declared use of such tools below, you have acknowledged this where appropriate in your manuscript and have made a summary of use and outputs available. <p><br><b>AI-assisted writing tools have been used in the preparation of this manuscript?	No

Manuscript
Click here to access/download;Manuscript;Acnthus_manuscript_revised.docx

Genome Assembly of Three Shrub Mangroves in the Genus *Acanthus* Reveals Two Polyploidy Events and Expansion of Genes Linked to Root Adaptation in Coastal Habitats

Wanapinun Nawae[1], Chaiwat Naktang[1], Peeraphat Paenpong[1], Duangjai Sangsrakru[1], Thippawan Yoocha[1], Sonicha U-thoomporn[1], Wasitthee Kongkachana[1], Poonsri Wanthongchai[2], Suchart Yamprasai[2], Chonlawit Samart[2], Sithichoke Tangphatsornruang[1], Wirulda Pootakham[1*]

[1]National Center for Genetic Engineering and Biotechnology (BIOTEC), National Science and Technology Development Agency (NSTDA), Pathum Thani, Thailand

[2]Department of Marine and Coastal Resources, 120 The Government Complex, Thung Song Hong, Bangkok, Thailand

[*]Corresponding author: wirulda.poo@biotec.or.th

Abstract

The genomes of mangrove *Acanthus* species have not been reported, despite their ecological and medicinal importance. Using PacBio and Hi-C data, we generated a chromosome-scale genome assembly of the recently identified allotetraploid species *Acanthus tetraploideus* (2n = 96). The genomes of diploid progenitors, *Acanthus ilicifolius* and *Acanthus ebracteatus* (2n = 48), were assembled from stLFR data. We identified an *Acanthus*-specific whole-genome duplication (WGD) event that occurred ~43 million years ago (Mya). Ancestral karyotype reconstruction revealed a shift in haploid chromosome number from 11 to 24 in the progenitors, following the WGD and subsequent chromosomal fission events. The hybridization that formed *A. tetraploideus* was estimated to have occurred 0.7-1.8 Mya. Phylogenomic and synteny analyses clearly showed that *A. tetraploideus* inherited subgenomes SG1 and SG2 from *A. ilicifolius* and *A. ebracteatus*, respectively. Gene structure and retention analyses revealed a smaller and more structurally flexible genome in *A. ebracteatus* and SG2 compared with *A. ilicifolius* and SG1. Gene family and machine learning analyses identified expansions in protein families related to Casparian strip formation, root development, and salt stress response. Several of these families were expanded in *A. ilicifolius* and SG1 but contracted in *A. ebracteatus* and SG2. These genomic patterns might have contributed to the establishment of *A. tetraploideus* within the habitat of *A. ebracteatus*. For all three species, population analysis revealed clear genetic divergence between samples from the eastern and western coasts of Thailand. This study provides valuable genomic resources and insights into the evolutionary adaptation of plants to intertidal environments.

Introduction

The genus *Acanthus* (family Acanthaceae) consists of approximately 30 species of flowering plants distributed across tropical and subtropical regions worldwide [1]. *Acanthus* species are highly adaptive as indicated by their significant diversity in morphology and habitat preferences, ranging from terrestrial to mangrove environments [2]. While many *Acanthus* species are terrestrial, three mangrove species—*Acanthus ilicifolius*, *Acanthus ebracteatus*, and *Acanthus volubilis*—inhabit intertidal zones where saltwater and freshwater converge [1]. These species have long been used as medicinal plants across Asia and Oceania [3]. Morphologically, *A. volubilis* is clearly different from the other two species. In contrast, *A. ilicifolius* and *A. ebracteatus* share highly similar leaf morphology, characterized by lanceolate, spiny, and leathery leaves (Fig. 1A). However, their floral characteristics differ, as *A. ilicifolius* produces violet flowers with bracteoles, while *A. ebracteatus* bears smaller white flowers. Recent phylogenetic and biogeographic investigations introduced a new *Acanthus* species, *Acanthus tetraploideus*, with mixed phenotypic and genotypic characteristics from *A. ilicifolius* and *A. ebracteatus* [1]. *A. tetraploideus* has 96 chromosomes (2n = 96), double the chromosome number of *A. ilicifolius* and *A. ebracteatus* (2n = 48) [1]. Exploring the genomes of these three species will provide valuable insights into the evolution of mangrove species within challenging and dynamic coastal habitats.

The genome assembly of *Acanthus* species has not yet been reported, although several sequencing efforts have been made to understand the genetics of this lineage. For example, transcriptomic analyses identified positively selected genes that were related to salt, heat, and ultraviolet stress tolerance in *A. ilicifolius* when compared to its terrestrial relative *Acanthus leucostachyus* [2]. *A. ilicifolius* likely diverged from *A. leucostachyus* approximately 11.6 to 22.1 million years ago (Mya), and the selection of these genes was suggested to be associated with its adaptation to intertidal zones [2]. Additionally, phylogenetic analyses based on chloroplast

genomes, eight nuclear genes, and transcriptome data have identified *A. ilicifolius* and *A. ebracteatus* as the putative progenitors of the allotetraploid *A. tetraploideus* genome [1,4]. These studies yet recommended that whole-genome sequencing is necessary for accurately identifying the of this hybrid the origin of this new allotetraploid species [1,4]. Genome sequencing technologies, including PacBio, Hi-C, and linked-read sequencing, have significantly advanced our understanding of complex plant genomes and their evolution [5]. These technologies have been applied to identify salinity tolerance genes and intertidal adaptations of *Avicennia marina* [6], uncover whole-genome duplication in *Ceriops tagal* [7], and reveal the origin of *Bruguiera hainesii* from the hybridization of *B. gymnorhiza* and *B. cylindrica* [8].

In this study, we generate a chromosome-level genome assembly of the tetraploid species *A. tetraploideus* using PacBio HiFi and Hi-C sequencing data. Additionally, we assemble the diploid genomes of *A. ilicifolius* and *A. ebracteatus*, the candidate parental species of the tetraploid, using single-tube long fragment read (stLFR) sequencing. These high-quality genomic resources enable comprehensive investigations into the evolutionary history, polyploidization events, and adaptive mechanisms of *Acanthus* species. Our work marks a significant advancement in mangrove genomics and provides a valuable foundation for future research on the conservation and sustainable use of these ecologically and economically important plants.

Materials and Methods

1. Plant materials and nucleic acid isolation

Young leaf tissues were collected from mature individuals of *A. ilicifolius*, *A. ebracteatus* and *A. tetraploideus* in natural mangrove habitats in Thailand. Samples of *A. tetraploideus* and *A. ebracteatus* were collected from Samut Sakhon province at coordinates 13°30'24.9"N, 100°16'15.2"E and 13°30'32.4"N, 100°15'16.1"E, respectively. *A. ilicifolius* leaves were obtained from Phuket province at 8°09'53.6"N, 98°18'17.2"E. All leaf samples were immediately flash-frozen in liquid nitrogen in the field and stored at –80°C until processing. High-molecular-weight genomic DNA was extracted using the QIAGEN Genomic-tip 100/G kit following the manufacturer's protocol. DNA quality and integrity were assessed using a Pippin Pulse Electrophoresis System (Sage Science) and quantified with a Qubit fluorometer prior to library preparation.

For transcriptome sequencing, total RNA was isolated from leaf tissues collected from the same individual used for genome sequencing, following the protocol of Pootakham, et al. [9]. Poly(A) mRNA was enriched using the Dynabeads mRNA Purification Kit (Thermo Fisher Scientific, Waltham, USA). The integrity of RNA samples was evaluated using the Fragment Analyzer System (Agilent, Santa Clara, USA) prior to library construction.

We also collected leaf samples from 90 accessions of *A. ilicifolius*, *A. ebracteatus*, and *A. tetraploideus* across mangrove forests in 14 provinces of Thailand. Sampling sites included Chumphon (CPN), Nakhon Si Thammarat (NST), Phatthalung (PLG), Phetchaburi (PBI), Samut Songkhram (SKM), Samut Sakhon (SKN), Surat Thani (SNI), and Trat (TRT) along the Gulf of Thailand, and Krabi (KBI), Phang Nga (PNG), Phuket (PKT), Ranong (RNG), Satun (STN), and Trang (TRG) along the Andaman coast. All leaf tissues were immediately flash-frozen in liquid nitrogen and stored at –80 °C until processing. Genomic DNA was extracted using the CTAB method as described by Pootakham et al. [10], and its quality was assessed using a Qubit fluorometer (Thermo Fisher Scientific). The genome sizes of all samples used in this study were

estimated using flow cytometry on the BD Accuri™ C6 Plus system (BD biosciences) and the maize genome as a reference standard.

2. Library Preparation and Sequencing

High-molecular-weight genomic DNA was sheared to ~15 kb using the Megaruptor® 2 system. SMRTbell libraries were prepared with the SMRTbell Express Template Prep Kit 2.0 (PacBio). The libraries were purified with AMPure PB beads and size-selected (15–18 kb) using the Sage ELF system. Quality and quantity were assessed using FEMTO Pulse and Qubit. Final libraries were bound to Sequel II polymerase and sequenced on the PacBio Sequel II platform (RRID:SCR_017990) using an 8M SMRT Cell with 1800-minute movies. To generate chromosome-scale scaffolds, a Dovetail Omni-C (Hi-C) library was prepared by Dovetail Genomics (Scotts Valley, CA, USA). The protocol involved cross-linking chromatin with formaldehyde, digesting with DNase I, repairing ends, and ligating biotinylated adapters, followed by proximity ligation and purification. Biotinylated DNA fragments were isolated using streptavidin beads, and sequencing libraries were constructed using NEBNext Ultra reagents. The library was sequenced on an Illumina HiSeq X (RRID:SCR_016385). PacBio HiFi and Dovetail Omni-C library preparation, quality control, and sequencing were carried out by BMKGENE (Biomarker Technologies, Beijing, China) following the provider's standard protocols.

For linked-read sequencing of *A. ilicifolius* and *A. ebracteatus*, high-molecular-weight genomic DNA was used to construct stLFR (single-tube long fragment read) libraries using the MGIEasy stLFR Library Prep Kit (MGI Tech, Shenzhen, China). For transcriptome sequencing, polyadenylated mRNA extracted from leaf tissue was used to prepare libraries with the MGIEasy RNA Library Prep Kit v3.0 (MGI Tech). To assess genetic variation across populations, RADseq libraries were constructed from individual samples using the MGIEasy RAD Library

Prep Kit (MGI Tech). All libraries were sequenced on the MGI DNBSEQ-G400 platform (RRID:SCR_017980).

4. Genome assembly and annotation

For *A. tetraploideus*, the genome was assembled using PacBio HiFi long reads and Hi-C scaffolding. HiFi reads were first assembled with Hifiasm 0.25 (RRID:SCR_021069) [11] in Hi-C mode to generate contigs. The Hi-C reads were mapped to the contigs following the pipeline described in Dovetail Omni-C document [12]. Scaffolding was subsequently performed using YaHS 1.2 (RRID:SCR_022965) [13] with default parameters. Juicebox was used to curate the scaffolding results and visualize hi-c contact map. BUSCO 5.2 (RRID:SCR_015008) [14] was used to assess assembly completeness.

The stLFR reads from *Acanthus ilicifolius* and *A. ebracteatus* were assembled using stLFRdenovo with default parameters [15]. D-GENIES 1.5 (RRID:SCR_018967) [16] was used to visualize dot plots of pairwise whole-genome alignments between the allotetraploid *A. tetraploideus* genome with *A. ilicifolius* and *A. ebracteatus* genomes. We used RagTag v2.1 (RRID:SCR_027293) [17], a widely applied scaffolding tool with robust filtering steps, to scaffold *A. ilicifolius* and *A. ebracteatus* contigs using the chromosomes of the corresponding subgenomes as references. RagTag employs minimap2 (RRID:SCR_018550) with default option (-x asm5), optimized for high-quality long-read assemblies, to map the query to the reference and does not alter the query sequences, but only orders and orients them, joining with gaps where necessary.

For gene prediction, genome annotation was performed using BRAKER2 3.0 (RRID:SCR_018964) [18], which integrated RNA-seq evidence and ab initio predictions. RNA-seq data were used as extrinsic evidence to train the gene models. Viridiplantae protein sequences from the OrthoDB 11 database (RRID:SCR_011980) [19] were incorporated to

support and refine gene predictions. Repeat masking was carried out using RepeatModeler (RRID:SCR_015027) and RepeatMasker (RRID:SCR_012954) prior to annotation.

5. Genome analysis

Assembly statistics, including total assembly size, contig/scaffold N50, and number of contigs were generated using QUAST 5.3 (RRID:SCR_001228) [20]. SubPhaser 1.2 [21] was used to assign homoeologous chromosome pairs, which were obtained from D-GENIES alignments, into subgenomes SG1 and SG2. Synteny blocks were then detected using MCScanX 1.0 (RRID:SCR_022067) [22] and JCVI 1.5 (RRID:SCR_021641) [23] with their default parameters. To show the relationship between *A. tetraploideus* chromosomes and progenitor sequences, JCVI was used to visualize pairs of matched synteny blocks. Circos 0.52 (RRID:SCR_011798) [24] was used to show relationship between subgenomes SG1 and SG2. To investigate synonymous substitution rates (Ks) among duplicated and orthologous gene pairs, collinear blocks were first identified using the WGDI pipeline [25]. Ks values for each gene pair within these blocks were calculated using MUSCLE 5.3 (RRID:SCR_011812) [26] and yn00 program of the PAML 4.9 package (RRID:SCR_014932) [27] under WGDI environment [25]. For each block, both the average and median Ks values across collinear gene pairs were extracted and fitted with Gaussian Mixture Models (GMMs) to visualize Ks distributions. To minimize the influence of recent tandem duplications, which can generate spurious low Ks values, paralogous pairs located within 200 base pairs on the same chromosome were excluded prior to plotting. WGDI was also employed to reconstruct ancestral chromosomes of all studied *Acanthus* species based on sorted dot plots of shared synteny blocks. Additionally, gene retention from the parental genomes on *A. tetraploideus* chromosomes was identified using WGDI. The Liftoff program was used to transfer the annotations from reference to target sequences. The completeness of the sequences within the transferred annotation region was checked to investigate possible structural aberrant of unretained genes. Homologous genes used as inputs

for MCScanX, JCVI, and WGDI were identified using BLASTP (RRID:SCR_001010) with e-value cutoff of 10^{-10}.

6. Comparative genomics

Protein sequences from *A. tetraploideus*, *A. ilicifolius*, *A. ebracteatus*, and additional 16 plant species, including *Nypa fruticans*[28], *Lumnitzera racemosa* [29], *Combretum micranthum* [29], *Sonneratia alba* [30], *Sonneratia caseolaris* (https://evolution.sysu.edu.cn/Sequences.html), *Bruguiera parviflora* [31], *Rhizophora apiculate* [32], *Kandelia obovata* [33], *Ceriops tagal* [7], *Aegiceras corniculatum* [34], *Olea europaea* (GCF_002742605.1), *Rehmannia glutinosa* [35], *Salvia hispanica* (GCF_023119035.1), *Avicennia marina* [36], *Strobilanthes cusia* [37], *Andrographis paniculata* [38] were used to identify orthologous groups (orthogroups) using OrthoFinder 2.5 (RRID:SCR_017118) [39]. The protein sequences of single-copy orthologs identified by OrthoFinder were aligned using MUSCLE 5.3 (RRID:SCR_011812) [26]. Poorly aligned regions were trimmed by trimAl 1.5 (RRID:SCR_017334) [40] with heuristic mode (-automated1 option). A phylogenetic tree was inferred from the processed alignment using RAxML-NG 1.2 (RRID:SCR_022066) [41] under the best-fit substitution model determined by ModelTest-NG 0.1 (RRID:SCR_026633) [42]. The tree topology was evaluated using 1,000 bootstrap replicates in RAxML-NG and further validated by comparison with previously published phylogenetic trees of mangroves and Acanthaceae. We used MCMCTree implemented in the PAML 4.9 (RRID:SCR_014932) [27] to calculate divergence times among species in the tree based on fossil calibration times obtained from and the Timetree database (RRID:SCR_021162) [43] and associated references therein.

7. Estimation of the timing of whole-genome duplication and hybridization events

The timing of the whole-genome duplication (WGD) event was estimated from peaks in the Ks distribution, following approaches described in recent genomic studies [44,45], based on the

neutral theory of molecular evolution [46]. In this framework, divergence time (T) is related to the number of synonymous substitutions per synonymous site (Ks) and the neutral substitution rate per site per year (μ), according to:

$$T = \frac{Ks}{2\mu}$$

In this study, the divergence between *A. ilicifolius* and *A. ebracteatus* was used as a calibration point to estimate μ. The divergence time ($T_{speciation}$) was independently inferred using MCMCTree, and the Ks peak corresponding to the WGD event (Ks_{WGD}) was scaled relative to the Ks value at the progenitor divergence peak ($Ks_{speciation}$) to estimate the timing of the WGD. Assuming both events follow the same lineage-specific substitution rate, their relationship can be expressed as:

$$2\mu = \frac{Ks_{speciation}}{T_{speciation}} = \frac{Ks_{WGD}}{T_{WGD}}$$

Rearranging gives:

$$T_{WGD} = \frac{Ks_{WGD}}{Ks_{speciation}} \times T_{speciation}$$

This internally calibrated approach allowed the estimation of the timing of the WGD event using relative Ks values and the independently inferred speciation time.

To estimate the timing of allotetraploidization, we followed a previously established analytical framework [47,48]. Transposable element (TE) divergence profiles for SG1 and SG2 were generated using RepeatMasker (RRID:SCR_012954) and plotted as distributions (Supplementary Fig. S1). The initial separation of TE divergence curves indicated the progenitor speciation event, and their later convergence, after independent evolution, marked the genome merger when both subgenomes began sharing a similar TE substitution rate. The TE divergence values at these two points ($D_{speciation}$ and D_{merger}, respectively) were then calibrated against

$T_{speciation}$ to estimate the timing of allotetraploidization using an equation analogous to the Ks-based estimation:

$$T_{merger} = \frac{D_{merger}}{D_{speciation}} \times T_{speciation}$$

8. Gene family expansion/contraction analysis and machine learning-based selection of lineage-specific gene families

Gene family size changes were analyzed using CAFE 5 (RRID:SCR_005983) [49]. The OrthoFinder (RRID:SCR_017118) protein count table and the calibrated phylogenetic tree were provided as input. A global λ parameter was estimated, and families with significantly expanded or contracted sizes ($p < 0.05$) were identified.

To identify orthogroups that distinguish the Acanthus lineage (Class A) from non-Acanthus species (Class B), we implemented a multi-step analytical pipeline incorporating phylogenetic filtering, statistical testing, and machine learning–based feature selection. The species phylogenetic tree and the protein count matrix from OrthoFinder were used as inputs for the analysis. First, gene count data were normalized and screened to exclude extremely high-count outliers. Z-scores were calculated for each orthogroup, and pre-outliers were defined as those showing unusually high expression in only one or two species. To enrich the feature space with phylogenetically informative signals, Blomberg's K and Pagel's λ were calculated for each orthogroup using the phylosig() function in the R package phytools 2.0 (RRID:SCR_015502) [50]. Orthogroups with intermediate conservation signals ($K > 1$ and $\lambda > 0.5$) were retained for downstream analysis. After filtering orthogroups with zero counts in all selected species out, we performed Welch's t-tests with applying a relaxed threshold ($p < 0.1$) to preliminarily screen for orthogroups showing differential copy number between the two classes. The retained orthogroups were then subjected to recursive feature elimination (RFE) with three classification algorithms—logistic regression, random forest, and gradient boosting—to prioritize orthogroups

with the strongest discriminatory power. These models were implemented with the scikit-learn library in Python. Feature importance scores were derived from model coefficients (logistic regression) or feature importance values (tree-based models). For each classifier, the top 20 ranked orthogroups were designated as lineage-associated gene families.

9. SNP calling and population structure analysis

Paired-end RADseq reads from 90 accessions of *Acanthus ilicifolius*, *A. ebracteatus*, and *A. tetraploideus* were mapped to their respective genome assemblies using BWA v0.7.17 (RRID:SCR_010910) [51] with default settings. SNPs were called separately for each species using the GATK v4.1.4.1 HaplotypeCaller (RRID:SCR_001876) [52]. Only high-quality biallelic SNPs were retained after filtering with the following thresholds: QUAL ≥ 30, read depth between 10× and 200×, minor allele frequency (MAF) ≥ 0.1, and missing data ≤ 5%.

Population structure was inferred from the filtered SNP dataset using STRUCTURE v2.3.4 (RRID:SCR_017637) under a Bayesian model-based framework [53]. Twenty independent replicates were performed for each *K* value ranging from 1 to 10, with a burn-in of 100,000 and 500,000 Markov Chain Monte Carlo (MCMC) iterations under an admixture model with correlated allele frequencies. The most likely number of genetic clusters (*K*) was determined using the ΔK method implemented in Structure Harvester (RRID:SCR_017636) [54,55]. Based on the optimal *K* value, CLUMPP v1.1.2 [56] was used to average individual assignment probabilities across replicates.

Results

1. Genomes of *Acanthus tetraploideus* and its progenitors

We generated a high-quality genome assembly of *Acanthus tetraploideus* using a combination of PacBio HiFi and Hi-C sequencing data. PacBio HiFi sequencing produced 63 Gb of long-read data, with an average read length of 14.36 kb and a sequencing depth of 33X. The HiFi data

assembly yielded a total contig length of 1.95 Gb and a contig N50 of 42.67 Mb, matching the estimated genome size (Supplementary Table S1; sample SKN-At-01). The contigs were scaffolded with a total of 230 Gb of Hi-C data into 48 chromosomes, with a total length of 1.92 Gb and a scaffold N50 of 43.75 Mb (Fig. 1B and Supplementary Table S2). Telomeric repeats (AAACCCT) were identified at both ends of 44 chromosomes, at a single end of three chromosomes, and were absent from only one chromosome (Supplementary Table S3). All chromosomes had repeat-dense central regions, with annotated genes located outside these areas (Fig. 1C). Benchmarking Universal Single-Copy Orthologs (BUSCO) analysis indicated that 99.2% of conserved Embryophyta genes were present (Supplementary Table S4), demonstrating the high completeness of the *A. tetraploideus* assembly. These results indicated a high completeness level of the assemble *A. tetraploideus* genome. BUSCO analysis revealed that duplicated genes accounted for 95.5% of the *A. tetraploideus* genome, reflecting the polyploid nature of the assembled genome. Further analysis of orthologous chromosome pairs using SubPhaser identified two clearly separated subgenomes, SG1 (1.03 Gb) and SG2 (0.89 Gb). Synteny block analysis confirmed a 1:1 relationship between SG1 and SG2 across the genome (Fig. 1C).

The genomes of *Acanthus ilicifolius* and *Acanthus ebracteatus*, the two putative progenitors of *A. tetraploideus*, were sequenced using the stLFR technique. The assembled genome sizes were 0.98 Gb for *A. ilicifolius* and 0.89 Gb for *A. ebracteatus* (Supplementary Table S5-S6). These numbers were also consistent with the genome sizes estimated by flow cytometry (Supplementary Table S7-S8). BUSCO analysis indicated that both assemblies contained 98% of Embryophyta conserved genes, with only 11% classified as duplicated genes (Supplementary Table S9-S10). Although the overall identity values were modest, genome alignments indicated that SG1 of *A. tetraploideus* shared greater similarity with the *A. ilicifolius* assembly, whereas SG2 showed higher similarity with *A. ebracteatus* (Supplementary Table S11). The modest

identity values reflected accumulated sequence divergence within long stretches of collinearity between each subgenome and its corresponding progenitor genome (Fig. 1D and Supplementary Table S11). Based on these matches, the contigs of *A. ilicifolius* and *A. ebracteatus* were scaffolded using SG1 and SG2 as reference sequences, respectively. The scaffolding resulted in 24 pseudochromosomes for each species, covering 93% and 94% of the initial lengths of the *A. ilicifolius* and *A. ebracteatus* assemblies, respectively. The genome annotation revealed 61,044, 30,210 and 30,799 protein-coding gene models in the *A. tetraploideus*, *A. ilicifolius* and *A. ebracteatus*, respectively (Supplementary Table S12-S14). In *A. tetraploideus*, BUSCO analysis indicated that the annotated protein set contained 98% of the conserved Embryophyta genes, with only 6% classified as duplicated (Supplementary Table S15). In *A. ilicifolius* and *A. ebracteatus*, the annotated proteins represented 97% of conserved genes, with 26% identified as duplicated (Supplementary Table S16 and 17). Synteny analysis of conserved gene order among genomes clearly demonstrated that *A. tetraploideus* inherited SG1 from *A. ilicifolius* and SG2 from *A. ebracteatus* (Fig. 1D).

2. Ancestral karyotypes

A dot plot from pairwise comparisons of all chromosomes within the *A. tetraploideus* genome revealed 11 protochromosomes in the ancestral karyotype, each defined by conserved intervals of syntenic blocks (Supplementary Fig. S2). This number of protochromosomes corresponded to that of the ancestral karyotype in Lamiales [57]. Chromosome mapping indicated that 18 chromosomes in each subgenome (SG1 and SG2) aligned with nine ancestral chromosomes in a 2:1 ratio. These results represented the signal of a whole-genome duplication (WGD) event experienced by the ancestor of the Acanthus lineage. The dot plot also showed that most duplicated chromosome pairs displayed extensive rearrangements, with numerous interleaved syntenic fragments, indicating that the WGD occurred long before the divergence of the progenitor lineages (Fig. 2A). Interestingly, the remaining six chromosomes in each subgenome

aligned with the remaining two ancestral chromosomes in a 3:1 ratio (Fig. 2A). For example, ancestral chromosome 1 aligned with chromosomes 1A, 11A, and 23A of SG1, and 1B, 11B, and 23B of SG2. In SG1, for example, chromosome 11A corresponded to approximately two-thirds of 1A, while 23A aligned with the remaining region, indicating that the two duplicated chromosomes were fragmented in the common ancestor of *A. ilicifolius* and *A. ebracteatus*.

Based on these results, we propose that the ancestral lineage of *Acanthus* and related species originally had 11 chromosomes (Fig. 2B). A WGD event doubled the haploid chromosome number to 22 (2n = 44). Subsequent chromosomal fissions increased the haploid chromosome number to 24 (2n = 48). This karyotype has been maintained in both *A. ilicifolius* and *A. ebracteatus*. Hybridization between these two species further raised the haploid chromosome number to 48 (2n = 96), resulting in the formation of the allotetraploid *A. tetraploideus*. The karyotype analysis also showed small segments of different colors embedded within the main bodies of some representative chromosomes of *A. tetraploideus*, *A. ilicifolius*, and *A. ebracteatus*, suggesting that the genomes underwent subtle chromosomal translocations and structural rearrangements (Fig. 2B). These structural modifications indicated limited genomic exchange between the two subgenomes following polyploid formation, implying that *A. tetraploideus* was a recently formed species.

3. Genome evolution

Synteny analysis indicated that the WGD event preceded the hybridization between *A. ilicifolius* (Ai) and *A. ebracteatus* (Ae) that formed *A. tetraploideus* (At). To clarify the sequence of genome duplication and divergence events, we examined the distributions of synonymous divergence (*Ks*) values for orthologous (interspecific) and paralogous (intraspecific) gene pairs from syntenic blocks (Fig. 3). The Ai–Ai, Ae–Ae, and At–At paralogous, as well as the Ai–Ae orthologous, comparisons each showed a *Ks* peak at approximately 0.35–0.40, indicating that *A. ilicifolius* and *A. ebracteatus* shared a recent lineage-specific WGD event (Fig. 3A). In

addition, a broader peak centered around ~1.2–1.4 was consistently detected in all comparisons, suggesting older duplication events. The Ai–Ae orthologous comparison displayed an additional *Ks* peak at approximately 0.05, corresponding to their recent speciation event. This peak was also observed in the At–At paralogous comparison, reflecting the coexistence of both progenitor genomes within the allotetraploid nucleus. Correspondingly, the SG1–SG2 subgenome comparison revealed the same three-peak pattern (Fig. 3B).

A. ilicifolius showed a Ks peak of ~1.0–1.3 when compared with the terrestrial relative *Andrographis paniculata* (Ap) and ~0.9–1.2 with the mangrove relative *Avicennia marina* (Am) within the Acanthaceae family (Fig. 3A). In addition, a *Ks* peak centered around ~1.6–1.8 was observed in the comparison between *A. ilicifolius* and *Aegiceras corniculatum* (Ac), which was a distantly related mangrove species within the Asterids clade. Together, these results indicate that the Ks peak at ~0.35–0.40 represents an *Acanthus*-specific WGD event that occurred after the divergence of the genus from its terrestrial and mangrove relatives, but before the speciation and subsequent hybridization event (Ks ≈ 0.05).

4. The expansion of chromosome sequence

To further examine sequence modifications in the genome, the ratio of genes retained from each progenitor across the chromosomes of *A. tetraploideus* was analyzed (Fig. 4A). A high gene retention ratio across broad chromosomal regions indicated that SG1 and SG2 chromosomes preserved nearly complete sets of genes from their respective progenitor chromosomes, with minimal sequence exchange after hybridization. Notably, evidence of sequence exchange was more pronounced in SG2 than in SG1. For example, the result for chromosome 24B of *A. tetraploideus* showed an insertion of Ae_A scaffold sequence within an Ae_B-derived chromosome (Fig. 4A). Moreover, there was a region on chromosome 20B of *A. tetraploideus* that lacked homologous gene matches from either the Ae_A scaffold or other scaffolds. Using genome sequences, annotation files, and the Liftoff program [58], gene annotations from

chromosome 20B were mapped to the corresponding Ae_A scaffold. The results also showed that the mapped regions on the Ae_A scaffold contained repeat elements. As a result, these sequences, which masked repeat sequences, were not annotated by the annotation pipeline. Sequence translation revealed that many of the mapped annotations contained premature stop codons within their coding regions. These results were consistent with previous studies showing higher nucleotide diversity and novel expression bias in *A. ebracteatus* and SG2 compared with *A. ilicifolius* and SG1 [1,4].

Chromosome 20B was longer than its homeologous chromosomes 15A, 20A, and 15B, as well as the *A. ebracteatus* scaffold Ae_A (Fig. 4A). Gene retention analysis revealed that the extended sequence of chromosome 20B comprised 176 genes that did not form any collinearity blocks with the progenitor genome sequences (Supplementary Table S18). In contrast, multiple internal collinearity blocks were detected within this region (Supplementary Fig. S4), indicating that it originated through segmental duplication. Orthologous analysis further assigned 105 of these genes to three gene families. BLAST research revealed that these genes matched uncharacterized or hypothetical proteins in the NCBI database, although they might play a role in the genome evolution of *A. tetraploideus*. The gene tree of the largest family showed 33 paralogous gene copies in *A. tetraploideus* that were orthologous to a single gene in *A. ebracteatus* (Fig. 4B). Paralogous relationships within this family were also found in other species, including *L. racemosa* (16 copies), *C. micranthum* (13 copies), and *A. paniculata* (10 copies), suggesting recurrent duplication of this gene group in mangrove and medicinal plants. Overall, this segmental duplication pattern likely contributed to the sequence extension observed in *A. tetraploideus*, as reported in other plants [59].

5. Comparative genomics

A phylogenetic tree of *A. ilicifolius*, *A. ebracteatus*, and the two subgenomes of *A. tetraploideus*, along with ten other mangrove species and six non-mangrove species, was constructed based

on the sequences of 70 single-copy orthologous genes (Fig. 5A). Data of comparable scale have been sufficient to resolve species relationships in previous studies [60,61]. The resulting topology was highly consistent with previously reported plastid- and genome-based phylogenies of mangroves and Acanthaceae [62–64]. The tree clearly separated the Rosids and Asterids clades, with the mangrove palm *Nypa fruticans* (monocot) serving as the outgroup. In this phylogeny, mangrove species were predominantly placed within the Rosids, whereas only five mangrove plants, including *Aegiceras corniculatum*, *Avicennia marina*, *A. ilicifolius*, *A. ebracteatus*, and *A. tetraploideus*, were located within the Asterids. *A. corniculatum* represented the earliest diverging lineage among them and the only member of the Ericales in this clade. The remaining species belonged to the order Lamiales, whose common ancestor diverged from *A. corniculatum* approximately 103-116 Mya. Most species in this group, including *Andrographis paniculata* and *Strobilanthes cusia* (both in the family Acanthaceae), are terrestrial medicinal plants. Within Acanthaceae, the *Acanthus* lineage diverged from its terrestrial relatives approximately 33-57 Mya (Fig. 5A). The speciation between *A. ilicifolius* and *A. ebracteatus* was estimated to have occurred approximately 3-8 Mya. Using the relationship between Ks values and divergence time (see Methods), the WGD event was dated by calibrating the Ks peak corresponding to the duplication event (Ks_{WGD} = 0.35) against the Ks value for progenitor divergence ($Ks_{speciation}$ = 0.05) and its associated divergence time ($T_{speciation}$ = 3-8 Mya). The WGD event was estimated to have occurred between 21-56 Mya, during the period when the Acanthus lineage diverged from its terrestrial relatives. Based on the estimated progenitor divergence time of 5.75, the substitution rate within *Acanthus* lineage was 4.3×10^{-9} substitutions per site per year. According to this rate, the major duplication event associated with the oldest peak within the *Acanthus* lineage (Ks ≈ 1.2-1.4) was estimated to have occurred approximately 138-161 Mya.

A new tetraploid species, recently named *A. tetraploideus* [1], was later present within *Acanthus* lineage through the hybridization of *A. ilicifolius* and *A. ebracteatus*. To estimate the genome merger time, the divergence time between each subgenome and its corresponding progenitor was estimated. The results, however, yielded two distinct divergence times. The SG1 subgenome was estimated to have diverged from *A. ilicifolius* approximately 0.7-2.5 Mya, whereas SG2 diverged from *A. ebracteatus* about 0.3-1.2 Mya. The divergence pattern between the subgenomes of *A. tetraploideus* and its diploid progenitors appeared to be influenced by geographic history and gene flow. The primary habitats of *A. ilicifolius* on the Andaman coast and *A. ebracteatus* and *A. tetraploideus* in the Gulf of Thailand suggested that geographic isolation contributed to their evolutionary divergence. The sequenced *A. ilicifolius* individual (collected from the Andaman coast) may have diverged substantially from the ancestral *A. ilicifolius* lineage that contributed as SG1 in *A. tetraploideus*. In contrast, *A. ebracteatus* and *A. tetraploideus* still co-occurred in the Gulf of Thailand and were subjected to similar environmental pressures. In addition, spatial overlap between *A. ebracteatus* and *A. tetraploideus* could have facilitated post-formation gene flow or backcrossing, potentially contributing to the lower divergence observed between SG2 and its diploid progenitor.

To support the geographic component of this hypothesis, SNP data from 90 accessions collected across 14 sampling sites were analyzed using population structure analysis. Consistent with their geographic distribution, the analysis identified two distinct genetic clusters, separating populations from the Gulf of Thailand and the Andaman coast in all *Acanthus* species (Fig. 5B). In *A. ilicifolius*, the single sample from the Gulf side was assigned to the blue cluster (the Gulf of Thailand cluster), while all individuals from Andaman provinces were consistently assigned to the orange cluster (the Andaman coast cluster) with little admixture. Similarly, in *A. ebracteatus*, individuals from Gulf provinces were uniformly assigned to the blue cluster, while only a few samples from the Andaman side exhibited partial Andaman ancestry.

Nearly all *A. tetraploideus* samples were assigned to the Andaman cluster, with only one sample from Satun (STN-At-01) was fully assigned to the Gulf of Thailand cluster. These results indicated a strong east–west genetic separation.

The timing of the hybridization event leading to allotetraploid *A. tetraploideus* was estimated from transposable element (TE) divergence profiles, following the framework described in [47,48]. Divergence profiles of TE sequences from SG1 and SG2 were compared, with the values corresponding to progenitor divergence and subsequent genome merger identified at 27.3% and 6.3%, respectively (Supplementary Fig. S1). Applying the same calibration framework used in Ks-based dating (see Methods), these divergence values were scaled against the estimated progenitor divergence time of 3-8 Mya, yielding an estimated genome merger (hybridization) time of 0.7-1.8 Mya.

6. Protein family analysis

To enhance our understanding of the evolution of *Acanthus* species, protein family expansion and contraction were analyzed. Protein families were defined as expanded when they contained more protein members in a given species (leaf node) or ancestor (internal node) than in their most recent common ancestor (MRCA) and contracted when the reverse pattern was observed. Using this framework, numerous cases of gene family expansion and contraction across *A. marina*, *A. paniculata*, *S. cusia*, *A. ebracteatus*, *A. ilicifolius*, and the two subgenomes of *A. tetraploideus* (SG1 and SG2) were identified (Supplementary Table S19).

Several families were expanded in the MRCA of *Acanthus* species and contracted in the MRCA of *A. paniculata* and *S. cusia*. Many of these families were associated with root development. For example, families OG0001555 and OG0000052 were exclusively expanded within *Acanthus*. OG0001555 contained NFD6/NOXY2-like proteins, which were associated with lateral root development, while OG0000052 included MYB36 transcription factors, essential for

Casparian strip formation. MYB36 was linked (via STRING co-occurrence) to Protein MIZU-KUSSEI 1 (Ai1gPKTg3706.t1) in OG0006410, which was associated with hydrotropism gene ontology (GO) term (GO:001027), suggesting a potential functional network involved in water-responsive root development. An additional family that could participate in Casparian strip formation was family OG0000343, which contained dirigent proteins. The protein counts in this family were higher in *Acanthus* species and Am than in Ap and Sc. In contrast, another root-related family (OG0001760), containing DEEPER ROOTING 1, showed reduced representation in all *Acanthus* species compared to other members of the Acanthaceae clade. The contraction of this family might reflect an evolutionary shift in *Acanthus* toward shallower root architectures, potentially as an adaptation to anoxic, waterlogged soils and surface-level substrate anchorage typical of intertidal environments.

Some families were expanded in the MRCA of *A. paniculata* and *S. cusia* but contracted in the broader Acanthaceae MRCAs. Many of these were involved in terpenoid biosynthesis. Their expansion seemed mainly driven by increased protein numbers in *S. cusia*, while other species maintained relatively stable counts. Some families also showed high variability across species. For instance, OG0000029 (germacrene synthases) exhibited two- to three-fold higher copy numbers in *A. paniculata* and *S. cusia* than in *A. marina*, *A. ilicifolius*, *A. ebracteatus*, SG1, and SG2 (Table 1). Family OG0000080 (beta-amyrin monooxygenases) displayed a gradual contraction across the phylogeny, with the fewest copies observed in both SG1 and SG2 of *A. tetraploideus*. These shifts implied that *Acanthus* species may have reduced their reliance on specific triterpenoid pathways relative to other Acanthaceae.

Several protein families exhibited opposing patterns of expansion and contraction between the Ai–SG1 and Ae–SG2 lineages. For instance, families OG0000012 and OG0000227, encoding probable xyloglucan endotransglucosylase/hydrolase (XTH) and putative pectinesterase/pectinesterase inhibitors (PMEI), were expanded in the MRCA of *A. ilicifolius* and

SG1 but contracted in *A. ebracteatus* and SG2. A similar trend was observed for families OG0010398 and OG0000848, both containing chalcone synthase in the flavonoid biosynthesis pathway, which were expanded in *A. ilicifolius* but contracted in *A. ebracteatus*. Likewise, the family of endodermis-specific peroxidase 64 (OG0002958) was expanded in SG1 but contracted in Ae and SG2. Family OG0000840, encoding mechanosensitive ion channel proteins, was also contracted in *A. ebracteatus*. Conversely, family OG0013347, comprising aquaporin PIP2-7 members, underwent significant expansion in the MRCA of *A. ebracteatus* and SG2, with the highest number of copies observed in *A. ebracteatus*. Family OG0000188 of WIP-type zinc finger proteins was expanded in Ae, SG2, and Am, but had lower copy numbers in Ap, Sc, Ai, and SG1. These contrasting patterns highlighted divergent evolutionary pressures acting on the two parental lineages and their respective contributions to the *A. tetraploideus* genome.

To complement the phylogenetic expansion and contraction analysis, we applied machine learning to identify protein families with markedly different member counts in *Acanthus* compared to other taxa. Several families exhibited Acanthus-specific abundance patterns. Examples were OG0003440 (PHD finger protein Alfin1), OG0004372 and OG0012170 (A20/AN1 stress-associated proteins), and OG0009241 and OG0017721 (homeobox-leucine zipper protein ANTHOCYANINLESS 2), associating with salt, osmotic, or water deprivation responses. They appeared specific to the Acanthaceae clade (except OG0003440) and were more abundant in *Acanthus* species than in all others studied. Family OG0000866 (Rho GDP-dissociation inhibitor 1), involved in root epidermal cell differentiation, was found in all species but was most abundant in *Acanthus*. Finally, families OG0006911 and OG0014066, containing ferruginol synthases, were uniquely present in *Acanthus*. Ferruginol is a diterpene phenol with antibacterial, antitumor, and antimalarial activities [65].

Table 1 Comparative analysis of gene family expansion and contraction in *Acanthus* and related Acanthaceae species

Functional Group	Family ID	Representative Protein	Pattern in Acanthus	Pattern in Other Species
Root Development & Casparian Strip	OG0001555	NFD6/NOXY2-like	Expanded in Acanthus MRCA	Contracted in Ap/Sc
	OG0000052	MYB36	Expanded in Acanthus MRCA	Contracted in Ap/Sc
	OG0000343	Dirigent protein	Higher in Acanthus and Am	Lower in Ap/Sc
	OG0002958	Peroxidase 64	Expanded in SG1	Contracted in Ae/SG2
	OG0001760	Deeper Rooting 1	Lower in Acanthus	Higher in other Acanthaceae
	OG0009241	Homeobox-leucine zipper ANL2	Higher in Acanthus	Lower in others
	OG0017721	Homeobox-leucine zipper ANL2	Higher in Acanthus	Lower in others
	OG0000866	Rho GDP-dissociation inhibitor 1	Highest in Acanthus	Present in all
Osmotic Stress & Water Transport	OG0000188	WIP-type Zinc finger	Expanded in Ae/SG2/Am	Lower in Ai/SG1/Ap/Sc
	OG0013347	Aquaporin PIP2-7	Expanded in Ae/SG2	Not expanded in others
	OG0000840	Mechanosensitive ion channel	Contracted in Ae	Present in others
	OG0004372	A20/AN1 stress protein	Higher in Acanthus	Lower in others
	OG0012170	A20/AN1 stress protein	Higher in Acanthus	Lower in others
Cell Wall Remodeling	OG0000012	Xyloglucan endotransglucosylase	Expanded in Ai/SG1	Contracted in Ae/SG2
	OG0000227	Pectinesterase inhibitor	Expanded in Ai/SG1	Contracted in Ae/SG2
Secondary Metabolism (Flavonoids & Terpenoids)	OG0010398	Chalcone synthase	Expanded in Ai	Contracted in Ae
	OG0000848	Chalcone synthase	Expanded in Ai	Contracted in Ae
	OG0000029	Germacrene synthase	Fluctuated	Higher in Ap/Sc
	OG0000080	Beta-amyrin monooxygenase	Contracted in Acanthus	Higher in Am
	OG0006911	Ferruginol synthase	Unique to Acanthus	Absent in others
	OG0014066	Ferruginol synthase	Unique to Acanthus	Absent in others
Stress/Signaling Regulators	OG0003440	PHD finger Alfin1	Higher in Acanthus	Lower in others
Unknown / Hypothetical	OG0000061	Hypothetical protein	Very high in Acanthus	Low in others

Discussion

Our genome assemblies confirm that *A. tetraploideus* is an allotetraploid derived from *A. ilicifolius* (SG1) and *A. ebracteatus* (SG2). The strong synteny between subgenomes and their progenitors, together with the high proportion of duplicated genes, underscores a relatively recent hybridization and whole-genome duplication event. Based on our findings, we propose that the evolutionary history of *Acanthus* was collectively shaped by genome duplication, ancient divergence, subsequent lineage-specific evolution, and recent hybridization.

The evolutionary trajectory of the genus began with the divergence of the *Acanthus* lineage from the ancestor of the terrestrial species *A. paniculata* and *S. cusia* approximately 33–57 Mya and was subsequently shaped by a WGD event dated to about 21–56 Mya. Both events coincided with early–middle Eocene (~50–40 Mya) climatic upheavals, which created unstable coastal habitats [66]. These stresses likely promoted ecological separation from terrestrial relatives, while WGD provided genetic redundancy and flexibility to adapt to salinity fluctuations, tidal inundation, and anoxic soils [67,68], similar to patterns reported in Malpighiales [67]. Following this duplication, *Acanthus* underwent lineage-specific remodeling of root-associated gene families. The expansions of *MYB36*, *DIR10-like*, and *PER64* might reinforced Casparian strip barriers [69–71], while *NOXY2-like*, *ANL2*, *RhoGDI1*, *Alfin1*, and *SAP* could support root development and stress tolerance [72–76] . In contrast, contraction of *DRO1* suggests a shift toward shallow, laterally spreading root systems suited to sediment-rich mangrove shorelines [58]. Contractions in terpenoid biosynthesis gene families, compared with the terrestrial medicinal plants *A. paniculata* [77] and *S. cusia* [78], further indicate a shift in secondary metabolism associated with mangrove adaptation. Together, these changes highlight how WGD and subsequent gene family evolution enabled *Acanthus* to persist and specialize in dynamic coastal environments.

Later, during the Late Miocene (5-10 Mya), *A. ilicifolius* and *A. ebracteatus* diverged under intensified monsoons, fluctuating sea levels, and tidal reorganization that reshaped Southeast Asian coastlines [79–81], which likely fragmented mangrove populations, promoting dispersal and allopatric divergence. This scenario is supported by modeled tidal ranges during the Messinian (~6 Mya) that align with the present-day distribution of *A. ilicifolius* along the open coasts of the Andaman Sea and *A. ebracteatus* in the more sheltered estuaries of the Gulf of Thailand [1,79]. The distribution of these species is further supported by genetic separation of populations from the east and west coasts, which appears to be influenced by environmental conditions such as salinity and hydrology [82]. Genomic comparisons revealed greater structural and sequence variation in *A. ebracteatus* than in *A. ilicifolius* [1], indicating independent evolutionary trajectories. Gene family expansions were more pronounced in *A. ilicifolius* and SG1, particularly in root-related families such as *PER64* and *XTH23*, which reinforced Casparian strip function and supported lateral root adaptation to salinity [83,84]. An expansion of *CHS*, the first enzyme in flavonoid biosynthesis and a contributor to stress tolerance [85], also corresponded with the high flavonoid and phenolic content of *A. ilicifolius* [3]. Because *CHS* and other flavonoid biosynthesis genes influence flower pigmentation [86], this expansion may also underlie differences in floral coloration—violet in *A. ilicifolius*, white in *A. ebracteatus*, and pale-violet in *A. tetraploideus*. Together, these lineage-specific genomic patterns and the east–west coastal separation indicate that *A. ilicifolius* and *A. ebracteatus* were shaped by contrasting ecological pressures.

Subsequently, glacial–interglacial cycles and sea-level fluctuations during the early Pleistocene (~1.3–1.5 Mya) repeatedly isolated and reconnected mangrove habitats [87–89], providing opportunities for secondary contact between *A. ilicifolius* and *A. ebracteatus* and ultimately leading to the formation of the allotetraploid *A. tetraploideus*. The contrasting genomic architectures of the two progenitor species may have played a role in the origin of the

allotetraploid. The smaller, more structurally dynamic genome of *A. ebracteatus* likely facilitated hybrid compatibility with the larger genome of *A. ilicifolius* [90]. In *A. tetraploideus*, transcriptomic analyses revealed a consistent expression bias toward SG1, reflecting subgenome dominance, whereas SG2 contributed lineage-specific and novel expression patterns [4]. Such asymmetric behavior mirrors that observed in other allopolyploids [91,92], where one subgenome maintains regulatory integrity while the other enhances adaptive potential. Together, these complementary roles may have underpinned both the successful formation and ecological expansion of *A. tetraploideus*, enabling it to thrive across diverse coastal habitats inherited from its progenitor lineages. Additionally, unlike sterile hybrids in other mangrove genera such as *Rhizophora* and *Avicennia* [93], *A. tetraploideus* is capable of producing viable seeds in addition to clonal propagation [1]. Subgenomes of *A. tetraploideus* remain largely intact and collinear with their progenitors, and limited homeologous recombination likely supports disomic pairing and balanced gamete formation [94]. Thus, the integration of adaptive genes from ecologically divergent parents, transcriptomic reprogramming, and the capacity for both sexual and asexual reproduction underpins the evolutionary success of *A. tetraploideus* in coastal ecosystems.

In summary, the interplay of genome duplication, ecological divergence, and hybridization highlights the evolutionary history of *Acanthus*. Divergence from terrestrial relatives with a WGD during the Eocene, the split between *A. ilicifolius* and *A. ebracteatus* in the Miocene, and the formation of the allotetraploid *A. tetraploideus* through secondary contact during the Pleistocene together define the key stages of *Acanthus* evolution. This sequence also illustrates how climatic change and genomic processes shaped mangrove diversification. Future studies on stress-related gene families and subgenome interactions will clarify how polyploidy and hybridization contribute to resilience in coastal ecosystems.

Abbreviations

BUSCO: Benchmarking Universal Single-Copy Orthologs, GO: Gene Ontology, Hi-C: High-throughput Chromosome Conformation Capture, Ks: Synonymous Substitution Rate, LTR: Long Terminal Repeat, MRCA: Most Recent Common Ancestor, Mya: Million Years Ago, N50: Contig or Scaffold Length at 50% of Genome Assembly, OG: Orthogroup, stLFR: Single-Tube Long Fragment Read, TE: Transposable Element, WGD: Whole-Genome Duplication.

Data Availability Statement

The assembled genome sequences of *A. tetraploideus*, *A. ilicifolius*, and *A. ebracteatus* were deposited in the NCBI database under BioProject PRJNA1102049, PRJNA1275650, and PRJNA1111239, respectively.

Acknowledgments

We thank the research team from the Mangrove Forest Research Center for their sample collection. The authors would also like to acknowledge funding from the National Science and Technology Development Agency, Thailand (project ID: P2351523).

Competing interests

The authors declare that they have no competing interests.

References

1. Feng H, Banerjee AK, Guo W, Yuan Y, Duan F, Ng WL, et al.. Origin and evolution of a new tetraploid mangrove species in an intertidal zone. *Plant Diversity*. 2024; doi: 10.1016/j.pld.2024.04.007.

2. Yang Y, Yang S, Li J, Deng Y, Zhang Z, Xu S, et al.. Transcriptome analysis of the Holly mangrove Acanthus ilicifolius and its terrestrial relative, Acanthus leucostachyus, provides insights into adaptation to intertidal zones. *BMC Genomics*. 2015; doi: 10.1186/s12864-015-1813-9.

3. Matos P, Batista MT, Figueirinha A. A review of the ethnomedicinal uses, chemistry, and pharmacological properties of the genus Acanthus (Acanthaceae). *Journal of Ethnopharmacology*. 2022; doi: 10.1016/j.jep.2022.115271.

4. Guo W, Banerjee AK, Feng H, Ng WL, Wu H, Li W, et al.. Recent allopolyploidization and transcriptomic asymmetry in the mangrove shrub Acanthus tetraploideus. *BMC Genomics*. 2025; doi: 10.1186/s12864-025-11557-2.

5. Kong W, Wang Y, Zhang S, Yu J, Zhang X. Recent Advances in Assembly of Complex Plant Genomes. *Genomics, Proteomics & Bioinformatics*. 2023; doi: 10.1016/j.gpb.2023.04.004.

6. Natarajan P, Murugesan AK, Govindan G, Gopalakrishnan A, Kumar R, Duraisamy P, et al.. A reference-grade genome identifies salt-tolerance genes from the salt-secreting mangrove species Avicennia marina. *Commun Biol*. Nature Publishing Group; 2021; doi: 10.1038/s42003-021-02384-8.

7. Pootakham W, Naktang C, Sonthirod C, Kongkachana W, Narong N, Sangsrakru D, et al.. Chromosome-level genome assembly of Indian mangrove (Ceriops tagal) revealed a genome-wide duplication event predating the divergence of Rhizophoraceae mangrove species. *The Plant Genome*. 2022; doi: 10.1002/tpg2.20217.

8. Shearman JR, Naktang C, Sonthirod C, Kongkachana W, U-thoomporn S, Jomchai N, et al.. Assembly of a hybrid mangrove, *Bruguiera hainesii*, and its two ancestral contributors, *Bruguiera cylindrica* and *Bruguiera gymnorhiza*. *Genomics*. 2022; doi: 10.1016/j.ygeno.2022.110382.

9. Pootakham W, Nawae W, Naktang C, Sonthirod C, Yoocha T, Kongkachana W, et al.. A chromosome-scale assembly of the black gram (Vigna mungo) genome. *Molecular Ecology Resources*. 2021; doi: 10.1111/1755-0998.13243.

10. Pootakham W, Naktang C, Sonthirod C, Kongkachana W, Yoocha T, Jomchai N, et al.. De Novo Reference Assembly of the Upriver Orange Mangrove (Bruguiera sexangula) Genome. *Genome Biology and Evolution*. 2022; doi: 10.1093/gbe/evac025.

11. Cheng H, Concepcion GT, Feng X, Zhang H, Li H. Haplotype-resolved de novo assembly using phased assembly graphs with hifiasm. *Nat Methods*. Nature Publishing Group; 2021; doi: 10.1038/s41592-020-01056-5.

12. Dovetail: Welcome to the Dovetail® Linked-Read Analysis Page — Dovetail Analysis 0.1 documentation. https://dovetail-analysis.readthedocs.io/en/latest/ Accessed 2025 Nov 5.

13. Zhou C, McCarthy SA, Durbin R. YaHS: yet another Hi-C scaffolding tool. *Bioinformatics*. 2023; doi: 10.1093/bioinformatics/btac808.

14. Manni M, Berkeley MR, Seppey M, Zdobnov EM. BUSCO: Assessing Genomic Data Quality and Beyond. *Current Protocols*. 2021; doi: 10.1002/cpz1.323.

15. BGI-biotools: BGI-biotools. https://github.com/BGI-biotools/stLFRdenovo (2024). Accessed 2025 Nov 5.

16. Cabanettes F, Klopp C. D-GENIES: dot plot large genomes in an interactive, efficient and simple way. *PeerJ*. PeerJ Inc.; 2018; doi: 10.7717/peerj.4958.

17. Alonge M, Lebeigle L, Kirsche M, Jenike K, Ou S, Aganezov S, et al.. Automated assembly scaffolding using RagTag elevates a new tomato system for high-throughput genome editing. *Genome Biology*. 2022; doi: 10.1186/s13059-022-02823-7.

18. Brůna T, Hoff KJ, Lomsadze A, Stanke M, Borodovsky M. BRAKER2: automatic eukaryotic genome annotation with GeneMark-EP+ and AUGUSTUS supported by a protein database. *NAR Genomics and Bioinformatics*. 2021; doi: 10.1093/nargab/lqaa108.

19. Kuznetsov D, Tegenfeldt F, Manni M, Seppey M, Berkeley M, Kriventseva EV, et al.. OrthoDB v11: annotation of orthologs in the widest sampling of organismal diversity. *Nucleic Acids Research*. 2023; doi: 10.1093/nar/gkac998.

20. Gurevich A, Saveliev V, Vyahhi N, Tesler G. QUAST: quality assessment tool for genome assemblies. *Bioinformatics*. 2013; doi: 10.1093/bioinformatics/btt086.

21. Jia K-H, Wang Z-X, Wang L, Li G-Y, Zhang W, Wang X-L, et al.. SubPhaser: a robust allopolyploid subgenome phasing method based on subgenome-specific k-mers. *New Phytologist*. 2022; doi: 10.1111/nph.18173.

22. Wang Y, Tang H, Wang X, Sun Y, Joseph PV, Paterson AH. Detection of colinear blocks and synteny and evolutionary analyses based on utilization of MCScanX. *Nat Protoc*. Nature Publishing Group; 2024; doi: 10.1038/s41596-024-00968-2.

23. Tang H, Krishnakumar V, Zeng X, Xu Z, Taranto A, Lomas JS, et al.. JCVI: A versatile toolkit for comparative genomics analysis. *iMeta*. 2024; doi: 10.1002/imt2.211.

24. Krzywinski M, Schein J, Birol İ, Connors J, Gascoyne R, Horsman D, et al.. Circos: An information aesthetic for comparative genomics. *Genome Res*. 2009; doi: 10.1101/gr.092759.109.

25. Sun P, Jiao B, Yang Y, Shan L, Li T, Li X, et al.. WGDI: A user-friendly toolkit for evolutionary analyses of whole-genome duplications and ancestral karyotypes. *Molecular Plant*. 2022; doi: 10.1016/j.molp.2022.10.018.

26. Edgar RC. MUSCLE: a multiple sequence alignment method with reduced time and space complexity. *BMC Bioinformatics*. 2004; doi: 10.1186/1471-2105-5-113.

27. Yang Z. PAML 4: Phylogenetic Analysis by Maximum Likelihood. *Mol Biol Evol*. Oxford Academic; 2007; doi: 10.1093/molbev/msm088.

28. Wu W, Feng X, Wang N, Shao S, Liu M, Si F, et al.. Genomic analysis of Nypa fruticans elucidates its intertidal adaptations and early palm evolution. *Journal of Integrative Plant Biology*. 2024; doi: 10.1111/jipb.13625.

29. Xie W, Guo Z, Wang J, He Z, Li Y, Feng X, et al.. Evolution of woody plants to the land-sea interface – The atypical genomic features of mangroves with atypical phenotypic adaptation. *Molecular Ecology*. 2023; doi: 10.1111/mec.16587.

30. Feng X, Chen Q, Wu W, Wang J, Li G, Xu S, et al.. Genomic evidence for rediploidization and adaptive evolution following the whole-genome triplication. *Nat Commun*. Nature Publishing Group; 2024; doi: 10.1038/s41467-024-46080-7.

31. Pootakham W, Sonthirod C, Naktang C, Kongkachana W, Sangsrakru D, U-thoomporn S, et al.. A chromosome-scale reference genome assembly of yellow mangrove (Bruguiera parviflora) reveals a whole genome duplication event associated with the Rhizophoraceae lineage. *Molecular Ecology Resources*. 2022; doi: 10.1111/1755-0998.13587.

32. Ruang-areerate P, Naktang C, Kongkachana W, Sangsrakru D, Narong N, Maknual C, et al.. Assessment of the Genetic Diversity and Population Structure of Rhizophora apiculata Blume (Rhizophoraceae) in Thailand. *Biology*. Multidisciplinary Digital Publishing Institute; 2022; doi: 10.3390/biology11101449.

33. Hu M-J, Sun W-H, Tsai W-C, Xiang S, Lai X-K, Chen D-Q, et al.. Chromosome-scale assembly of the Kandelia obovata genome. *Hortic Res*. Nature Publishing Group; 2020; doi: 10.1038/s41438-020-0300-x.

34. Ma D, Guo Z, Ding Q, Zhao Z, Shen Z, Wei M, et al.. Chromosome-level assembly of the mangrove plant Aegiceras corniculatum genome generated through Illumina, PacBio and Hi-C sequencing technologies. *Molecular Ecology Resources*. 2021; doi: 10.1111/1755-0998.13347.

35. Ma L, Dong C, Song C, Wang X, Zheng X, Niu Y, et al.. De novo genome assembly of the potent medicinal plant Rehmannia glutinosa using nanopore technology. *Computational and Structural Biotechnology Journal*. Elsevier; 2021; doi: 10.1016/j.csbj.2021.07.006.

36. Ma D, Ding Q, Guo Z, Xu C, Liang P, Zhao Z, et al.. The genome of a mangrove plant, Avicennia marina, provides insights into adaptation to coastal intertidal habitats. *Planta*. 2022; doi: 10.1007/s00425-022-03916-0.

37. Hu Y, Ma D, Ning S, Ye Q, Zhao X, Ding Q, et al.. High-Quality Genome of the Medicinal Plant Strobilanthes cusia Provides Insights Into the Biosynthesis of Indole Alkaloids. *Front Plant Sci*. Frontiers; 2021; doi: 10.3389/fpls.2021.742420.

38. Liang Y, Chen S, Wei K, Yang Z, Duan S, Du Y, et al.. Chromosome Level Genome Assembly of Andrographis paniculata. *Front Genet*. Frontiers; 2020; doi: 10.3389/fgene.2020.00701.

39. Emms DM, Kelly S. OrthoFinder: phylogenetic orthology inference for comparative genomics. *Genome Biology*. 2019; doi: 10.1186/s13059-019-1832-y.

40. Capella-Gutiérrez S, Silla-Martínez JM, Gabaldón T. trimAl: a tool for automated alignment trimming in large-scale phylogenetic analyses. *Bioinformatics*. 2009; doi: 10.1093/bioinformatics/btp348.

41. Kozlov AM, Darriba D, Flouri T, Morel B, Stamatakis A. RAxML-NG: a fast, scalable and user-friendly tool for maximum likelihood phylogenetic inference. *Bioinformatics*. 2019; doi: 10.1093/bioinformatics/btz305.

42. Darriba D, Posada D, Kozlov AM, Stamatakis A, Morel B, Flouri T. ModelTest-NG: A New and Scalable Tool for the Selection of DNA and Protein Evolutionary Models. *Molecular Biology and Evolution*. 2020; doi: 10.1093/molbev/msz189.

43. Kumar S, Suleski M, Craig JM, Kasprowicz AE, Sanderford M, Li M, et al.. TimeTree 5: An Expanded Resource for Species Divergence Times. *Mol Biol Evol*. 2022; doi: 10.1093/molbev/msac174.

44. Sensalari C, Maere S, Lohaus R. ksrates: positioning whole-genome duplications relative to speciation events in KS distributions. *Bioinformatics*. 2022; doi: 10.1093/bioinformatics/btab602.

45. Padgitt-Cobb LK, Pitra NJ, Matthews PD, Henning JA, Hendrix DA. An improved assembly of the “Cascade” hop (Humulus lupulus) genome uncovers signatures of molecular evolution and refines time of divergence estimates for the Cannabaceae family. *Hortic Res*. 2023; doi: 10.1093/hr/uhac281.

46. Kimura M. A simple method for estimating evolutionary rates of base substitutions through comparative studies of nucleotide sequences. *J Mol Evol*. 1980; doi: 10.1007/BF01731581.

47. Xu P, Xu J, Liu G, Chen L, Zhou Z, Peng W, et al.. The allotetraploid origin and asymmetrical genome evolution of the common carp Cyprinus carpio. *Nat Commun*. Nature Publishing Group; 2019; doi: 10.1038/s41467-019-12644-1.

48. Wang Y, Li Y, Wu W, Shao S, Fang Q, Xu S, et al.. The evolution history of an allotetraploid mangrove tree analysed with a new tool Allo4D. *Plant Biotechnology Journal*. 2024; doi: 10.1111/pbi.14281.

49. Mendes FK, Vanderpool D, Fulton B, Hahn MW. CAFE 5 models variation in evolutionary rates among gene families. *Bioinformatics*. 2020; doi: 10.1093/bioinformatics/btaa1022.

50. Revell LJ. phytools 2.0: an updated R ecosystem for phylogenetic comparative methods (and other things). *PeerJ*. PeerJ Inc.; 2024; doi: 10.7717/peerj.16505.

51. Li H: lh3/bwa. https://github.com/lh3/bwa (2025). Accessed 2025 Nov 5.

52. Poplin R, Ruano-Rubio V, DePristo MA, Fennell TJ, Carneiro MO, Auwera GAV der, et al.. Scaling accurate genetic variant discovery to tens of thousands of samples. bioRxiv;

53. Pritchard JK, Stephens M, Donnelly P. Inference of Population Structure Using Multilocus Genotype Data. *Genetics*. 2000; doi: 10.1093/genetics/155.2.945.

54. Earl DA, vonHoldt BM. STRUCTURE HARVESTER: a website and program for visualizing STRUCTURE output and implementing the Evanno method. *Conservation Genet Resour*. 2012; doi: 10.1007/s12686-011-9548-7.

55. Evanno G, Regnaut S, Goudet J. Detecting the number of clusters of individuals using the software structure: a simulation study. *Molecular Ecology*. 2005; doi: 10.1111/j.1365-294X.2005.02553.x.

56. Jakobsson M, Rosenberg NA. CLUMPP: a cluster matching and permutation program for dealing with label switching and multimodality in analysis of population structure. *Bioinformatics*. 2007; doi: 10.1093/bioinformatics/btm233.

57. Chen B-Z, Li D-W, Luo K-Y, Jiu S-T, Dong X, Wang W-B, et al.. Chromosome-level assembly of Lindenbergia philippensis and comparative genomic analyses shed light on genome evolution in Lamiales. *Front Plant Sci*. Frontiers; 2024; doi: 10.3389/fpls.2024.1444234.

58. Shumate A, Salzberg SL. Liftoff: accurate mapping of gene annotations. *Bioinformatics*. 2021; doi: 10.1093/bioinformatics/btaa1016.

59. Qiao X, Li Q, Yin H, Qi K, Li L, Wang R, et al.. Gene duplication and evolution in recurring polyploidization–diploidization cycles in plants. *Genome Biology*. 2019; doi: 10.1186/s13059-019-1650-2.

60. Shen Z, Li W, Li Y, Liu M, Cao H, Provart N, et al.. The red flower wintersweet genome provides insights into the evolution of magnoliids and the molecular mechanism for tepal color development. *The Plant Journal*. 2021; doi: 10.1111/tpj.15533.

61. Zhang B, Yao X, Chen H, Lu L. High-quality chromosome-level genome assembly of *Litsea coreana* L. provides insights into Magnoliids evolution and flavonoid biosynthesis. *Genomics*. 2022; doi: 10.1016/j.ygeno.2022.110394.

62. Xu L, Wang X-R, Sun K, Yu T, Xu J-H, Ding P-X, et al.. The complete chloroplast genome of Acanthus ilicifolius, an excellent mangrove plant. *Mitochondrial DNA Part B*. Taylor & Francis; 2021; doi: 10.1080/23802359.2021.1884022.

63. He Z, Feng X, Chen Q, Li L, Li S, Han K, et al.. Evolution of coastal forests based on a full set of mangrove genomes. *Nat Ecol Evol*. Nature Publishing Group; 2022; doi: 10.1038/s41559-022-01744-9.

64. Pootakham W, Sonthirod C, Naktang C, Kongkachana W, U-thoomporn S, Phetchawang P, et al.. A de novo reference assembly of the yellow mangrove Ceriops zippeliana genome. *G3 Genes|Genomes|Genetics*. 2022; doi: 10.1093/g3journal/jkac025.

65. González-Cardenete MA, Rivas F, Basset R, Stadler M, Hering S, Padrón JM, et al.. Biological Profiling of Semisynthetic C19-Functionalized Ferruginol and Sugiol Analogues. *Antibiotics*. Multidisciplinary Digital Publishing Institute; 2021; doi: 10.3390/antibiotics10020184.

66. Srivastava J, Prasad V. Evolution and paleobiogeography of mangroves. *Marine Ecology*. 2019; doi: 10.1111/maec.12571.

67. Cai L, Xi Z, Amorim AM, Sugumaran M, Rest JS, Liu L, et al.. Widespread ancient whole-genome duplications in Malpighiales coincide with Eocene global climatic upheaval. *New Phytologist*. 2019; doi: 10.1111/nph.15357.

68. Van de Peer Y, Mizrachi E, Marchal K. The evolutionary significance of polyploidy. *Nat Rev Genet*. Nature Publishing Group; 2017; doi: 10.1038/nrg.2017.26.

69. Chen T, Cai ,Xia, Wu ,Xiaoqin, Karahara ,Ichirou, Schreiber ,Lucas, and Lin J. Casparian strip development and its potential function in salt tolerance. *Plant Signaling & Behavior*. Taylor & Francis; 2011; doi: 10.4161/psb.6.10.17054.

70. Kamiya T, Borghi M, Wang P, Danku JMC, Kalmbach L, Hosmani PS, et al.. The MYB36 transcription factor orchestrates Casparian strip formation. *Proceedings of the National*

Academy of Sciences. Proceedings of the National Academy of Sciences; 2015; doi: 10.1073/pnas.1507691112.

71. Gao Y-Q, Huang J-Q, Reyt G, Song T, Love A, Tiemessen D, et al.. A dirigent protein complex directs lignin polymerization and assembly of the root diffusion barrier. *Science*. American Association for the Advancement of Science; 2023; doi: 10.1126/science.adi5032.

72. Winicov I. Alfin1 transcription factor overexpression enhances plant root growth under normal and saline conditions and improves salt tolerance in alfalfa. *Planta*. 2000; doi: 10.1007/PL00008150.

73. Liu X, Yu X, Shi Y, Ma L, Fu Y, Guo Y. Phosphorylation of RhoGDI1, a Rho GDP dissociation inhibitor, regulates root hair development in Arabidopsis under salt stress. *Proceedings of the National Academy of Sciences*. Proceedings of the National Academy of Sciences; 2023; doi: 10.1073/pnas.2217957120.

74. Vellosillo T, Martínez M, López MA, Vicente J, Cascón T, Dolan L, et al.. Oxylipins Produced by the 9-Lipoxygenase Pathway in Arabidopsis Regulate Lateral Root Development and Defense Responses through a Specific Signaling Cascade. *The Plant Cell*. 2007; doi: 10.1105/tpc.106.046052.

75. Roy R, Bassham DC. Root growth movements: Waving and skewing. *Plant Science*. 2014; doi: 10.1016/j.plantsci.2014.01.007.

76. Porat A, Tekinalp A, Bhosale Y, Gazzola M, Meroz Y. On the mechanical origins of waving, coiling and skewing in Arabidopsis thaliana roots. *Proceedings of the National Academy of Sciences*. Proceedings of the National Academy of Sciences; 2024; doi: 10.1073/pnas.2312761121.

77. Sun W, Leng L, Yin Q, Xu M, Huang M, Xu Z, et al.. The genome of the medicinal plant Andrographis paniculata provides insight into the biosynthesis of the bioactive diterpenoid neoandrographolide. *The Plant Journal*. John Wiley & Sons, Ltd; 2019; doi: 10.1111/tpj.14162.

78. Hu Y, Ma D, Ning S, Ye Q, Zhao X, Ding Q, et al.. High-Quality Genome of the Medicinal Plant Strobilanthes cusia Provides Insights Into the Biosynthesis of Indole Alkaloids. *Front Plant Sci*. Frontiers; 2021; doi: 10.3389/fpls.2021.742420.

79. Collins DS, Avdis A, Allison PA, Johnson HD, Hill J, Piggott MD, et al.. Tidal dynamics and mangrove carbon sequestration during the Oligo–Miocene in the South China Sea. *Nat Commun*. Nature Publishing Group; 2017; doi: 10.1038/ncomms15698.

80. Holbourn AE, Kuhnt W, Clemens SC, Kochhann KGD, Jöhnck J, Lübbers J, et al.. Late Miocene climate cooling and intensification of southeast Asian winter monsoon. *Nat Commun*. Nature Publishing Group; 2018; doi: 10.1038/s41467-018-03950-1.

81. Herbert TD, Lawrence KT, Tzanova A, Peterson LC, Caballero-Gill R, Kelly CS. Late Miocene global cooling and the rise of modern ecosystems. *Nature Geosci*. Nature Publishing Group; 2016; doi: 10.1038/ngeo2813.

82. Aksornkoae S. Ecology and management of mangroves. IUCN;

83. Xu P, Fang S, Chen H, Cai W. The brassinosteroid-responsive xyloglucan endotransglucosylase/hydrolase 19 (XTH19) and XTH23 genes are involved in lateral root development under salt stress in Arabidopsis. *The Plant Journal*. 2020; doi: 10.1111/tpj.14905.

84. Lee Y, Rubio MC, Alassimone J, Geldner N. A Mechanism for Localized Lignin Deposition in the Endodermis. *Cell*. Elsevier; 2013; doi: 10.1016/j.cell.2013.02.045.

85. Lijuan C, Huiming G, Yi L, Hongmei C. Chalcone synthase EaCHS1 from Eupatorium adenophorum functions in salt stress tolerance in tobacco. *Plant Cell Rep*. 2015; doi: 10.1007/s00299-015-1751-7.

86. Wang J-L, Zhang W-D, Yang X-D, Zhao P-G, Wang X-Y, Zhao S-Y, et al.. Chromosome-level genome assembly of Pontederia cordata L. provides insights into its rapid adaptation and variation of flower colours. *DNA Res*. 2025; doi: 10.1093/dnares/dsaf002.

87. Li J, Yang Y, Chen Q, Fang L, He Z, Guo W, et al.. Pronounced genetic differentiation and recent secondary contact in the mangrove tree Lumnitzera racemosa revealed by population genomic analyses. *Sci Rep*. Nature Publishing Group; 2016; doi: 10.1038/srep29486.

88. Yang Y, Li J, Yang S, Li X, Fang L, Zhong C, et al.. Effects of Pleistocene sea-level fluctuations on mangrove population dynamics: a lesson from Sonneratia alba. *BMC Evolutionary Biology*. 2017; doi: 10.1186/s12862-016-0849-z.

89. Dumitru OA, Austermann J, Polyak VJ, Fornós JJ, Asmerom Y, Ginés J, et al.. Sea-level stands from the Western Mediterranean over the past 6.5 million years. *Sci Rep*. Nature Publishing Group; 2021; doi: 10.1038/s41598-020-80025-6.

90. BUREŠ P, WANG Y-F, HOROVÁ L, SUDA J. Genome Size Variation in Central European Species of Cirsium (Compositae) and their Natural Hybrids. *Annals of Botany*. 2004; doi: 10.1093/aob/mch151.

91. Cai X, Chang L, Zhang T, Chen H, Zhang L, Lin R, et al.. Impacts of allopolyploidization and structural variation on intraspecific diversification in Brassica rapa. *Genome Biology*. 2021; doi: 10.1186/s13059-021-02383-2.

92. Wang Z, Yang J, Cheng F, Li P, Xin X, Wang W, et al.. Subgenome dominance and its evolutionary implications in crop domestication and breeding. *Hortic Res*. 2022; doi: 10.1093/hr/uhac090.

93. Ragavan P, Zhou R, Ng WL, Rana TS, Mageswaran T, Mohan PM, et al.. Natural hybridization in mangroves – an overview. *Botanical Journal of the Linnean Society*. 2017; doi: 10.1093/botlinnean/box053.

94. Soltis PS, Soltis DE. The role of genetic and genomic attributes in the success of polyploids. *Proceedings of the National Academy of Sciences*. Proceedings of the National Academy of Sciences; 2000; doi: 10.1073/pnas.97.13.7051.

Figure legends

Figure 1. Chromosome-scale genome assembly of *Acanthus tetraploideus*. (A) Photograph of *A. tetraploideus* showing its characteristic lanceolate, spiny, leathery leaves and pale violet flowers. (B) Hi-C contact matrix of the *A. tetraploideus* genome assembly showing strong intrachromosomal interactions and clear chromosome boundaries, confirming high-quality scaffolding of 48 chromosomes. (C) Circos plot displaying syntenic relationships between subgenomes SG1 (orange) and SG2 (light blue) of *A. tetraploideus*. The outermost ring represents 48 chromosomes (24 homeologous pairs). Inner tracks show gene density (blue), repeat content (yellow), and colored ribbons connecting syntenic blocks between homeologous chromosomes. (D) Sankey-style diagram illustrating genome-wide syntenic relationships between *A. tetraploideus* and its progenitor species, where SG1 (blue bars) corresponds to *A. ilicifolius* (orange bars) and SG2 (violet bars) corresponds to *A. ebracteatus* (green bars), indicating their respective parental contributions to the allotetraploid genome.

Figure 2. Ancestral karyotype reconstruction and chromosome evolution in *Acanthus tetraploideus*. (A) Alignment of *A. tetraploideus* chromosomes (columns) to 11 reconstructed ancestral protochromosomes (rows). Chromosomes belonging to subgenome SG1 are labeled in black (top) and shown with red and yellow alignment lines, while those of subgenome SG2 are labeled in blue and shown with green and blue alignment lines. Diagonal alignments indicate homeologous chromosome pairs derived from the same ancestral protochromosome. (B) Schematic model illustrating chromosome evolution in the *Acanthus* lineage. The ancestral karyotype (n = 11) underwent a whole-genome duplication (WGD), yielding a diploid genome with n = 22 chromosomes. Subsequent chromosomal fissions (see alignments between chromosome 1 with chromosomes 11 and 23, and between chromosome 10 with chromosomes 21 and 24) increased the haploid chromosome number to n = 24. Hybridization between two diploid progenitors with this karyotype led to the formation of the allotetraploid species *A. tetraploideus* (n = 48). Mixed-color bars in the final karyotype indicate chromosomal translocations and rearrangements.

Figure 3. Ks distributions showing speciation and WGD signals in the Acanthus lineage. (A) Ks distributions of paralogous and orthologous gene pairs among Acanthus species. Two major peaks correspond to a recent speciation event (Ks ≈ 0.05) and an older whole-genome duplication (WGD; Ks ≈ 0.35). (B) Ks distributions between and within subgenomes of *A. tetraploideus*. The SG1–SG2 comparison shows the same WGD peak as observed in the diploid progenitors, indicating that the WGD occurred before hybridization and subgenome divergence.

Figure 4. Subgenome-specific gene retention and phylogenetic relationships of duplicated genes on chromosome 20B. (A) Gene retention ratios along selected chromosomes of *A. tetraploideus*. Ratios represent the proportion of genes retained from each progenitor, ranging from 0 to 1. Red and yellow lines indicate genes retained from the two groups of WGD-derived scaffolds in *A. ilicifolius*, whereas green and blue lines represent genes inherited from *A. ebracteatus* (see matched colors in Fig. 2). Gene retention patterns across all *A. tetraploideus* chromosomes are shown in Supplementary Fig. S3.(B) Phylogenetic tree of a representative gene family containing multiple paralogous copies within the extended sequence of chromosome 20B (highlighted in pink). The paralogous gene copies in *A. tetraploideus* (subgenome SG2) are shown in bold black, and the orthologous gene in *A. ebracteatus* is shown in bold blue. Homologous genes from *L. racemosa* (Lra), *B. parviflora* (Bpa), *C. tagal* (Cta), *C. micranthum* (Cmmi), *O. europaea* (Oeu), *A. paniculata* (Apa), *S. cusia* (Scu), and *R. glutinosa* (Rgl) within the same gene family are also shown.

Figure 5. Divergence time and population structure of Acanthus species. (A) Maximum-likelihood phylogenetic tree showing divergence times and gene family evolution among 19 plant species, including mangrove and non-mangrove taxa. Numbers at each node indicate divergence time estimates (in million years ago) with 95% confidence intervals in brackets. Green and red numbers represent significantly expanded (+) and contracted (−) gene families, respectively. Orange circles indicate calibration points derived from the TimeTree database, and blue stars mark lineage-specific WGD events in *Avicennia* and *Acanthus*. Subgenomes (SG1 and SG2) of *A. tetraploideus* are shown separately. Distinct background shades denote the clade, order, and family of each analyzed species. (B) Population structure analysis of *A. ebracteatus* (n = 29), *A. tetraploideus* (n = 27), and *A. ilicifolius* (n = 36) based on SNP variation. Each vertical bar represents an individual, with blue and orange segments indicating ancestry

associated with Gulf of Thailand and Andaman coast populations, respectively. Gulf of Thailand populations included samples from Chumphon (CPN), Nakhon Si Thammarat (NST), Phatthalung (PLG), Phetchaburi (PBI), Samut Songkhram (SKM), Samut Sakhon (SKN), Surat Thani (SNI), and Trat (TRT), whereas Andaman coast populations included samples from Krabi (KBI), Phang Nga (PNG), Phuket (PKT), Ranong (RNG), Satun (STN), and Trang (TRG).

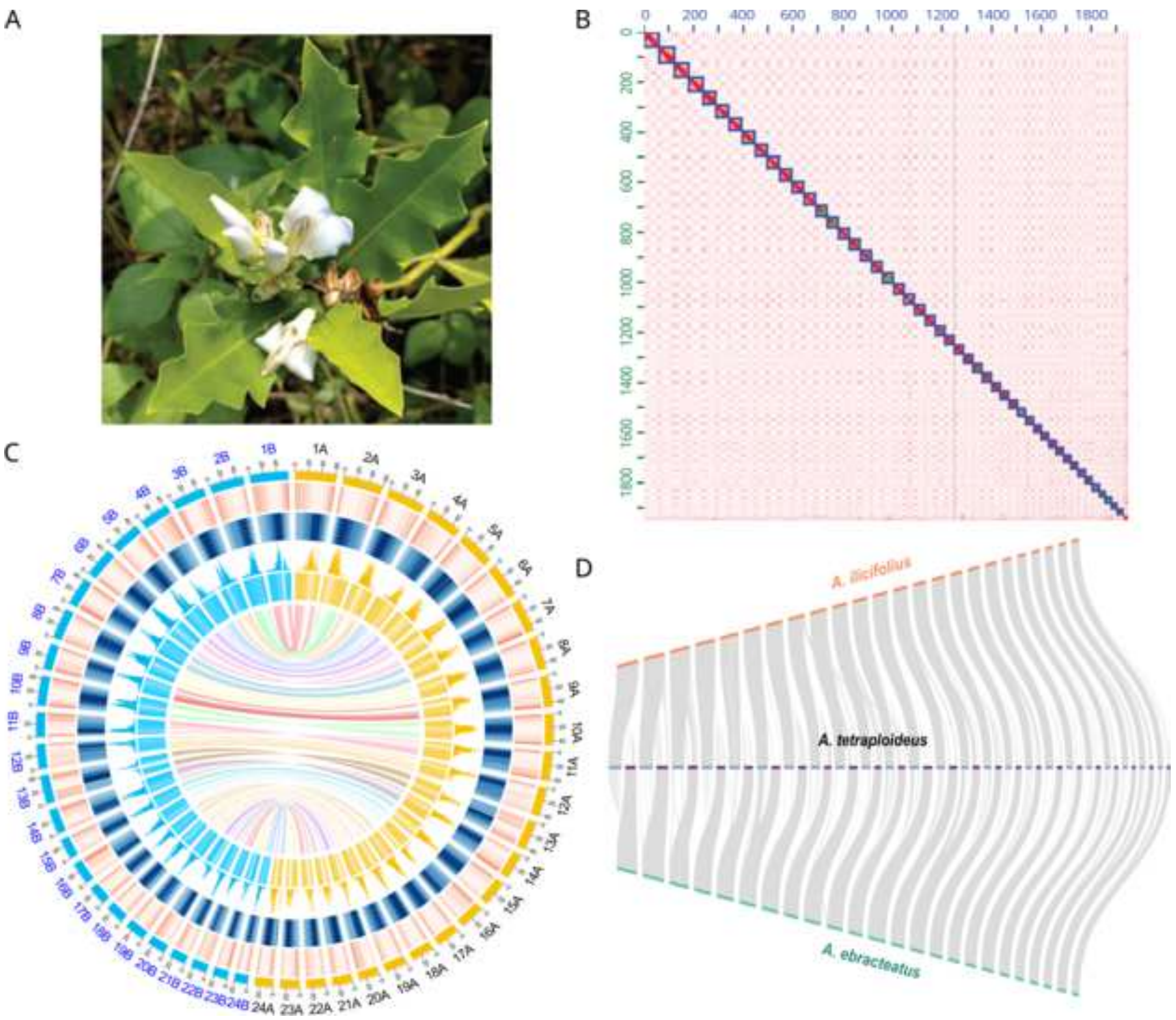
A
B
C
D
A. ilicifolius
A. tetraploideus
A. ebracteatus

Figure2 Click here to access/download;Figure;Figure2.tiff

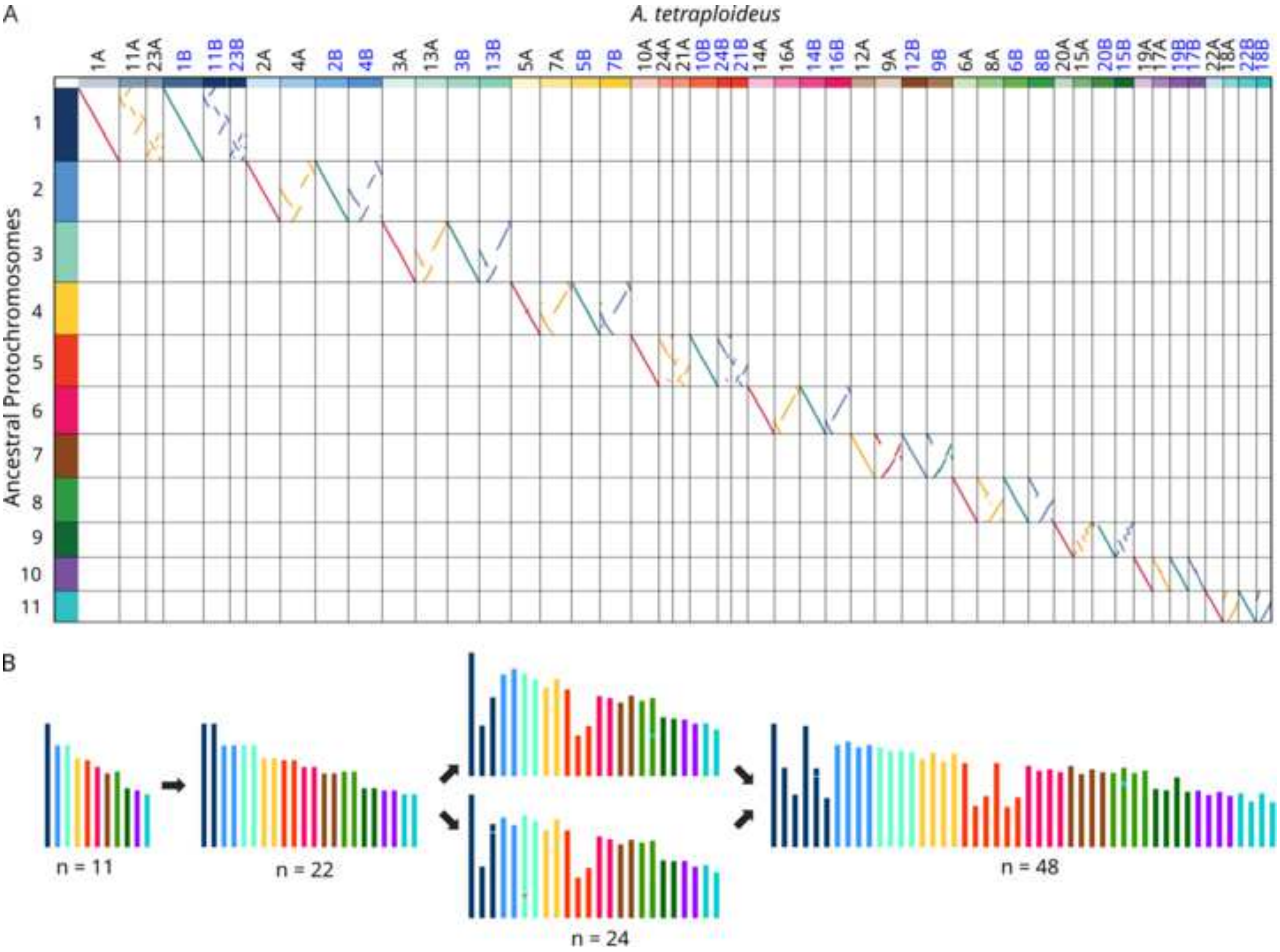

 Click here to access/download;Figure;Figure3_new.tif

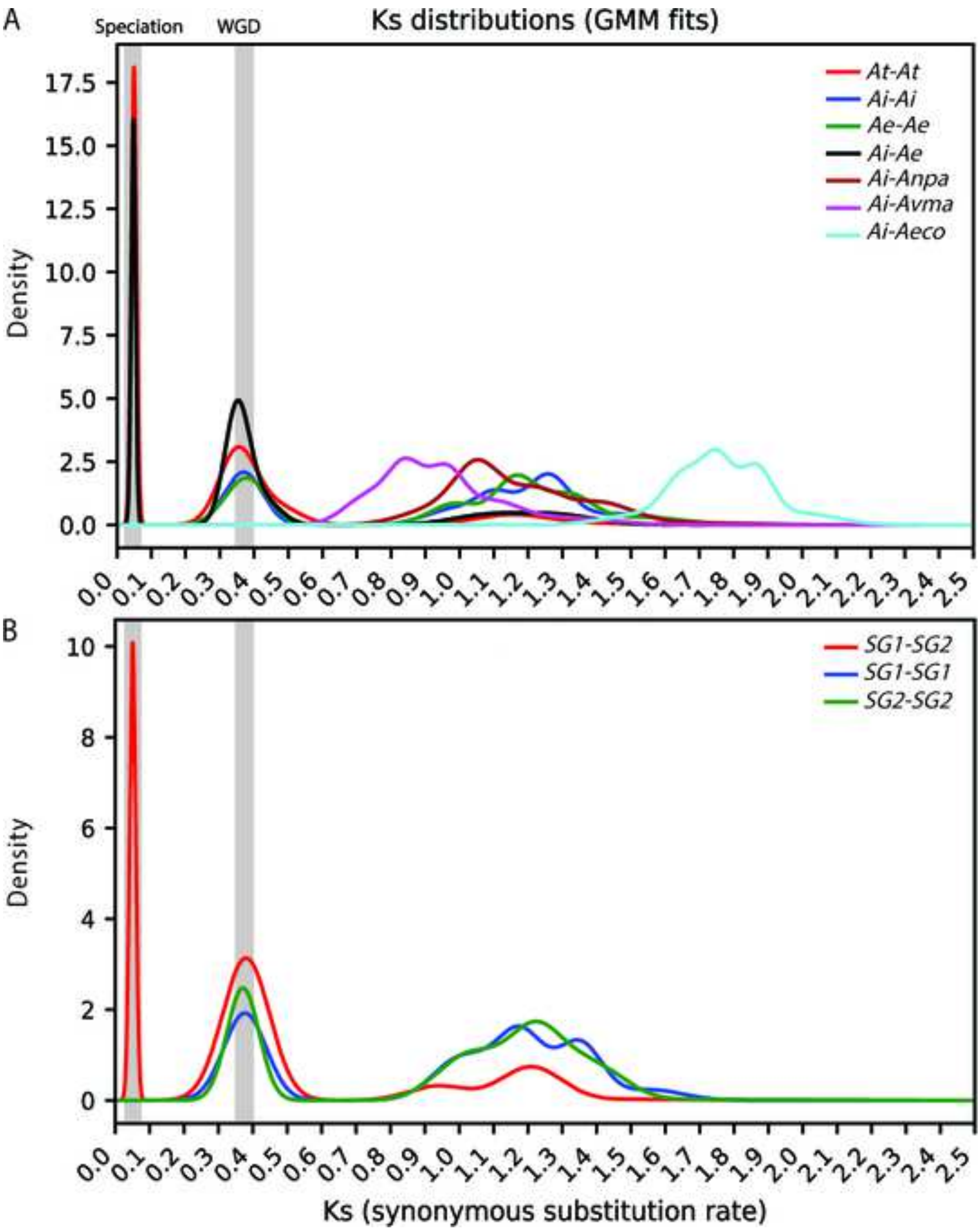

Figure4

Click here to access/download;Figure;Figure4_new.tif

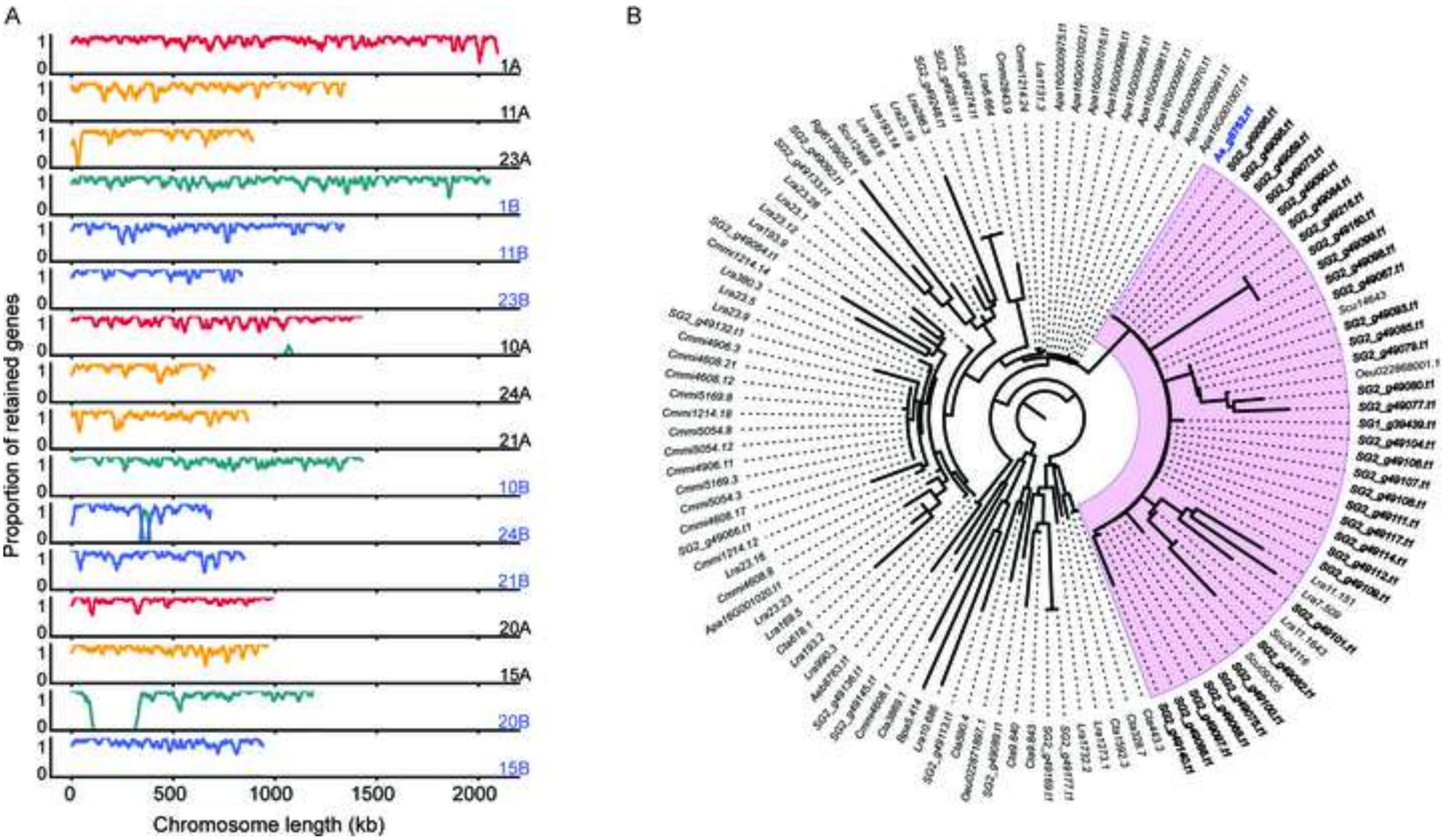

Figure5

Click here to access/download;Figure;Figure5_new.tif

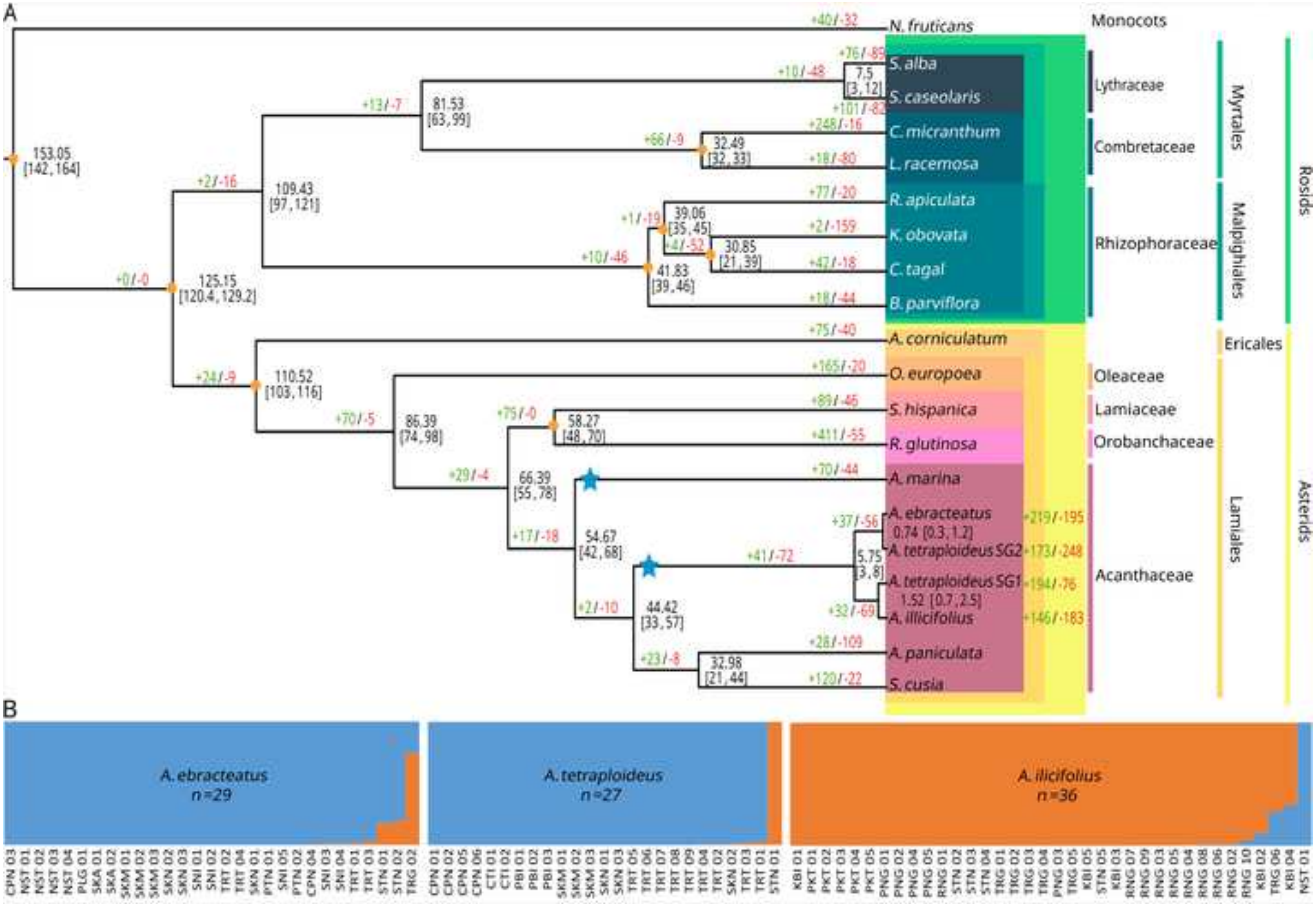

Supplementary_Figures_1-4

Click here to access/download
Supplementary Material
Supplementary_Figures.pdf

Click here to access/download
Supplementary Material
Supplementary_Table_S1.xlsx

Click here to access/download
Supplementary Material
Supplementary_Table_S2.xlsx

Click here to access/download
Supplementary Material
Supplementary_Table_S3.xlsx

Click here to access/download
Supplementary Material
Supplementary_Table_S4.xlsx

Click here to access/download
Supplementary Material
Supplementary_Table_S5.xlsx

Click here to access/download
Supplementary Material
Supplementary_Table_S6.xlsx

Click here to access/download
Supplementary Material
Supplementary_Table_S7.xlsx

Click here to access/download
Supplementary Material
Supplementary_Table_S8.xlsx

Click here to access/download
Supplementary Material
Supplementary_Table_S9.xlsx

Click here to access/download
Supplementary Material
Supplementary_Table_S10.xlsx

Click here to access/download
Supplementary Material
Supplementary_Table_S11.xlsx

Click here to access/download
Supplementary Material
Supplementary_Table_S12.xlsx

Click here to access/download
Supplementary Material
Supplementary_Table_S13.xlsx

Click here to access/download
Supplementary Material
Supplementary_Table_S14.xlsx

Click here to access/download
Supplementary Material
Supplementary_Table_S15.xlsx

Click here to access/download
Supplementary Material
Supplementary_Table_S16.xlsx

Click here to access/download
Supplementary Material
Supplementary_Table_S17.xlsx

Click here to access/download
Supplementary Material
Supplementary_Table_S18.xlsx

Click here to access/download
Supplementary Material
Supplementary_Table_S19.xlsx

Editor's Comment

1. **All web links and URLs should be given a reference number and included in the reference list rather than within the text of the manuscript. Please remove the URLs, cite them as reference and adjust the order of the reference accordingly. If you are using a reference manager then it's much easier for you to fix this. Response: All web links and URLs are now given a reference number and included in the reference list.**

   Response:

   All web links and URLs have been assigned reference numbers and are now included in the reference list. The in-text URLs have been removed.

2. **In the methods section it would be useful if you add RRID details to some of the software tools and sequencers. These needed to be listed after resources in brackets. If you are citing papers for these resources, the RRID do not replace these, and both should be included. This can be included in the methods section of the paper similar to the RRIDs included here: DNBSEQ-T7 (RRID:SCR_017981); PacBio Sequel II System (RRID:SCR_017990); PLINK (RRID:SCR_001757)**

   Response:

   RRID identifiers have been added for the relevant software tools and sequencing platforms in the Methods section, following the format suggested by the editor.

Reviewer #1:

This paper makes significant contributions to the genome assembly and polyploidization analysis of the Acanthus genus, particularly in identifying adaptation-related genes in mangrove plants. The paper is well-structured, logically organized, and supported by rich figures and tables, which help readers understand the complex da ta and analysis results. However, there are still the following suggestions for this article:

1. **Ln 80 In the final paragraph of the introduction, please use the present tense, and ensure that the first occurrence of any abbreviation is accompanied by its full form in parentheses, such as stLFR (single-tube long fragment read), to facilitate reader comprehension. Additionally, please review the rest of the article for consistency.**
   Response:
   We thank the reviewer for this helpful suggestion. The final paragraph of the Introduction has been revised to use the present tense throughout (lines 77–84 in the revised manuscript; line numbers in the revised MS word file may differ from those in the generated PDF). The full form of each abbreviation, including *stLFR* (single-tube long fragment read), has been provided at its first occurrence. We have also reviewed the entire manuscript to ensure consistent usage of tense and abbreviations.

2. **Ln 218 The estimated genome size in Supplementary Table S1 ranges from 1366 to 2496 Mb, which is a very wide range. Please specify which individual in the table corresponds to the assembled individual, as this is necessary to determine whether the assembled size matches the estimated genome size.**
   Response:
   We thank the reviewer for pointing this out. In the revised manuscript, we have specified that the SKN-At-01 individual listed in Supplementary Table S1 was used for *Acanthus tetraploideus* genome sequencing (lines 273-274).

3. **Ln 220, Ln 223 Fig 1A shows a plant photograph and does not display genome-related data, while Fig 1B is a Hi-C contact heatmap, which also does not align with the description in the text. Please verify the correspondence between all figure captions, table captions, and the content of the article. Additionally, the high repeat density observed in the central regions of chromosomes does not necessarily correspond to**

centromeric regions; it is essential to confirm whether these regions contain centromeric repeat sequences.

Response:

We thank the reviewer for this careful observation. In the revised manuscript, we have verified and corrected the correspondence between all figure citations and the text. We revised the citations of Fig. 1B (line 276), Fig. 1C (lines 279 and 287), and Fig. 1D (lines 298 and 309). Regarding the statement about centromeric regions, we agree with the reviewer's comment. Instead of referring to these as centromeric regions, we now describe them more cautiously as repeat-dense regions, with gene-annotated regions located outside these areas (lines 278-279).

4. **Ln 232 Is there any literature or experimental evidence supporting the identification of these two species as the putative progenitors? According to Supplementary Table 11, most of the alignment thresholds are below 75%. If there are other diploid species within this genus, it cannot be conclusively determined that these two species are the progenitors. The accuracy of homologous scaffolding is also questionable, which further casts doubt on the reliability of subsequent analyses.**

   Response:

   We thank the reviewer for this insightful comment. In the revised manuscript, we added supporting evidence from recent studies (Feng et al., 2024; Guo et al., 2025), which used chloroplast, nuclear, and transcriptomic data to confirm *A. ilicifolius* and *A. ebracteatus* as the most likely progenitors of *A. tetraploideus*, while excluding other congeners such as *A. volubilis* (lines 67–69).

   We agree that the overall identity values are relatively modest. This is because the values were calculated across long stretches of collinearity, where sequence divergence between the subgenomes and their progenitor genomes has accumulated over time. Nevertheless, multiple lines of genome-wide evidence, including synteny block analysis (Fig. 1D) and phylogenetic reconstruction Fig. 5 (renumbered from Fig. 4), consistently reveal one-to-one correspondences between SG1 and *A. ilicifolius*, and between SG2 and *A. ebracteatus*. We have clarified this point in lines 293–298 of the revised manuscript.

   Regarding scaffolding accuracy, we clarified that RagTag was used with minimap2 (asm5) for assembly-to-assembly alignment, which orders contigs without altering sequences (lines 149-

154). This approach yielded 24 pseudochromosomes per progenitor, capturing >93% of total lengths with strong collinearity, supporting the reliability of the scaffolding (lines 300-302).

References:

Feng H, Banerjee AK, Guo W, Yuan Y, Duan F, Ng WL, et al. Origin and evolution of a new tetraploid mangrove species in an intertidal zone. *Plant Diversity*. 2024; doi:10.1016/j.pld.2024.04.007.

Guo W, Banerjee AK, Feng H, Ng WL, Wu H, Li W, et al. Recent allopolyploidization and transcriptomic asymmetry in the mangrove shrub *Acanthus tetraploideus*. *BMC Genomics*. 2025; doi:10.1186/s12864-025-11557-2.

(*Reference numbers 1 and 4 in the revised manuscript.*)

5. **Ln 272 How were the WGD time and divergence time in Fig 2B obtained prior to the construction of the phylogenetic tree, and are they accurate, especially without 95% confidence interval? Additionally, how was the hybridization time between the two species calculated?**

Response:

We thank the reviewer for this insightful comment. In the revised manuscript, the WGD and divergence times previously shown in Fig. 2B have been removed to avoid confusion, as these values were derived from the phylogenetic framework later presented in Fig. 5 (renumbered from Fig. 4). We also noted in lines 313-314 that the reconstructed ancestral karyotype corresponded to that of Lamiales (Chen et al., 2024). To address the reviewer's concern regarding accuracy, 95% confidence intervals from the MCMCTree analysis were added to Fig. 5, and the divergence times in the text were mentioned with the 95% confidence intervals.
The hybridization time leading to the formation of the allotetraploid *A. tetraploideus* was estimated using transposable element (TE) divergence profiles, following the approach of Xu et al. (2019) and Wang et al. (2024). This method compares divergence distributions of LTR retrotransposons between subgenomes, where their initial separation reflects progenitor divergence and subsequent convergence indicates genome merger. This TE-based approach complements Ks-based dating and was applied to the SG1 and SG2 profiles to infer the timing of hybridization. Corresponding revisions have been made in the Methods (lines 220–228) and Results (lines 446–453) sections of the revised manuscript.

References:

Xu P, Xu J, Liu G, Chen L, Zhou Z, Peng W, et al.. The allotetraploid origin and asymmetrical genome evolution of the common carp Cyprinus carpio. *Nat Commun*. Nature Publishing Group; 2019; doi: 10.1038/s41467-019-12644-1.

Wang Y, Li Y, Wu W, Shao S, Fang Q, Xu S, et al.. The evolution history of an allotetraploid mangrove tree analysed with a new tool Allo4D. *Plant Biotechnology Journal*. 2024; doi: 10.1111/pbi.14281.

Chen B-Z, Li D-W, Luo K-Y, Jiu S-T, Dong X, Wang W-B, et al.. Chromosome-level assembly of Lindenbergia philippensis and comparative genomic analyses shed light on genome evolution in Lamiales. Front Plant Sci. Frontiers; 2024; doi: 10.3389/fpls.2024.1444234.

(*Reference numbers 47-48 and 57 in the revised manuscript.*)

6. **Ln 293 Ln 299 Has the Ks peak been fitted and the corresponding peak range obtained, as this step significantly impacts the determination of the WGD timing? Additionally, Ks values very close to 0 are most likely caused by tandem duplications, which could result in the subsequent peaks being shifted forward. Has filtering been applied to remove these tandem duplications?**

   Response:

   We thank the reviewer for this valuable comment. We replaced the previous kernel density estimation (KDE) with Gaussian mixture modeling (GMM) fitting, which provided a smoother and more accurate fit of the Ks distribution. We also applied a tandem duplication filter, and the minor peaks with Ks values close to 0 disappeared from the plots. Accordingly, we removed the text that previously referred to these minor peaks. The new plots showed a slight shift in the positions of both major peaks (from 0.04 to 0.05 and from 0.3 to 0.35). These new values were used to infer WGD and hybridization timing. In addition, we observed a broader peak around Ks ≈ 1.2 in *A. tetraploideus*, *A. ebracteatus*, and *A. ilicifolius*, as well as SG1 and SG2. Based on the updated results, Fig. 3 and the "3. Genome evolution" subsection of the results (lines 338-359) have been revised.

7. **Ln 322 What is the biological significance of sequence modifications, and are the genomic differences between the two species and the two subgenomes related to environmental adaptation and trait variation?**

Response:

We thank the reviewer for this insightful question. *A. ebracteatus* and SG2 exhibit greater sequence and structural variation than *A. ilicifolius* and SG1, consistent with previous reports of higher nucleotide diversity and novel expression bias (Feng et al., 2024; Guo et al., 2025). Such genomic asymmetry may have facilitated both the formation and adaptive success of the allotetraploid (Bureš et al., 2004; Cai et al., 2021; Wang et al., 2022), with SG1 providing regulatory stability and SG2 contributing adaptive flexibility. These findings indicate that the observed sequence modifications are biologically meaningful and linked to the environmental adaptation of *A. tetraploideus*. This discussion has been added to lines 561–570 of the revised manuscript.

As noted in the previous comment, the text referring to minor peaks has been removed. The original sentence, "These results, together with the presence of a small peak in the Ae–Ae Ks plot (Fig. 3A), suggested a greater degree of sequence modification in the *A. ebracteatus* genome compared to that of *A. ilicifolius*," has been replaced with: "These results were consistent with previous studies showing higher nucleotide diversity and novel expression bias in *A. ebracteatus* and SG2 compared with *A. ilicifolius* and SG1 (Feng et al., 2024; Guo et al., 2025)," summarizing the relevant findings described in the paragraph (lines 375–377).

We also revised the abstract for accuracy by replacing "Gene structure and retention analyses suggested greater structural instability in the *A. ebracteatus* genome compared to the *A. ilicifolius* genome" with "Gene structure and retention analyses revealed a smaller and more structurally flexible genome in *A. ebracteatus* and SG2 compared with *A. ilicifolius* and SG1," which is more directly supported by the results (lines 32-33).

References:

Feng H, Banerjee AK, Guo W, Yuan Y, Duan F, Ng WL, et al.. Origin and evolution of a new tetraploid mangrove species in an intertidal zone. Plant Diversity. 2024; doi: 10.1016/j.pld.2024.04.007.

Guo W, Banerjee AK, Feng H, Ng WL, Wu H, Li W, et al.. Recent allopolyploidization and transcriptomic asymmetry in the mangrove shrub Acanthus tetraploideus. BMC Genomics. 2025; doi: 10.1186/s12864-025-11557-2.

BUREŠ P, WANG Y-F, HOROVÁ L, SUDA J. Genome Size Variation in Central European Species of Cirsium (Compositae) and their Natural Hybrids. Annals of Botany. 2004; doi: 10.1093/aob/mch151.

Cai X, Chang L, Zhang T, Chen H, Zhang L, Lin R, et al.. Impacts of allopolyploidization and structural variation on intraspecific diversification in Brassica rapa. Genome Biology. 2021; doi: 10.1186/s13059-021-02383-2.
Wang Z, Yang J, Cheng F, Li P, Xin X, Wang W, et al.. Subgenome dominance and its evolutionary implications in crop domestication and breeding. Hortic Res. 2022; doi: 10.1093/hr/uhac090.

(*Reference numbers 1, 4 and 90-92 in the revised manuscript.*)

8. **Ln 334 The use of only 70 single-copy genes is notably insufficient and may significantly impact the stability of the phylogenetic tree's topology. It is recommended to replace some species and the outgroup. Additionally, neither the divergence time nor the WGD time is accompanied by a 95% confidence interval. Has bootstrap validation been performed to confirm their accuracy?**

Response:

We thank the reviewer for this important comment. The moderate number of single-copy orthologues reflects both the broad taxon sampling and the recent WGD shared by all *Acanthus* species, which together reduced the number of genes retained as single copies. Data of comparable scale have been sufficient to resolve species relationships in previous genome studies (Shen et al., 2021; Zhang et al., 2022). Our sampling strategy, which included both mangrove and non-mangrove species, was designed to maximize taxonomic diversity and ensure robust phylogenetic inference across lineages. Bootstrap validation was performed using 1,000 replicates in RAxML-NG, with most internal nodes showing 100% support. Additionally, the resulting topology is consistent with previous genomic and plastid-based phylogenies of mangroves and Acanthaceae (Xu et al., 2017; He et al., 2022; Pootakham et al., 2022), confirming the robustness of our phylogenetic reconstruction. We have now included 95% highest posterior density (HPD) intervals for both divergence and WGD times in Fig. 5 (renumbered from Fig. 4). These updates and clarifications were additionally shown in the Methods (lines 197–199) and Results (lines 394–400) sections of the revised manuscript.

References:

Shen Z, Li W, Li Y, Liu M, Cao H, Provart N, et al.. The red flower wintersweet genome provides insights into the evolution of magnoliids and the molecular mechanism for tepal color development. The Plant Journal. 2021; doi: 10.1111/tpj.15533.

Zhang B, Yao X, Chen H, Lu L. High-quality chromosome-level genome assembly of Litsea coreana L. provides insights into Magnoliids evolution and flavonoid biosynthesis. Genomics. 2022; doi: 10.1016/j.ygeno.2022.110394.

Xu L, Wang X-R, Sun K, Yu T, Xu J-H, Ding P-X, et al.. The complete chloroplast genome of Acanthus ilicifolius, an excellent mangrove plant. Mitochondrial DNA Part B. Taylor & Francis; 2021; doi: 10.1080/23802359.2021.1884022.

He Z, Feng X, Chen Q, Li L, Li S, Han K, et al.. Evolution of coastal forests based on a full set of mangrove genomes. Nat Ecol Evol. Nature Publishing Group; 2022; doi: 10.1038/s41559-022-01744-9.

Pootakham W, Sonthirod C, Naktang C, Kongkachana W, U-thoomporn S, Phetchawang P, et al.. A de novo reference assembly of the yellow mangrove Ceriops zippeliana genome. G3 Genes|Genomes|Genetics. 2022; doi: 10.1093/g3journal/jkac025.

(*Reference numbers 60-64 in the revised manuscript.*)

9. **Ln 353 According to this formula, the WGD time is significantly influenced by the calculated divergence time. Has it been corrected based on the substitution rate?**
   Response:
   We thank the reviewer for this important observation. The timing of the whole-genome duplication (WGD) event was estimated from peaks in the Ks distribution, following approaches used in previous genomic studies (Sensalari et al., 2022; Padgitt-Cobb et al., 2023). In our analysis, the substitution rate (μ) was inferred by calibrating Ks values against the divergence time between *A. ilicifolius* and *A. ebracteatus*, which was independently estimated using MCMCTree with multiple fossil calibrations under a relaxed molecular clock framework. The derived substitution rate was then incorporated into the calculation according to the relationship:

$$2\mu = \frac{Ks_{speciation}}{T_{speciation}} = \frac{Ks_{WGD}}{T_{WGD}}$$

Here, μ represents the lineage-specific substitution rate. Therefore, the WGD estimate was corrected for substitution rate variation as accounted for by the MCMCTree calibration. To improve clarity, we have revised the Methods (lines 203–228) and Results (lines 408-418 and 446–453) sections in the revised manuscript.

References:

Sensalari C, Maere S, Lohaus R. ksrates: positioning whole-genome duplications relative to speciation events in KS distributions. *Bioinformatics*. 2022; doi: 10.1093/bioinformatics/btab602.

Padgitt-Cobb LK, Pitra NJ, Matthews PD, Henning JA, Hendrix DA. An improved assembly of the “Cascade” hop (Humulus lupulus) genome uncovers signatures of molecular evolution and refines time of divergence estimates for the Cannabaceae family. *Hortic Res*. 2023; doi: 10.1093/hr/uhac281.

(*Reference numbers 44-45 in the revised manuscript.*)

10. **Ln 364 Does the CV value support K=2 as the optimal choice?**

    Response:

    We thank the reviewer for the comment. Details of the population structure analysis have been clarified in the *Methods* (Lines 254–267). STRUCTURE analysis (K = 1–10) identified K = 2 as the optimal number of clusters based on the ΔK method in *Structure Harvester*. This grouping corresponds to two major genetic groups consistent with the geographic distribution of samples along the Andaman Sea and Gulf of Thailand coasts. Relevant text (Lines 435–438) has been revised accordingly.

11. **Ln 416, Ln 432 Is the expansion and contraction of gene families related to the medicinal properties and floral trait differences mentioned in the abstract? Additionally, what specific machine learning methods were used, and what parameters and code were applied?**

    Response:

    We thank the reviewer for this insightful question. We observed contractions in terpenoid biosynthesis families, suggesting a shift in secondary metabolism associated with mangrove adaptation, in contrast to the terpenoid-rich terrestrial medicinal relatives *A. paniculata* and *S. cusia*. These results were mentioned in lines 477-486 and further clarified in the Discussion (Lines 534-538) of the revised manuscript.

    For floral traits, *A. ilicifolius* showed an expansion of chalcone synthase (CHS), consistent with its high flavonoid/phenolic content and violet flowers, whereas *A. ebracteatus* and *A. tetraploideus* displayed white and pale-violet flowers, respectively. These results were

mentioned in Lines 491–493 and further clarified in the Discussion (Lines 551–555) of the revised manuscript.

Regarding the machine learning analysis, we employed logistic regression, random forest (500 estimators), and gradient boosting (learning rate = 0.1), implemented in Python using scikit-learn. Orthogroups were prefiltered using phylogenetic signal metrics and Welch's t-test before applying recursive feature elimination. The methodology has been detailed in the revised *Methods* section (Lines 235–253), and all codes and parameters have been provided in the GigaDB database.

12. **Ln 452 The discussion section could be more concise to avoid repetition of results. Could it focus more on the biological significance and future research directions?**
    Are all the codes and scripts used for analysis provided? This is crucial for the reproducibility of the study.
    Response:
    We thank the reviewers for these valuable suggestions. In lines 516-585 of the manuscript, the Discussion section has been restructured to improve clarity, focus, and logical flow. Instead of repeating results, the revised text emphasizes the biological significance of our findings and situates them within the broader evolutionary and ecological context of mangrove adaptation. We now highlight how ancient WGD, lineage-specific gene family evolution, and recent hybridization jointly shaped the evolutionary trajectory of *Acanthus*. We have also added a conclusion and future research directions at the end of the discussion.

Reviewer #2:

This study presents valuable new genome assemblies for three Acanthus mangrove species, employed to investigate their evolutionary history—including an ancient whole-genome duplication (WGD) event and a recent allopolyploidization—as well as adaptive gene family evolution and population structure. While the breadth of analyses is commendable, the current work lacks sufficient analytical depth and a cohesive narrative. Connections between distinct analytical components are often unclear, and comparative genomic analyses, in particular, require more thorough investigation.

Major points:

1. **In Line 285, the Ai-Ae comparison exhibits a peak trend opposite to that of the At-At comparison. Further analysis is advised to elucidate the underlying reasons for this discrepancy.**

   Response:

   We thank the reviewer for this valuable suggestion. In the original analysis, Ks distributions were estimated using Gaussian kernel density estimation (KDE). In the revised version, we replotted the Ks histograms and applied Gaussian mixture modeling (GMM) to visualize the distributions. The updated distribution plots have been updated in Fig. 3. In these new plots, the peak patterns in the Ai–Ae and At–At comparisons now follow the same trend. The subsection describing the Ks analysis has been reorganized in lines 338–359 (line numbers in the revised MS word file may differ from those in the generated PDF) of the revised manuscript.

2. **In Lines 299 and 302, the two noted sets of Ks-related genes should be explicitly identified and screened. I recommend analyzing whether their distribution within each genome is random and investigating whether they exhibit significant enrichment in KEGG or GO functional categories.**

   Response:

   We thank the reviewer for this valuable suggestion. As noted by another reviewer, we reanalyzed the Ks distribution and found that the minor peaks near Ks ≈ 0 resulted from tandem duplication. After applying tandem duplication filtering, these minor peaks disappeared. We, therefore, removed the text mentioning these peaks in the revised manuscript.

3. **In Line 326, since the 176 genes annotated in this region do not derive from any ancestral genome sequence, it is critical to discuss the potential origin of this genomic region. Additionally, 105 of these 176 genes belong to three gene families; please elucidate the functions of these families. The results suggest this region may have arisen via tandem or segmental duplication, and further in-depth analysis of its origin and the characteristics of its constituent genes is recommended.**

   Response:

   We thank the reviewer for this insightful comment. In the revised manuscript, we clarified that the 176 genes in the extended region of chromosome 20B do not form any collinearity blocks with the progenitor genomes but likely originated through segmental duplication, supported by the detection of multiple internal collinearity blocks. Among the three gene families identified, we visualized the gene tree of the largest family, which showed 33 paralogous gene copies in *A. tetraploideus* that were orthologous to a single gene in *A. ebracteatus*. Similar duplication patterns were also observed in *L. racemosa*, *C. micranthum*, and *A. paniculata* within the same tree. We have created new Fig. 4 combining the gene retention analysis results previously shown in Fig. 3C with this gene tree. Consistently, previous studies have shown that local duplications often contribute to genome expansion and structural variation in polyploids (Qiao et al., 2019). BLAST searches of these 105 proteins revealed matches to proteins from multiple plant species, which were annotated as uncharacterized or hypothetical. Further investigation is needed in the future to clarify their functions and evolutionary significance. The results have been revised accordingly in lines 378-392.

   References

   Qiao X. et al. (2019). Gene duplication and evolution in recurring polyploidization–diploidization cycles in plants. Genome Biology, 20: 38. https://doi.org/10.1186/s13059-019-1650-2

   (*Reference numbers 59 in the revised manuscript.*)

4. **In Line 359, the explanation that inconsistent divergence times (between the tetraploid's subgenomes and their diploid progenitors) stem from differences in geographic distribution is overly simplistic. Specific underlying mechanisms must be elaborated. For example, investigate whether this discrepancy could arise from post-formation gene introgression via backcrossing between the allopolyploid and its diploid ancestors.**

Response:

We thank the reviewer for this valuable suggestion. The collection sources of our materials (*A. ilicifolius* from the Andaman coast and *A. ebracteatus* and *A. tetraploideus* from the Gulf of Thailand), together with the observed genetic separation between Andaman and Gulf populations, suggest a link between their geographic distributions and evolutionary divergence. We hypothesize that the sequenced *A. ilicifolius* individual from the Andaman coast may have diverged substantially from the ancestral *A. ilicifolius* lineage that contributed as SG1 in *A. tetraploideus* around ~5.7 Mya. In contrast, *A. ebracteatus* and *A. tetraploideus* still co-occur in the Gulf of Thailand and may therefore have been subjected to more similar environmental conditions. Consequently, the divergence between *A. ilicifolius* and SG1 appears greater than that between *A. ebracteatus* and SG2.

We agree that geographic separation alone cannot fully explain this pattern. However, our current dataset, which lacks *A. ilicifolius* genome data from the Gulf of Thailand, does not permit a formal test of introgression. In Response: to the reviewer's suggestion, we have clarified in the revised manuscript that spatial overlap between *A. ebracteatus* and *A. tetraploideus* could have facilitated post-formation gene flow or backcrossing, potentially contributing to the lower divergence observed between SG2 and its diploid progenitor. The continued geographic proximity of these species may have promoted stronger genomic continuity or reduced divergence along the SG2 lineage. All these clarifications have been added to lines 424–434 in the revised manuscript.

5. **In Line 364, the manuscript must clarify its SNP calling strategy in the Methods section, including details of the reference genome used, specific procedures, and all software parameters. The current variant calling approach appears methodologically flawed: if the allotetraploid A. tetraploideus genome was used as the reference, calling SNPs from diploid samples (A. ilicifolius and A. ebracteatus) would be highly problematic due to ambiguous read mapping between homeologous subgenomes, leading to unreliable variant data. Conversely, using a diploid genome as the reference would introduce severe mapping bias for tetraploid reads, as reads from the non-reference subgenome would fail to align properly, resulting in an incomplete and skewed SNP dataset. This methodology is thus not robust for this diploid-polyploid system, and the validity of downstream analyses is questionable.**

Response:

We thank the reviewer for this insightful comment. In the revised manuscript, we have clarified the SNP calling strategy in a new subsection of the Methods (Lines 254–260). To avoid mapping bias in the diploid–polyploid system, RADseq reads from each species were mapped to their respective genome assemblies, and SNPs were called independently for each population. This strategy minimized cross-subgenome misalignment and ensured reliable variant detection across both diploid and tetraploid genomes.

The population structure analysis is also described in detail in the revised Methods (Lines 261–267), including the use of STRUCTURE v2.3.4 with 20 replicates for K = 1–10, 100,000 burn-in steps, and 500,000 MCMC iterations under an admixture model. The optimal K = 2 was determined using the ΔK method implemented in Structure Harvester, revealing two major genetic clusters corresponding to the Andaman and Gulf of Thailand coasts.

6. **In Line 377, supporting scientific literature must be provided to validate the use of TE divergence rates for dating subgenome merger and divergence events. The current approach— inferring the timing of this event solely from the intersection of two kernel density estimates of LTR retrotransposon divergence in Supplementary Fig. S3—lacks sufficient theoretical and methodological justification. Pinpointing this intersection as the definitive merger time is an overreach, as it is not adequately supported by a single data feature and requires validation via established methods.**

Response:

We appreciate the reviewer's comment. The age of the hybridization event leading to the allotetraploid *A. tetraploideus* was inferred from transposable element (TE) divergence profiles, following the approach of Xu et al. (2019). This method was also used by Wang et al. (2024) to estimate the timing of polyploidization and hybridization events in mangrove evolution. Both studies compared the divergence distributions of LTR retrotransposons between parental subgenomes, showing that the initial separation of TE divergence distributions corresponds to the substitution rate at progenitor divergence, while their subsequent convergence reflects the substitution rate at genome merger. This approach complements Ks-based dating, particularly for the hybridization event where corresponding Ks values were unavailable. Following this framework, we compared the TE divergence profiles of SG1 and SG2 in *A. tetraploideus* and applied the same calibration method to estimate the

timing of the genome merger event. For clarity, we have revised the Methods (lines 203–228) and Results (lines 446–453) in the revised manuscript. Supplementary Fig. S3 has been renumbered as Supplementary Fig. S1 in the revised manuscript.

References:

Xu P, Xu J, Liu G, Chen L, Zhou Z, Peng W, et al.. The allotetraploid origin and asymmetrical genome evolution of the common carp Cyprinus carpio. *Nat Commun*. Nature Publishing Group; 2019; doi: 10.1038/s41467-019-12644-1.

Wang Y, Li Y, Wu W, Shao S, Fang Q, Xu S, et al.. The evolution history of an allotetraploid mangrove tree analysed with a new tool Allo4D. *Plant Biotechnology Journal*. 2024; doi: 10.1111/pbi.14281.

(*Reference numbers 47 and 48 in the revised manuscript.*)

7. **In Line 452, the Discussion section is overly verbose and requires restructuring. It tends to summarize results rather than interpret their implications. Please rewrite this section to be more focused, logical, and analytical, with clear explanations of the significance and context of your findings.**

   Response:

   We thank the reviewers for these valuable suggestions. In lines 516-585 of the revised manuscript, the Discussion section has been restructured to improve clarity, focus, and logical flow. Instead of repeating results, the revised text emphasizes the biological significance of our findings and situates them within the broader evolutionary and ecological context of mangrove adaptation. We now highlight how ancient WGD, lineage-specific gene family evolution, and recent hybridization jointly shaped the evolutionary trajectory of *Acanthus*. We have also added a conclusion and future research directions at the end of the discussion.

Minor points:

8. **In Line 217, please explicitly indicate which table reports the values "a total contig length of 1.95 Gb" and "a contig N50 of 42.67 Mb."**

   Response:

We appreciate the reviewer's comment. These values correspond to the preliminary PacBio HiFi assembly, which served as the template contigs for Hi-C scaffolding. Therefore, they were not presented in a separate table but are directly reported in the text.

9. **The numbering of Supplementary Table S2 and all subsequent supplementary tables appears incorrect; please carefully review and revise table numbering accordingly.**
   Response:
   We appreciate the reviewer's comment. We have carefully reviewed and corrected the numbering of Supplementary Table S2 and all subsequent supplementary tables throughout the manuscript and supporting files.

10. **In Lines 220, 223, 231, and 247, citations to specific panels of Fig. 1 are misaligned with the text. Please revise figure citations to ensure the correct figure components are referenced in their respective contexts.**
   Response:
   We thank the reviewer for this careful observation. In the revised manuscript, we have verified and corrected the correspondence between figure citations and text. Specifically, we revised the citations of Fig. 1B (line 276), Fig. 1C (lines 279 and 287), and Fig. 1D (lines 298 and 309) in the revised manuscript.

11. **In Line 224, Supplementary Table S4 reports BUSCO completeness at the genome level under genome mode, which does not reflect gene annotation completeness. Please provide an additional BUSCO assessment based on predicted protein sequences of all annotated genes to evaluate gene annotation quality.**
   Response:
   We thank the reviewer for this helpful suggestion. We have performed an additional BUSCO assessment in protein mode using the predicted protein sequences of all annotated genes. The results have been added in lines 304–308 of the revised manuscript and were highly consistent with those obtained from the BUSCO analysis in genome mode.

12. **In Line 336, for the phylogenetic tree in Fig. 4A, please use distinct colored blocks to indicate the clade, order, and family of each analyzed species.**
   Response:

We thank the reviewer for this helpful suggestion. We have added distinct colored blocks to the phylogenetic tree to indicate the clade, order, and family of each analyzed species. In the revised manuscript, the phylogenetic tree has been renumbered from Fig. 5 (renumbered from Fig. 4).

13. **In Line 351, please relocate mathematical formulas to the Methods section for detailed explanation and clarification.**

    Response:

    We appreciate the reviewer's comment. The corresponding equation has been relocated to the new subsection titled "7. Estimation of the timing of whole-genome duplication and hybridization events" in the Methods section for detailed explanation and clarification (Lines 203–228).

14. **In Line 431, please provide detailed analytical procedures of the machine learning approach in the Methods section.**

    Response:

    We thank the reviewer for this helpful suggestion. In the revised manuscript, we have expanded the Methods section to include a detailed description of the machine learning workflow, covering data inputs, phylogenetic filtering, feature selection, and model implementation. To enhance clarity and accessibility, this content has been organized into a new subsection titled **"**Gene family expansion/contraction analysis and machine learning-based selection of lineage-specific gene families**"** (Lines 235–253).

15. **In Line 441, add a citation for Table 1 in the main text.**

    Response:

    We thank the reviewer for the suggestion. A citation for Table 1 has been added to Line 482 of the revised manuscript.
