# Supplementary material for: Genome Assembly of Three Shrub Mangroves in the Genus Acanthus Reveals Two Polyploidy Events and Expansion of Genes Linked to Root Adaptation in Coastal Habitats
Source: Gigascience. 2026 Jan 2;15:giaf162. doi: 10.1093/gigascience/giaf162 (PMC12903786; doi:10.1093/gigascience/giaf162)
Supplement: giaf162_Authors_Response_To_Reviewer_Comments_original_submission [file giaf162_authors_response_to_reviewer_comments_original_submission.pdf]

Author's Response To Reviewer Comments

Editor's Comment
All web links and URLs should be given a reference number and included in the reference list rather than within the text of the manuscript. Please remove the URLs, cite them as reference and adjust the order of the reference accordingly. If you are using a reference manager then it's much easier for you to fix this.
Response: All web links and URLs are now given a reference number and included in the reference list.
Response:
All web links and URLs have been assigned reference numbers and are now included in the reference list. The in-text URLs have been removed.
In the methods section it would be useful if you add RRID details to some of the software tools and sequencers. These needed to be listed after resources in brackets. If you are citing papers for these resources, the RRID do not replace these, and both should be included. This can be included in the methods section of the paper similar to the RRIDs included here: DNBSEQ-T7 (RRID:SCR_017981); PacBio Sequel II System (RRID:SCR_017990); PLINK (RRID:SCR_001757)
Response:
RRID identifiers have been added for the relevant software tools and sequencing platforms in the Methods section, following the format suggested by the editor.

Reviewer #1:
This paper makes significant contributions to the genome assembly and polyploidization analysis of the Acanthus genus, particularly in identifying adaptation-related genes in mangrove plants. The paper is well-structured, logically organized, and supported by rich figures and tables, which help readers understand the complex da ta and analysis results. However, there are still the following suggestions for this article:
Ln 80 In the final paragraph of the introduction, please use the present tense, and ensure that the first occurrence of any abbreviation is accompanied by its full form in parentheses, such as stLFR (single-tube long fragment read), to facilitate reader comprehension. Additionally, please review the rest of the article for consistency.
Response:
We thank the reviewer for this helpful suggestion. The final paragraph of the Introduction has been revised to use the present tense throughout (lines 77–84 in the revised manuscript; line numbers in the revised MS word file may differ from those in the generated PDF). The full form of each abbreviation, including stLFR (single-tube long fragment read), has been provided at its first occurrence. We have also reviewed the entire manuscript to ensure consistent usage of tense and abbreviations.
Ln 218 The estimated genome size in Supplementary Table S1 ranges from 1366 to 2496 Mb, which is a very wide range. Please specify which individual in the table corresponds to the assembled individual, as this is necessary to determine whether the assembled size matches the estimated genome size.
Response:
We thank the reviewer for pointing this out. In the revised manuscript, we have specified that the SKN-At-01 individual listed in Supplementary Table S1 was used for Acanthus tetraploideus genome sequencing (lines 273-274).
Ln 220, Ln 223 Fig 1A shows a plant photograph and does not display genome-related data, while Fig 1B is a Hi-C contact heatmap, which also does not align with the description in the text. Please verify the correspondence between all figure captions, table captions, and the content of the article. Additionally, the high repeat density observed in the central regions of chromosomes does not necessarily correspond to centromeric regions; it is essential to confirm whether these regions contain centromeric repeat sequences.
Response:
We thank the reviewer for this careful observation. In the revised manuscript, we have verified and corrected the correspondence between all figure citations and the text. We revised the citations of Fig. 1B (line 276), Fig. 1C (lines 279 and 287), and Fig. 1D (lines 298 and 309). Regarding the statement about centromeric regions, we agree with the reviewer's comment. Instead of referring to these as centromeric regions, we now describe them more cautiously as repeat-dense regions, with gene-annotated regions located outside these areas (lines 278-279).
Ln 232 Is there any literature or experimental evidence supporting the identification of these two species

as the putative progenitors? According to Supplementary Table 11, most of the alignment thresholds are below 75%. If there are other diploid species within this genus, it cannot be conclusively determined that these two species are the progenitors. The accuracy of homologous scaffolding is also questionable, which further casts doubt on the reliability of subsequent analyses.

Response:

We thank the reviewer for this insightful comment. In the revised manuscript, we added supporting evidence from recent studies (Feng et al., 2024; Guo et al., 2025), which used chloroplast, nuclear, and transcriptomic data to confirm A. ilicifolius and A. ebracteatus as the most likely progenitors of A. tetraploideus, while excluding other congeners such as A. volubilis (lines 67–69).

We agree that the overall identity values are relatively modest. This is because the values were calculated across long stretches of collinearity, where sequence divergence between the subgenomes and their progenitor genomes has accumulated over time. Nevertheless, multiple lines of genome-wide evidence, including synteny block analysis (Fig. 1D) and phylogenetic reconstruction Fig. 5 (renumbered from Fig. 4), consistently reveal one-to-one correspondences between SG1 and A. ilicifolius, and between SG2 and A. ebracteatus. We have clarified this point in lines 293–298 of the revised manuscript.

Regarding scaffolding accuracy, we clarified that RagTag was used with minimap2 (asm5) for assembly-to-assembly alignment, which orders contigs without altering sequences (lines 149-154). This approach yielded 24 pseudochromosomes per progenitor, capturing >93% of total lengths with strong collinearity, supporting the reliability of the scaffolding (lines 300-302).

References:

Feng H, Banerjee AK, Guo W, Yuan Y, Duan F, Ng WL, et al. Origin and evolution of a new tetraploid mangrove species in an intertidal zone. Plant Diversity. 2024; doi:10.1016/j.pld.2024.04.007.

Guo W, Banerjee AK, Feng H, Ng WL, Wu H, Li W, et al. Recent allopolyploidization and transcriptomic asymmetry in the mangrove shrub Acanthus tetraploideus. BMC Genomics. 2025; doi:10.1186/s12864-025-11557-2.

(Reference numbers 1 and 4 in the revised manuscript.)

Ln 272 How were the WGD time and divergence time in Fig 2B obtained prior to the construction of the phylogenetic tree, and are they accurate, especially without 95% confidence interval? Additionally, how was the hybridization time between the two species calculated?

Response:

We thank the reviewer for this insightful comment. In the revised manuscript, the WGD and divergence times previously shown in Fig. 2B have been removed to avoid confusion, as these values were derived from the phylogenetic framework later presented in Fig. 5 (renumbered from Fig. 4). We also noted in lines 313-314 that the reconstructed ancestral karyotype corresponded to that of Lamiales (Chen et al., 2024). To address the reviewer's concern regarding accuracy, 95% confidence intervals from the MCMCTree analysis were added to Fig. 5, and the divergence times in the text were mentioned with the 95% confidence intervals.

The hybridization time leading to the formation of the allotetraploid A. tetraploideus was estimated using transposable element (TE) divergence profiles, following the approach of Xu et al. (2019) and Wang et al. (2024). This method compares divergence distributions of LTR retrotransposons between subgenomes, where their initial separation reflects progenitor divergence and subsequent convergence indicates genome merger. This TE-based approach complements Ks-based dating and was applied to the SG1 and SG2 profiles to infer the timing of hybridization. Corresponding revisions have been made in the Methods (lines 220–228) and Results (lines 446–453) sections of the revised manuscript.

References:

Xu P, Xu J, Liu G, Chen L, Zhou Z, Peng W, et al.. The allotetraploid origin and asymmetrical genome evolution of the common carp Cyprinus carpio. Nat Commun. Nature Publishing Group; 2019; doi: 10.1038/s41467-019-12644-1.

Wang Y, Li Y, Wu W, Shao S, Fang Q, Xu S, et al.. The evolution history of an allotetraploid mangrove tree analysed with a new tool Allo4D. Plant Biotechnology Journal. 2024; doi: 10.1111/pbi.14281.

Chen B-Z, Li D-W, Luo K-Y, Jiu S-T, Dong X, Wang W-B, et al.. Chromosome-level assembly of Lindenbergia philippensis and comparative genomic analyses shed light on genome evolution in Lamiales. Front Plant Sci. Frontiers; 2024; doi: 10.3389/fpls.2024.1444234.

(Reference numbers 47-48 and 57 in the revised manuscript.)

Ln 293 Ln 299 Has the Ks peak been fitted and the corresponding peak range obtained, as this step significantly impacts the determination of the WGD timing? Additionally, Ks values very close to 0 are most likely caused by tandem duplications, which could result in the subsequent peaks being shifted forward.

Has filtering been applied to remove these tandem duplications?
Response:
We thank the reviewer for this valuable comment. We replaced the previous kernel density estimation (KDE) with Gaussian mixture modeling (GMM) fitting, which provided a smoother and more accurate fit of the Ks distribution. We also applied a tandem duplication filter, and the minor peaks with Ks values close to 0 disappeared from the plots. Accordingly, we removed the text that previously referred to these minor peaks. The new plots showed a slight shift in the positions of both major peaks (from 0.04 to 0.05 and from 0.3 to 0.35). These new values were used to infer WGD and hybridization timing. In addition, we observed a broader peak around Ks ≈ 1.2 in A. tetraploideus, A. ebracteatus, and A. ilicifolius, as well as SG1 and SG2. Based on the updated results, Fig. 3 and the "3. Genome evolution" subsection of the results (line 338-359) have been revised.
Ln 322 What is the biological significance of sequence modifications, and are the genomic differences between the two species and the two subgenomes related to environmental adaptation and trait variation?
Response:
We thank the reviewer for this insightful question. A. ebracteatus and SG2 exhibit greater sequence and structural variation than A. ilicifolius and SG1, consistent with previous reports of higher nucleotide diversity and novel expression bias (Feng et al., 2024; Guo et al., 2025). Such genomic asymmetry may have facilitated both the formation and adaptive success of the allotetraploid (Bureš et al., 2004; Cai et al., 2021; Wang et al., 2022), with SG1 providing regulatory stability and SG2 contributing adaptive flexibility. These findings indicate that the observed sequence modifications are biologically meaningful and linked to the environmental adaptation of A. tetraploideus. This discussion has been added to lines 561–570 of the revised manuscript.
As noted in the previous comment, the text referring to minor peaks has been removed. The original sentence, "These results, together with the presence of a small peak in the Ae–Ae Ks plot (Fig. 3A), suggested a greater degree of sequence modification in the A. ebracteatus genome compared to that of A. ilicifolius," has been replaced with: "These results were consistent with previous studies showing higher nucleotide diversity and novel expression bias in A. ebracteatus and SG2 compared with A. ilicifolius and SG1 (Feng et al., 2024; Guo et al., 2025)," summarizing the relevant findings described in the paragraph (lines 375–377).
We also revised the abstract for accuracy by replacing "Gene structure and retention analyses suggested greater structural instability in the A. ebracteatus genome compared to the A. ilicifolius genome" with "Gene structure and retention analyses revealed a smaller and more structurally flexible genome in A. ebracteatus and SG2 compared with A. ilicifolius and SG1," which is more directly supported by the results (lines 32-33).
References:
Feng H, Banerjee AK, Guo W, Yuan Y, Duan F, Ng WL, et al.. Origin and evolution of a new tetraploid mangrove species in an intertidal zone. Plant Diversity. 2024; doi: 10.1016/j.pld.2024.04.007.
Guo W, Banerjee AK, Feng H, Ng WL, Wu H, Li W, et al.. Recent allopolyploidization and transcriptomic asymmetry in the mangrove shrub Acanthus tetraploideus. BMC Genomics. 2025; doi: 10.1186/s12864-025-11557-2.
BUREŠ P, WANG Y-F, HOROVÁ L, SUDA J. Genome Size Variation in Central European Species of Cirsium (Compositae) and their Natural Hybrids. Annals of Botany. 2004; doi: 10.1093/aob/mch151.
Cai X, Chang L, Zhang T, Chen H, Zhang L, Lin R, et al.. Impacts of allopolyploidization and structural variation on intraspecific diversification in Brassica rapa. Genome Biology. 2021; doi: 10.1186/s13059-021-02383-2.
Wang Z, Yang J, Cheng F, Li P, Xin X, Wang W, et al.. Subgenome dominance and its evolutionary implications in crop domestication and breeding. Hortic Res. 2022; doi: 10.1093/hr/uhac090.
(Reference numbers 1, 4 and 90-92 in the revised manuscript.)
Ln 334 The use of only 70 single-copy genes is notably insufficient and may significantly impact the stability of the phylogenetic tree's topology. It is recommended to replace some species and the outgroup. Additionally, neither the divergence time nor the WGD time is accompanied by a 95% confidence interval. Has bootstrap validation been performed to confirm their accuracy?
Response:
We thank the reviewer for this important comment. The moderate number of single-copy orthologues reflects both the broad taxon sampling and the recent WGD shared by all Acanthus species, which together reduced the number of genes retained as single copies. Data of comparable scale have been sufficient to resolve species relationships in previous genome studies (Shen et al., 2021; Zhang et al., 2022). Our

sampling strategy, which included both mangrove and non-mangrove species, was designed to maximize taxonomic diversity and ensure robust phylogenetic inference across lineages. Bootstrap validation was performed using 1,000 replicates in RAxML-NG, with most internal nodes showing 100% support. Additionally, the resulting topology is consistent with previous genomic and plastid-based phylogenies of mangroves and Acanthaceae (Xu et al., 2017; He et al., 2022; Pootakham et al., 2022), confirming the robustness of our phylogenetic reconstruction. We have now included 95% highest posterior density (HPD) intervals for both divergence and WGD times in Fig. 5 (renumbered from Fig. 4). These updates and clarifications were additionally shown in the Methods (lines 197–199) and Results (lines 394–400) sections of the revised manuscript.

References:

Shen Z, Li W, Li Y, Liu M, Cao H, Provart N, et al.. The red flower wintersweet genome provides insights into the evolution of magnoliids and the molecular mechanism for tepal color development. The Plant Journal. 2021; doi: 10.1111/tpj.15533.

Zhang B, Yao X, Chen H, Lu L. High-quality chromosome-level genome assembly of Litsea coreana L. provides insights into Magnoliids evolution and flavonoid biosynthesis. Genomics. 2022; doi: 10.1016/j.ygeno.2022.110394.

Xu L, Wang X-R, Sun K, Yu T, Xu J-H, Ding P-X, et al.. The complete chloroplast genome of Acanthus ilicifolius, an excellent mangrove plant. Mitochondrial DNA Part B. Taylor & Francis; 2021; doi: 10.1080/23802359.2021.1884022.

He Z, Feng X, Chen Q, Li L, Li S, Han K, et al.. Evolution of coastal forests based on a full set of mangrove genomes. Nat Ecol Evol. Nature Publishing Group; 2022; doi: 10.1038/s41559-022-01744-9.

Pootakham W, Sonthirod C, Naktang C, Kongkachana W, U-thoomporn S, Phetchawang P, et al.. A de novo reference assembly of the yellow mangrove Ceriops zippeliana genome. G3 Genes|Genomes|Genetics. 2022; doi: 10.1093/g3journal/jkac025.

(Reference numbers 60-64 in the revised manuscript.)

Ln 353 According to this formula, the WGD time is significantly influenced by the calculated divergence time. Has it been corrected based on the substitution rate?

Response:

We thank the reviewer for this important observation. The timing of the whole-genome duplication (WGD) event was estimated from peaks in the Ks distribution, following approaches used in previous genomic studies (Sensalari et al., 2022; Padgitt-Cobb et al., 2023). In our analysis, the substitution rate (μ) was inferred by calibrating Ks values against the divergence time between A. ilicifolius and A. ebracteatus, which was independently estimated using MCMCTree with multiple fossil calibrations under a relaxed molecular clock framework. The derived substitution rate was then incorporated into the calculation according to the relationship:

$$2\mu = \frac{Ks_{speciation}}{T_{speciation}} = \frac{Ks_{WGD}}{T_{WGD}}$$

Here, μ represents the lineage-specific substitution rate. Therefore, the WGD estimate was corrected for substitution rate variation as accounted for by the MCMCTree calibration. To improve clarity, we have revised the Methods (lines 203–228) and Results (lines 408-418 and 446–453) sections in the revised manuscript.

References:

Sensalari C, Maere S, Lohaus R. ksrates: positioning whole-genome duplications relative to speciation events in KS distributions. Bioinformatics. 2022; doi: 10.1093/bioinformatics/btab602.

Padgitt-Cobb LK, Pitra NJ, Matthews PD, Henning JA, Hendrix DA. An improved assembly of the “Cascade” hop (Humulus lupulus) genome uncovers signatures of molecular evolution and refines time of divergence estimates for the Cannabaceae family. Hortic Res. 2023; doi: 10.1093/hr/uhac281.

(Reference numbers 44-45 in the revised manuscript.)

Ln 364 Does the CV value support K=2 as the optimal choice?

Response:

We thank the reviewer for the comment. Details of the population structure analysis have been clarified in the Methods (Lines 254–267). STRUCTURE analysis (K = 1–10) identified K = 2 as the optimal number of clusters based on the ΔK method in Structure Harvester. This grouping corresponds to two major genetic groups consistent with the geographic distribution of samples along the Andaman Sea and Gulf of Thailand coasts. Relevant text (Lines 435–438) has been revised accordingly.

Ln 416, Ln 432 Is the expansion and contraction of gene families related to the medicinal properties and floral trait differences mentioned in the abstract? Additionally, what specific machine learning methods were used, and what parameters and code were applied?

Response:
We thank the reviewer for this insightful question. We observed contractions in terpenoid biosynthesis families, suggesting a shift in secondary metabolism associated with mangrove adaptation, in contrast to the terpenoid-rich terrestrial medicinal relatives A. paniculata and S. cusia. These results were mentioned in lines 477-486 and further clarified in the Discussion (Lines 534-538) of the revised manuscript.
For floral traits, A. ilicifolius showed an expansion of chalcone synthase (CHS), consistent with its high flavonoid/phenolic content and violet flowers, whereas A. ebracteatus and A. tetraploideus displayed white and pale-violet flowers, respectively. These results were mentioned in Lines 491–493 and further clarified in the Discussion (Lines 551–555) of the revised manuscript.
Regarding the machine learning analysis, we employed logistic regression, random forest (500 estimators), and gradient boosting (learning rate = 0.1), implemented in Python using scikit-learn. Orthogroups were prefiltered using phylogenetic signal metrics and Welch's t-test before applying recursive feature elimination. The methodology has been detailed in the revised Methods section (Lines 235–253), and all codes and parameters have been provided in the GigaDB database.
Ln 452 The discussion section could be more concise to avoid repetition of results. Could it focus more on the biological significance and future research directions?
Are all the codes and scripts used for analysis provided? This is crucial for the reproducibility of the study.
Response:
We thank the reviewers for these valuable suggestions. In lines 516-585 of the manuscript, the Discussion section has been restructured to improve clarity, focus, and logical flow. Instead of repeating results, the revised text emphasizes the biological significance of our findings and situates them within the broader evolutionary and ecological context of mangrove adaptation. We now highlight how ancient WGD, lineage-specific gene family evolution, and recent hybridization jointly shaped the evolutionary trajectory of Acanthus. We have also added a conclusion and future research directions at the end of the discussion.

Reviewer #2:
This study presents valuable new genome assemblies for three Acanthus mangrove species, employed to investigate their evolutionary history—including an ancient whole-genome duplication (WGD) event and a recent allopolyploidization—as well as adaptive gene family evolution and population structure. While the breadth of analyses is commendable, the current work lacks sufficient analytical depth and a cohesive narrative. Connections between distinct analytical components are often unclear, and comparative genomic analyses, in particular, require more thorough investigation.
Major points:
In Line 285, the Ai-Ae comparison exhibits a peak trend opposite to that of the At-At comparison. Further analysis is advised to elucidate the underlying reasons for this discrepancy.
Response:
We thank the reviewer for this valuable suggestion. In the original analysis, Ks distributions were estimated using Gaussian kernel density estimation (KDE). In the revised version, we replotted the Ks histograms and applied Gaussian mixture modeling (GMM) to visualize the distributions. The updated distribution plots have been updated in Fig. 3. In these new plots, the peak patterns in the Ai–Ae and At–At comparisons now follow the same trend. The subsection describing the Ks analysis has been reorganized in lines 338–359 (line numbers in the revised MS word file may differ from those in the generated PDF) of the revised manuscript.
In Lines 299 and 302, the two noted sets of Ks-related genes should be explicitly identified and screened. I recommend analyzing whether their distribution within each genome is random and investigating whether they exhibit significant enrichment in KEGG or GO functional categories.
Response:
We thank the reviewer for this valuable suggestion. As noted by another reviewer, we reanalyzed the Ks distribution and found that the minor peaks near Ks ≈ 0 resulted from tandem duplication. After applying tandem duplication filtering, these minor peaks disappeared. We, therefore, removed the text mentioning these peaks in the revised manuscript.
In Line 326, since the 176 genes annotated in this region do not derive from any ancestral genome sequence, it is critical to discuss the potential origin of this genomic region. Additionally, 105 of these 176 genes belong to three gene families; please elucidate the functions of these families. The results suggest this region may have arisen via tandem or segmental duplication, and further in-depth analysis of its origin and the characteristics of its constituent genes is recommended.

Response:
We thank the reviewer for this insightful comment. In the revised manuscript, we clarified that the 176 genes in the extended region of chromosome 20B do not form any collinearity blocks with the progenitor genomes but likely originated through segmental duplication, supported by the detection of multiple internal collinearity blocks. Among the three gene families identified, we visualized the gene tree of the largest family, which showed 33 paralogous gene copies in A. tetraploideus that were orthologous to a single gene in A. ebracteatus. Similar duplication patterns were also observed in L. racemosa, C. micranthum, and A. paniculata within the same tree. We have created new Fig. 4 combining the gene retention analysis results previously shown in Fig. 3C with this gene tree. Consistently, previous studies have shown that local duplications often contribute to genome expansion and structural variation in polyploids (Qiao et al., 2019). BLAST searches of these 105 proteins revealed matches to proteins from multiple plant species, which were annotated as uncharacterized or hypothetical. Further investigation is needed in the future to clarify their functions and evolutionary significance. The results have been revised accordingly in lines 378-392.
References
Qiao X. et al. (2019). Gene duplication and evolution in recurring polyploidization–diploidization cycles in plants. Genome Biology, 20: 38. https://doi.org/10.1186/s13059-019-1650-2
(Reference numbers 59 in the revised manuscript.)
In Line 359, the explanation that inconsistent divergence times (between the tetraploid's subgenomes and their diploid progenitors) stem from differences in geographic distribution is overly simplistic. Specific underlying mechanisms must be elaborated. For example, investigate whether this discrepancy could arise from post-formation gene introgression via backcrossing between the allopolyploid and its diploid ancestors.
Response:
We thank the reviewer for this valuable suggestion. The collection sources of our materials (A. ilicifolius from the Andaman coast and A. ebracteatus and A. tetraploideus from the Gulf of Thailand), together with the observed genetic separation between Andaman and Gulf populations, suggest a link between their geographic distributions and evolutionary divergence. We hypothesize that the sequenced A. ilicifolius individual from the Andaman coast may have diverged substantially from the ancestral A. ilicifolius lineage that contributed as SG1 in A. tetraploideus around ~5.7 Mya. In contrast, A. ebracteatus and A. tetraploideus still co-occur in the Gulf of Thailand and may therefore have been subjected to more similar environmental conditions. Consequently, the divergence between A. ilicifolius and SG1 appears greater than that between A. ebracteatus and SG2.
We agree that geographic separation alone cannot fully explain this pattern. However, our current dataset, which lacks A. ilicifolius genome data from the Gulf of Thailand, does not permit a formal test of introgression. In Response: to the reviewer's suggestion, we have clarified in the revised manuscript that spatial overlap between A. ebracteatus and A. tetraploideus could have facilitated post-formation gene flow or backcrossing, potentially contributing to the lower divergence observed between SG2 and its diploid progenitor. The continued geographic proximity of these species may have promoted stronger genomic continuity or reduced divergence along the SG2 lineage. All these clarifications have been added to lines 424–434 in the revised manuscript.
In Line 364, the manuscript must clarify its SNP calling strategy in the Methods section, including details of the reference genome used, specific procedures, and all software parameters. The current variant calling approach appears methodologically flawed: if the allotetraploid A. tetraploideus genome was used as the reference, calling SNPs from diploid samples (A. ilicifolius and A. ebracteatus) would be highly problematic due to ambiguous read mapping between homeologous subgenomes, leading to unreliable variant data. Conversely, using a diploid genome as the reference would introduce severe mapping bias for tetraploid reads, as reads from the non-reference subgenome would fail to align properly, resulting in an incomplete and skewed SNP dataset. This methodology is thus not robust for this diploid-polyploid system, and the validity of downstream analyses is questionable.
Response:
We thank the reviewer for this insightful comment. In the revised manuscript, we have clarified the SNP calling strategy in a new subsection of the Methods (Lines 254–260). To avoid mapping bias in the diploid–polyploid system, RADseq reads from each species were mapped to their respective genome assemblies, and SNPs were called independently for each population. This strategy minimized cross-subgenome misalignment and ensured reliable variant detection across both diploid and tetraploid genomes.
The population structure analysis is also described in detail in the revised Methods (Lines 261–267),

including the use of STRUCTURE v2.3.4 with 20 replicates for K = 1–10, 100,000 burn-in steps, and 500,000 MCMC iterations under an admixture model. The optimal K = 2 was determined using the ΔK method implemented in Structure Harvester, revealing two major genetic clusters corresponding to the Andaman and Gulf of Thailand coasts.

In Line 377, supporting scientific literature must be provided to validate the use of TE divergence rates for dating subgenome merger and divergence events. The current approach— inferring the timing of this event solely from the intersection of two kernel density estimates of LTR retrotransposon divergence in Supplementary Fig. S3—lacks sufficient theoretical and methodological justification. Pinpointing this intersection as the definitive merger time is an overreach, as it is not adequately supported by a single data feature and requires validation via established methods.

Response:

We appreciate the reviewer’s comment. The age of the hybridization event leading to the allotetraploid A. tetraploideus was inferred from transposable element (TE) divergence profiles, following the approach of Xu et al. (2019). This method was also used by Wang et al. (2024) to estimate the timing of polyploidization and hybridization events in mangrove evolution. Both studies compared the divergence distributions of LTR retrotransposons between parental subgenomes, showing that the initial separation of TE divergence distributions corresponds to the substitution rate at progenitor divergence, while their subsequent convergence reflects the substitution rate at genome merger. This approach complements Ks-based dating, particularly for the hybridization event where corresponding Ks values were unavailable. Following this framework, we compared the TE divergence profiles of SG1 and SG2 in A. tetraploideus and applied the same calibration method to estimate the timing of the genome merger event. For clarity, we have revised the Methods (lines 203–228) and Results (lines 446–453) in the revised manuscript. Supplementary Fig. S3 has been renumbered as Supplementary Fig. S1 in the revised manuscript.

References:

Xu P, Xu J, Liu G, Chen L, Zhou Z, Peng W, et al.. The allotetraploid origin and asymmetrical genome evolution of the common carp Cyprinus carpio. Nat Commun. Nature Publishing Group; 2019; doi: 10.1038/s41467-019-12644-1.

Wang Y, Li Y, Wu W, Shao S, Fang Q, Xu S, et al.. The evolution history of an allotetraploid mangrove tree analysed with a new tool Allo4D. Plant Biotechnology Journal. 2024; doi: 10.1111/pbi.14281.

(Reference numbers 47 and 48 in the revised manuscript.)

In Line 452, the Discussion section is overly verbose and requires restructuring. It tends to summarize results rather than interpret their implications. Please rewrite this section to be more focused, logical, and analytical, with clear explanations of the significance and context of your findings.

Response:

We thank the reviewers for these valuable suggestions. In lines 516-585 of the revised manuscript, the Discussion section has been restructured to improve clarity, focus, and logical flow. Instead of repeating results, the revised text emphasizes the biological significance of our findings and situates them within the broader evolutionary and ecological context of mangrove adaptation. We now highlight how ancient WGD, lineage-specific gene family evolution, and recent hybridization jointly shaped the evolutionary trajectory of Acanthus. We have also added a conclusion and future research directions at the end of the discussion.

Minor points:

In Line 217, please explicitly indicate which table reports the values "a total contig length of 1.95 Gb" and "a contig N50 of 42.67 Mb."

Response:

We appreciate the reviewer’s comment. These values correspond to the preliminary PacBio HiFi assembly, which served as the template contigs for Hi-C scaffolding. Therefore, they were not presented in a separate table but are directly reported in the text.

The numbering of Supplementary Table S2 and all subsequent supplementary tables appears incorrect; please carefully review and revise table numbering accordingly.

Response:

We appreciate the reviewer’s comment. We have carefully reviewed and corrected the numbering of Supplementary Table S2 and all subsequent supplementary tables throughout the manuscript and supporting files.

In Lines 220, 223, 231, and 247, citations to specific panels of Fig. 1 are misaligned with the text. Please revise figure citations to ensure the correct figure components are referenced in their respective contexts.

Response:

We thank the reviewer for this careful observation. In the revised manuscript, we have verified and

corrected the correspondence between figure citations and text. Specifically, we revised the citations of Fig. 1B (line 276), Fig. 1C (lines 279 and 287), and Fig. 1D (lines 298 and 309) in the revised manuscript.
In Line 224, Supplementary Table S4 reports BUSCO completeness at the genome level under genome mode, which does not reflect gene annotation completeness. Please provide an additional BUSCO assessment based on predicted protein sequences of all annotated genes to evaluate gene annotation quality.
Response:
We thank the reviewer for this helpful suggestion. We have performed an additional BUSCO assessment in protein mode using the predicted protein sequences of all annotated genes. The results have been added in lines 304–308 of the revised manuscript and were highly consistent with those obtained from the BUSCO analysis in genome mode.
In Line 336, for the phylogenetic tree in Fig. 4A, please use distinct colored blocks to indicate the clade, order, and family of each analyzed species.
Response:
We thank the reviewer for this helpful suggestion. We have added distinct colored blocks to the phylogenetic tree to indicate the clade, order, and family of each analyzed species. In the revised manuscript, the phylogenetic tree has been renumbered from Fig. 5 (renumbered from Fig. 4).
In Line 351, please relocate mathematical formulas to the Methods section for detailed explanation and clarification.
Response:
We appreciate the reviewer's comment. The corresponding equation has been relocated to the new subsection titled "7. Estimation of the timing of whole-genome duplication and hybridization events" in the Methods section for detailed explanation and clarification (Lines 203–228).
In Line 431, please provide detailed analytical procedures of the machine learning approach in the Methods section.
Response:
We thank the reviewer for this helpful suggestion. In the revised manuscript, we have expanded the Methods section to include a detailed description of the machine learning workflow, covering data inputs, phylogenetic filtering, feature selection, and model implementation. To enhance clarity and accessibility, this content has been organized into a new subsection titled "Gene family expansion/contraction analysis and machine learning-based selection of lineage-specific gene families" (Lines 235–253).
In Line 441, add a citation for Table 1 in the main text.
Response:
We thank the reviewer for the suggestion. A citation for Table 1 has been added to Line 482 of the revised manuscript.

Editorial Manager®

Cookie Preference Center

We use cookies which are necessary to make our site work. We may also use additional cookies to analyze, improve and personalize our content and your digital experience. For more information, see our Cookie Policy.

You may choose not to allow some types of cookies. However, blocking some types may impact your experience of our site and the services we are able to offer. See the different category headings below to find out more or change your settings.

Allow all

Manage Consent Preferences

Strictly Necessary Cookies

Always active

These cookies are necessary for the website to function and cannot be switched off in our systems. They are usually only set in response to actions made by you which amount to a request for services, such as setting your privacy preferences, logging in or filling in forms. You can set your browser to block or alert you about these cookies, but some parts of the site will not then work.

Functional Cookies

These cookies enable the website to provide enhanced functionality and personalisation. They may be set by us or by third party providers whose services we have added to our pages. If you do not allow these cookies then some or all of these services may not function properly.

Performance Cookies

These cookies allow us to count visits and traffic sources so we can measure and improve the performance of our site. They help us to know which pages are the most and least popular and see how visitors move around the site.

Targeting Cookies

These cookies may be set through our site by our advertising partners. They may be used by those companies to build a profile of your interests and show you relevant adverts on other sites. If you do not allow these cookies, you will experience less targeted advertising.

Cookie List

Clear

Apply Cancel

Consent Leg.Interest

Confirm my choices

Powered by onetrust
