# Supplementary material for: Genome Assembly of Three Shrub Mangroves in the Genus Acanthus Reveals Two Polyploidy Events and Expansion of Genes Linked to Root Adaptation in Coastal Habitats
Source: Gigascience. 2026 Jan 2;15:giaf162. doi: 10.1093/gigascience/giaf162 (PMC12903786; doi:10.1093/gigascience/giaf162)
Supplement: giaf162_Authors_Response_To_Reviewer_Comments_Revision_1 [file giaf162_authors_response_to_reviewer_comments_revision_1.pdf]

# Author's Response To Reviewer Comments

Reviewer reports:
Reviewer #1: The current version of the manuscript shows significant improvement compared to the previously submitted ve
addressed the issues I raised. Here are some minor suggestions for further consideration:

1. In the RagTag homologous mapping section, a common issue is that minimap2 retains only the optimal alignment, especia
without fragmentation is performed. This can lead to conflicts between nested or overlapping contigs, resulting in the loss of
over-assembly at the chromosome ends is prone to occur. To ensure the accuracy of this section, it is recommended to add a
results of ragtag correct + ragtag scaffold and those using only ragtag scaffold. Furthermore, cross-validation with gene-leve
related species can be employed to solidify the accuracy of the mapping.
Response
RagTag was used solely to cluster and scaffold stLFR contigs rather than to reassemble them. The contigs were first generate
RagTag was subsequently applied only for ordering, orienting, and scaffolding using the SG1 and SG2 chromosome sequence
RagTag does not modify contig sequences but links them with gap placeholders where needed.
We have now added the assembly statistics before and after RagTag scaffolding in Supplementary Tables S5 and S6. RagTag
from 8.2 Mb to 40.5 Mb in A. ilicifolius and from 6.9 Mb to 36.4 Mb in A. ebracteatus (Supplementary Tables S5 and S6). 93%
assembly lengths were successfully incorporated into scaffolds for A. ilicifolius and A. ebracteatus, respectively (lines 301–30
The synteny comparison demonstrates strong collinearity between A. tetraploideus SG1 and A. ilicifolius, and between A. tetr
ebracteatus (Fig. 1D).

To directly address the reviewer's suggestion, we compared RagTag scaffold only versus correct + scaffold (table below).

| | Placed sequences | Placed bases (Mb) | contig number | min | max | N50 | L50 |
|---|---|---|---|---|---|---|---|
| Scaffolding | 2573 | 839.0 | 9835 | 1000 | 52672276 | 36,414,290 | 11 |
| Correction + scaffolding | 2670 | 834.8 | 10249 | 103 | 51419172 | 35,889,547 | 11 |

The correction step resulted in increased fragmentation (9,835 → 10,249), a modest reduction in N50 (36.4 Mb → 35.8 Mb),
bases (839 Mb → 835 Mb). For this reason, we chose to retain the RagTag scaffold version.
2. In the section on constructing phylogenetic trees using single-copy genes, I previously mentioned that 70 single-copy gene
the number of single-copy genes and the stability of tree construction can be improved by utilizing conserved single-copy ger
It is recommended to give this approach a try.
Response
We appreciate the reviewer's suggestion. We initially constructed a phylogenetic tree using BUSCO single-copy genes. Howev
topology misplaced Rhizophora and Bruguiera (Fig. A below), which contradicted previously published phylogenies that includ
C below). In contrast, the OrthoFinder-based tree inferred from single-copy orthogroups yielded a topology fully consistent w
Therefore, we retained the OrthoFinder-based tree as the final phylogenetic reconstruction in this study.

Figure A Phylogenetic tree from BUSCO genes. Rhizophora and Bruguiera species are shown in blue and red. (please see figu
Response_to_reviewers_round2.pdf file)

Figure B Phylogenetic tree from the study by Xu S, et al. in National Science Review. 2017; doi: 10.1093/nsr/nwx065.

Figure C Phylogenetic tree from the study by Pootakham W, et al. in Molecular Ecology Resources. 2022; doi: 10.1111/1755-

##########################################################################################

Reviewer #2: The authors have addressed most of the issues raised in the previous review. However, several points still requ
additional analysis.

1. In line 185, please clarify how the 200-base-pair threshold was established. If this value is derived from previous work, su
Response
We thank the reviewer for raising this point. The 200-bp cutoff corresponds to the default proximity filter in the WGDI pipelin

ultra-proximal tandem duplicates while retaining all genuine collinear paralogs. Gene pairs occurring at such short physical di by recent tandem duplication events rather than representing true WGD-derived paralogs (Qiao et al., 2019). A clarification h manuscript lines 177-178.

Reference
59. Qiao X, Li Q, Yin H, Qi K, Li L, Wang R, et al.. Gene duplication and evolution in recurring polyploidization–diploidization c Biology. 2019; doi: 10.1186/s13059-019-1650-2.

2. In line 287, the last column of Supplementary Table S1 lacks a unit, which hampers clarity. Please add the appropriate uni should be carefully checked, and units must be explicitly provided wherever applicable.

Response

We thank the reviewer for pointing this out. The unit “megabase (Mb)” has now been added to the relevant column in Supple to Supplementary Tables S7 and S8. Units for count have been added to Supplementary Table S1 and to Supplementary Tabl S17. Percentage units have been added to Supplementary Table S11, and column categories have been added to Supplemen

3. In line 289, the contig shown at the bottom right of Fig. 2B that was not anchored to any chromosome must be clarified. P represents redundancy, contamination, or unanchored contigs. If it is redundant or contaminated, it should be removed and s figure. In addition, please provide high-resolution Hi-C contact maps for each individual chromosome as supplementary files, allow readers to adequately assess the quality of the chromosome-level assembly. For Table S2, please clarify the meaning o intuitively interpretable must be explicitly defined to avoid ambiguity and potential confusion for readers.

Response

We thank the reviewer for pointing this out. The Hi-C contact map is shown in Fig. 1B. The revised Fig. 1B now displays only The extra blocks have been removed, as only the chromosome sequences were used in downstream analyses. This clarificatio 144–145 of the revised manuscript. High-resolution Hi-C maps for each chromosome have been included as Supplementary F definition of auN has been added to Supplementary Tables S2, S5, and S6. Specifically, auN (area under the $N_x$ curve) is a le metric that summarizes the entire contig size distribution and reflects the expected contig length for a randomly selected bas a more contiguous assembly.

4. In line 293, the circos plot in Fig. 1C is unclear. Please specify exactly how many layers are included, label each layer with detailed explanation in the figure legend.

Response

We thank the reviewer for this suggestion. The circos plot in Fig. 1C has now been revised to clearly indicate each layer. Rom added to label the layers, and the figure legend has been updated to provide a detailed explanation of all included layers.

5. In line 320, the term "duplicated genes" (6%) is incorrect and must be corrected to "single-copy genes." Please ensure tha consistent throughout the manuscript.

Response

We thank the reviewer for identifying this error. The statement has been corrected in the revised manuscript to “with 92% cl 306–307), and all related text and tables have been updated for consistency.

6. In line 411, consistent with another reviewer's comment, the phylogenetic tree constructed using only 70 single-copy orth insufficient. Please refer to the 2025 Science paper "A genome-based phylogeny for Mollusca is concordant with fossils and m phylogenies based on BUSCO genes. Reconstruct the phylogeny using a similar approach and compare the topology with the robustness and stability.

Response

We appreciate the reviewer’s suggestion. We initially constructed a phylogenetic tree using BUSCO single-copy genes. Howev topology misplaced Rhizophora and Bruguiera (Fig. A below), which contradicted previously published phylogenies that includ C below). In contrast, the OrthoFinder-based tree inferred from single-copy orthogroups yielded a topology fully consistent w Therefore, we retained the OrthoFinder-based tree as the final phylogenetic reconstruction in this study.

Figure A Phylogenetic tree from BUSCO genes. Rhizophora and Bruguiera species are shown in blue and red. (please see figu Response_to_reviewers_round2.pdf file)

Figure B Phylogenetic tree from the study by Xu S, et al. in National Science Review. 2017; doi: 10.1093/nsr/nwx065.

Figure C Phylogenetic tree from the study by Pootakham W, et al. in Molecular Ecology Resources. 2022; doi: 10.1111/1755-

## Cookie Preference Center

We use cookies which are necessary to make our site work. We may also use additional cookies to analyze, improve and personalize our content and your digital experience. For more information, see our Cookie Policy.

You may choose not to allow some types of cookies. However, blocking some types may impact your experience of our site and the services we are able to offer. See the different category headings below to find out more or change your settings.

Allow all

## Manage Consent Preferences

### Strictly Necessary Cookies

**Always active**

These cookies are necessary for the website to function and cannot be switched off in our systems. They are usually only set in response to actions made by you which amount to a request for services, such as setting your privacy preferences, logging in or filling in forms. You can set your browser to block or alert you about these cookies, but some parts of the site will not then work.

### Functional Cookies

These cookies enable the website to provide enhanced functionality and personalisation. They may be set by us or by third party providers whose services we have added to our pages. If you do not allow these cookies then some or all of these services may not function properly.

### Performance Cookies

These cookies allow us to count visits and traffic sources so we can measure and improve the performance of our site. They help us to know which pages are the most and least popular and see how visitors move around the site.

**Targeting Cookies**

These cookies may be set through our site by our advertising partners. They may be used by those companies to build a profile of your interests and show you relevant adverts on other sites. If you do not allow these cookies, you will experience less targeted advertising.

**Cookie List**

Clear

Apply Cancel

Consent Leg.Interest

Confirm my choices

Powered by onetrust
