# Supplementary material for: Genome Assembly of Three Shrub Mangroves in the Genus Acanthus Reveals Two Polyploidy Events and Expansion of Genes Linked to Root Adaptation in Coastal Habitats
Source: Gigascience. 2026 Jan 2;15:giaf162. doi: 10.1093/gigascience/giaf162 (PMC12903786; doi:10.1093/gigascience/giaf162)
Supplement: giaf162_Reviewer_1_Report_Original_Submission — Minghui Kang -- 7/31/2025 [file giaf162_reviewer_1_report_original_submission.pdf]

Reviewer Report

Title: Genome Assembly of Three Shrub Mangroves in the Genus Acanthus Reveals Two Polyploidy Events and Expansion of Genes Linked to Root Adaptation in Coastal Habitats

Version: Original Submission **Date:** 7/31/2025

Reviewer name: Minghui Kang

Reviewer Comments to Author:

This paper makes significant contributions to the genome assembly and polyploidization analysis of the Acanthus genus, particularly in identifying adaptation-related genes in mangrove plants. The paper is well-structured, logically organized, and supported by rich figures and tables, which help readers understand the complex data and analysis results. However, there are still the following suggestions for this article:Ln 80 In the final paragraph of the introduction, please use the present tense, and ensure that the first occurrence of any abbreviation is accompanied by its full form in parentheses, such as stLFR (single-tube long fragment read), to facilitate reader comprehension. Additionally, please review the rest of the article for consistency.Ln 218 The estimated genome size in Supplementary Table S1 ranges from 1366 to 2496 Mb, which is a very wide range. Please specify which individual in the table corresponds to the assembled individual, as this is necessary to determine whether the assembled size matches the estimated genome size.Ln 220, Ln 223 Fig 1A shows a plant photograph and does not display genome-related data, while Fig 1B is a Hi-C contact heatmap, which also does not align with the description in the text. Please verify the correspondence between all figure captions, table captions, and the content of the article. Additionally, the high repeat density observed in the central regions of chromosomes does not necessarily correspond to centromeric regions; it is essential to confirm whether these regions contain centromeric repeat sequences.Ln 232 Is there any literature or experimental evidence supporting the identification of these two species as the putative progenitors? According to Supplementary Table 11, most of the alignment thresholds are below 75%. If there are other diploid species within this genus, it cannot be conclusively determined that these two species are the progenitors. The accuracy of homologous scaffolding is also questionable, which further casts doubt on the reliability of subsequent analyses.Ln 272 How were the WGD time and divergence time in Fig 2B obtained prior to the construction of the phylogenetic tree, and are they accurate, especially without 95% confidence interval? Additionally, how was the hybridization time between the two species calculated?Ln 293 Ln 299 Has the Ks peak been fitted and the corresponding peak range obtained, as this step significantly impacts the determination of the WGD timing? Additionally, Ks values very close to 0 are most likely caused by tandem duplications, which could result in the subsequent peaks being shifted forward. Has filtering been applied to remove these tandem duplications?Ln 322 What is the biological significance of sequence modifications, and are the genomic differences between the two species and the two subgenomes related to environmental adaptation and trait variation?Ln 334 The use of only 70 single-copy genes is notably insufficient and may significantly impact the stability of the phylogenetic tree's topology. It is recommended to replace some species and the outgroup. Additionally, neither the divergence time nor the WGD time is accompanied by a 95% confidence

interval. Has bootstrap validation been performed to confirm their accuracy?Ln 353 According to this formula, the WGD time is significantly influenced by the calculated divergence time. Has it been corrected based on the substitution rate?Ln 364 Does the CV value support K=2 as the optimal choice?Ln 416, Ln 432 Is the expansion and contraction of gene families related to the medicinal properties and floral trait differences mentioned in the abstract? Additionally, what specific machine learning methods were used, and what parameters and code were applied?Ln 452 The discussion section could be more concise to avoid repetition of results. Could it focus more on the biological significance and future research directions?Are all the codes and scripts used for analysis provided? This is crucial for the reproducibility of the study.

Level of Interest

Please indicate how interesting you found the manuscript: Choose an item.

Quality of Written English

Please indicate the quality of language in the manuscript: Choose an item.

Declaration of Competing Interests

Please complete a declaration of competing interests, considering the following questions:

- Have you in the past five years received reimbursements, fees, funding, or salary from an organisation that may in any way gain or lose financially from the publication of this manuscript, either now or in the future?
- Do you hold any stocks or shares in an organisation that may in any way gain or lose financially from the publication of this manuscript, either now or in the future?
- Do you hold or are you currently applying for any patents relating to the content of the manuscript?
- Have you received reimbursements, fees, funding, or salary from an organization that holds or has applied for patents relating to the content of the manuscript?
- Do you have any other financial competing interests?
- Do you have any non-financial competing interests in relation to this paper?

If you can answer no to all of the above, write 'I declare that I have no competing interests' below. If your reply is yes to any, please give details below.

I declare that I have no competing interests

I agree to the open peer review policy of the journal. I understand that my name will be included on my report to the authors and, if the manuscript is accepted for publication, my named report including any attachments I upload will be posted on the website along with the authors' responses. I agree for my

report to be made available under an Open Access Creative Commons CC-BY license (http://creativecommons.org/licenses/by/4.0/). I understand that any comments which I do not wish to be included in my named report can be included as confidential comments to the editors, which will not be published.

Choose an item.

To further support our reviewers, we have joined with Publons, where you can gain additional credit to further highlight your hard work (see: https://publons.com/journal/530/gigascience). On publication of this paper, your review will be automatically added to Publons, you can then choose whether or not to claim your Publons credit. I understand this statement.

Yes Choose an item.
