# Supplementary material for: Genome Assembly of Three Shrub Mangroves in the Genus Acanthus Reveals Two Polyploidy Events and Expansion of Genes Linked to Root Adaptation in Coastal Habitats
Source: Gigascience. 2026 Jan 2;15:giaf162. doi: 10.1093/gigascience/giaf162 (PMC12903786; doi:10.1093/gigascience/giaf162)
Supplement: giaf162_Reviewer_1_Report_Revision_1 — Minghui Kang -- 11/14/2025 [file giaf162_reviewer_1_report_revision_1.pdf]

Reviewer Report

Title: Genome Assembly of Three Shrub Mangroves in the Genus Acanthus Reveals Two Polyploidy Events and Expansion of Genes Linked to Root Adaptation in Coastal Habitats

Version: Revision 1 **Date:** 11/14/2025

Reviewer name: Minghui Kang

Reviewer Comments to Author:

The current version of the manuscript shows significant improvement compared to the previously submitted version, and it has essentially addressed the issues I raised. Here are some minor suggestions for further consideration:1. In the RagTag homologous mapping section, a common issue is that minimap2 retains only the optimal alignment, especially when only concatenation without fragmentation is performed. This can lead to conflicts between nested or overlapping contigs, resulting in the loss of some sequences. Additionally, over-assembly at the chromosome ends is prone to occur. To ensure the accuracy of this section, it is recommended to add a comparison between the results of ragtag correct + ragtag scaffold and those using only ragtag scaffold. Furthermore, cross-validation with gene-level synteny results from closely related species can be employed to solidify the accuracy of the mapping.2. In the section on constructing phylogenetic trees using single-copy genes, I previously mentioned that 70 single-copy genes are relatively few. In fact, the number of single-copy genes and the stability of tree construction can be improved by utilizing conserved single-copy genes from the BUSCO dataset. It is recommended to give this approach a try.

Level of Interest

Please indicate how interesting you found the manuscript: Choose an item.

Quality of Written English

Please indicate the quality of language in the manuscript: Choose an item.

Declaration of Competing Interests

Please complete a declaration of competing interests, considering the following questions:

- Have you in the past five years received reimbursements, fees, funding, or salary from an organisation that may in any way gain or lose financially from the publication of this manuscript, either now or in the future?
- Do you hold any stocks or shares in an organisation that may in any way gain or lose financially from the publication of this manuscript, either now or in the future?
- Do you hold or are you currently applying for any patents relating to the content of the manuscript?

- Have you received reimbursements, fees, funding, or salary from an organization that holds or has applied for patents relating to the content of the manuscript?
- Do you have any other financial competing interests?
- Do you have any non-financial competing interests in relation to this paper?

If you can answer no to all of the above, write 'I declare that I have no competing interests' below. If your reply is yes to any, please give details below.

I declare that I have no competing interests.

I agree to the open peer review policy of the journal. I understand that my name will be included on my report to the authors and, if the manuscript is accepted for publication, my named report including any attachments I upload will be posted on the website along with the authors' responses. I agree for my report to be made available under an Open Access Creative Commons CC-BY license (http://creativecommons.org/licenses/by/4.0/). I understand that any comments which I do not wish to be included in my named report can be included as confidential comments to the editors, which will not be published.

Choose an item.

To further support our reviewers, we have joined with Publons, where you can gain additional credit to further highlight your hard work (see: https://publons.com/journal/530/gigascience). On publication of this paper, your review will be automatically added to Publons, you can then choose whether or not to claim your Publons credit. I understand this statement.

Yes Choose an item.
