# Supplementary material for: Genome Assembly of Three Shrub Mangroves in the Genus Acanthus Reveals Two Polyploidy Events and Expansion of Genes Linked to Root Adaptation in Coastal Habitats
Source: Gigascience. 2026 Jan 2;15:giaf162. doi: 10.1093/gigascience/giaf162 (PMC12903786; doi:10.1093/gigascience/giaf162)
Supplement: giaf162_Reviewer_2_Report_Original_Submission — Zhe Liang -- 8/5/2025 [file giaf162_reviewer_2_report_original_submission.pdf]

Reviewer Report

Title: Genome Assembly of Three Shrub Mangroves in the Genus Acanthus Reveals Two Polyploidy Events and Expansion of Genes Linked to Root Adaptation in Coastal Habitats

Version: Original Submission **Date:** 8/5/2025

Reviewer name: Zhe Liang

Reviewer Comments to Author:

This study presents valuable new genome assemblies for three Acanthus mangrove species, employed to investigate their evolutionary historyâ€”including an ancient whole-genome duplication (WGD) event and a recent allopolyploidizationâ€"as well as adaptive gene family evolution and population structure. While the breadth of analyses is commendable, the current work lacks sufficient analytical depth and a cohesive narrative. Connections between distinct analytical components are often unclear, and comparative genomic analyses, in particular, require more thorough investigation.Major points:1. In Line 285, the Ai-Ae comparison exhibits a peak trend opposite to that of the At-At comparison. Further analysis is advised to elucidate the underlying reasons for this discrepancy.2. In Lines 299 and 302, the two noted sets of Ks-related genes should be explicitly identified and screened. I recommend analyzing whether their distribution within each genome is random and investigating whether they exhibit significant enrichment in KEGG or GO functional categories.3. In Line 326, since the 176 genes annotated in this region do not derive from any ancestral genome sequence, it is critical to discuss the potential origin of this genomic region. Additionally, 105 of these 176 genes belong to three gene families; please elucidate the functions of these families. The results suggest this region may have arisen via tandem or segmental duplication, and further in-depth analysis of its origin and the characteristics of its constituent genes is recommended.4. In Line 359, the explanation that inconsistent divergence times (between the tetraploid's subgenomes and their diploid progenitors) stem from differences in geographic distribution is overly simplistic. Specific underlying mechanisms must be elaborated. For example, investigate whether this discrepancy could arise from post-formation gene introgression via backcrossing between the allopolyploid and its diploid ancestors.5. In Line 364, the manuscript must clarify its SNP calling strategy in the Methods section, including details of the reference genome used, specific procedures, and all software parameters. The current variant calling approach appears methodologically flawed: if the allotetraploid A. tetraploideus genome was used as the reference, calling SNPs from diploid samples (A. ilicifolius and A. ebracteatus) would be highly problematic due to ambiguous read mapping between homeologous subgenomes, leading to unreliable variant data. Conversely, using a diploid genome as the reference would introduce severe mapping bias for tetraploid reads, as reads from the non-reference subgenome would fail to align properly, resulting in an incomplete and skewed SNP dataset. This methodology is thus not robust for this diploid-polyploid system, and the validity of downstream analyses is questionable.6. In Line 377, supporting scientific literature must be provided to validate the use of TE divergence rates for dating subgenome merger and divergence events. The current approachâ€" inferring the timing of this event solely from the intersection of two kernel density estimates of LTR retrotransposon divergence in Supplementary Fig.

S3â€"lacks sufficient theoretical and methodological justification. Pinpointing this intersection as the definitive merger time is an overreach, as it is not adequately supported by a single data feature and requires validation via established methods.7. In Line 452, the Discussion section is overly verbose and requires restructuring. It tends to summarize results rather than interpret their implications. Please rewrite this section to be more focused, logical, and analytical, with clear explanations of the significance and context of your findings.Minor points:1. In Line 217, please explicitly indicate which table reports the values "a total contig length of 1.95 Gb" and "a contig N50 of 42.67 Mb."2. The numbering of Supplementary Table S2 and all subsequent supplementary tables appears incorrect; please carefully review and revise table numbering accordingly.3. In Lines 220, 223, 231, and 247, citations to specific panels of Fig. 1 are misaligned with the text. Please revise figure citations to ensure the correct figure components are referenced in their respective contexts.4. In Line 224, Supplementary Table S4 reports BUSCO completeness at the genome level under genome mode, which does not reflect gene annotation completeness. Please provide an additional BUSCO assessment based on predicted protein sequences of all annotated genes to evaluate gene annotation quality.5. In Line 336, for the phylogenetic tree in Fig. 4A, please use distinct colored blocks to indicate the clade, order, and family of each analyzed species.6. In Line 351, please relocate mathematical formulas to the Methods section for detailed explanation and clarification.7. In Line 431, please provide detailed analytical procedures of the machine learning approach in the Methods section.8. In Line 441, add a citation for Table 1 in the main text.

Level of Interest

Please indicate how interesting you found the manuscript: Choose an item.

Quality of Written English

Please indicate the quality of language in the manuscript: Choose an item.

Declaration of Competing Interests

Please complete a declaration of competing interests, considering the following questions:

- Have you in the past five years received reimbursements, fees, funding, or salary from an organisation that may in any way gain or lose financially from the publication of this manuscript, either now or in the future?
- Do you hold any stocks or shares in an organisation that may in any way gain or lose financially from the publication of this manuscript, either now or in the future?
- Do you hold or are you currently applying for any patents relating to the content of the manuscript?
- Have you received reimbursements, fees, funding, or salary from an organization that holds or has applied for patents relating to the content of the manuscript?
- Do you have any other financial competing interests?

- Do you have any non-financial competing interests in relation to this paper?

If you can answer no to all of the above, write 'I declare that I have no competing interests' below. If your reply is yes to any, please give details below.

I declare that I have no competing interests.

I agree to the open peer review policy of the journal. I understand that my name will be included on my report to the authors and, if the manuscript is accepted for publication, my named report including any attachments I upload will be posted on the website along with the authors' responses. I agree for my report to be made available under an Open Access Creative Commons CC-BY license (http://creativecommons.org/licenses/by/4.0/). I understand that any comments which I do not wish to be included in my named report can be included as confidential comments to the editors, which will not be published.

Choose an item.

To further support our reviewers, we have joined with Publons, where you can gain additional credit to further highlight your hard work (see: https://publons.com/journal/530/gigascience). On publication of this paper, your review will be automatically added to Publons, you can then choose whether or not to claim your Publons credit. I understand this statement.

Yes Choose an item.
