# Supplementary material for: Genome Assembly of Three Shrub Mangroves in the Genus Acanthus Reveals Two Polyploidy Events and Expansion of Genes Linked to Root Adaptation in Coastal Habitats
Source: Gigascience. 2026 Jan 2;15:giaf162. doi: 10.1093/gigascience/giaf162 (PMC12903786; doi:10.1093/gigascience/giaf162)
Supplement: giaf162_Reviewer_2_Report_Revision_1 — Zhe Liang -- 11/21/2025 [file giaf162_reviewer_2_report_revision_1.pdf]

Reviewer Report

Title: Genome Assembly of Three Shrub Mangroves in the Genus Acanthus Reveals Two Polyploidy Events and Expansion of Genes Linked to Root Adaptation in Coastal Habitats

Version: Revision 1 **Date:** 11/21/2025

Reviewer name: Zhe Liang

Reviewer Comments to Author:

The authors have addressed most of the issues raised in the previous review. However, several points still require further clarification and additional analysis.1. In line 185, please clarify how the 200-base-pair threshold was established. If this value is derived from previous work, supply the relevant references.2. In line 287, the last column of Supplementary Table S1 lacks a unit, which hampers clarity. Please add the appropriate unit. In addition, all other tables should be carefully checked, and units must be explicitly provided wherever applicable.3. In line 289, the contig shown at the bottom right of Fig. 2B that was not anchored to any chromosome must be clarified. Please confirm whether it represents redundancy, contamination, or unanchored contigs. If it is redundant or contaminated, it should be removed and should not appear in the figure. In addition, please provide high-resolution Hi-C contact maps for each individual chromosome as supplementary files, as the current figure does not allow readers to adequately assess the quality of the chromosome-level assembly. For Table S2, please clarify the meaning of "auN." Terms that are not intuitively interpretable must be explicitly defined to avoid ambiguity and potential confusion for readers.4. In line 293, the circos plot in Fig. 1C is unclear. Please specify exactly how many layers are included, label each layer with letters, and provide a detailed explanation in the figure legend.5. In line 320, the term "duplicated genes" (6%) is incorrect and must be corrected to "single-copy genes." Please ensure that all text and table data are consistent throughout the manuscript.6. In line 411, consistent with another reviewer's comment, the phylogenetic tree constructed using only 70 single-copy orthologous genes is notably insufficient. Please refer to the 2025 Science paper "A genome-based phylogeny for Mollusca is concordant with fossils and morphology," which constructs phylogenies based on BUSCO genes. Reconstruct the phylogeny using a similar approach and compare the topology with the current tree to assess its robustness and stability.

Level of Interest

Please indicate how interesting you found the manuscript: Choose an item.

Quality of Written English

Please indicate the quality of language in the manuscript: Choose an item.

Declaration of Competing Interests

Please complete a declaration of competing interests, considering the following questions:

- Have you in the past five years received reimbursements, fees, funding, or salary from an organisation that may in any way gain or lose financially from the publication of this manuscript, either now or in the future?
- Do you hold any stocks or shares in an organisation that may in any way gain or lose financially from the publication of this manuscript, either now or in the future?
- Do you hold or are you currently applying for any patents relating to the content of the manuscript?
- Have you received reimbursements, fees, funding, or salary from an organization that holds or has applied for patents relating to the content of the manuscript?
- Do you have any other financial competing interests?
- Do you have any non-financial competing interests in relation to this paper?

If you can answer no to all of the above, write 'I declare that I have no competing interests' below. If your reply is yes to any, please give details below.

I declare that I have no competing interests

I agree to the open peer review policy of the journal. I understand that my name will be included on my report to the authors and, if the manuscript is accepted for publication, my named report including any attachments I upload will be posted on the website along with the authors' responses. I agree for my report to be made available under an Open Access Creative Commons CC-BY license (http://creativecommons.org/licenses/by/4.0/). I understand that any comments which I do not wish to be included in my named report can be included as confidential comments to the editors, which will not be published.

Choose an item.

To further support our reviewers, we have joined with Publons, where you can gain additional credit to further highlight your hard work (see: https://publons.com/journal/530/gigascience). On publication of this paper, your review will be automatically added to Publons, you can then choose whether or not to claim your Publons credit. I understand this statement.

Yes Choose an item.
